# SET activation of nitroarenes by 2-azaallyl anions as a straightforward access to 2,5-dihydro-1,2,4-oxadiazoles

**DOI:** 10.1038/s41467-021-26767-x

**Published:** 2021-12-03

**Authors:** Dong Zou, Lishe Gan, Fan Yang, Huan Wang, Youge Pu, Jie Li, Patrick J. Walsh

**Affiliations:** 1 Department of Pharmacy, School of Medicine, Zhejiang University City College, No. 48, Huzhou Road, 310015 Hangzhou, P. R. China; 2 School of Biotechnology and Health Sciences, Wuyi University, 529020 Jiangmen, P. R. China; 3 Roy and Diana Vagelos Laboratories, Department of Chemistry, University of Pennsylvania, 231 South 34th Street, Philadelphia, PA 19104-6323 USA

**Keywords:** Synthetic chemistry methodology, Reaction mechanisms

## Abstract

The use of nitroarenes as amino sources in synthesis is challenging. Herein is reported an unusual, straightforward, and transition metal-free method for the net [3 + 2]-cycloaddition reaction of 2-azaallyl anions with nitroarenes. The products of this reaction are diverse 2,5-dihydro-1,2,4-oxadiazoles (>40 examples, up to 95% yield). This method does not require an external reductant to reduce nitroarenes, nor does it employ nitrosoarenes, which are often used in N–O cycloadditions. Instead, it is proposed that the 2-azaallyl anions, which behave as super electron donors (SEDs), deliver an electron to the nitroarene to generate a nitroarene radical anion. A downstream 2-azaallyl radical coupling with a newly formed nitrosoarene is followed by ring closure to afford the observed products. This proposed reaction pathway is supported by computational studies and experimental evidence. Overall, this method uses readily available materials, is green, and exhibits a broad scope.

## Abstract (web summary)

Oxadiazoles are five-membered ring heterocycles comprising an oxygen and two nitrogens, and those with the 1,2,4-oxadiazole skeleton are of interest due to their prevalence in biologically active compounds. Here, the authors report a transition metal-free [3+2]-cycloaddition of nitroarenes with 2-azaallyl anions under basic conditions to access 2,5-dihydro-1,2,4-oxadiazoles.

## Introduction

**Fig. 1:**
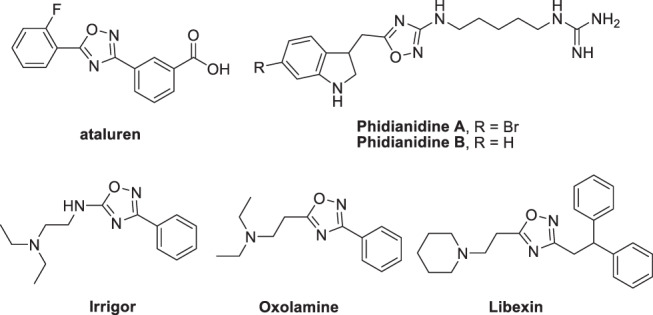
Some biologically important compounds containing 1,2,4-oxadiazole core.

Heterocyclic compounds are of great importance in the pharmaceutical and pesticide industries. Among heterocycles, oxadiazoles, five-membered ring heterocycles containing an oxygen and two nitrogens, and their derivatives, have attracted considerable interests^1–10^. Among oxadiazoles, those containing the 1,2,4-oxadiazole skeleton, including 2,5-dihydro-1,2,4-oxadiazoles, are of interest because of their prevalence in various biologically active compounds (Fig. 1). They have been found to exhibit antiviral^11^, anticancer^12,13^, anti-inflammatory^14^, antirhinovirus^15^, and antiparasitic properties^16–19^. They are also known as muscarinic agonists^20–22^, GABA modulators^23^, and benzodiazepine receptor agonists^24^.

**Fig. 2:**
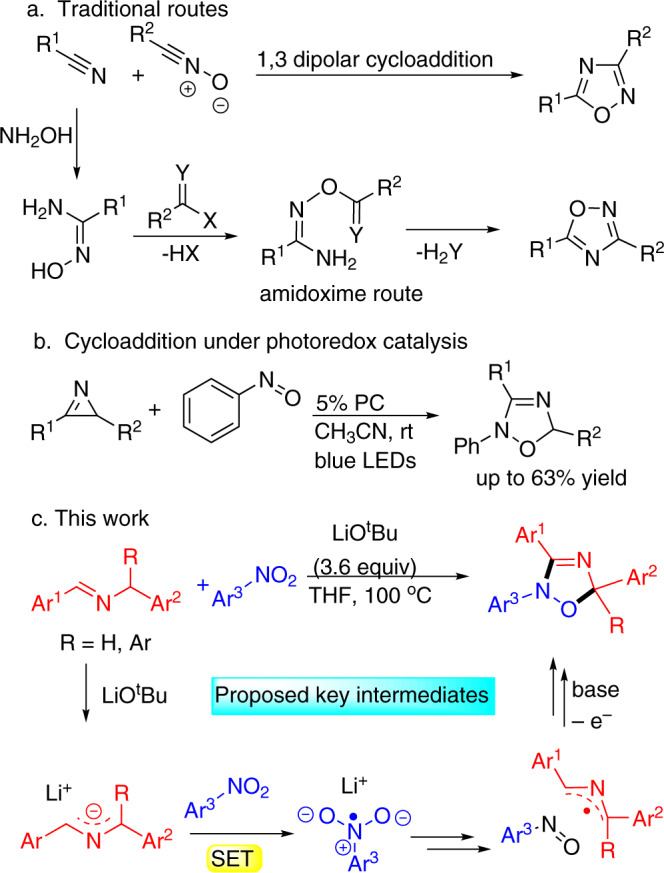
Synthetic methods for 1,2,4-oxadiazoles and 2,5-dihydro-1,2,4-oxadiazoles. **a** Traditional route oxadiazoles. **b** Cycloaddition under photoredox catalysis. **c** This work with key intermediates.

Due to the utility of 1,2,4-oxadiazoles in the pharmaceutical industry, their synthesis has received significant attention. Traditional methods for the synthesis of 1,2,4-oxadiazoles are largely based on two approaches (Fig. 2a): (1) 1,3-dipolar cycloaddition of nitriles to nitrile oxides and (2) thermally promoted cyclization of amidoxime derivatives^25,26^. Despite the popularity of these methods, they suffer from limited precursor availability. Recently, Xuan, Xiao, and co-workers developed a [3 + 2]-cycloaddition of 2H-azirines with nitrosoarenes under photoredox catalysis with visible light. In their study, a series of 2,5-dihydro-1,2,4-oxadiazole derivatives were prepared in moderate yields (up to 63%, Fig. 2b)^27^. A drawback of this, and related cycloadditions^28,29^, is the use of nitrosoarenes, which have very limited commercial availability and stability.

In contrast to nitrosoarenes, nitroarenes are feedstocks and represent one of the most readily available starting materials in organic synthesis. They are easily prepared from the parent arenes via Friedel–Crafts nitration and many are commercially available and inexpensive. Recent years have witnessed the use of nitroarenes as amino sources, often in transition metal catalyzed processes^30–37^. A more formidable challenge is the use of nitroarenes as amino precursors in the absence of transition metal catalysts^38–45^.

Herein, we report a transition-metal-free net [3 + 2]-cycloaddition of nitroarenes with 2-azaallyl anions under basic conditions to afford 2,5-dihydro-1,2,4-oxadiazoles in good to excellent yields (Fig. 2c). A unique reaction mechanism is proposed, wherein single electron transfer (SET) from the 2-azaallyl anion to the nitroarene eventually leads to a 2-azaallyl radical that couples with a newly formed nitrosoarene. Considering the significant role of nitroarenes in various aspects of modern chemistry, we envision that this straightforward method will be of interest in organic synthesis and medicinal chemistry.

## Results

### Background

**Fig. 3:**
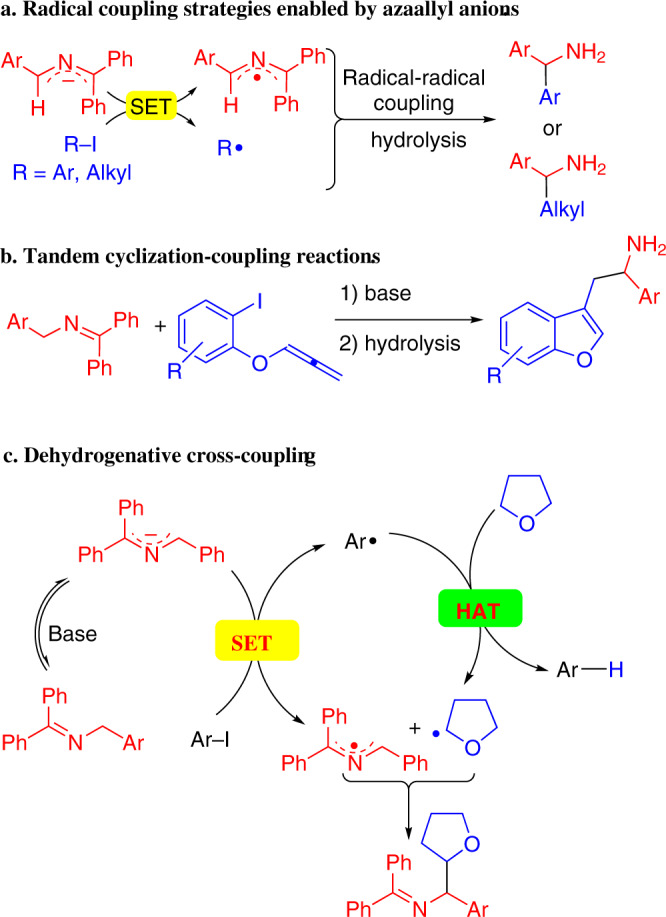
SET reactions with 2-azaallyl anions. **a** Transition-metal-free cross-coupling reactions. **b** Cyclization/radical-radical coupling processes for the synthesis of benzofurans. **c** Dehydrogenative cross-coupling reactions.

We have been interested in the fascinating chemistry of *N*-benzyl ketimines, which undergo deprotonation to give 2-azaallyl anions that exhibit umpolung reactivity^46^. In our initial investigations, we studied the use of 2-azaally anions and related pronucleophiles in palladium catalyzed cross-coupling reactions with aryl halides^47,48^. In these reactions, the 2-azaally anions were produced in situ from either aldimines or ketimines under basic reaction conditions. Subsequently, we found that 2-azaallyl anions behave as super electron donors (SEDs)^49^, as defined by Murphy and co-workers, and we characterized their reducing properties and the structures of the 2-azaallyl anions and radical^50^. The 2-azaallyl anions promote transition metal-free arylation with aryl iodides and alkylation with sterically encumbered alkyl iodides (Fig. 3a)^51–54^. They are synthetically useful for the synthesis of benzofurylethylamines (Fig. 3b) and their isochromene analogs^55,56^. In the presence of bulky aryl iodides, where the coupling in Fig. 3a is slow, cross-dehydrogenative coupling reactions take place. As shown in Fig. 3c, the aryl radical undergoes hydrogen atom transfer (HAT) with ethers (such as tetrahydrofuran (THF), shown), amines, or toluenes. Radical–radical coupling then leads to functionalized products^57^.

### Reaction development and optimization

In the present study, we were interested in capitalizing on the SED properties of 2-azaallyl anions and explored their reactivity with nitroarenes. We initiated our studies with nitrobenzene (**1a**) and *N*-benzylidene-1,1-diphenylmethanamine (**2a**), which leads to a 2-azaallyl anion upon deprotonation. For the deprotonation, seven bases were screened in THF [KO^*t*^Bu, NaO^*t*^Bu, LiO^*t*^Bu, LDA, KOH, NaOH, and LiOH] at 100 °C for 12 h (Table 1, entries 1–7). LDA and KOH caused decomposition of the aldimine and all reactions that did not generate product resulted in recovered nitroarene. Only NaOH and LiO^*t*^Bu were suitable bases for the desired transformation, giving the corresponding product **3aa** in 35% and 53% yields (entries 2 and 7), respectively. Testing a collection of solvents [1,4-dioxane, DME (dimethoxy ethane), toluene, and CPME (cyclopentyl methyl ether), Table 1, entries 8–11] indicated that the reaction in THF outperformed those in other solvents. Performing the reaction at 120 °C did not improve the yield (Table 1, entry 12), while only trace product was observed when the reaction was conducted at 80 °C. The excess amount of aldimine is essential for high conversion (Table 1, entries 14–17). The yield was elevated to 77% with two equiv. of imine **2a**, and 86% yield was obtained when the ratio of imine **2a** to nitrobenzene was 3:1. We note that the reaction at RT under blue light gave only 13% yield of the oxadiazole product after 24 h irradiation (entry 18). The structure of **3aa** was confirmed by X-ray diffraction (CCDC 2032323).

**Table 1:** Reaction optimization^a^.


Entry	Solvent	Base	Temp (°C)	1a:2a	Yield (%)^b^
1	THF	LDA	100 °C	1:1	—
2	THF	NaOH	100 °C	1:1	35
3	THF	KOH	100 °C	1:1	—
4	THF	LiOH	100 °C	1:1	—
5	THF	NaO^*t*^Bu	100 °C	1:1	—
6	THF	KO^*t*^Bu	100 °C	1:1	—
7	THF	LiO^*t*^Bu	100 °C	1:1	53
8	Dioxane	LiO^*t*^Bu	100 °C	1:1	39
9	DME	LiO^*t*^Bu	100 °C	1:1	46
10	Toluene	LiO^*t*^Bu	100 °C	1:1	—
11	CPME	LiO^*t*^Bu	100 °C	1:1	—
12	THF	LiO^*t*^Bu	120 °C	1:1	41
13	THF	LiO^*t*^Bu	80 °C	1:1	Trace
14^c^	THF	LiO^*t*^Bu	100 °C	1:2	77
15^d^	THF	LiO^*t*^Bu	100 °C	1:3	86
16^c^	THF	LiO^*t*^Bu	100 °C	2:1	61
17^d^	THF	LiO^*t*^Bu	100 °C	3:1	55
18^e^	THF	LiO^*t*^Bu	RT	1:3	13

^a^Reactions were conducted with **1a** (0.1 mmol), **2a** (0.1 mmol), base (0.12 mmol), solvent (1 mL), 12 h.

^b^Isolated yields.

^c^0.24 mmol of LiO^*t*^Bu.

^d^0.36 mmol of LiO^*t*^Bu.

^e^Under blue LED irradiation.

### Scope of the aldimine

**Fig. 4:**
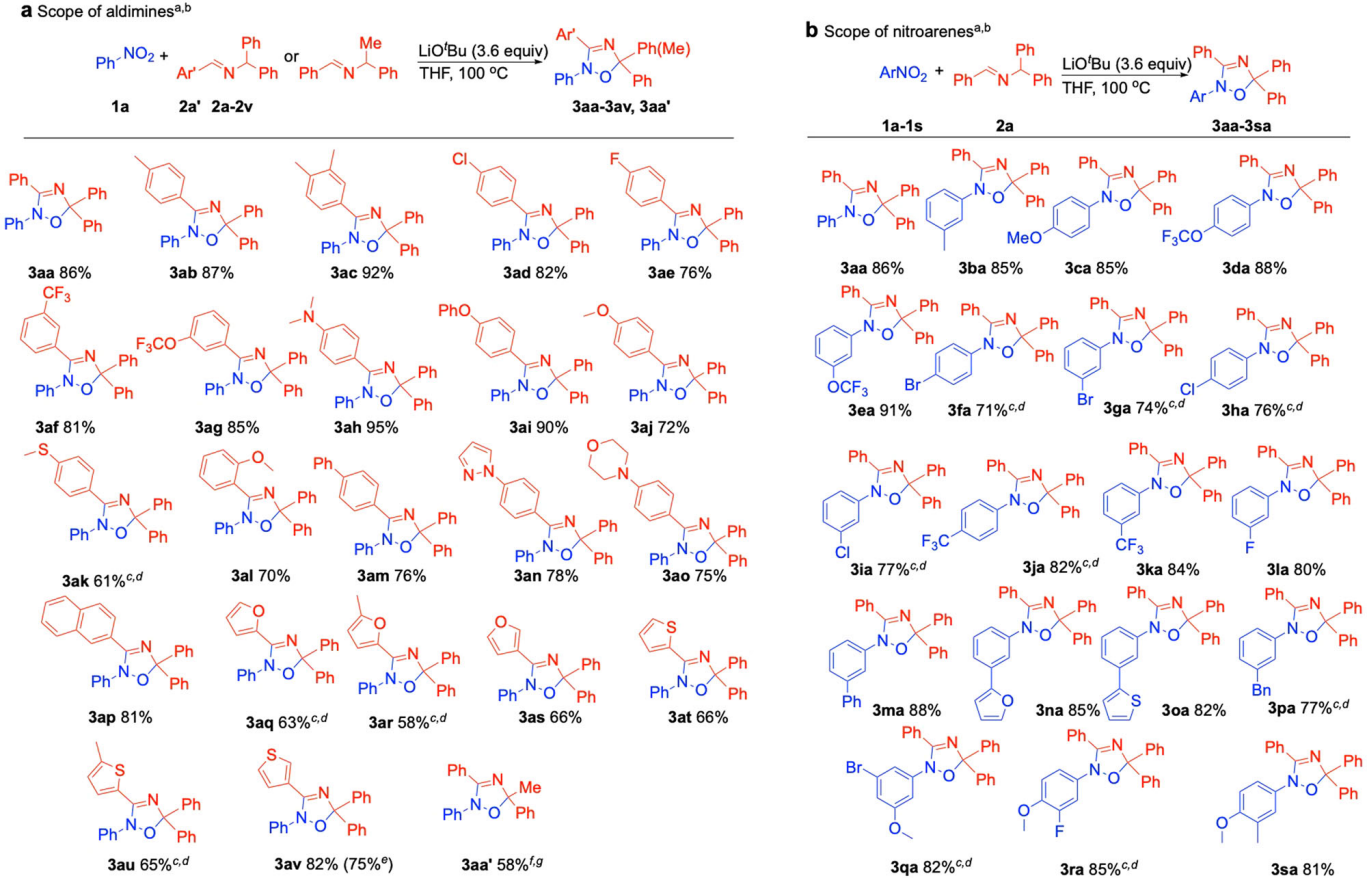
Substrate scope^a,b^. **a** Scope of the aldimine. **b** Scope of the nitroarene. ^a^Reaction conditions: nitroarene (0.1 mmol), aldimine (0.3 mmol), LiO^*t*^Bu (0.36 mmol), THF (0.1 M), 100 °C, 12 h. ^b^Isolated yield. ^c^0.2 mmol of aldimine. ^d^0.24 mmol of LiO^*t*^Bu. ^e^Reaction performed on 5 mmol scale. ^f^0.36 mmol of NaO^*t*^Bu. ^g^DME (0.1 M).

With the optimized reaction conditions in hand (Table 1, entry 15), the scope of the *N*-benzyl group of the aldimine partner was examined. As shown in Fig. 4, various aldimines bearing substituents on the *N*-benzyl group gave the desired products in moderate-to-good yields (58–95%). Aldimines possessing alkyl groups (4-Me and 3,4-Me_2_) gave products **3ab** and **3ac** in 87 and 92% yield, respectively. Substrates bearing electronegative groups and electron-withdrawing substituents (4-Cl, 4-F, 3-CF_3_, and 3-OCF_3_) underwent reaction in 76–85% yield to furnish the oxadiazole products (**3ad**, **3ae**, **3af**, and **3ag**). Electron-donating substrates (4-NMe_2_, 4-OPh, 4-OMe, 4-SMe, and 2-OMe) provided desired products (**3ah**, **3ai**, **3aj**, **3ak**, and **3al**) in 61–95% yield. Aldimines possessing biphenyl (**3am**), 4-pyrazolylphenyl (**3an**), 4-morpholinophenyl (**3ao**) and 2-naphthyl (**3ap**) groups were all tolerated in this protocol, furnishing the cycloaddition products in 75–81% yield. Additionally, aldimines containing heterocycles were also well tolerated. For example, 2-azaallyls bearing 2-furanyl, 3-furanyl, 2-thiofuranyl, and 3-thiofuranyl heteroarenes participated in this reaction, giving the products **3aq**–**3av** in 58–82% yield. Replacing one of the phenyl groups of **2a** with a methyl group to give **2a’**, the oxadiazole product (**3aa'**) was prepared in 58% yield with NaO^*t*^Bu as base. To illustrate the scalability of this method, we conducted the reaction of nitrobenzene (**1a**) with *N*-benzhydryl-1-(thiophen-3-yl)methanimine (**2v**) on a 5 mmol scale. The cyclized product **3av** was isolated in 75% yield (1.43 g).

### Scope of the nitroarene coupling partner

The range of nitroarene substrates was next explored. Nitroarenes bearing diverse substituents exhibited good to excellent reactivity, including those with alkyl (3-Me, **3ba**, 85% yield), electron-donating (4-OMe, **3ca**, 85% yield), and electronegative or electron-withdrawing substituents [4-OCF_3_ (**3da**), 3-OCF_3_ (**3ea**), 4-Br (**3fa**), 3-Br (**3ga**), 4-Cl (**3ha**), 3-Cl (**3ia**), 4-CF_3_ (**3ja**), 3-CF_3_ (**3ka**), and 3-F (**3la**), 71–91% yield]. Nitroarenes possessing 4-Ph (**3ma**), heterocyclic 2-furanyl and 2-thiofuranyl (**3na**, **3oa**), and benzyl (**3pa**) groups all worked well in this transformation (77–88% yield). In addition to monosubstituted nitroarenes, disubstituted nitroarenes such as 1-bromo-3-methoxy-5-nitrobenzene **1q**, 2-fluoro-1-methoxy-4-nitrobenzene **1r**, and 1-methoxy-2-methyl-4-nitrobenzene **1s** were also suitable substrates in this protocol, affording the cyclized products **3qa**–**3sa** in 81–85% yields. These substrates are primed for further functionalization through cross-coupling strategies. Overall, a variety of 2,5-dihydro-1,2,4-oxadiazoles were readily prepared by net [3 + 2] cycloaddition of simple nitroarenes with aldimines.

**Fig. 5:**
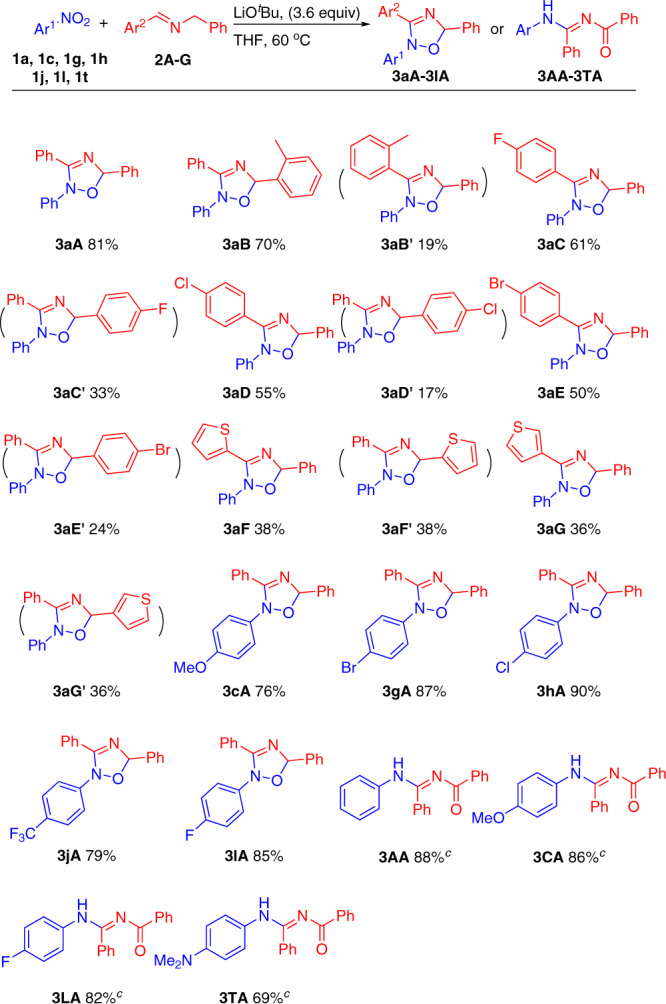
Substrate scope in the synthesis of triaryl-substituted oxadiazoles and ring-opening products^a,b^. ^a^Reaction conditions: nitroarene (0.1 mmol), **2a** (0.3 mmol), LiO^*t*^Bu (0.36 mmol), THF (0.1 M), 60 °C, 12 h. ^b^Isolated yield. ^c^100 °C.

In an effort to broaden the types of oxadiazoles accessible with this method, we targeted 1,3-diaryl 2-azaallyl anions with the goal of preparing triaryl-substituted oxadiazoles. As shown in Fig. 5, *N*-benzylidene-1-phenylmethanamine (**2A**) underwent reaction at 60 °C to afford **3aA** in 81% yield. We next wished to examine unsymmetrical 1,3-diaryl 2-azaallyl precursors to explore regioselectivity in this reaction. When the aldimine **2B**, prepared from 2-methyl benzaldehyde, was employed, the 2-tolyl group of the product was preferentially located in the sterically least hindered position with a regioisomeric ratio (rr) of 78: 22. The rr of all products was determined by ^1^H nuclear magnetic resonance (NMR) analysis of the crude reaction mixture. In the case of **3aB**, the major regioisomer was isolated by column chromatography in 70% and its assignment as the 5-tolyl derivative made based on heteronuclear multiple quantum coherence and heteronuclear multiple bond coherence experiments. In contrast, when aldimines **2C**–**E**, generated from 4-halo-benzaldehydes, were employed (halogen = F, Cl, Br) the opposite regioisomer was observed with rr = 35: 65 (X = F), 24: 76 (X = Cl) and 32: 68 (X = Br). The isolated yields of the major regioisomers were 61% (**3aC**), 55% (**3aD**), and 50% (**3aE**). The regiochemistry of the major regioisomer **3aE** was further confirmed through characterization by X-ray crystallography (CCDC 2068961).

A brief study of nitroarenes with the 1,3-diphenyl aldimine **2A** was performed. Nitroarenes bearing electron-donating (4-OMe), electronegative (4-Br, 4-Cl, 4-F) and electron-withdrawing (4-CF_3_) substituents were well tolerated in this reaction and furnished the products in 76–90% yield.

Furthermore, we were pleased to find that the diversity of the compounds accessible could be extended by increasing the reaction temperature by 40 °C. Thus, when the reaction between **1a** and **2A** was conducted at 100 °C the N–O bond in the oxadiazole was cleaved and the ring-opening product **3AA** was isolated in 88% yield. A succinct study of the cyclization/ring opening reaction was performed with representative nitroarenes bearing electron-donating (4-OMe, **3CA** and 4-NMe_2_, **3TA**) and electronegative (4-F, **3LA**) groups. These substrates performed well in this cyclization/ring-opening transformation, giving the products in 69–86% yield. These examples highlight the utility of nitroarenes as amino sources in this chemistry.

**Fig. 6:**
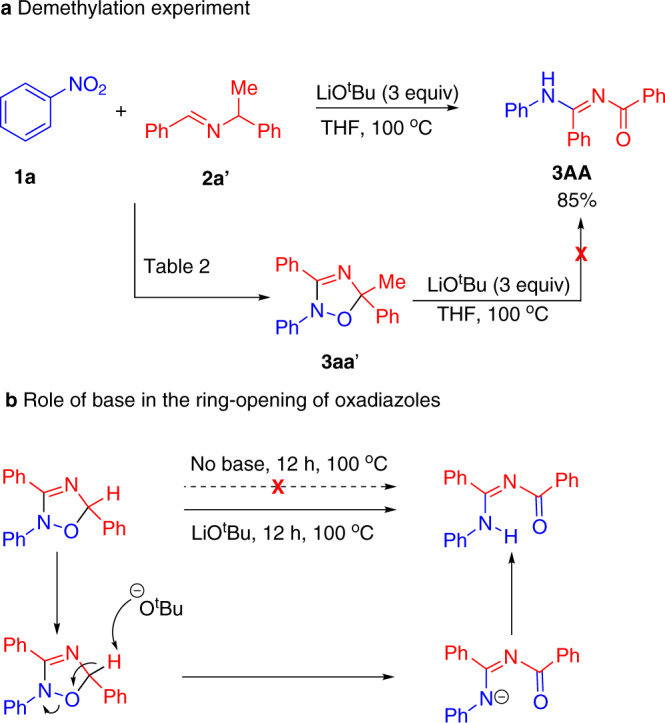
Ring-opening experiments. **a** Demethylation experiment. **b** Role of base in ring-opening of oxadiazoles.

Interestingly, reaction of **2a’** with nitrobenzene in the presence of LiO^*t*^Bu (3 equiv.) at 100 °C resulted in the formation of the ring-opened product **3AA**, which has undergone demethylation. Initially, we suspected that **3aa’** from Fig. 4 is formed as an intermediate and underwent demethylation by LiO^*t*^Bu. However, subjecting **3aa’** to the reaction conditions gave no **3AA** (Fig. 6a). Thus, demethylation occurs earlier in the reaction. Further, we also conducted the reaction without the addition of PhNO_2_, but the demethylation product was not observed in the recovered materials, which indicated that the demethylation did not occur before the participation of the nitroarene.

We were interested in gaining insight into the nature of the ring-opening reactions in Fig. 5. Thus, subjecting the oxadiazole **3aA** to heating at 100 °C in THF for 12 h resulted in recovery of the oxadiazole. When the same reaction was performed in the presence of 2 equiv. LiO-*t*-Bu, which is necessary for the formation of the oxadiazole, the ring-opened product was obtained in 80% yield. This result suggests that the ring-opening is promoted by base, as shown in Fig. 6b. It is noteworthy that the *N*-benzoylbenzamidinate products formed in this tandem reaction are useful ligands in the area of materials science, especially for ring opening polymerization processes^58–60^.

### Mechanistic experiments

**Fig. 7:**
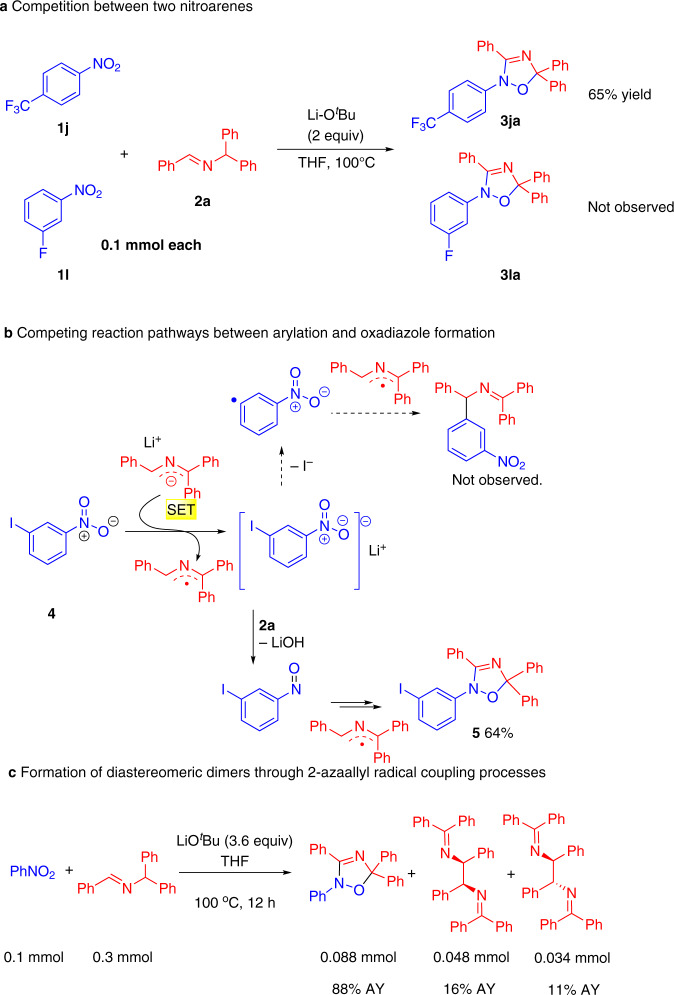
Competition experiments and dimerization of the 2-azaallyl radical. **a** Competition between two nitroarenes. **b** Competing reaction pathways between arylation and oxadiazole formation. **c** Formation of the oxadiazole and diastereomeric dimers through 2-azaallyl radical coupling processes.

To probe the mechanism of this unusual transformation, several experiments were conducted. When the nitroarene was subjected to 1 equiv. LiO^*t*^Bu for 12 h at 100 °C in THF in the absence of imine, the nitroarene was recovered in 87% yield. This result indicates that the LiO^*t*^Bu does not act as the reducing agent to reduce the nitroarene. A competition experiment between 0.1 mmol of 3-fluoro nitrobenzene (**1l**) and 0.1 mmol 4-trifluoromethyl nitrobenzene (**1j**) with 0.1 mmol imine **2a** in the presence of 2 equiv. LiO^*t*^Bu resulted in the formation of product derived from the 4-trifluoromethyl nitrobenzene in 65% yield and no detectable amount of product formed from the 3-fluoro nitrobenzene (Fig. 7a). The faster reaction of the 4-trifluoromethyl nitrobenzene is consistent with a mechanism involving initial reduction of the nitroarene.

Next, we focused on the chemoselectivity between two reactive functional groups. 3-Iodo nitrobenzene **4** has two reactive functional groups that are susceptible to reduction, providing two possible reaction pathways (Fig. 7b). A reasonable first step in both reactions is SET from the 2-azaallyl anion to 3-iodo nitrobenzene to generate the arene radical anion. The top pathway in Fig. 7b involves loss of iodide from the arene radical anion and generation of an aryl radical. Reaction of the persistent 2-azaallyl radical with the aryl radical would give the tetraaryl product, as was previously reported for aryl iodides not bearing nitro groups (Fig. 3a)^61^. However, the arylated product was not detected by ^1^H NMR spectroscopy under these conditions. Instead, it appears that the reduced nitro arene leads to the oxadiazole product **5** in 64% yield (Fig. 7b, bottom pathway). This reaction is proposed to proceed through nitrosobenzene, as discussed further below. The results of Fig. 7b highlight the lower barrier to reaction at the nitro center vs. loss of iodide.

Finally, we note that the dimerization of the 2-azaallyl radical takes place to generate the diimine products, as is commonly observed in reactions of 2-azaallyl anions that proceed through open-shell intermediates (Fig. 7c). Note that the assay yields (AY) were determined by ^1^H NMR integration of the crude reaction mixture. The AY of the diimines are based on the 0.3 mmol aldimine employed in this reaction, whereas the oxadiazole AY is based on the nitrobenzene.

### Mechanistic studies by DFT

**Fig. 8:**
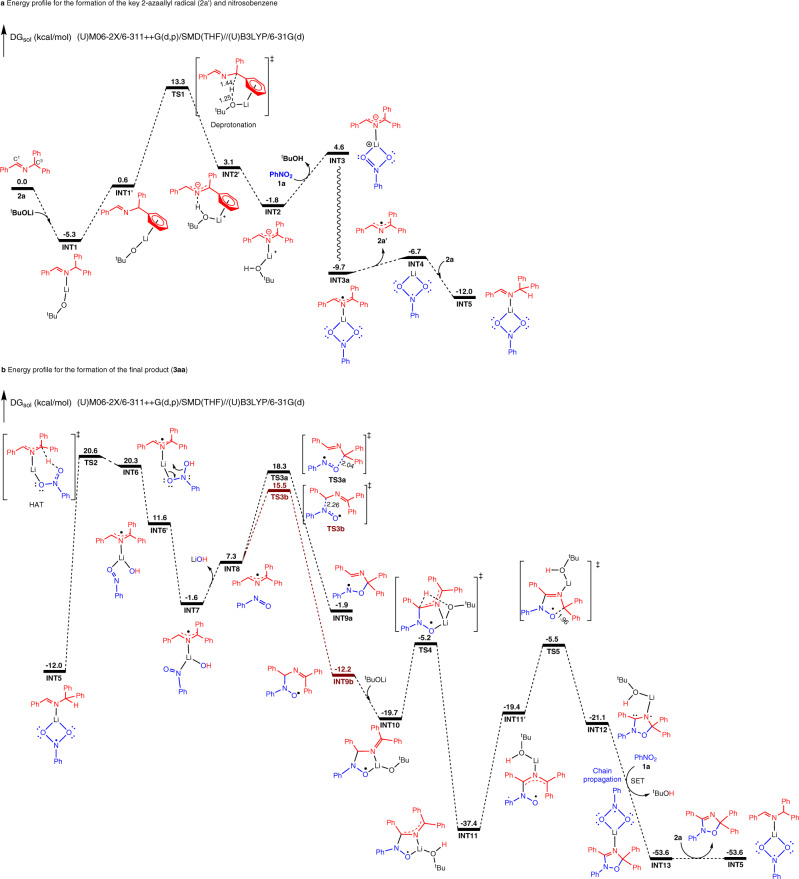
Computational studies. **a** Energy profile for the formation of the key 2-azaallyl radical (**2a′**) and nitrosobenzene. **b** Energy profile for the formation of the final product (**3aa**). Bond distances are shown in Å.

Computational studies were carried out to shed light on the mechanistic pathway. First, the deprotonation of aldimine **2a** in the presence of LiO^*t*^Bu was considered. Two possible adducts between LiO^*t*^Bu and the aldimine were examined (**INT1** and **INT1ʹ**, Fig. 8a). In **INT1** the ketimine **2a** binds to LiO^*t*^Bu via Li–N dative interaction and in **INT1ʹ** a cation–π interaction is formed. Computational results show that the cation–π interaction in **INT1ʹ** is ca. 6 kcal/mol higher in energy than the dative adduct in **INT1**. A transition state for deprotonation via the cation–π adduct was located as shown in **TS1**, in which the C^3^•••H distance is lengthened to 1.44 Å while O•••H distance is shortened to 1.25 Å. The resulting cation–π complex formed after deprotonation (**INT2ʹ**) is reminiscent of crystallographically characterized structures of 2-azaallyl anions with main group metals^50^. This structure is predicted to rearrange to the more stable adduct **INT2** with a Li–N dative interaction.

Next, a ligand exchange step between ^*t*^BuOH and nitrobenzene (**1a**) generates the O-bound κ^2^-nitrobenzene adduct **INT3**. While this step is uphill by 6.4 kcal/mol, **INT3** can undergo an exergonic intersphere SET to afford a diradical complex **INT3a** in triplet state. The open-shell singlet of **INT3a** was also considered, which was 0.2 kcal/mol higher in energy than the triplet state of **INT3a**. The SET from the 2-azaallyl anion to the nitrobenzene is downhill by 14.3 kcal/mol. Intermediate **INT3a** possesses the bound 2-azaallyl radical (**2aʹ**) and the nitrobenzene radical anion. In addition, substituent effects on the nitroarene were also considered for the SET step. When nitroarene **1j**, possessing a 4-CF_3_ substituent, and **1l**, bearing a 3-F substituent, were used in the calculations, the calculated energy gap for **1j** (21.6 kcal/mol) was significantly larger than that of **1l** (18.0 kcal/mol), implying that the generation of the corresponding radicals is more efficient for **1j** than for **1l**. This computational prediction is consistent with the experimental observations that the 4-trifluoromethyl nitrobenzene reacts faster than 3-fluoro nitrobenzene in the competitive experiment shown in Fig. 7a).

We initially expected that diradical intermediate **INT3a** might undergo internal C–N or C–O bond formation (Supplementary Fig. 1). Calculated barriers for these processes, however, exceeded 50 kcal/mol. Thus, we envisioned exchange of the bound 2-azaallyl radical for the aldimine starting material. The liberated 2-azaallyl radical is known to undergo dimerization to afford diimines, which are observed products (Fig. 7c). The calculations indicate that the lithium salt of the nitrobenzene radical anion, **INT4**, can bind to the aldimine **2a** through the nitrogen atom to generate a new adduct, **INT5**. With the nitrobenzene radical anion and the aldimine **2a** both bound to Li^+^, HAT from the benzylic C–H of the bound aldimine by the oxygen of the nitrobenzene radical anion via **TS2** generates **INT6**. Hydroxide migrates from the nitrogen of **INT6** to lithium to form a complex of the 2-azaallyl radical, nitrosobenzene, and LiOH (**INT7**) in the coordination sphere of the Li^+^ (Attempts to locate the transition state of hydroxyl migration failed. The favorable electrostatic attractive interaction between Li cation and hydroxyl group is mainly responsible for the facile formation of LiOH from **INT6** to **INT6’**.). Thus, the reduction of PhNO_2_ to PhNO is proposed, along with the formation of LiOH. Dissociation of the nitrosobenzene and 2-azaallyl radical is next proposed.

After the formation of nitrosobenzene, radical additions with the 2-azaallyl radical **2aʹ** were examined computationally. To form the product **3aa**, both O-attack of PhNO on C^3^ of **2aʹ** and N-attack to C^1^ were considered (Fig. 8b). Computational results show that it is more favorable for N-attack at C^1^ via **TS3b** to afford **INT9b**. One may propose that the subsequent intramolecular O-attack of **INT9b** at C^3^ via **TS4b** might occur to furnish a cyclized intermediate **INT10**. However, computational results show that this cyclization step needs to overcome a much higher activation barrier (41.7 kcal/mol), suggesting that this pathway is not feasible (Supplementary Fig. 2). On the other hand, the formed **INT9b** might undergo a proton transfer step with LiO^*t*^Bu via **TS4** to afford the radical anion **INT11**. Subsequently, the intramolecular cyclization of **INT11** could follow via **TS5** to afford radical anion **INT12**. The calculated activation energy for this step is 31.9 kcal/mol, which is reasonable under these reaction conditions. We also examined the pathway with O-attack of PhNO at C^3^ of the 2-azaallyl radical **2aʹ** (via **TS3a)** as the first step in the cyclization, followed by N-attack to C^1^ via **TS4a** to generate the cyclized species. However, the predicted overall activation energy for this pathway is 37.6 kcal/mol (Supplementary Fig. 2), which is substantially higher than the proposed pathway shown in Fig. 8b.

Finally, the formed radical anion **INT12** could undergo SET with nitrobenzene to form the oxadiazole and **INT13**. The formed **INT13** could undergo another ligand exchange step with substrate **2a** to generate the final product **3aa** and **INT5**, which enters a next cycle.

### Mechanistic considerations

**Fig. 9:**
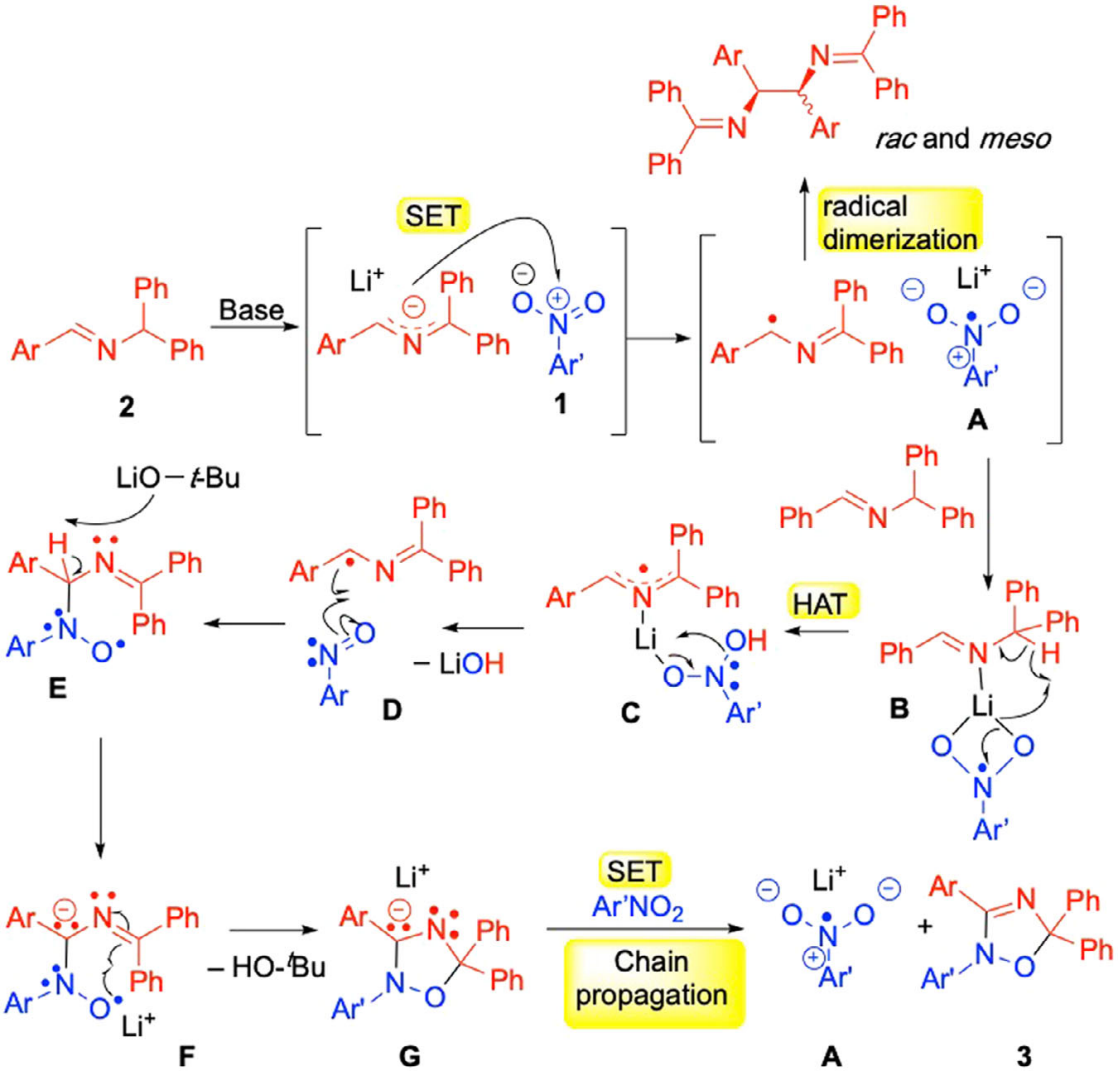
Proposed reaction pathway. Key steps include reduction of the nitroarene by the 2-azaallyl anion, HAT from the aldimine to the nitroarene radical anion (**B**), formation of the nitrosoarene (**D**), and radical-chain propagation from **G**.

Grounded in the computational and experimental results above, and observations reported in the literature, a reaction mechanism is provided in Fig. 9 and discussed here. Deprotonation of the aldimine **2** generates the 2-azaallyl anion. Given that reactions with stronger bases, such as LDA, do not give the oxadiazole products in appreciable yields, we interpret these observations to suggest that low concentrations of the 2-azaallyl anion, or downstream intermediates like the 2-azaallyl radical, are crucial to the success of this reaction. The strong base LDA would rapidly convert the aldimine to the 2-azaallyl anion, depleting the aldimine, which is a key intermediate as seen in Fig. 8b. As noted above, SET from the 2-azaallyl anion to the nitroarene is proposed to afford the nitroarene radical anion, **A**. It is noteworthy that the radical anion of nitrobenzene has been characterized experimentally and computationally^62,63^. As might be expected, most of the increased electron density on forming the nitrobenzene radical anion is located on the nitro group^63^. From electrochemical data it is known that the reduction of nitroarenes occurs more readily than the reduction of aryl iodides^64^. Nonetheless, as shown in Fig. 3a, in the absence of a nitro group, 2-azaallyl anions reduce aryl iodides to aryl radicals^51–54^. Also formed in this SET step is the persistent 2-azaallyl radical, which undergoes dimerization to form the *rac*- and *meso*-diimines outlined in Fig. 7c.

**Fig. 10:**
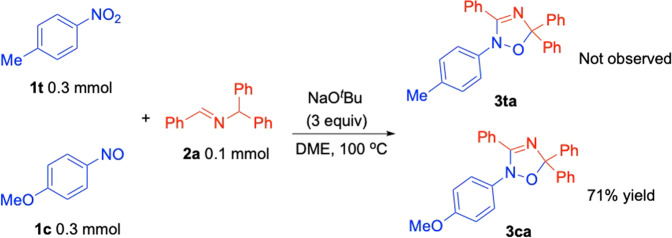
Competition reaction with nitrosoarene. Product **3ca** is consistent with the intermediacy of the nitrosoarene in the net [3+2] reaction. Similar results were obtained with 4-nitro anisole and 4-nitrosotoluene, which gave **3ta** (not shown).

Based on the computational study, the nitroarene radical anion **A** does not react with the 2-azaallyl radical. Instead, calculations show a lower barrier for HAT between the lithium bound nitro arene radical anion and the aldimine via the arrow pushing shown in **B**. The next step involves elimination of LiOH via **C**. The products of this step are a nitrosoarenes and the 2-azaallyl radical. We then set out to determine whether the nitrosoarene was a viable intermediate in this reaction, as shown in Fig. 10. In the presence of 4-nitrotoluene and 4-nitroso anisole, imine, and base, the net [3 + 2] product was formed from the nitrosoarene, consistent with the calculations. The 2-azaallyl radical and nitrosoarene undergo addition to form the C–N bond in **E** with a lower barrier than the formation of the C–O bond (Fig. 8b). Intermediate **E** is proposed to undergo deprotonation by LiO^*t*^Bu and isomerization via **F** to afford the radical anion (**G**). Radical chain propagation occurs when **G** transfers an electron to a nitroarene to regenerate the nitroarene radical anion and the oxadiazole product **3**.

## Discussion

We have developed a transition-metal-free reaction of aldimines with nitroarenes. This method provides a green and operationally simple approach to afford a variety of 2,5-dihydro-1,2,4-oxadiazole derivatives in good yields. The scope of the reaction is broad with high functional group compatibility and enables rapid incorporation of heteroaryl groups. 2,5-Dihydro-1,2,4-oxadiazoles belong to a class of biologically important heterocyclic compounds that are employed in the pharmaceutical industry and found in natural products. This new protocol is distinct from previously reported cycloadditions, because it involves nitroarenes rather than nitrosoarenes. Nitroarenes are abundant, commercially available feedstocks, but have proven difficult to employ as amino sources in organic synthesis. A unique aspect of this reaction is the distinct mechanism, where the 2-azaallyl anion serves to activate the nitroarene by SET, eliminating the need for external reducing agents. Computational studies suggest that the lowest energy pathway involves a radical-chain process. Considering the great potential of nitroarenes in the chemical sciences, we envision that this new protocol will be of interest in modern chemistry and hope that it inspires chemists to revisit the use of nitroarenes as amino sources.

## Methods

### General procedures for synthesis of 3aa

An oven-dried 10 mL vial equipped with a stir bar was charged with aldimine (0.3 mmol) and LiO^*t*^Bu (28.8 mg, 0.36 mmol) under a nitrogen atmosphere in a glovebox. THF (1 mL) was added to the reaction followed by addition of nitroarene (10.2 μL, 0.1 mmol) by syringe at room temperature. The color of the reaction mixture turned to light yellow. The vial was capped, removed from the glovebox, and stirred for 12 h at 100 °C. After cooling to room temperature, the reaction mixture was quenched with three drops of H_2_O, and the vial was open to air, passed through a short pad of silica gel, and eluted with ethyl acetate (1 mL × 3). The combined organic solution was concentrated under reduced pressure. The crude material was loaded onto a silica gel column and purified by flash chromatography.

## Supplementary information

Supplementary Information

## Data Availability

The authors declare that all the data supporting the findings of this study are available within the paper and its Supplementary Information files or from the corresponding author upon request. For the experimental procedures and spectroscopic and physical data of compounds, see Supplementary Methods. For ^1^H and ^13^C{^1^H} NMR spectra of compounds, see Supplementary Figs. 3–113. The X-ray crystallographic coordinates for structures reported in this study have been deposited at the Cambridge Crystallographic Data Centre (CCDC), under deposition numbers CCDC 2032323 (**3aa**) and 2068961 (**3aE**). These data can be obtained free of charge from The Cambridge Crystallographic Data Centre via https://www.ccdc.cam.ac.uk/data_request/cif.
