# Supplementary material for: SET activation of nitroarenes by 2-azaallyl anions as a straightforward access to 2,5-dihydro-1,2,4-oxadiazoles
Source: Nat Commun. 2021 Dec 3;12:7060. doi: 10.1038/s41467-021-26767-x (PMC8642414; doi:10.1038/s41467-021-26767-x)

Supplementary Information

SET Activation of Nitroarenes by 2-Azaallyl Anions as a Straightforward Access to 2,5-Dihydro-1,2,4-oxadiazoles

Dong Zou,[1,4] Lishe Gan,[3,4] Fan Yang,[1] Huan Wang,[1] Youge Pu,[2] Jie Li,*[1] & Patrick J. Walsh*[2]

[1]Department of Pharmacy, School of Medicine, Zhejiang University City College, No. 48, Huzhou Road, Hangzhou 310015, P. R. China

E-mail: lijie@zucc.edu.cn

[2] Roy and Diana Vagelos Laboratories, Penn/Merck Laboratory for High-Throughput Experimentation, University of Pennsylvania, Department of Chemistry, 231 South 34th Street, Philadelphia, Pennsylvania 19104, USA.

E-mail: pwalsh@sas.upenn.edu

[3]School of Biotechnology and Health Sciences, Wuyi University, Jiangmen 529020, P. R. China

[4]These authors contributed equally

Table of Contents

General Information S2

Preparation of *N*-benzylidene-1,1-diphenylmethanamines S2

Preparation of *N*-benzylidene-1-phenylmethanimines S2

Synthesis of 2,5-dihydro-1,2,4-oxadiazoles S2

Computational details S23

Supplementary References S67

NMR Spectra S68

The X-ray structures of 3aa and 3aE S123

General Information

All reactions were conducted under an atmosphere of dry nitrogen with oven-dried glassware or vacuum line techniques. All anhydrous solvents were purchased from Sigma-Aldrich and directly used without further purification. Unless otherwise stated, reagents were commercially available and used as purchased without further purification. Chemicals were purchased from Sigma-Aldrich, TCI China, Acros, Alfa Aesar or J&K.

Progress of reactions was monitored by thin-layer chromatography using TLC plates and visualized by short-wave ultraviolet light. Flash chromatography was performed with Qingdao Haiyang flash silica gel (200–300 mesh). The NMR spectra were obtained using a Bruker AVANCE III 500 MHz spectrometer with TMS as the internal standard. The infrared spectra were obtained with KBr plates by using an FTIR650 FT-IR Spectrometer. High resolution mass spectrometry (HRMS) data were obtained on an Agilent Q-TOF 1290 LC/6224 MS system using electrospray ionization (ESI) in positive or negative mode. Melting points were determined on a Thermal Values analytical microscope and were uncorrected.

Preparation of *N*-benzylidene-1,1-diphenylmethanamines: *N*-benzylidene-1,1-diphenylmethanamines were prepared according to literature procedures.[1]

Preparation of *N*-benzylidene-1-phenylmethanimines: *N*-benzylidene-1-phenylmethanimines were prepared according to literature procedures.[2]

Synthesis of 2,5-dihydro-1,2,4-oxadiazoles

General Procedure

An oven-dried 10 mL vial equipped with a stir bar was charged with aldimine (0.3 mmol) and LiO^{t}Bu (28.8 mg, 0.36 mmol) under a nitrogen atmosphere in a glovebox. THF (1 mL) was added to the reaction followed by addition of nitroarene (10.2 μL, 0.1 mmol) by syringe at room temperature. The color of the reaction mixture turned to light yellow. The vial was capped, removed from the glovebox, and stirred for 12 h at 100 °C. After cooling to room temperature, the reaction mixture was quenched with three drops of H_2O and the vial was open to the air, passed through a short pad of silica gel and eluted with ethyl acetate (1 mL × 3). The combined organic solution was concentrated under reduced pressure. The crude material was loaded onto a silica gel column and purified by flash chromatography.

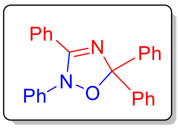

2,3,5,5-Tetraphenyl-2,5-dihydro-1,2,4-oxadiazole (3aa). The reaction was performed following the General Procedure with nitrobenzene **1a** (10.2 µL, 0.1 mmol), LiO^{t}Bu (28.8 mg, 0.36 mmol), and **2a** (81.3 mg, 0.3 mmol) dissolved in THF (1 mL) at 100 °C for 12 h. The crude material was purified by flash chromatography on silica gel (eluted with hexanes:EtOAc = 200:1) to give the product (32.3 mg, 86% yield) as a white solid. mp = 92−94 °C. ^{1}H NMR (500 MHz, $CDCl_3$): δ 7.78 – 7.76 (m, 2H), 7.59 – 7.57 (m, 4H), 7.39 – 7.27 (m, 9H), 7.25 – 7.19 (m, 5H); $^{13}C\{^1H\}$ NMR (125 MHz, $CDCl_3$): δ 160.3, 143.9, 131.3, 129.24, 129.21, 128.5, 128.33, 128.26, 128.1, 128.0, 127.0, 126.7, 109.8, one resonance was not observed due to coincidental overlap; IR (thin film): 3057, 1631, 1595, 1487, 1448, 1325, 1261 cm^{-1}; HRMS (ESI) m/z: $[M + H]^+$ calcd for $C_{26}H_{21}N_2O$ 377.1648; found 377.1644.

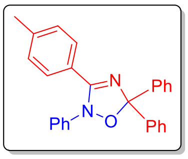

2,5,5-Triphenyl-3-(*p*-tolyl)-2,5-dihydro-1,2,4-oxadiazole (3ab). The reaction was performed following the General Procedure with nitrobenzene **1a** (10.2 µL, 0.1 mmol), LiO^{t}Bu (28.8 mg, 0.36 mmol), and **2b** (85.5 mg, 0.3 mmol) dissolved in THF (1 mL) at 100 °C for 12 h. The crude material was purified by flash chromatography on silica gel (eluted with hexanes:EtOAc = 200:1) to give the product (33.9 mg, 87% yield) as a white solid. mp = 113−115 °C. ^{1}H NMR (500 MHz, $CDCl_3$): δ 7.65 (d, J = 8.2 Hz, 2H), 7.58 – 7.55 (m, 4H), 7.33 – 7.30 (m, 4H), 7.27 – 7.24 (m, 2H), 7.21 – 7.18 (m, 5H), 7.08 (d, J = 8.1 Hz, 2H), 2.29 (s, 3H); $^{13}C\{^1H\}$ NMR (125 MHz, $CDCl_3$): δ 160.3, 144.1, 144.0, 141.7, 129.21, 129.18, 129.16, 128.2, 127.9, 127.0, 126.7, 125.2, 109.8, 21.6, one resonance was not observed due to coincidental overlap; IR (thin film): 3057, 2850, 1630, 1595, 1488, 1331, 1260 cm^{-1}; HRMS (ESI) m/z: $[M + H]^+$ calcd for $C_{27}H_{23}N_2O$ 391.1805; found 391.1812.

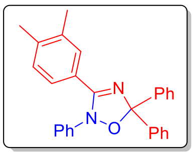

3-(3,4-Dimethylphenyl)-2,5,5-triphenyl-2,5-dihydro-1,2,4-oxadiazole (3ac). The reaction was performed following the General Procedure with nitrobenzene **1a** (10.2 µL, 0.1 mmol), LiO^{t}Bu (28.8 mg, 0.36 mmol), and **2c** (89.7 mg, 0.3 mmol) dissolved in THF (1 mL) at 100 °C for 12 h. The crude material was purified by flash chromatography on silica gel (eluted with hexanes:EtOAc = 200:1) to give the product (36.8 mg, 92% yield) as a white solid. mp = 120−122 °C. ^{1}H NMR (500 MHz, $CDCl_3$): δ 7.65 (d, J = 1.3 Hz, 1H), 7.58 – 7.56 (m, 4H), 7.39 (dd, J = 7.8, 1.7 Hz, 1H), 7.35 – 7.31 (m, 4H), 7.29 – 7.27 (m, 2H), 7.24 – 7.18 (m, 5H), 7.03 (d, J = 7.2 Hz, 1H), 2.22 (s, 6H); $^{13}C\{^1H\}$ NMR (125 MHz, $CDCl_3$): δ 160.5, 144.3, 144.0, 140.4, 137.0, 130.1, 129.7, 129.2, 128.2, 128.1, 127.9, 126.9, 126.8, 126.7, 125.6, 109.7, 20.0, 19.8; IR (thin film):

3056, 2920, 1654, 1628, 1489, 1446, 1328 cm^{-1}; HRMS (ESI) m/z: [M + H]$^+$ calcd for $C_{28}H_{25}N_2O$ 405.1961; found 405.1947.

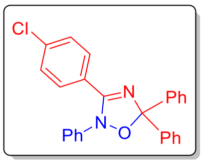

3-(4-Chlorophenyl)-2,5,5-triphenyl-2,5-dihydro-1,2,4-oxadiazole (3ad). The reaction was performed following the General Procedure with nitrobenzene **1a** (10.2 μL, 0.1 mmol), LiO^{t}Bu (28.8 mg, 0.36 mmol), and **2d** (91.5 mg, 0.3 mmol) dissolved in THF (1 mL) at 100 °C for 12 h. The crude material was purified by flash chromatography on silica gel (eluted with hexanes:EtOAc = 200:1) to give the product (33.6 mg, 82% yield) as a white solid. mp = 107−108 °C. ^{1}H NMR (500 MHz, $CDCl_3$): δ 7.71 – 7.68 (m, 2H), 7.57 – 7.54 (m, 4H), 7.34 – 7.31 (m, 4H), 7.29 – 7.21 (m, 7H), 7.19 – 7.17 (m, 2H); $^{13}C\{^1H\}$ NMR (125 MHz, $CDCl_3$): δ 159.4, 143.8, 143.7, 137.5, 130.5, 129.4, 128.8, 128.7, 128.3, 128.1, 127.2, 126.6, 126.5, 110.0; IR (thin film): 3061, 1632, 1594, 1491, 1450, 1325, 1206 cm^{-1}; HRMS (ESI) m/z: [M + H]$^+$ calcd for $C_{26}H_{20}ClN_2O$ 411.1259; found 411.1255.

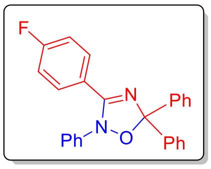

3-(4-Fluorophenyl)-2,5,5-triphenyl-2,5-dihydro-1,2,4-oxadiazole (3ae). The reaction was performed following the General Procedure with nitrobenzene **1a** (10.2 μL, 0.1 mmol), LiO^{t}Bu (28.8 mg, 0.36 mmol), and **2e** (86.7 mg, 0.3 mmol) dissolved in THF (1 mL) at 100 °C for 12 h. The crude material was purified by flash chromatography on silica gel (eluted with hexanes:EtOAc = 200:1) to give the product (29.9 mg, 76% yield) as a white solid. mp = 92−94 °C. ^{1}H NMR (500 MHz, $CDCl_3$): δ 7.80 – 7.76 (m, 2H), 7.58 – 7.56 (m, 4H), 7.36 – 7.32 (m, 4H), 7.31 – 7.27 (m, 2H), 7.26 – 7.23 (m, 3H), 7.21 – 7.17 (m, 2H), 7.01 – 6.97 (m, 2H); $^{13}C\{^1H\}$ NMR (125 MHz, $CDCl_3$): δ 164.5 (d, $J^1_{C\text{-}F}$ = 252.2 Hz), 159.4, 143.8, 143.7, 131.4 (d, $J^3_{C\text{-}F}$ = 8.8 Hz), 129.3, 128.6, 128.3, 128.1, 127.2, 126.6, 124.2 (d, $J^4_{C\text{-}F}$ = 3.2 Hz), 115.7 (d, $J^2_{C\text{-}F}$ = 21.9 Hz), 109.9; IR (thin film): 1633, 1509, 1489, 1449, 1325 cm^{-1}; HRMS (ESI) m/z: [M + H]$^+$ calcd for $C_{26}H_{20}FN_2O$ 395.1554; found 395.1546.

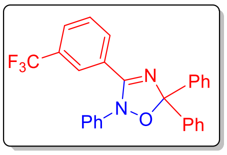

2,5,5-Triphenyl-3-(3-(trifluoromethyl)phenyl)-2,5-dihydro-1,2,4-oxadiazole (3af). The reaction was performed following the General Procedure with nitrobenzene **1a** (10.2 μL, 0.1 mmol), LiO^{t}Bu (28.8 mg, 0.36 mmol), and **2f** (101.7 mg, 0.3 mmol) dissolved in THF (1 mL) at 100 °C for 12 h. The crude material was purified by flash chromatography on silica gel (eluted with hexanes:EtOAc = 200:1) to give the product (35.9 mg, 81% yield) as a colorless oil. ^{1}H NMR (500 MHz, $CDCl_3$): δ 8.03 (s, 1H), 7.77 (d, J = 7.9 Hz, 1H), 7.54 – 7.49 (m, 5H), 7.33 – 7.25 (m, 5H), 7.23 – 7.12 (m, 7H); $^{13}C\{^1H\}$ NMR (125 MHz, $CDCl_3$): δ 159.2,

143.6, 143.4, 132.4, 131.1 (q, $J^2_{C(Ar)-F}$ = 32.9 Hz) 130.2, 129.5, 129.1, 128.9, 128.4, 128.2, 127.9 (q, $J^3_{C(Ar)-F}$ = 3.6 Hz), 127.3, 126.6, 126.1 (q, $J^3_{C(Ar)-F}$ = 3.9 Hz), 123.7 (q, J^1_{C-F} = 272.5 Hz), 110.1; IR (thin film): 1637, 1523, 1486, 1428, 1325 cm^{-1}; HRMS (ESI) m/z: $[M + H]^+$ calcd for $C_{27}H_{20}F_3N_2O$ 445.1522; found 445.1533.

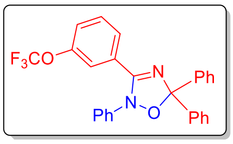

2,5,5-Triphenyl-3-(3-(trifluoromethoxy)phenyl)-2,5-dihydro-1,2,4-oxadiazole (3ag). The reaction was performed following the General Procedure with nitrobenzene **1a** (10.2 μL, 0.1 mmol), LiO^{t}Bu (28.8 mg, 0.36 mmol), and **2g** (106.5 mg, 0.3 mmol) dissolved in THF (1 mL) at 100 °C for 12 h. The crude material was purified by flash chromatography on silica gel (eluted with hexanes:EtOAc = 200:1) to give the product (39.1 mg, 85% yield) as a colorless oil. ^{1}H NMR (500 MHz, $CDCl_3$): δ 7.59 (d, J = 7.8 Hz, 1H), 7.54 (s, 1H), 7.49 (d, J = 7.3 Hz, 4H), 7.27 – 7.23 (m, 4H), 7.22 – 7.11 (m, 9H); $^{13}C\{^1H\}$ NMR (125 MHz, $CDCl_3$): δ 159.0, 149.04, 143.6, 143.4, 130.0, 129.9, 129.4, 128.8, 128.3, 128.1, 127.5, 127.2, 126.5, 123.6, 121.7, 120.4 (q, J_{C-F} = 258.1 Hz), 110.0; IR (thin film): 1634, 1598, 1483, 1450, 1259, 1217 cm^{-1}; HRMS (ESI) m/z: $[M + H]^+$ calcd for $C_{27}H_{20}F_3N_2O_2$ 461.1471; found 461.1488

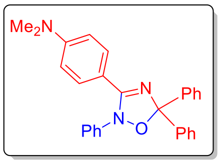

***N,N*-Dimethyl-4-(2,5,5-triphenyl-2,5-dihydro-1,2,4-oxadiazol-3-yl)aniline (3ah).** The reaction was performed following the General Procedure with nitrobenzene **1a** (10.2 μL, 0.1 mmol), LiO^{t}Bu (28.8 mg, 0.36 mmol), and **2h** (94.2 mg, 0.3 mmol) dissolved in THF (1 mL) at 100 °C for 12 h. The crude material was purified by flash chromatography on silica gel (eluted with hexanes:EtOAc = 100:1) to give the product (39.8 mg, 95% yield) as a yellow solid. mp = 151−153 °C. ^{1}H NMR (500 MHz, $CDCl_3$): δ 7.66 – 7.64 (m, 2H), 7.56 – 7.54 (m, 4H), 7.32 – 7.28 (m, 4H), 7.25 (m, 2H), 7.23 – 7.18 (m, 5H), 6.56 – 6.53 (m, 2H), 2.95 (s, 6H); $^{13}C\{^1H\}$ NMR (125 MHz, CDC13): δ 160.3, 152.1, 145.0, 144.4, 130.7, 129.1, 128.1, 127.9, 127.8, 127.0, 126.8, 114.9, 111.2, 109.7, 40.2; IR (thin film): 2921, 1613, 1529, 1487, 1448, 1396, 1330, 1193 cm^{-1}; HRMS (ESI) m/z: $[M + H]^+$ calcd for $C_{28}H_{26}N_3O$ 420.2070; found 420.2060.

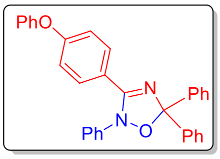

3-(4-Phenoxyphenyl)-2,5,5-triphenyl-2,5-dihydro-1,2,4-oxadiazole (3ai). The reaction was performed following the General Procedure with nitrobenzene **1a** (10.2 μL, 0.1 mmol), LiO^{t}Bu (28.8 mg, 0.36 mmol), and **2i** (108.9 mg, 0.3 mmol) dissolved in THF (1 mL) at 100 °C for 12 h. The crude material was purified by flash chromatography on silica gel (eluted with hexanes:EtOAc = 200:1) to give the product (42.1 mg, 90% yield) as a white solid. mp = 129−131 °C. ^{1}H NMR (500 MHz, $CDCl_3$): δ 7.66 – 7.64 (m, 2H), 7.50 – 7.47 (m,

4H), 7.27 – 7.23 (m, 6H), 7.21 – 7.17 (m, 3H), 7.15 – 7.11 (m, 4H), 7.07 – 7.04 (m, 1H), 6.92 – 6.89 (m, 2H), 6.80 – 6.78 (m, 2H); $^{13}C\{^1H\}$ NMR (125 MHz, $CDCl_3$): δ 160.3, 159.7, 155.9, 144.1, 144.0, 131.1, 130.1, 129.3, 128.4, 128.2, 128.0, 127.2, 126.7, 124.4, 122.3, 120.0, 117.7, 109.8; IR (thin film): 3060, 1630, 1587, 1488, 1448, 1243 cm^{-1}; HRMS (ESI) m/z: $[M + Na]^+$ calcd for $C_{32}H_{24}N_2NaO_2$ 491.1730; found 491.1736.

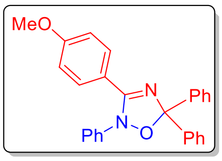

3-(4-Methoxyphenyl)-2,5,5-triphenyl-2,5-dihydro-1,2,4-oxadiazole (3aj). The reaction was performed following the General Procedure with nitrobenzene **1a** (10.2 µL, 0.1 mmol), LiO^{t}Bu (28.8 mg, 0.36 mmol), and **2j** (90.3 mg, 0.3 mmol) dissolved in THF (1 mL) at 100 °C for 12 h. The crude material was purified by flash chromatography on silica gel (eluted with hexanes:EtOAc = 200:1) to give the product (29.2 mg, 72% yield) as a colorless oil. ^1H NMR (500 MHz, $CDCl_3$): δ 7.73 – 7.70 (m, 2H), 7.57 – 7.55 (m, 4H), 7.34 – 7.30 (m, 4H), 7.28 – 7.27 (m, 2H), 7.23 – 7.18 (m, 5H), 6.81 – 6.79 (m, 2H), 3.77 (s, 3H); $^{13}C\{^1H\}$ NMR (125 MHz, $CDCl_3$): δ 161.9, 159.9, 144.3, 144.1, 131.0, 129.2, 128.2, 127.9, 127.1, 126.7, 120.4, 113.9, 109.8, 55.4, one resonance was not observed due to coincidental overlap; IR (thin film): 3056, 2922, 1628, 1597, 1489, 1403, 1100 cm^{-1}; HRMS (ESI) m/z: $[M + Na]^+$ calcd for $C_{27}H_{22}N_2NaO_2$ 429.1573; found 429.1589.

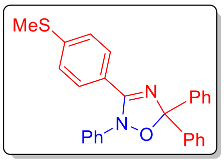

3-(4-(Methylthio)phenyl)-2,5,5-triphenyl-2,5-dihydro-1,2,4-oxadiazole (3ak). The reaction was performed following the General Procedure with nitrobenzene **1a** (10.2 µL, 0.1 mmol), LiO^{t}Bu (19.2 mg, 0.24 mmol), and **2k** (63.4 mg, 0.2 mmol) dissolved in THF (1 mL) at 100 °C for 12 h. The crude material was purified by flash chromatography on silica gel (eluted with hexanes:EtOAc = 200:1) to give the product (25.7 mg, 61% yield) as a colorless oil. ^1H NMR (500 MHz, $CDCl_3$): δ 7.69 – 7.66 (m, 2H), 7.58 – 7.55 (m, 4H), 7.35 – 7.31 (m, 4H), 7.30 – 7.27 (m, 3H), 7.25 – 7.22 (m, 2H), 7.21 – 7.18 (m, 2H), 7.14 – 7.11 (m, 2H), 2.44 (s, 3H); $^{13}C\{^1H\}$ NMR (125 MHz, $CDCl_3$): δ 159.9, 144.1, 143.9, 143.2, 129.5, 129.3, 128.4, 128.2, 128.0, 127.1, 126.7, 125.3, 124.20, 109.9, 15.0; IR (thin film): 2938, 2850, 1610, 1596, 1488, 1398 cm^{-1}; HRMS (ESI) m/z: $[M + H]^+$ calcd for $C_{27}H_{23}N_2OS$ 423.1526; found 423.1536.

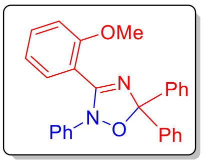

3-(2-Methoxyphenyl)-2,5,5-triphenyl-2,5-dihydro-1,2,4-oxadiazole (3al). The reaction was performed following the General Procedure with nitrobenzene **1a** (10.2 µL, 0.1 mmol), LiO^{t}Bu (28.8 mg, 0.36 mmol), and **2l** (90.3 mg, 0.3 mmol) dissolved in THF (1 mL) at 100 °C for 12 h. The crude material was purified by flash chromatography

on silica gel (eluted with hexanes:EtOAc = 200:1) to give the product (28.4 mg, 70% yield) as a colorless oil. ^{1}H NMR (500 MHz, $CDCl_3$): δ 7.67 – 7.65 (m, 4H), 7.45 (dd, J = 7.6, 1.7 Hz, 1H), 7.39 – 7.36 (m, 4H), 7.35 – 7.30 (m, 3H), 7.17 – 7.13 (m, 2H), 7.09 – 7.06 (m, 3H), 6.94 – 6.90 (m, 1H), 6.80 (d, J = 8.2 Hz, 1H), 3.58 (s, 3H); ^{13}C{^{1}H} NMR (125 MHz, $CDCl_3$): δ 157.8, 157.6, 143.7, 142.4, 132.1, 131.1, 128.6, 128.2, 128.0, 126.9, 126.4, 122.8, 120.6, 117.8, 111.6, 108.8, 55.6; IR (thin film): 2961, 1630, 1565, 1489, 1448, 1094 cm^{-1}; HRMS (ESI) m/z: [M + H]$^{+}$ calcd for $C_{27}H_{23}N_2O_2$ 407.1754; found 407.1762.

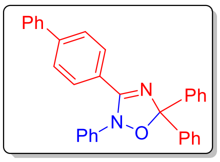

3-([1,1'-Biphenyl]-4-yl)-2,5,5-triphenyl-2,5-dihydro-1,2,4-oxadiazole (3am). The reaction was performed following the General Procedure with nitrobenzene **1a** (10.2 µL, 0.1 mmol), LiO^{t}Bu (28.8 mg, 0.36 mmol), and **2m** (104.1 mg, 0.3 mmol) dissolved in THF (1 mL) at 100 °C for 12 h. The crude material was purified by flash chromatography on silica gel (eluted with hexanes:EtOAc = 200:1) to give the product (34.4 mg, 76% yield) as a white solid. mp = 134−135 °C. ^{1}H NMR (500 MHz, $CDCl_3$): δ 7.85 – 7.83 (m, 2H), 7.60 – 7.58 (m, 4H), 7.55 – 7.52 (m, 4H), 7.44 – 7.40 (m, 2H), 7.37 – 7.32 (m, 5H), 7.31 – 7.28 (m, 2H), 7.26 – 7.23 (m, 5H); ^{13}C{^{1}H} NMR (125 MHz, $CDCl_3$): δ 160.1, 144.02, 143.98, 143.9, 140.1, 129.7, 129.3, 129.0, 128.4, 128.3, 128.1, 128.0, 127.24, 127.16, 126.8, 126.7, 109.9, one resonance was not observed due to coincidental overlap; IR (thin film): 1630, 1560, 1486, 1447, 1407, 1261, 1093, 803 cm^{-1}; HRMS (ESI) m/z: [M + H]$^{+}$ calcd for $C_{32}H_{25}N_2O$ 453.1961; found 453.1940.

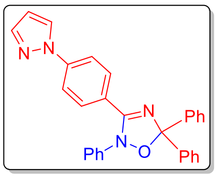

3-(4-(1*H*-Pyrazol-1-yl)phenyl)-2,5,5-triphenyl-2,5-dihydro-1,2,4-oxadiazole (3an). The reaction was performed following the General Procedure with nitrobenzene **1a** (10.2 µL, 0.1 mmol), LiO^{t}Bu (28.8 mg, 0.36 mmol), and **2n** (101.1 mg, 0.3 mmol) dissolved in THF (1 mL) at 100 °C for 12 h. The crude material was purified by flash chromatography on silica gel (eluted with hexanes:EtOAc = 100:1) to give the product (34.5 mg, 78% yield) as a colorless oil. ^{1}H NMR (500 MHz, $CDCl_3$): δ 7.81 – 7.76 (m, 3H), 7.63 (dd, J = 10.2, 1.7 Hz, 1H), 7.57 – 7.54 (m, 2H), 7.51 – 7.49 (m, 4H), 7.27 – 7.24 (m, 4H), 7.22 – 7.19 (m, 2H), 7.17 – 7.13 (m, 5H), 6.36 (dd, J = 2.5, 1.8 Hz, 1H); ^{13}C{^{1}H} NMR (125 MHz, $CDCl_3$): δ 159.5, 143.82, 143.80, 142.0, 141.8, 130.6, 129.3, 128.6, 128.3, 128.0, 127.2, 126.8, 126.6, 125.8, 118.6, 110.0, 108.4; IR (thin film): 1617, 1585, 1447, 1338, 1280, 1191 cm^{-1}; HRMS (ESI) m/z: [M + Na]$^{+}$ calcd for $C_{29}H_{22}N_4NaO$ 465.1686; found 465.1701.

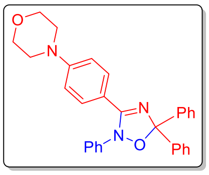

4-(4-(2,5,5-Triphenyl-2,5-dihydro-1,2,4-oxadiazol-3-yl)phenyl)morpholine (3ao). The reaction was performed following the General Procedure with nitrobenzene **1a** (10.2 μL, 0.1 mmol), LiO^{t}Bu (28.8 mg, 0.36 mmol), and **2o** (106.8 mg, 0.3 mmol) dissolved in THF (1 mL) at 100 °C for 12 h. The crude material was purified by flash chromatography on silica gel (eluted with hexanes:EtOAc = 100:1) to give the product (34.5 mg, 75% yield) as a colorless oil. ^{1}H NMR (500 MHz, $CDCl_3$): δ 7.69 – 7.66 (m, 2H), 7.56 – 7.53 (m, 4H), 7.32 – 7.29 (m, 4H), 7.27 – 7.26 (m, 1H), 7.25 – 7.17 (m, 6H), 6.76 – 6.73 (m, 2H), 3.81 – 3.79 (m, 4H), 3.18 – 3.16 (m, 4H); ^{13}C{^{1}H} NMR (125 MHz, $CDCl_3$): δ 160.0, 153.0, 144.6, 144.2, 130.6, 129.2, 128.2, 128.1, 127.9, 127.1, 126.7, 118.3, 114.1, 109.8, 66.8, 48.0; IR (thin film): 3058, 2962, 2853, 1607, 1518, 1448, 1237, 1123 cm^{-1}; HRMS (ESI) m/z: [M + Na]$^+$ calcd for $C_{30}H_{27}N_3NaO_2$ 484.1995; found 484.2005.

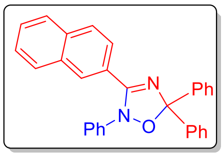

3-(Naphthalen-2-yl)-2,5,5-triphenyl-2,5-dihydro-1,2,4-oxadiazole (3ap). The reaction was performed following the General Procedure with nitrobenzene **1a** (10.2 μL, 0.1 mmol), LiO^{t}Bu (28.8 mg, 0.36 mmol), and **2p** (96.3 mg, 0.3 mmol) dissolved in THF (1 mL) at 100 °C for 12 h. The crude material was purified by flash chromatography on silica gel (eluted with hexanes:EtOAc = 200:1) to give the product (34.5 mg, 81% yield) as a white solid. mp = 137−138 °C. ^{1}H NMR (500 MHz, $CDCl_3$): δ 8.27 (d, J = 1.2 Hz, 1H), 7.86 (dd, J = 8.6, 1.7 Hz, 1H), 7.80 – 7.76 (m, 3H), 7.63 – 7.60 (m, 4H), 7.52 – 7.45 (m, 2H), 7.37 – 7.33 (m, 4H), 7.31 – 7.28 (m, 2H), 7.25 – 7.18 (m, 5H); ^{13}C{^{1}H} NMR (125 MHz, $CDCl_3$): δ 160.4, 144.1, 143.9, 134.6, 132.7, 129.9, 129.3, 128.9, 128.6, 128.32, 128.28, 128.0, 127.9, 127.7, 127.0, 126.7, 125.54, 125.47, 110.0, one resonance was not observed due to coincidental overlap; IR (thin film): 1630, 1595, 1488, 1446, 1261, 1070, 808 cm^{-1}; HRMS (ESI) m/z: [M + H]$^+$ calcd for $C_{30}H_{23}N_2O$ 427.1805; found 427.1815.

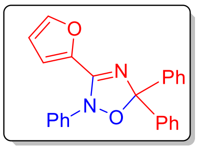

3-(Furan-2-yl)-2,5,5-triphenyl-2,5-dihydro-1,2,4-oxadiazole (3aq). The reaction was performed following the General Procedure with nitrobenzene **1a** (10.2 μL, 0.1 mmol), LiO^{t}Bu (19.2 mg, 0.24 mmol), and **2q** (52.2 mg, 0.2 mmol) dissolved in THF (1 mL) at 100 °C for 12 h. The crude material was purified by flash chromatography on silica gel (eluted with hexanes:EtOAc = 200:1) to give the product (23.0 mg, 63% yield) as a colorless oil. ^{1}H NMR (500 MHz, $CDCl_3$): δ 7.61 – 7.60 (m, 4H), 7.47 (dd, J = 1.7, 0.7 Hz, 1H), 7.37 – 7.33 (m, 9H), 7.31 – 7.27 (m, 2H), 6.37 (dd, J = 3.5, 0.6 Hz, 1H), 6.32 (dd, J = 3.5, 1.8 Hz, 1H); ^{13}C{^{1}H} NMR (125

MHz, $CDCl_3$): δ 152.1, 145.4, 143.7, 143.4, 142.4, 129.42, 129.39, 128.3, 128.1, 127.8, 126.8, 116.0, 111.6, 109.5; IR (thin film): 3115, 1667, 1524, 1487, 1448, 1331, 1120 cm^{-1}; HRMS (ESI) m/z: $[M + H]^+$ calcd for $C_{24}H_{19}N_2O_2$ 367.1441; found 367.1452.

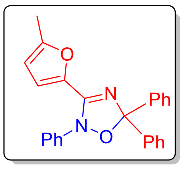

3-(5-Methylfuran-2-yl)-2,5,5-triphenyl-2,5-dihydro-1,2,4-oxadiazole (3ar). The reaction was performed following the General Procedure with nitrobenzene **1a** (10.2 µL, 0.1 mmol), LiO^{t}Bu (19.2 mg, 0.24 mmol), and **2r** (55.0 mg, 0.2 mmol) dissolved in THF (1 mL) at 100 °C. The crude material was purified by flash chromatography on silica gel (eluted with hexanes:EtOAc = 200:1) to give the product (22.0 mg, 58% yield) as a colorless oil. ^1H NMR (500 MHz, $CDCl_3$): δ 7.61 – 7.59 (m, 4H), 7.38 – 7.33 (m, 9H), 7.31 – 7.27 (m, 2H), 6.20 (d, J = 3.4 Hz, 1H), 5.91 (dd, J = 3.4, 0.9 Hz, 1H), 2.31 (s, 3H); $^{13}C\{^1H\}$ NMR (125 MHz, $CDCl_3$): δ 156.2, 152.2, 144.2, 143.4, 140.7, 129.32, 129.25, 128.2, 128.0, 127.8, 126.9, 117.6, 109.4, 108.2, 14.0; IR (thin film): 3112, 2963, 1613, 1585, 1448, 1329 cm^{-1}; HRMS (ESI) m/z: $[M + Na]^+$ calcd for $C_{25}H_{20}N_2NaO_2$ 403.1417; found 403.1425.

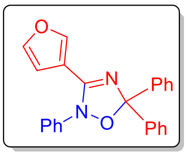

3-(Furan-3-yl)-2,5,5-triphenyl-2,5-dihydro-1,2,4-oxadiazole (3as). The reaction was performed following the General Procedure with nitrobenzene **1a** (10.2 µL, 0.1 mmol), LiO^{t}Bu (28.8 mg, 0.36 mmol), and **2s** (78.3 mg, 0.3 mmol) dissolved in THF (1 mL) at 100 °C for 12 h. The crude material was purified by flash chromatography on silica gel (eluted with hexanes:EtOAc = 200:1) to give the product (24.1 mg, 66% yield) as a colorless oil. ^1H NMR (500 MHz, $CDCl_3$): δ 7.61 – 7.59 (m, 4H), 7.38 – 7.35 (m, 10H), 7.32 – 7.29 (m, 3H), 6.66 – 6.65 (m, 1H); $^{13}C\{^1H\}$ NMR (125 MHz, $CDCl_3$): δ 154.6, 144.9, 143.6, 143.5, 143.3, 129.5, 129.4, 128.3, 128.1, 127.9, 126.7, 115.0, 110.3, 109.2; IR (thin film): 3112, 2963, 1644, 1555, 1489, 1448, 1389, 1237 cm^{-1}; HRMS (ESI) m/z: $[M + Na]^+$ calcd for $C_{24}H_{18}N_2NaO_2$ 389.1260; found 389.1270.

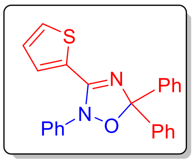

2,5,5-Triphenyl-3-(thiophen-2-yl)-2,5-dihydro-1,2,4-oxadiazole (3at). The reaction was performed following the General Procedure with nitrobenzene **1a** (10.2 µL, 0.1 mmol), LiO^{t}Bu (28.8 mg, 0.36 mmol), and **2t** (86.1 mg, 0.3 mmol) dissolved in THF (1 mL) at 100 °C for 12 h. The crude material was purified by flash chromatography on silica gel (eluted with hexanes:EtOAc = 200:1) to give the product (25.2 mg, 66% yield) as a white solid. mp = 109−111 °C. ^1H NMR (500 MHz, $CDCl_3$): δ 7.60 – 7.58 (m, 4H), 7.38 – 7.32 (m, 10H), 7.31 – 7.28 (m, 2H), 7.14 (dd, J = 3.8, 1.1 Hz, 1H), 6.90 (dd, J = 5.0, 3.8 Hz, 1H); $^{13}C\{^1H\}$ NMR (125 MHz, $CDCl_3$): δ 155.4, 143.9, 143.6, 131.4, 130.2, 130.0, 129.4, 129.3, 128.3, 128.14, 128.05, 127.6,

126.8, 109.4; IR (thin film): 3058, 1618, 1594, 1487, 1448, 1429, 1261, 762 cm^{-1}; HRMS (ESI) m/z: $[M + H]^+$ calcd for $C_{24}H_{19}N_2OS$ 383.1213; found 383.1227.

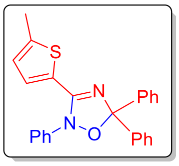

3-(5-Methylthiophen-2-yl)-2,5,5-triphenyl-2,5-dihydro-1,2,4-oxadiazole (3au). The reaction was performed following the General Procedure with nitrobenzene **1a** (10.2 μL, 0.1 mmol), LiO^{t}Bu (19.2 mg, 0.24 mmol), and **2u** (58.2 mg, 0.2 mmol) dissolved in THF (1 mL) at 100 °C for 12 h. The crude material was purified by flash chromatography on silica gel (eluted with hexanes:EtOAc = 200:1) to give the product (25.7 mg, 65% yield) as a colorless oil. ^{1}H NMR (500 MHz, $CDCl_3$): δ 7.58 – 7.56 (m, 4H), 7.35 – 7.31 (m, 9H), 7.29 – 7.27 (m, 2H), 6.93 (d, J = 3.6 Hz, 1H), 6.55 (dd, J = 3.7, 1.0 Hz, 1H), 2.42 (d, J = 0.7 Hz, 3H); $^{13}C\{^1H\}$ NMR (125 MHz, $CDCl_3$): δ 155.4, 145.5, 144.1, 143.7, 131.8, 129.4, 129.2, 128.2, 128.1, 128.0, 127.4, 126.7, 126.1, 109.2, 15.5; IR (thin film): 3060, 2922, 2852, 1628, 1537, 1488, 1448, 1071 cm^{-1}; HRMS (ESI) m/z: $[M + H]^+$ calcd for $C_{25}H_{21}N_2OS$ 397.1369; found 397.1374.

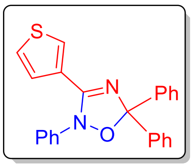

2,5,5-Triphenyl-3-(thiophen-3-yl)-2,5-dihydro-1,2,4-oxadiazole (3av). The reaction was performed following the General Procedure with nitrobenzene **1a** (10.2 μL, 0.1 mmol), LiO^{t}Bu (28.8 mg, 0.36 mmol), and **2v** (86.1 mg, 0.3 mmol) dissolved in THF (1 mL) at 100 °C for 12 h. The crude material was purified by flash chromatography on silica gel (eluted with hexanes:EtOAc = 200:1) to give the product (31.3 mg, 82% yield) as a white solid. mp = 131−132 °C. ^{1}H NMR (500 MHz, $CDCl_3$): δ 7.60 – 7.58 (m, 4H), 7.53 (dd, J = 2.9, 1.2 Hz, 1H), 7.41 (dd, J = 5.1, 1.1 Hz, 1H), 7.37 – 7.33 (m, 4H), 7.31 – 7.29 (m, 7H), 7.22 (dd, J = 5.1, 3.0 Hz, 1H); $^{13}C\{^1H\}$ NMR (125 MHz, $CDCl_3$): δ 155.9, 143.9, 143.8, 129.5, 129.4, 129.2, 129.0, 128.2, 128.1, 128.0, 127.6, 126.7, 125.9, 109.3; IR (thin film): 3080, 1626, 1594, 1527, 1488, 1449, 1284, 1177 cm^{-1}; HRMS (ESI) m/z: $[M + H]^+$ calcd for $C_{24}H_{19}N_2OS$ 383.1213; found 383.1219.

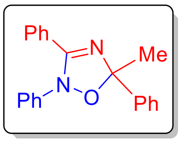

5-Methyl-2,3,5-triphenyl-2,5-dihydro-1,2,4-oxadiazole (3aa'). The reaction was performed following the General Procedure with **1a** (10.2 μL, 0.1 mmol), NaO^{t}Bu (34.6 mg, 0.36 mmol), and **2a'** (62.7 mg, 0.3 mmol) dissolved in DME (1 mL) at 100 °C for 12 h. The crude material was purified by flash chromatography on silica gel (eluted with hexanes:EtOAc = 200:1) to give the product (18.2 mg, 58% yield) as a colorless oil. ^{1}H NMR (500 MHz, $CDCl_3$) δ 7.66 – 7.64 (m, 2H), 7.55 – 7.53 (m, 2H), 7.32 – 7.20 (m, 6H), 7.18 – 7.10 (m, 5H), 1.89 (s, 3H); $^{13}C\{^1H\}$ NMR (125 MHz, $CDCl_3$) δ 159.8, 144.5, 144.2, 131.3, 129.3, 129.1, 128.6, 128.3, 128.1, 127.9, 126.4, 125.3, 107.9, 29.6, one resonance was not observed due to coincidental overlap; IR

(thin film): 3061, 2983, 2927, 1633, 1595, 1489, 1449, 1327, 1259, 1177, 762 cm^{-1}; HRMS (ESI) m/z: $[M + H]^+$ calcd for $C_{21}H_{19}N_2O$ 315.1492; found 315.1491.

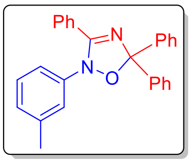

3,5,5-Triphenyl-2-(*m*-tolyl)-2,5-dihydro-1,2,4-oxadiazole (3ba). The reaction was performed following the General Procedure with **2a** (81.3 mg, 0.3 mmol), LiO^{t}Bu (28.8 mg, 0.36 mmol), and **1b** (11.8 μL, 0.1 mmol) dissolved in THF (1 mL) at 100 °C for 12 h. The crude material was purified by flash chromatography on silica gel (eluted with hexanes:EtOAc = 200:1) to give the product (33.1 mg, 85% yield) as a white solid. mp = 100−101 °C. ^{1}H NMR (500 MHz, CDCl$_3$): δ 7.79 – 7.76 (m, 2H), 7.60 – 7.58 (m, 4H), 7.39 – 7.28 (m, 9H), 7.12 – 7.07 (m, 2H), 7.02 (d, J = 7.6 Hz, 1H), 6.96 (dd, J = 7.8, 0.7 Hz, 1H), 2.24 (s, 3H); $^{13}C\{^1H\}$ NMR (125 MHz, CDCl$_3$): δ 160.3, 144.0, 143.7, 139.3, 131.2, 129.24, 129.20, 129.0, 128.5, 128.2, 128.1, 128.0, 127.9, 126.7, 124.1, 109.7, 21.3; IR (thin film): 2923, 2850, 1646, 1557, 1487, 1448, 1189 cm^{-1}; HRMS (ESI) m/z: $[M + H]^+$ calcd for $C_{27}H_{23}N_2O$ 391.1805; found 391.1801.

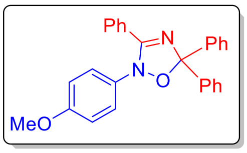

2-(4-Methoxyphenyl)-3,5,5-triphenyl-2,5-dihydro-1,2,4-oxadiazole (3ca). The reaction was performed following the General Procedure with **2a** (81.3 mg, 0.3 mmol), LiO^{t}Bu (28.8 mg, 0.36 mmol), and **1c** (15.3 mg, 0.1 mmol) dissolved in THF (1 mL) at 100 °C for 12 h. The crude material was purified by flash chromatography on silica gel (eluted with hexanes:EtOAc = 200:1) to give the product (34.5 mg, 85% yield) as a colorless oil. ^{1}H NMR (500 MHz, CDCl$_3$): δ 7.79 – 7.77 (m, 2H), 7.63 – 7.60 (m, 4H), 7.38– 7.34 (m, 5H), 7.31 – 7.28 (m, 4H), 7.21 – 7.17 (m, 2H), 6.77 – 6.74 (m, 2H), 3.74 (s, 3H); $^{13}C\{^1H\}$ NMR (125 MHz, CDCl$_3$): δ 160.7, 159.7, 144.2, 136.7, 131.1, 129.4, 129.2, 128.4, 128.2, 128.0, 127.9, 126.6, 114.4, 109.4, 55.5; IR (thin film): 3059, 2961, 1635, 1596, 1507, 1489, 1448, 1251, 1208 cm^{-1}; HRMS (ESI) m/z: $[M + H]^+$ calcd for $C_{27}H_{23}N_2O_2$ 407.1755; found 407.1781.

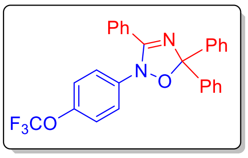

3,5,5-Triphenyl-2-(4-(trifluoromethoxy)phenyl)-2,5-dihydro-1,2,4-oxadiazole (3da). The reaction was performed following the General Procedure with **2a** (81.3 mg, 0.3 mmol), LiO^{t}Bu (28.8 mg, 0.36 mmol), and **1d** (14.3 μL, 0.1 mmol) dissolved in THF (1 mL) at 100 °C for 12 h. The crude material was purified by flash chromatography on silica gel (eluted with hexanes:EtOAc = 200:1) to give the product (40.5 mg, 88% yield) as a colorless oil. ^{1}H NMR (500 MHz, CDCl$_3$): δ 7.77 – 7.75 (m, 2H), 7.57 – 7.55 (m, 4H), 7.43 – 7.40 (m, 1H), 7.36 – 7.32 (m, 6H), 7.31 – 7.27 (m, 2H), 7.22 – 7.19 (m, 2H), 7.07 – 7.05 (m, 2H); $^{13}C\{^1H\}$ NMR (125 MHz, CDCl$_3$): δ 160.0, 148.4, 143.6, 142.5, 131.6, 129.2, 128.7, 128.3, 128.2, 128.1, 127.8,

126.6, 121.5, 120.4 (q, J_{C-F} = 257.9 Hz), 110.2; IR (thin film): 3070, 1632, 1557, 1477, 1432, 1333 cm^{-1}; HRMS (ESI) m/z: $[M + H]^+$ calcd for $C_{27}H_{20}F_3N_2O_2$ 461.1471; found 461.1481.

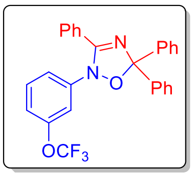

3,5,5-Triphenyl-2-(3-(trifluoromethoxy)phenyl)-2,5-dihydro-1,2,4-oxadiazole (3ea). The reaction was performed following the General Procedure with **2a** (81.3 mg, 0.3 mmol), LiO^{t}Bu (28.8 mg, 0.36 mmol), and **1e** (14.9 μL, 0.1 mmol) dissolved in THF (1 mL) at 100 °C for 12 h. The crude material was purified by flash chromatography on silica gel (eluted with hexanes:EtOAc = 200:1) to give the product (41.8 mg, 91% yield) as a white solid. mp = 90–92 °C. ^{1}H NMR (500 MHz, $CDCl_3$): δ 7.78 – 7.76 (m, 2H), 7.58 – 7.56 (m, 4H), 7.45 – 7.42 (m, 1H), 7.37 – 7.33 (m, 6H), 7.31 – 7.28 (m, 2H), 7.23 – 7.19 (m, 1H), 7.06 – 7.01 (m, 3H); $^{13}C\{^1H\}$ NMR (125 MHz, $CDCl_3$): δ 159.6, 149.4, 145.4, 143.4, 131.7, 130.2, 129.2, 128.8, 128.4, 128.2, 127.8, 126.6, 123.8, 120.4 (q, J_{C-F} = 258.1 Hz) 120.0, 118.6, 110.4; IR (thin film): 3078, 1634, 1577, 1493, 1450, 1341, 1259 cm^{-1}; HRMS (ESI) m/z: $[M + H]^+$ calcd for $C_{27}H_{20}F_3N_2O_2$ 461.1471; found 461.1485.

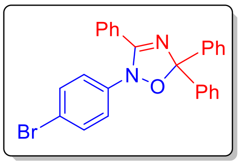

2-(4-Bromophenyl)-3,5,5-triphenyl-2,5-dihydro-1,2,4-oxadiazole (3fa). The reaction was performed following the General Procedure with **2a** (54.2 mg, 0.2 mmol), LiO^{t}Bu (19.2 mg, 0.24 mmol), and **1f** (20.0 mg, 0.1 mmol) dissolved in THF (1 mL) at 100 °C for 12 h. The crude material was purified by flash chromatography on silica gel (eluted with hexanes:EtOAc = 200:1) to give the product (32.2 mg, 71% yield) as a yellow solid. mp = 114–116 °C. ^{1}H NMR (500 MHz, $CDCl_3$): δ 7.77 – 7.74 (m, 2H), 7.57 – 7.55 (m, 4H), 7.43 – 7.39 (m, 1H), 7.36 – 7.27 (m, 10H), 7.06 – 7.03 (m, 2H); $^{13}C\{^1H\}$ NMR (125 MHz, $CDCl_3$): δ 159.9, 143.7, 143.1, 132.4, 131.5, 129.2, 128.7, 128.3, 128.2, 128.1, 127.8, 126.6, 121.9, 110.2; IR (thin film): 3056, 1629, 1591, 1481, 1448, 1334, 1068 cm^{-1}; HRMS (ESI) m/z: $[M + H]^+$ calcd for $C_{26}H_{20}BrN_2O$ 455.0754; found 455.0765.

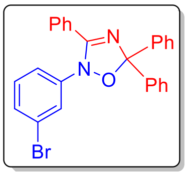

2-(3-Bromophenyl)-3,5,5-triphenyl-2,5-dihydro-1,2,4-oxadiazole (3ga). The reaction was performed following the General Procedure with **2a** (54.2 mg, 0.2 mmol), LiO^{t}Bu (19.2 mg, 0.24 mmol), and **1g** (20.0 mg, 0.1 mmol) dissolved in THF (1 mL) at 100 °C for 12 h. The crude material was purified by flash chromatography on silica gel (eluted with hexanes:EtOAc = 200:1) to give the product (33.6 mg, 74% yield) as a colorless oil. ^{1}H NMR (500 MHz, $CDCl_3$): δ 7.77 – 7.73 (m, 2H), 7.57 – 7.55 (m, 4H), 7.44 – 7.39 (m, 2H), 7.36 – 7.27 (m, 9H), 7.07 – 7.01 (m, 2H); $^{13}C\{^1H\}$ NMR (125 MHz, $CDCl_3$): δ 159.7, 145.2, 143.5, 131.6, 131.1,

130.4, 129.6, 129.2, 128.7, 128.3, 128.2, 127.8, 126.6, 124.8, 122.6, 110.2; IR (thin film): 3060, 1626, 1570, 1492, 1332, 1262, 1026 cm^{-1}; HRMS (ESI) m/z: $[M + H]^+$ calcd for $C_{26}H_{20}BrN_2O$ 455.0754; found 455.0760.

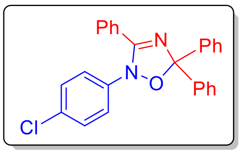

2-(4-Chlorophenyl)-3,5,5-triphenyl-2,5-dihydro-1,2,4-oxadiazole (3ha). The reaction was performed following the General Procedure with **2a** (54.2 mg, 0.2 mmol), LiO^{t}Bu (19.2 mg, 0.24 mmol), and **1h** (15.6 mg, 0.1 mmol) dissolved in THF (1 mL) at 100 °C for 12 h. The crude material was purified by flash chromatography on silica gel (eluted with hexanes:EtOAc = 200:1) to give the product (31.1 mg, 76% yield) as a white solid. mp = 132−133 °C. ^{1}H NMR (500 MHz, $CDCl_3$): δ 7.77 – 7.75 (m, 2H), 7.59 – 7.56 (m, 4H), 7.43 – 7.39 (m, 1H), 7.37 – 7.29 (m, 8H), 7.21 – 7.18 (m, 2H), 7.14 – 7.11 (m, 2H); $^{13}C\{^1H\}$ NMR (125 MHz, $CDCl_3$): δ 160.0, 143.7, 142.6, 133.9, 131.5, 129.4, 129.2, 128.7, 128.3, 128.11, 128.09, 127.8, 126.6, 110.2; IR (thin film): 3058, 1629, 1613, 1486, 1450, 1325, 1083 cm^{-1}; HRMS (ESI) m/z: $[M + H]^+$ calcd for $C_{26}H_{20}ClN_2O$ 411.1259; found 411.1275.

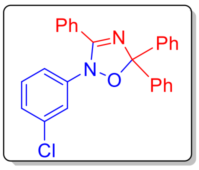

2-(3-Chlorophenyl)-3,5,5-triphenyl-2,5-dihydro-1,2,4-oxadiazole (3ia). The reaction was performed following the General Procedure with **2a** (54.2 mg, 0.2 mmol), LiO^{t}Bu (19.2 mg, 0.24 mmol), and **1i** (15.6 mg, 0.1 mmol) dissolved in THF (1 mL) at 100 °C for 12 h. The crude material was purified by flash chromatography on silica gel (eluted with hexanes:EtOAc = 200:1) to give the product (31.5 mg, 77% yield) as a colorless oil. ^{1}H NMR (500 MHz, $CDCl_3$): δ 7.77 – 7.76 (m, 2H), 7.59 – 7.56 (m, 4H), 7.44 – 7.40 (m, 1H), 7.37 – 7.33 (m, 6H), 7.31 – 7.28 (m, 2H), 7.24 (t, J = 2.0 Hz, 1H), 7.18 – 7.16 (m, 1H), 7.12 (t, J = 7.9 Hz, 1H), 7.00 – 6.98 (m, 1H); $^{13}C\{^1H\}$ NMR (125 MHz, $CDCl_3$): δ 159.7, 145.1, 143.6, 134.7, 131.6, 130.1, 129.2, 128.7, 128.3, 128.2, 127.8, 126.64, 126.61, 124.3, 110.2, one resonance was not observed due to coincidental overlap; IR (thin film): 3062, 1630, 1555, 1498, 1448, 1450, 1102 cm^{-1}; HRMS (ESI) m/z: $[M + H]^+$ calcd for $C_{26}H_{20}ClN_2O$ 411.1259; found 411.1273.

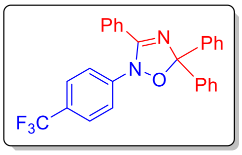

3,5,5-Triphenyl-2-(4-(trifluoromethyl)phenyl)-2,5-dihydro-1,2,4-oxadiazole (3ja). The reaction was performed following the General Procedure with **2a** (54.2 mg, 0.2 mmol), LiO^{t}Bu (19.2 mg, 0.24 mmol), and **1j** (19.1 mg, 0.1 mmol) dissolved in THF (1 mL) at 100 °C for 12 h. The crude material was purified by flash chromatography on silica gel (eluted with hexanes:EtOAc = 200:1) to give the product (36.4 mg, 82% yield) as a white solid. mp = 123−124 °C. ^{1}H NMR (500 MHz, $CDCl_3$): δ 7.79 – 7.76 (m, 2H), 7.57 – 7.54 (m,

4H), 7.47 – 7.44 (m, 3H), 7.39 – 7.32 (m, 6H), 7.31 – 7.28 (m, 2H), 7.21 (d, J = 8.3 Hz, 2H); $^{13}C\{^1H\}$ NMR (125 MHz, $CDCl_3$): δ 159.1, 146.8, 143.3, 131.8, 129.2, 128.92 (q, $J^2_{C(Ar)\text{-}F}$ = 32.7 Hz), 128.91, 128.4, 128.3, 127.9, 126.6, 126.3 (q, $J^3_{C(Ar)\text{-}F}$ = 3.7 Hz), 124.7, 123.9 (q, $J^1_{C\text{-}F}$ = 272.2 Hz), 110.4; IR (thin film): 3058, 1633, 1493, 1450, 1325, 1171, 1064 cm^{-1}; HRMS (ESI) m/z: $[M + H]^+$ calcd for $C_{27}H_{20}F_3N_2O$ 445.1522; found 445.1534.

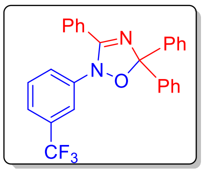

3,5,5-triphenyl-2-(3-(Trifluoromethyl)phenyl)-2,5-dihydro-1,2,4-oxadiazole (3ka). The reaction was performed following the General Procedure with **2a** (81.3 mg, 0.3 mmol), LiO^tBu (28.8 mg, 0.36 mmol), and **1k** (13.3 µL, 0.1 mmol) dissolved in THF (1 mL) at 100 °C for 12 h. The crude material was purified by flash chromatography on silica gel (eluted with hexanes:EtOAc = 200:1) to give the product (37.3 mg, 84% yield) as a colorless oil. ^1H NMR (500 MHz, $CDCl_3$): δ 7.79 – 7.76 (m, 2H), 7.58 – 7.56 (m, 4H), 7.49 (s, 1H), 7.45 – 7.41 (m, 2H), 7.37 – 7.33 (m, 6H), 7.32 – 7.28 (m, 3H), 7.24 (d, J = 8.2 Hz, 1H); $^{13}C\{^1H\}$ NMR (125 MHz, $CDCl_3$): δ 159.7, 144.6, 143.4, 131.72, 131.70 (q, $J^2_{C(Ar)\text{-}F}$ = 32.9 Hz), 129.7, 129.2, 128.8, 128.4, 128.2, 127.7, 126.6, 124.4 (q, $J^3_{C(Ar)\text{-}F}$ = 3.7 Hz), 123.6 (q, $J^1_{C\text{-}F}$ = 272.6 Hz), 123.1 (q, $J^3_{C(Ar)\text{-}F}$ = 3.8 Hz), 110.5, one resonance was not observed due to coincidental overlap; IR (thin film): 3066, 1642, 1598, 1492, 1466, 1323 cm^{-1}; HRMS (ESI) m/z: $[M + H]^+$ calcd for $C_{27}H_{20}F_3N_2O$ 445.1522; found 445.1530.

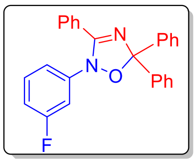

2-(3-Fluorophenyl)-3,5,5-triphenyl-2,5-dihydro-1,2,4-oxadiazole (3la). The reaction was performed following the General Procedure with **2a** (81.3 mg, 0.3 mmol), LiO^tBu (28.8 mg, 0.36 mmol), and **1l** (10.6 µL, 0.1 mmol) dissolved in THF (1 mL) at 100 °C for 12 h. The crude material was purified by flash chromatography on silica gel (eluted with hexanes:EtOAc = 200:1) to give the product (31.5 mg, 80% yield) as a colorless oil. ^1H NMR (500 MHz, $CDCl_3$): δ 7.78 – 7.76 (m, 2H), 7.57 – 7.55 (m, 4H), 7.43 – 7.40 (m, 1H), 7.36 – 7.32 (m, 6H), 7.30 – 7.27 (m, 2H), 7.18 – 7.14 (m, 1H), 6.93 – 6.88 (m, 3H); $^{13}C\{^1H\}$ NMR (125 MHz, $CDCl_3$): δ 162.7 (d, $J^1_{C\text{-}F}$ = 247.8 Hz), 159.7, 145.5 (d, $J^3_{C\text{-}F}$ = 9.0 Hz), 143.6, 131.6, 130.2 (d, $J^3_{C\text{-}F}$ = 9.0 Hz), 129.2, 128.7, 128.3, 128.2, 127.9, 126.6, 121.7 (d, $J^4_{C\text{-}F}$ = 3.2 Hz), 114.9 (d, $J^2_{C\text{-}F}$ = 21.2 Hz), 113.5 (d, $J^2_{C\text{-}F}$ = 23.2 Hz), 110.2; IR (thin film): 3061, 1629, 1593, 1483, 1446, 1329, 1268, 1178 cm^{-1}; HRMS (ESI) m/z: $[M + H]^+$ calcd for $C_{26}H_{20}FN_2O$ 395.1554; found 395.1572.

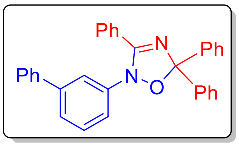

2-([1,1'-Biphenyl]-3-yl)-3,5,5-triphenyl-2,5-dihydro-1,2,4-oxadiazole (3ma). The reaction was performed following the General Procedure with **2a**

(81.3 mg, 0.3 mmol), LiO^{t}Bu (28.8 mg, 0.36 mmol), and **1m** (19.9 mg, 0.1 mmol) dissolved in THF (1 mL) at 100 °C for 12 h. The crude material was purified by flash chromatography on silica gel (eluted with hexanes:EtOAc = 200:1) to give the product (39.8 mg, 88% yield) as a white solid. mp = 118−120 °C. ^{1}H NMR (500 MHz, $CDCl_3$): δ 7.84 – 7.81 (m 2H), 7.63 – 7.61 (m, 4H), 7.45 – 7.43 (m, 1H), 7.41 – 7.29 (m, 16H), 7.20 – 7.18 (m, 1H); $^{13}C\{^1H\}$ NMR (125 MHz, $CDCl_3$): δ 160.4, 144.4, 144.0, 142.3, 140.0, 131.4, 129.6, 129.3, 128.9, 128.6, 128.3, 128.0, 127.8, 127.1, 126.9, 126.7, 125.7, 125.6, 110.1, one resonance was not observed due to coincidental overlap; IR (thin film): 3064, 1636, 1577, 1492, 1322, 1262, 1026 cm^{-1}; HRMS (ESI) m/z: [M + H]$^+$ calcd for $C_{32}H_{25}N_2O$ 453.1961; found 453.1967.

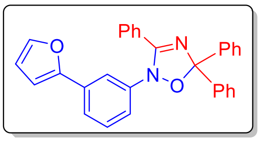

2-(3-(Furan-2-yl)phenyl)-3,5,5-triphenyl-2,5-dihydro-1,2,4-oxadiazole (3na). The reaction was performed following the General Procedure with **2a** (81.3 mg, 0.3 mmol), LiO^{t}Bu (28.8 mg, 0.36 mmol), and **1n** (18.9 mg, 0.1 mmol) dissolved in THF (1 mL) at 100 °C for 12 h. The crude material was purified by flash chromatography on silica gel (eluted with hexanes:EtOAc = 200:1) to give the product (37.6 mg, 85% yield) as a colorless oil. ^{1}H NMR (500 MHz, $CDCl_3$): δ 7.82 – 7.81 (m, 2H), 7.63 – 7.61 (m, 4H), 7.57 (t, J = 1.8 Hz, 1H), 7.53 – 7.51 (m, 1H), 7.43 (dd, J = 1.7, 0.5 Hz, 1H), 7.41 – 7.29 (m, 9H), 7.23 – 7.20 (m, 1H), 7.02 – 7.00 (m, 1H), 6.49 (dd, J = 3.3, 0.5 Hz, 1H), 6.44 (dd, J = 3.4, 1.8 Hz, 1H); $^{13}C\{^1H\}$ NMR (125 MHz, $CDCl_3$): δ 160.3, 152.9, 144.4, 143.9, 142.5, 132.1, 131.4, 129.5, 129.2, 128.6, 128.3, 128.1, 127.9, 126.7, 125.4, 123.5, 122.5, 111.8, 110.1, 105.9; IR (thin film): 3059, 1637, 1611, 1577, 1492, 1448, 1328, 1261 cm^{-1}; HRMS (ESI) m/z: [M + H]$^+$ calcd for $C_{30}H_{23}N_2O_2$ 443.1754; found 443.1779

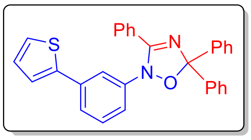

3,5,5-Triphenyl-2-(3-(thiophen-2-yl)phenyl)-2,5-dihydro-1,2,4-oxadiazole (3oa). The reaction was performed following the General Procedure with **2a** (81.3 mg, 0.3 mmol), LiO^{t}Bu (28.8 mg, 0.36 mmol), and **1o** (20.5 mg, 0.1 mmol) dissolved in THF (1 mL) at 100 °C for 12 h. The crude material was purified by flash chromatography on silica gel (eluted with hexanes:EtOAc = 200:1) to give the product (37.5 mg, 82% yield) as a colorless oil. ^{1}H NMR (500 MHz, $CDCl_3$): δ 7.73 – 7.71 (m, 2H), 7.53 – 7.52 (m, 4H), 7.39 (t, J = 1.8 Hz, 1H), 7.35 – 7.19 (m, 10H), 7.17 – 7.15 (m, 1H), 7.11 (t, J = 7.9 Hz, 1H), 7.01 (dd, J = 3.6, 1.1 Hz, 1H), 6.97 – 6.92 (m, 2H); $^{13}C\{^1H\}$ NMR (125 MHz, $CDCl_3$): δ 160.2, 144.5, 143.8, 143.2, 135.5, 131.4, 129.7, 129.2, 128.6, 128.3, 128.2, 128.1, 127.9, 126.7, 125.6, 125.5, 125.4, 124.2, 123.8, 110.2; IR (thin film): 1661, 1635, 1558, 1532, 1489, 1447, 1261 cm^{-1}; HRMS (ESI) m/z: [M + Na]$^+$ calcd for

$C_{30}H_{22}N_2NaOS$ 481.1345; found 481.1355.

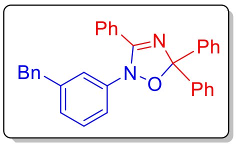

2-(3-Benzylphenyl)-3,5,5-triphenyl-2,5-dihydro-1,2,4-oxadiazole (3pa). The reaction was performed following the General Procedure with **2a** (54.2 mg, 0.2 mmol), LiO^{t}Bu (19.2 mg, 0.24 mmol), and **1p** (21.3 mg, 0.1 mmol) dissolved in THF (1 mL) at 100 °C for 12 h. The crude material was purified by flash chromatography on silica gel (eluted with hexanes:EtOAc = 200:1) to give the product (35.9 mg, 77% yield) as a colorless oil. ^{1}H NMR (500 MHz, $CDCl_3$): δ 7.74 (d, J = 7.5 Hz, 2H), 7.58 (d, J = 7.3 Hz, 4H), 7.41 – 7.37 (m, 1H), 7.35 – 7.32 (m, 4H), 7.31 – 7.27 (m, 4H), 7.25 – 7.19 (m, 3H), 7.15 (t, J = 7.8 Hz, 1H), 7.06 (d, J = 8.5 Hz, 1H), 7.03 – 7.02 (m, 2H), 6.98 (d, J = 6.9 Hz, 2H), 3.83 (s, 2H); $^{13}C\{^1H\}$ NMR (125 MHz, $CDCl_3$): δ 160.4, 143.9, 143.8, 142.3, 140.4, 131.2, 129.3, 129.2, 129.0, 128.6, 128.5, 128.2, 128.1, 128.0, 127.6, 126.7, 126.3, 124.6, 109.9, 41.6, one resonance was not observed due to coincidental overlap; IR (thin film): 3089, 2931, 1628, 1524, 1487, 1438, 1349 cm^{-1}; HRMS (ESI) m/z: [M + Na]$^+$ calcd for $C_{33}H_{26}N_2NaO$ 489.1937; found 489.1954.

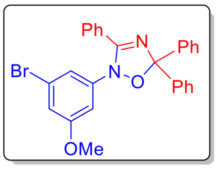

2-(3-Bromo-5-methoxyphenyl)-3,5,5-triphenyl-2,5-dihydro-1,2,4-oxadiazole (3qa). The reaction was performed following the General Procedure with **2a** (54.2 mg, 0.2 mmol), LiO^{t}Bu (19.2 mg, 0.24 mmol), and **1q** (23.0 mg, 0.1 mmol) dissolved in THF (1 mL) at 100 °C for 12 h. The crude material was purified by flash chromatography on silica gel (eluted with hexanes:EtOAc = 200:1) to give the product (39.7 mg, 82% yield) as a yellow solid. mp = 97−98 °C. ^{1}H NMR (500 MHz, $CDCl_3$): δ 7.80 – 7.77 (m, 2H), 7.58 – 7.56 (m, 4H), 7.45 – 7.41 (m, 1H), 7.37 – 7.33 (m, 6H), 7.31 – 7.28 (m, 2H), 6.95 (t, J = 1.7 Hz, 1H), 6.87 (t, J = 2.2 Hz, 1H), 6.57 – 6.56 (m, 1H), 3.55 (s, 3H); $^{13}C\{^1H\}$ NMR (125 MHz, $CDCl_3$): δ 160.5, 159.7, 145.8, 143.6, 131.6, 129.2, 128.7, 128.3, 128.2, 127.8, 126.6, 122.6, 121.7, 117.3, 110.6, 110.3, 55.6; IR (thin film): 3059, 2963, 1637, 1596, 1569, 1459, 1262, 1051 cm^{-1}; HRMS (ESI) m/z: [M + H]$^+$ calcd for $C_{27}H_{22}BrN_2O_2$ 485.0859; found 485.0876.

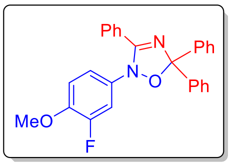

2-(3-Fluoro-4-methoxyphenyl)-3,5,5-triphenyl-2,5-dihydro-1,2,4-oxadiazole (3ra). The reaction was performed following the General Procedure with **2a** (54.2 mg, 0.2 mmol), LiO^{t}Bu (19.2 mg, 0.24 mmol), and **1r** (17.1 mg, 0.1 mmol) dissolved in THF (1 mL) at 100 °C for 12 h. The crude material was purified by flash chromatography on silica gel (eluted with hexanes:EtOAc = 200:1) to give the product (36.0 mg, 85% yield) as a colorless oil. ^{1}H NMR (500 MHz, $CDCl_3$): δ 7.79 (d, J = 7.6 Hz, 2H), 7.61 (d, J = 8.0 Hz, 4H), 7.41 –

7.29 (m, 9H), 7.03 – 6.99 (m, 2H), 6.78 (t, J = 8.8 Hz, 1H), 3.81 (s, 3H); $^{13}C\{^1H\}$ NMR (125 MHz, $CDCl_3$): δ 160.3, 151.8 (d, $J^1_{C(Ar)-F}$ = 248.8 Hz), 148.1 (d, J^2_{C-F} = 10.7 Hz), 143.9, 136.6 (d, J^3_{C-F} = 7.4 Hz), 131.4, 129.1, 128.5, 128.3, 128.0, 127.7, 126.5, 124.0 (d, J^3_{C-F} = 3.4 Hz), 115.7 (d, J^2_{C-F} = 19.1 Hz), 112.9 (d, J^4_{C-F} = 2.3 Hz), 109.8, 56.2; IR (thin film): 3060, 2963, 2842, 1637, 1512, 1449, 1310, 1264 cm^{-1}; HRMS (ESI) m/z: $[M + H]^+$ calcd for $C_{27}H_{22}FN_2O_2$ 425.1660; found 425.1686.

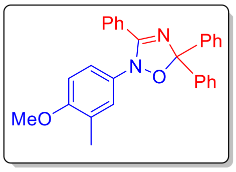

2-(4-Methoxy-3-methylphenyl)-3,5,5-triphenyl-2,5-dihydro-1,2,4-oxadiazole (3sa). The reaction was performed following the General Procedure with **2a** (81.3 mg, 0.3 mmol), LiO^{t}Bu (28.8 mg, 0.36 mmol), and **1s** (16.7 mg, 0.1 mmol) dissolved in THF (1 mL) at 100 °C for 12 h. The crude material was purified by flash chromatography on silica gel (eluted with hexanes:EtOAc = 200:1) to give the product (34.0 mg, 81% yield) as a colorless oil. ^1H NMR (500 MHz, $CDCl_3$): δ 7.78 (dd, J = 5.2, 3.3 Hz, 2H), 7.61 – 7.59 (m, 4H), 7.38 – 7.33 (m, 5H), 7.31 – 7.27 (m, 4H), 7.05 – 7.01 (m, 2H), 6.64 (d, J = 8.5 Hz, 1H), 3.76 (s, 3H), 2.08 (s, 3H); $^{13}C\{^1H\}$ NMR (125 MHz, $CDCl_3$): δ 160.7, 158.0, 144.3, 136.0, 131.1, 130.4, 129.2, 128.4, 128.20, 128.16, 127.9, 127.7, 126.72, 126.69, 110.0, 109.2, 55.5, 16.3; IR (thin film): 3063, 2965, 1621, 1566, 1501, 1489, 1448, 1250; HRMS (ESI) m/z: $[M + H]^+$ calcd for $C_{28}H_{25}N_2O_2$ 421.1911; found 421.1908.

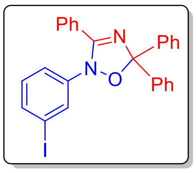

2-(3-Iodophenyl)-3,5,5-triphenyl-2,5-dihydro-1,2,4-oxadiazole (5). The reaction was performed following the General Procedure with **2a** (81.3 mg, 0.3 mmol), LiO^{t}Bu (28.8 mg, 0.36 mmol), and **4** (24.9 mg, 0.1 mmol) dissolved in THF (1 mL) at 100 °C for 12 h. The crude material was purified by flash chromatography on silica gel (eluted with hexanes:EtOAc = 200:1) to give the product (32.0 mg, 64% yield) as a white solid. mp = 93−95 °C. ^1H NMR (500 MHz, $CDCl_3$): δ 7.76 – 7.75 (m, 2H), 7.59 – 7.55 (m, 5H), 7.53 – 7.50 (m, 1H), 7.44 – 7.40 (m, 1H), 7.36 – 7.33 (m, 6H), 7.31 – 7.28 (m, 2H), 7.07 – 7.04 (m, 1H), 6.91 (t, J = 8.0 Hz, 1H); $^{13}C\{^1H\}$ NMR (125 MHz, $CDCl_3$): δ 159.7, 145.0, 143.5, 137.0, 135.6, 131.6, 130.5, 129.2, 128.7, 128.3, 128.2, 127.7, 126.6, 125.5, 110.2, 93.9; IR (thin film): 3058, 1616, 1565, 1491, 1465, 1447, 1325 cm^{-1}; HRMS (ESI) m/z: $[M + H]^+$ calcd for $C_{26}H_{20}IN_2O$ 503.0615; found 503.0609.

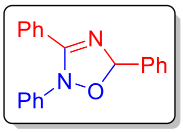

2,3,5-Triphenyl-2,5-dihydro-1,2,4-oxadiazole (3aA). The reaction was performed following the General Procedure with nitrobenzene **1a** (10.2 μL, 0.1 mmol), LiO^{t}Bu (28.8 mg, 0.36 mmol), and **2A** (58.5 mg, 0.3 mmol) dissolved in THF (1 mL) at 60 °C for 12 h. The crude material was purified by flash chromatography on silica gel (eluted with hexanes:EtOAc = 200:1) to give the product (24.3 mg, 81% yield) as a colorless oil. ^1H

NMR (500 MHz, $CDCl_3$): δ 7.80 – 7.78 (m, 2H), 7.57 – 7.54 (m, 2H), 7.43 – 7.31 (m, 10H), 7.26 – 7.25 (m, 1H), 6.97 (s, 1H). The NMR spectral data match the previously published data.[3]

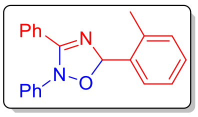

2,5-Diphenyl-3-(*o*-tolyl)-2,5-dihydro-1,2,4-oxadiazole (3aB). The reaction was performed following the General Procedure with nitrobenzene **1a** (10.2 µL, 0.1 mmol), LiO^{t}Bu (28.8 mg, 0.36 mmol), and **2B** (62.7 mg, 0.3 mmol) dissolved in THF (1 mL) at 60 °C for 12 h. The crude material was purified by flash chromatography on silica gel (eluted with hexanes:EtOAc = 200:1) to give the product (22.0 mg, 70% yield) as a colorless oil. ^{1}H NMR (500 MHz, $CDCl_3$): δ 7.72 – 7.70 (m, 2H), 7.51 – 7.49 (m, 1H), 7.35 – 7.31 (m, 1H), 7.27 – 7.24 (m, 2H), 7.22 – 7.21 (m, 4H), 7.18 – 7.15 (m, 3H), 7.13 – 7.11 (m, 1H), 7.08 (s, 1H), 2.43 (s, 3H); $^{13}C\{^1H\}$ NMR (125 MHz, $CDCl_3$): δ 161.8, 144.3, 136.9, 136.6, 131.4, 130.7, 129.4, 129.1, 129.0, 128.6, 128.3, 128.1, 126.8, 126.5, 126.2, 101.0, 19.3; IR (thin film): 3063, 2923, 1633, 1595, 1488, 1449, 1327, 751 cm^{-1}; HRMS (ESI) m/z: $[M + H]^+$ calcd for $C_{21}H_{19}N_2O$ 315.1492; found 315.1509.

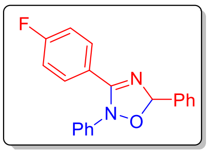

3-(4-Fluorophenyl)-2,5-diphenyl-2,5-dihydro-1,2,4-oxadiazole (3aC). The reaction was performed following the General Procedure with nitrobenzene **1a** (10.2 µL, 0.1 mmol), LiO^{t}Bu (28.8 mg, 0.36 mmol), and **2C** (63.9 mg, 0.3 mmol) dissolved in THF (1 mL) at 60 °C for 12 h. The crude material was purified by flash chromatography on silica gel (eluted with hexanes:EtOAc = 200:1) to give the product (19.4 mg, 61% yield) as a colorless oil. ^{1}H NMR (500 MHz, $CDCl_3$): δ 7.73 – 7.68 (m, 2H), 7.47 – 7.43 (m, 2H), 7.34 – 7.29 (m, 3H), 7.27 – 7.19 (m, 5H), 6.96 – 6.92 (m, 2H), 6.86 (s, 1H). The NMR spectral data match the previously published data and confirm the regiochemistry show for the major regioisomer.[3]

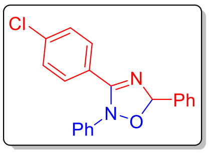

3-(4-Chlorophenyl)-2,5-diphenyl-2,5-dihydro-1,2,4-oxadiazole (3aD). The reaction was performed following the General Procedure with nitrobenzene **1a** (10.2 µL, 0.1 mmol), LiO^{t}Bu (28.8 mg, 0.36 mmol), and **2D** (68.7 mg, 0.3 mmol) dissolved in THF (1 mL) at 60 °C for 12 h. The crude material was purified by flash chromatography on silica gel (eluted with hexanes:EtOAc = 200:1) to give the product (18.1 mg, 55% yield) as a colorless oil. ^{1}H NMR (500 MHz, $CDCl_3$): δ 7.63 (d, J = 8.5 Hz, 2H), 7.45 (d, J = 6.8 Hz, 2H), 7.36 – 7.14 (m, 10H), 6.86 (s, 1H). The NMR spectral data match the previously published data and confirm the regiochemistry show for the major regioisomer.[3]

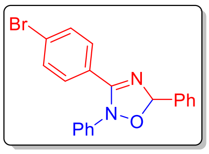

3-(4-Bromophenyl)-2,5-diphenyl-2,5-dihydro-1,2,4-oxadiazole (3aE). The reaction was performed following the General Procedure with nitrobenzene **1a** (10.2 μL, 0.1 mmol), LiO^{t}Bu (28.8 mg, 0.36 mmol), and **2E** (81.9 mg, 0.3 mmol) dissolved in THF (1 mL) at 60 °C for 12 h. The crude material was purified by flash chromatography on silica gel (eluted with hexanes:EtOAc = 200:1) to give the product (18.9 mg, 50% yield) as a white solid. mp = 100–101 °C. ^{1}H NMR (500 MHz, $CDCl_3$): δ 7.58 – 7.55 (m, 2H), 7.46 – 7.44 (m, 2H), 7.40 – 7.38 (m, 2H), 7.35 – 7.29 (m, 3H), 7.27 – 7.20 (m, 5H), 6.86 (s, 1H); $^{13}C\{^1H\}$ NMR (125 MHz, $CDCl_3$): δ 161.0, 144.0, 138.7, 131.95, 130.6, 129.6, 129.3, 128.8, 128.7, 127.0, 126.8, 126.6, 126.2, 103.4; IR (thin film): 2923, 1631, 1588, 1487, 1457, 1397, 1333, 753 cm^{-1}; HRMS (ESI) m/z: $[M + H]^+$ calcd for $C_{20}H_{16}BrN_2O$ 379.0441; found 379.0446. The regiochemistry of the major diastereomer was confirmed by X-ray. See the structure of 3aE after the NMR spectra.

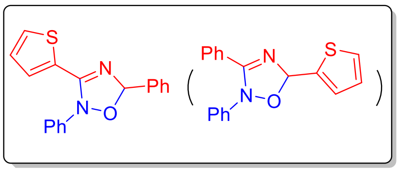

2,5-Diphenyl-3-(thiophen-2-yl)-2,5-dihydro-1,2,4-oxadiazole (3aF) and 2,3-Diphenyl-5-(thiophen-2-yl)-2,5-dihydro-1,2,4-oxadiazole (3aF'). The reaction was performed following the General Procedure with nitrobenzene **1a** (10.2 μL, 0.1 mmol), LiO^{t}Bu (28.8 mg, 0.36 mmol), and **2F** (60.3 mg, 0.3 mmol) dissolved in THF (1 mL) at 60 °C for 12 h. The crude material was purified by flash chromatography on silica gel (eluted with hexanes:EtOAc = 200:1) to give the product **3aF** (11.7 mg, 38% yield) and **3aF'** (11.7 mg, 38% yield) as colorless oils. ^{1}H NMR (500 MHz, $CDCl_3$) δ 7.71 – 7.67 (m, 2H), 7.48 – 7.46 (m, 2H), 7.37 – 7.35 (m, 2H), 7.33 – 7.28 (m, 8H), 7.26 – 7.20 (m, 7H), 7.17 – 7.14 (m, 2H), 7.10 (s, 1H), 7.08 (dd, J = 3.8, 1.1 Hz, 1H), 6.93 (dd, J = 5.0, 3.5 Hz, 1H), 6.84 (dd, J = 5.0, 3.8 Hz, 1H), 6.81 (s, 1H); $^{13}C\{^1H\}$ NMR (125 MHz, $CDCl_3$): δ 162.3, 156.9, 144.1, 144.0, 142.9, 138.6, 131.6, 131.5, 130.4, 129.9, 129.6, 129.4, 129.3, 129.2, 129.1, 128.7, 128.6, 128.5, 127.72, 127.67, 127.5, 127.2, 127.0, 126.9, 126.7, 126.6, 102.8, 99.0; IR (thin film): 3063, 3034, 2854, 1628, 1594, 1488, 1451, 1429, 1372, 1328, 1310, 1088, 758, 693 cm^{-1}; HRMS (ESI) m/z: $[M + H]^+$ calcd for $C_{18}H_{15}N_2OS$ 307.0900; found 307.0893 and 307.0912.

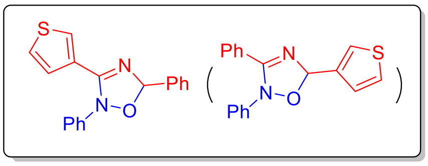

2,5-Diphenyl-3-(thiophen-3-yl)-2,5-dihydro-1,2,4-oxadiazole (3aG) and 2,3-Diphenyl-5-(thiophen-3-yl)-2,5-dihydro-1,2,4-oxadiazole (3aG'). The reaction was performed fol-

lowing the General Procedure with nitrobenzene **1a** (10.2 µL, 0.1 mmol), LiO^{t}Bu (28.8 mg, 0.36 mmol), and **2G** (60.3 mg, 0.3 mmol) dissolved in THF (1 mL) at 60 °C for 12 h. The crude material was purified by flash chromatography on silica gel (eluted with hexanes:EtOAc = 200:1) to give the product **3aG** (11.1 mg, 36% yield) and **3aG'** (11.1 mg, 36% yield) as colorless oils. ^{1}H NMR (500 MHz, $CDCl_3$) δ 7.70 – 7.68 (m, 2H), 7.48 – 7.46 (m, 3H), 7.36 – 7.35 (m, 1H), 7.32 – 7.27 (m, 9H), 7.26 – 7.20 (m, 8H), 7.16 – 7.14 (m, 3H), 6.94 (s, 1H), 6.82 (s, 1H); $^{13}C\{^1H\}$ NMR (125 MHz, $CDCl_3$): δ 161.8, 157.4, 144.3, 144.2, 140.8, 138.8, 131.5, 129.7, 129.6, 129.4, 129.2, 129.12, 129.08, 129.0, 128.7, 128.6, 128.3, 127.9, 127.8, 127.13, 127.06, 126.6, 126.4, 126.2, 126.1, 124.0, 102.9, 99.5; IR (thin film): 3081, 2925, 1617, 1593, 1488, 1450, 1370, 1300, 1041, 797, 694 cm^{-1}; HRMS (ESI) m/z: $[M + H]^+$ calcd for $C_{18}H_{15}N_2OS$ 307.0900; found 307.0897 and 307.0908.

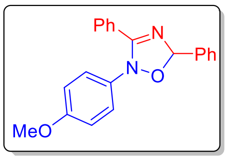

2-(4-Methoxyphenyl)-3,5-diphenyl-2,5-dihydro-1,2,4-oxadiazole (3cA). The reaction was performed following the General Procedure with **1c** (15.3 mg, 0.1 mmol), LiO^{t}Bu (28.8 mg, 0.36 mmol), and **2A** (58.5 mg, 0.3 mmol) dissolved in THF (1 mL) at 60 °C for 12 h. The crude material was purified by flash chromatography on silica gel (eluted with hexanes:EtOAc = 200:1) to give the product (25.1 mg, 76% yield) as a colorless oil. ^{1}H NMR (500 MHz, $CDCl_3$): δ 7.77 – 7.75 (m, 2H), 7.56 – 7.55 (m, 2H), 7.42 – 7.36 (m, 4H), 7.33 – 7.27 (m, 4H), 6.94 (s, 1H), 6.84 – 6.81 (m, 2H), 3.76 (s, 3H); $^{13}C\{^1H\}$ NMR (125 MHz, $CDCl_3$): δ 162.1, 159.8, 139.2, 137.0, 131.3, 129.1, 128.9, 128.7, 128.5, 127.9, 127.1, 114.7, 102.9, 55.6, one resonance was not observed due to coincidental overlap; IR (thin film): 2925, 2843, 1618, 1491, 1581, 1561, 1433, 1060 cm^{-1}; HRMS (ESI) m/z: $[M + H]^+$ calcd for $C_{21}H_{19}N_2O_2$ 331.1441; found 331.1458.

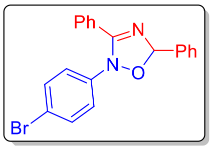

2-(4-Bromophenyl)-3,5-diphenyl-2,5-dihydro-1,2,4-oxadiazole (3gA). The reaction was performed following the General Procedure with **1g** (20.1 mg, 0.1 mmol), LiO^{t}Bu (28.8 mg, 0.36 mmol), and **2A** (58.5 mg, 0.3 mmol) dissolved in THF (1 mL) at 60 °C for 12 h. The crude material was purified by flash chromatography on silica gel (eluted with hexanes:EtOAc = 200:1) to give the product (32.9 mg, 87% yield) as a colorless oil. ^{1}H NMR (500 MHz, $CDCl_3$): δ 7.79 – 7.77 (m, 2H), 7.55 – 7.54 (m, 2H), 7.46 – 7.35 (m, 8H), 7.18 – 7.15 (m, 2H), 6.97 (s, 1H). The NMR spectral data match the previously published data.[3]

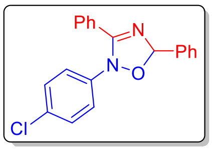

2-(4-Chlorophenyl)-3,5-diphenyl-2,5-dihydro-1,2,4-oxadiazole (3hA). The

reaction was performed following the General Procedure with **1h** (15.7 mg, 0.1 mmol), LiO^{t}Bu (28.8 mg, 0.36 mmol), and **2A** (58.5 mg, 0.3 mmol) dissolved in THF (1 mL) at 60 °C for 12 h. The crude material was purified by flash chromatography on silica gel (eluted with hexanes:EtOAc = 200:1) to give the product (30.1 mg, 90% yield) as a colorless oil. ^{1}H NMR (500 MHz, CDCl$_3$): δ 7.68 – 7.66 (m, 2H), 7.44 – 7.43 (m, 2H), 7.34 – 7.23 (m, 6H), 7.18 – 7.11 (m, 4H), 6.86 (s, 1H). The NMR spectral data match the previously published data.[3]

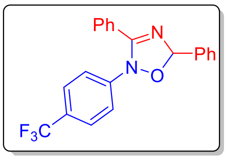

3,5-Diphenyl-2-(4-(trifluoromethyl)phenyl)-2,5-dihydro-1,2,4-oxadiazole (3jA). The reaction was performed following the General Procedure with **1j** (19.1 mg, 0.1 mmol), LiO^{t}Bu (28.8 mg, 0.36 mmol), and **2A** (58.5 mg, 0.3 mmol) dissolved in THF (1 mL) at 60 °C for 12 h. The crude material was purified by flash chromatography on silica gel (eluted with hexanes:EtOAc = 200:1) to give the product (29.1 mg, 79% yield) as a yellow oil. ^{1}H NMR (500 MHz, DMSO-d_6): δ 7.77 – 7.75 (m, 2H), 7.70 (d, J = 8.5 Hz, 2H), 7.55 – 7.52 (m, 1H), 7.50 – 7.40 (m, 9H), 7.03 (s, 1H); ^{13}C{^{1}H} NMR (125 MHz, DMSO-d_6): δ 160.1, 147.5, 138.3, 132.1, 129.3, 129.1, 128.7, 128.6, 127.6 (q, $J^2_{C(Ar)-F}$= 32.2 Hz), 127.2, 127.1, 126.7 (q, $J^3_{C(Ar)-F}$= 3.7 Hz), 125.0, 123.8 (q, J^1_{C-F}= 271.9 Hz), 102.8; IR (thin film): 2962, 1627, 1577, 1494, 1458, 1325, 1113, 1065 cm^{-1}; HRMS (ESI) m/z: [M + H]$^+$ calcd for $C_{21}H_{16}F_3N_2O$ 369.1209; found 369.1222.

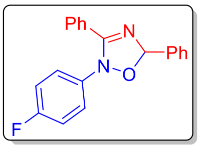

2-(4-Fluorophenyl)-3,5-diphenyl-2,5-dihydro-1,2,4-oxadiazole (3lA). The reaction was performed following the General Procedure with **1l** (14.1 mg, 0.1 mmol), LiO^{t}Bu (28.8 mg, 0.36 mmol), and **2A** (58.5 mg, 0.3 mmol) dissolved in THF (1 mL) at 60 °C for 12 h. The crude material was purified by flash chromatography on silica gel (eluted with hexanes:EtOAc = 200:1) to give the product (27.0 mg, 85% yield) as a yellow oil. ^{1}H NMR (500 MHz, CDCl$_3$): δ 7.77 – 7.76 (m, 2H), 7.56 – 7.54 (m, 2H), 7.44 – 7.30 (m, 8H), 7.03 – 6.98 (m, 2H), 6.96 (s, 1H); ^{13}C{^{1}H} NMR (125 MHz, CDCl$_3$): δ 162.3 (d, J^1_{C-F}= 249.0 Hz), 161.8, 140.4 (d, J^4_{C-F}= 3.2 Hz), 138.9, 131.6, 129.2, 129.0, 128.9 (d, J^3_{C-F}= 8.9 Hz), 128.74, 128.67, 127.6, 127.0, 116.4 (d, J^2_{C-F}= 22.8 Hz), 103.2; IR (thin film): 3036, 2866, 1631, 1596, 1502, 1458, 1371, 1214, 759 cm^{-1}; HRMS (ESI) m/z: [M + H]$^+$ calcd for $C_{20}H_{16}FN_2O$ 319.1241; found 319.1251.

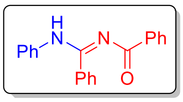

(*E*)-*N*-(Phenyl(phenylamino)methylene)benzamide (3AA). The reaction was performed following the General Procedure with nitrobenzene **1a** (10.2 μL, 0.1 mmol), LiO^{t}Bu (28.8 mg, 0.36 mmol), and **2A** (58.5 mg, 0.3 mmol) dissolved in THF (1 mL) at 100 °C for 12 h. The crude material was purified by flash chromatography on silica gel (eluted with hex-

anes:EtOAc = 200:1) to give the product (26.4 mg, 88% yield) as a yellow solid. ^{1}H NMR (500 MHz, $CDCl_3$): δ 12.11 (s, 1H), 8.25 (d, J = 7.3 Hz, 2H), 7.53 (d, J = 7.4 Hz, 2H), 7.47 – 7.44 (m, 1H), 7.39 – 7.33 (m, 3H), 7.25 (t, J = 7.5 Hz, 2H), 7.18 – 7.14 (m, 2H), 7.05 (t, J = 7.4 Hz, 1H), 6.97 (d, J = 7.4 Hz, 2H). The NMR spectral data match the previously published data.[3]

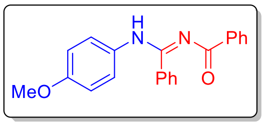

(*E*)-*N*-(((4-Methoxyphenyl)amino)(phenyl)methylene)benzamide (3CA). The reaction was performed following the General Procedure with **1c** (15.3 mg, 0.1 mmol), LiO^{t}Bu (28.8 mg, 0.36 mmol), and **2A** (58.5 mg, 0.3 mmol) dissolved in THF (1 mL) at 100 °C for 12 h. The crude material was purified by flash chromatography on silica gel (eluted with hexanes:EtOAc = 200:1) to give the product (28.4 mg, 86% yield) as a colorless oil. ^{1}H NMR (500 MHz, $CDCl_3$): δ 12.62 (s, 1H), 8.26 (d, J = 7.4 Hz, 2H), 7.52 (d, J = 7.3 Hz, 2H), 7.45 – 7.42 (m, 1H), 7.38 – 7.35 (m, 2H), 7.33 – 7.30 (m, 1H), 7.26 – 7.21 (m, 2H), 6.89 – 6.86 (m, 2H), 6.68 – 6.65 (m, 2H), 3.66 (s, 3H). The NMR spectral data match the previously published data.[4]

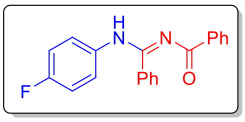

(*E*)-*N*-(((4-Fluorophenyl)amino)(phenyl)methylene)benzamide (3LA). The reaction was performed following the General Procedure with **1l** (14.1 mg, 0.1 mmol), LiO^{t}Bu (28.8 mg, 0.36 mmol), and **2A** (58.5 mg, 0.3 mmol) dissolved in THF (1 mL) at 100 °C for 12 h. The crude material was purified by flash chromatography on silica gel (eluted with hexanes:EtOAc = 200:1) to give the product (26.1 mg, 82% yield) as a yellow oil. ^{1}H NMR (500 MHz, $CDCl_3$): δ 12.11 (s, 1H), 8.24 (d, J = 7.4 Hz, 2H), 7.51 (d, J = 7.3 Hz, 2H), 7.48 – 7.44 (m, 1H), 7.40 – 7.34 (m, 3H), 7.28 – 7.24 (m, 2H), 6.95 – 6.93 (m, 2H), 6.88 – 6.83 (m, 2H). The NMR spectral data match the previously published data.[4]

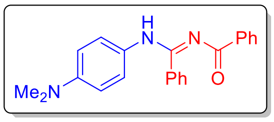

(*E*)-*N*-(((4-(Dimethylamino)phenyl)amino)(phenyl)methylene)benzamide (3TA). The reaction was performed following the General Procedure with **1t** (16.6 mg, 0.1 mmol), LiO^{t}Bu (28.8 mg, 0.36 mmol), and **2A** (58.5 mg, 0.3 mmol) dissolved in THF (1 mL) at 100 °C for 12 h. The crude material was purified by flash chromatography on silica gel (eluted with hexanes:EtOAc = 200:1) to give the product (23.7 mg, 69% yield) as a yellow solid. mp = 164–165 °C. ^{1}H NMR (500 MHz, $CDCl_3$): δ 12.99 (s, 1H), 8.28 (d, J = 7.4 Hz, 2H), 7.58 (d, J = 7.2 Hz, 2H), 7.44 – 7.42 (m, 1H), 7.39 – 7.36 (m, 2H), 7.34 – 7.30 (m, 1H), 7.26 – 7.23 (m, 2H), 6.74 (s, 2H), 6.48 – 6.47 (m, 2H), 2.84 (s, 6H); $^{13}C\{^{1}H\}$ NMR (125 MHz, $CDCl_3$): δ 179.7, 148.7, 137.7, 135.1, 132.0, 130.8, 129.9, 129.7, 129.0, 128.3, 128.2, 127.6, 125.3, 112.6, 40.6; IR (thin film): 2920, 1734, 1588, 1552, 1436, 1360, 1325, 1062 cm^{-1}; HRMS (ESI) m/z: $[M + H]^{+}$

calcd for $C_{22}H_{22}N_3O$ 344.1757; found 344.1764.

Procedure for competition reaction with nitrosoarene

An oven-dried 10 mL vial equipped with a stir bar was charged with aldimine (0.1 mmol, 1 equiv), 1-methyl-4-nitrobenzene (41.1 mg, 0.3 mmol, 3 equiv), 1-methoxy-4-nitrosobenzene (41.1 mg, 0.3 mmol, 3 equiv) and NaO^{t}Bu (28.8 mg, 0.3 mmol, 3 equiv) under a nitrogen atmosphere in a glovebox. DME (1 mL) was added to the reaction by syringe at room temperature. The color of the reaction mixture turned to light blue. The vial was capped, removed from the glovebox, and stirred for 12 h at 100 °C. After cooling to room temperature, the reaction mixture was quenched with three drops of H_2O and the vial was open to the air, passed through a short pad of silica gel and eluted with ethyl acetate (1 mL × 3). The combined organic solution was concentrated under reduced pressure and the crude material was purified by flash chromatography on silica gel (eluted with hexanes:EtOAc = 200:1) to give the product **3ca** (28.8 mg, 71% yield) as a colorless oil.

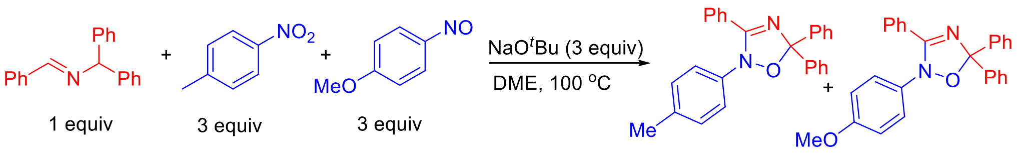

Computational Details

The B3LYP density functional theory (DFT) method[5] and the 6-31G(d) basis set[6] was employed to carry out geometry optimizations. Vibrational frequency analyses at the same level of theory were performed on all the optimized geometries to characterize stationary points as local minima (no imaginary frequency) or transition states (one imaginary frequency). In addition, intrinsic reaction coordinate (IRC) calculations[7] were used to verify that the transition state connects with appropriate reactant and product. To consider the effect of solvation, single-point energy calculations with the SMD[8] continuum solvation model (in THF solvent) were used. Moreover, the M06-2X method[9] with a larger basis set, 6-311++G(d,p), was employed in these single-point energy calculations. The solvation Gibbs energy was used for discussion and its value was obtained from the addition of solvation single-point energy and gas-phase thermal correction to Gibbs energy obtained from the vibrational frequency analysis. All calculations were carried out with the Gaussian 09 suite of programs.[10]

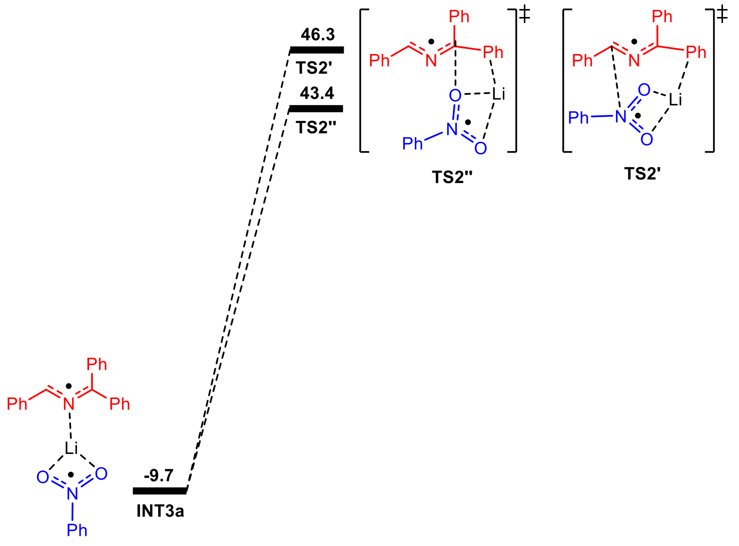

Supplementary Figure 1. Energy profiles for the addition of **INT4** to the key 2-azaallyl radical (**2a′**).

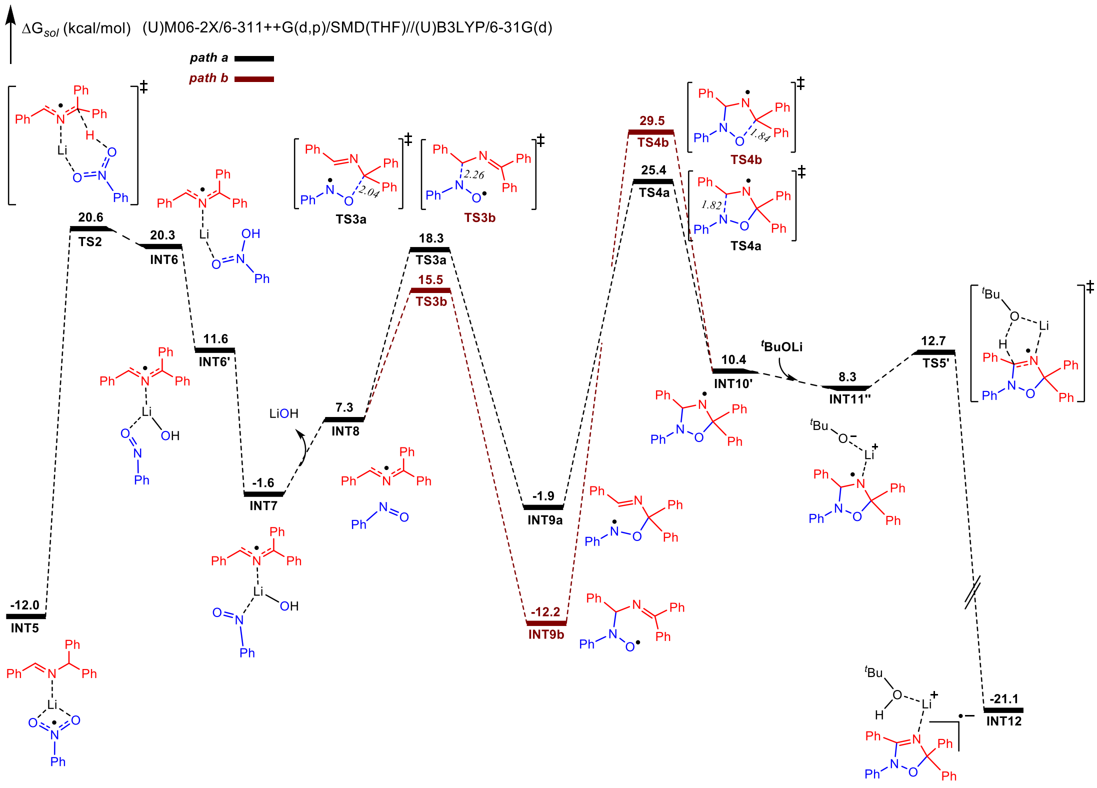

Supplementary Figure 2. Energy profiles for the formation of **INT12** via intramolecular cyclization pathways from **INT9a/9b** followed by deprotonation step. Bond distances are shown in Å.

Cartesian Coordinates and Energies

1a

Center Number	Atomic Number	Atomic Type	Coordinates (Angstroms) X	Y	Z
1	6	0	-1.820987	-1.212289	-0.000002

2	6	0	-0.427592	-1.220789	0.000039
3	6	0	0.244570	-0.000006	0.000055
4	6	0	-0.427600	1.220795	0.000022
5	6	0	-1.820978	1.212293	-0.000025
6	6	0	-2.516451	-0.000006	-0.000034
7	1	0	-2.363262	-2.153235	-0.000009
8	1	0	0.140240	-2.143090	0.000066
9	1	0	0.140260	2.143080	0.000036
10	1	0	-2.363280	2.153225	-0.000053
11	1	0	-3.602919	0.000010	-0.000068
12	7	0	1.718018	-0.000000	0.000108
13	8	0	2.289814	1.089596	-0.000040
14	8	0	2.289820	-1.089594	-0.000093

Zero-point correction= 0.103581 (Hartree/Particle)
Thermal correction to Energy= 0.110353
Thermal correction to Enthalpy= 0.111297
Thermal correction to Gibbs Free Energy= 0.071755
Sum of electronic and zero-point Energies= -436.647004
Sum of electronic and thermal Energies= -436.640232
Sum of electronic and thermal Enthalpies= -436.639287
Sum of electronic and thermal Free Energies= -436.678830
M06-2X/6-311++G(d,p)/SMD(THF)//B3LYP/6-31G(d) energy = -436.6997223

2a

Center Number	Atomic Number	Atomic Type	Coordinates (Angstroms) X	Y	Z
1	6	0	-5.500750	-0.735638	-0.208804
2	6	0	-5.052045	0.533454	-0.576693
3	6	0	-3.707096	0.867721	-0.417887
4	6	0	-2.797752	-0.061016	0.108227
5	6	0	-3.259247	-1.335653	0.477824
6	6	0	-4.600380	-1.668387	0.319046
7	1	0	-6.548086	-0.999227	-0.330747
8	1	0	-5.747311	1.261273	-0.986279
9	1	0	-3.354304	1.856203	-0.704458
10	1	0	-2.548012	-2.045246	0.888376
11	1	0	-4.950126	-2.656217	0.607496
12	6	0	-1.381840	0.323739	0.258855
13	1	0	-1.143415	1.353114	-0.041650
14	7	0	-0.504362	-0.485477	0.706051
15	6	0	0.894860	-0.078728	0.885500
16	6	0	1.828013	-1.178168	0.370752
17	6	0	1.377878	-2.200118	-0.469991
18	6	0	3.179399	-1.157971	0.743683
19	6	0	2.261361	-3.179207	-0.933227
20	1	0	0.329294	-2.234528	-0.745808
21	6	0	4.060710	-2.135394	0.286211
22	1	0	3.543418	-0.365302	1.393906
23	6	0	3.603709	-3.151420	-0.557602
24	1	0	1.894660	-3.967062	-1.586514
25	1	0	5.104266	-2.105591	0.589189
26	1	0	4.289481	-3.914919	-0.915480
27	6	0	1.280138	1.299040	0.345147
28	6	0	1.355452	2.397501	1.210095
29	6	0	1.526510	1.503874	-1.020374
30	6	0	1.676070	3.669444	0.730027
31	1	0	1.163820	2.254638	2.271771

32	6	0	1.841885	2.772823	-1.504452
33	1	0	1.482792	0.659250	-1.702828
34	6	0	1.919790	3.860159	-0.630132
35	1	0	1.737167	4.507843	1.419104
36	1	0	2.034036	2.912603	-2.565157
37	1	0	2.171951	4.847655	-1.007468
38	1	0	1.030911	-0.042490	1.976949

Zero-point correction= 0.313136 (Hartree/Particle)
Thermal correction to Energy= 0.330116
Thermal correction to Enthalpy= 0.331061
Thermal correction to Gibbs Free Energy= 0.265344
Sum of electronic and zero-point Energies= -826.793730
Sum of electronic and thermal Energies= -826.776749
Sum of electronic and thermal Enthalpies= -826.775805
Sum of electronic and thermal Free Energies= -826.841522
M06-2X/6-311++G(d,p)/SMD(THF)//B3LYP/6-31G(d) energy = -826.9821687

LiO^{t}Bu

Center Number	Atomic Number	Atomic Type	Coordinates (Angstroms) X	Y	Z
1	6	0	-0.116073	0.000023	0.000145
2	6	0	-0.629948	1.338109	0.574703
3	1	0	-1.726299	1.390838	0.595796
4	1	0	-0.258597	1.471201	1.597950
5	1	0	-0.256191	2.171584	-0.032223
6	6	0	-0.630625	-0.170331	-1.445846
7	1	0	-0.258502	0.649027	-2.072629
8	1	0	-0.258289	-1.113041	-1.864576
9	1	0	-1.727044	-0.176954	-1.501912
10	6	0	-0.632739	-1.166301	0.870464
11	1	0	-0.260680	-2.119128	0.474962
12	1	0	-0.261947	-1.058209	1.896895
13	1	0	-1.729221	-1.210261	0.902349
14	8	0	1.271117	-0.001235	0.000734
15	3	0	2.874713	-0.001392	0.000240

Zero-point correction= 0.126445 (Hartree/Particle)
Thermal correction to Energy= 0.134087
Thermal correction to Enthalpy= 0.135031
Thermal correction to Gibbs Free Energy= 0.095819
Sum of electronic and zero-point Energies= -240.507699
Sum of electronic and thermal Energies= -240.500056
Sum of electronic and thermal Enthalpies= -240.499112
Sum of electronic and thermal Free Energies= -240.538325
M06-2X/6-311++G(d,p)/SMD(THF)//B3LYP/6-31G(d) energy = -240.6032029

^{t}BuOH

Center Number	Atomic Number	Atomic Type	Coordinates (Angstroms) X	Y	Z
1	1	0	0.945239	-0.000816	1.727995
2	6	0	-0.005349	0.000008	0.013785
3	6	0	0.688079	-1.266820	-0.510022
4	1	0	0.651294	-1.323016	-1.604280
5	1	0	1.745358	-1.283000	-0.213219
6	1	0	0.205672	-2.159813	-0.099187

7	6	0	-1.490491	0.002505	-0.356656
8	1	0	-1.985081	-0.882927	0.056045
9	1	0	-1.981978	0.889909	0.055502
10	1	0	-1.622994	0.002380	-1.443815
11	6	0	0.692493	1.264232	-0.510449
12	1	0	0.213329	2.159063	-0.099819
13	1	0	1.749874	1.276714	-0.213807
14	1	0	0.655796	1.320254	-1.604715
15	8	0	0.014388	0.000212	1.452419

Zero-point correction= 0.136172 (Hartree/Particle)
Thermal correction to Energy= 0.142900
Thermal correction to Enthalpy= 0.143844
Thermal correction to Gibbs Free Energy= 0.107168
Sum of electronic and zero-point Energies= -233.534786
Sum of electronic and thermal Energies= -233.528058
Sum of electronic and thermal Enthalpies= -233.527114
Sum of electronic and thermal Free Energies= -233.563790
M06-2X/6-311++G(d,p)/SMD(THF)//B3LYP/6-31G(d) energy = -233.6386807

INT1

Center Number	Atomic Number	Atomic Type	Coordinates (Angstroms) X	Y	Z
1	6	0	2.483469	-4.532977	-0.522085
2	6	0	1.136151	-4.897214	-0.480521
3	6	0	0.169754	-3.928407	-0.224472
4	6	0	0.533605	-2.586665	-0.006013
5	6	0	1.895327	-2.227049	-0.046779
6	6	0	2.855076	-3.202334	-0.305908
7	1	0	3.243171	-5.284249	-0.721789
8	1	0	0.840666	-5.929331	-0.647073
9	1	0	-0.881283	-4.207621	-0.192400
10	1	0	2.227120	-1.200816	0.119107
11	1	0	3.902220	-2.915301	-0.337358
12	6	0	-0.555383	-1.639563	0.253876
13	1	0	-1.554006	-2.090110	0.266941
14	7	0	-0.418411	-0.379882	0.454489
15	6	0	-1.601109	0.463731	0.760211
16	6	0	-1.386074	1.846601	0.146450
17	6	0	-0.709932	2.014596	-1.070879
18	6	0	-1.876561	2.982556	0.805257
19	6	0	-0.523132	3.290455	-1.612736
20	1	0	-0.327421	1.146207	-1.603457
21	6	0	-1.697058	4.253488	0.262549
22	1	0	-2.403280	2.867672	1.749909
23	6	0	-1.016884	4.411683	-0.948199
24	1	0	0.012400	3.400941	-2.551262
25	1	0	-2.079667	5.123129	0.789810
26	1	0	-0.868109	5.403591	-1.365284
27	6	0	-2.966341	-0.129109	0.416020
28	6	0	-3.771430	-0.655872	1.433786
29	6	0	-3.435763	-0.176572	-0.904702
30	6	0	-5.018193	-1.214928	1.144740
31	1	0	-3.420431	-0.626143	2.463257
32	6	0	-4.678128	-0.737532	-1.196672
33	1	0	-2.831975	0.238930	-1.706528
34	6	0	-5.474281	-1.257044	-0.172559
35	1	0	-5.630585	-1.613834	1.948893

36	1	0	-5.028759	-0.762873	-2.224923
37	1	0	-6.444834	-1.688655	-0.401371
38	1	0	-1.579365	0.598302	1.850788
39	6	0	4.191025	1.316536	0.292594
40	6	0	5.105058	0.106483	-0.018136
41	1	0	6.168680	0.377815	-0.052702
42	1	0	4.974226	-0.665242	0.750972
43	1	0	4.828901	-0.325693	-0.988094
44	6	0	4.405080	2.397808	-0.792600
45	1	0	4.119654	1.998845	-1.773759
46	1	0	3.767178	3.265032	-0.581793
47	1	0	5.446991	2.741655	-0.848835
48	6	0	4.579364	1.899471	1.671438
49	1	0	3.939101	2.759716	1.902473
50	1	0	4.422544	1.143665	2.450536
51	1	0	5.627055	2.227568	1.713165
52	8	0	2.867889	0.917084	0.306649
53	3	0	1.217906	0.850149	0.349401

Zero-point correction= 0.441166 (Hartree/Particle)
Thermal correction to Energy= 0.467441
Thermal correction to Enthalpy= 0.468385
Thermal correction to Gibbs Free Energy= 0.379993
Sum of electronic and zero-point Energies= -1067.342114
Sum of electronic and thermal Energies= -1067.315839
Sum of electronic and thermal Enthalpies= -1067.314895
Sum of electronic and thermal Free Energies= -1067.403287
M06-2X/6-311++G(d,p)/SMD(THF)//B3LYP/6-31G(d) energy = -1067.612713

INT1′

Center Number	Atomic Number	Atomic Type	Coordinates (Angstroms) X	Y	Z
1	6	0	-4.459145	-4.299320	-0.330410
2	6	0	-5.159211	-3.163413	0.078532
3	6	0	-4.477536	-1.963906	0.281785
4	6	0	-3.092371	-1.886636	0.074715
5	6	0	-2.395283	-3.036640	-0.333259
6	6	0	-3.075914	-4.232718	-0.534067
7	1	0	-4.986954	-5.236245	-0.487224
8	1	0	-6.232532	-3.211770	0.240276
9	1	0	-5.020129	-1.077023	0.602138
10	1	0	-1.321652	-2.972238	-0.478258
11	1	0	-2.530175	-5.119031	-0.846212
12	6	0	-2.407587	-0.601236	0.293460
13	1	0	-3.042893	0.206636	0.681271
14	7	0	-1.166196	-0.438824	0.048651
15	6	0	-0.498884	0.835167	0.330093
16	6	0	0.549295	1.116202	-0.752832
17	6	0	0.665416	0.316199	-1.898749
18	6	0	1.445010	2.188022	-0.588643
19	6	0	1.648940	0.583778	-2.861313
20	1	0	-0.002440	-0.529522	-2.013963
21	6	0	2.429461	2.454178	-1.544126
22	1	0	1.377238	2.809975	0.299634
23	6	0	2.535474	1.651392	-2.688252
24	1	0	1.731325	-0.055016	-3.735987
25	1	0	3.125907	3.272155	-1.386270
26	1	0	3.310817	1.846239	-3.422845

27	6	0	-1.398440	2.047287	0.568277
28	6	0	-1.584396	2.544163	1.863521
29	6	0	-2.077489	2.663491	-0.493610
30	6	0	-2.422194	3.637765	2.096632
31	1	0	-1.067768	2.073132	2.697065
32	6	0	-2.916183	3.752792	-0.263879
33	1	0	-1.943266	2.289485	-1.505680
34	6	0	-3.089330	4.244744	1.032920
35	1	0	-2.550219	4.013433	3.108220
36	1	0	-3.432392	4.221420	-1.097542
37	1	0	-3.739946	5.096635	1.210799
38	1	0	0.066566	0.661370	1.258119
39	6	0	4.916219	-1.380507	0.936555
40	6	0	5.198336	-2.758342	0.292378
41	1	0	5.931100	-3.349238	0.858835
42	1	0	4.266808	-3.333713	0.226711
43	1	0	5.580485	-2.618694	-0.726283
44	6	0	6.236064	-0.577076	1.012175
45	1	0	6.629202	-0.415971	0.000898
46	1	0	6.045308	0.405855	1.460222
47	1	0	7.008311	-1.084189	1.606651
48	6	0	4.370585	-1.593466	2.368514
49	1	0	4.160557	-0.621582	2.832049
50	1	0	3.430617	-2.157213	2.326381
51	1	0	5.072814	-2.139263	3.013467
52	8	0	3.991136	-0.690684	0.181236
53	3	0	2.907523	0.123365	-0.718471

Zero-point correction= 0.440493 (Hartree/Particle)
Thermal correction to Energy= 0.467204
Thermal correction to Enthalpy= 0.468148
Thermal correction to Gibbs Free Energy= 0.377079
Sum of electronic and zero-point Energies= -1067.328194
Sum of electronic and thermal Energies= -1067.301483
Sum of electronic and thermal Enthalpies= -1067.300539
Sum of electronic and thermal Free Energies= -1067.391608
M06-2X/6-311++G(d,p)/SMD(THF)//B3LYP/6-31G(d) energy = -1067.600282

TS1

Center Number	Atomic Number	Atomic Type	Coordinates (Angstroms) X	Y	Z
1	6	0	-5.802137	0.672191	0.687772
2	6	0	-5.517430	-0.461809	-0.074396
3	6	0	-4.197141	-0.878519	-0.240626
4	6	0	-3.136288	-0.171351	0.349838
5	6	0	-3.437302	0.969209	1.117102
6	6	0	-4.754233	1.384425	1.282555
7	1	0	-6.830153	0.999446	0.819568
8	1	0	-6.323800	-1.022592	-0.540376
9	1	0	-3.978263	-1.762852	-0.835685
10	1	0	-2.619958	1.514481	1.578022
11	1	0	-4.969890	2.267672	1.879065
12	6	0	-1.759422	-0.645689	0.152921
13	1	0	-1.659114	-1.558526	-0.449294
14	7	0	-0.740264	-0.041456	0.651436
15	6	0	0.591705	-0.448485	0.407602
16	6	0	1.568166	0.110495	1.366620
17	6	0	1.272001	1.287052	2.108106

18	6	0	2.929119	-0.299102	1.350580
19	6	0	2.278985	2.053429	2.696619
20	1	0	0.238686	1.610733	2.149919
21	6	0	3.934328	0.470299	1.943191
22	1	0	3.199597	-1.201679	0.812329
23	6	0	3.629708	1.676524	2.594223
24	1	0	2.012315	2.964503	3.227120
25	1	0	4.966396	0.133852	1.881772
26	1	0	4.412109	2.279342	3.044545
27	6	0	0.849972	-1.867234	-0.060956
28	6	0	1.446333	-2.136028	-1.301454
29	6	0	0.495749	-2.959553	0.751405
30	6	0	1.687369	-3.449088	-1.716499
31	1	0	1.729042	-1.304585	-1.940561
32	6	0	0.729369	-4.270751	0.339235
33	1	0	0.030862	-2.771048	1.716256
34	6	0	1.328221	-4.520201	-0.898750
35	1	0	2.153634	-3.632370	-2.681548
36	1	0	0.448313	-5.099073	0.985185
37	1	0	1.512604	-5.541851	-1.221095
38	1	0	1.028192	0.488315	-0.593018
39	6	0	0.932752	2.095036	-2.194674
40	6	0	-0.096582	3.019971	-1.521103
41	1	0	-0.689835	3.571543	-2.260602
42	1	0	-0.779253	2.436599	-0.894718
43	1	0	0.411576	3.754286	-0.882047
44	6	0	1.947350	2.919475	-3.001370
45	1	0	2.463479	3.638625	-2.351552
46	1	0	2.699802	2.261837	-3.450772
47	1	0	1.458586	3.485020	-3.803923
48	6	0	0.217965	1.094280	-3.118144
49	1	0	0.943982	0.427617	-3.596900
50	1	0	-0.489145	0.481273	-2.548393
51	1	0	-0.341711	1.612380	-3.905723
52	8	0	1.661169	1.383689	-1.192316
53	3	0	2.677697	1.773427	0.240706

Zero-point correction= 0.435657 (Hartree/Particle)
Thermal correction to Energy= 0.461150
Thermal correction to Enthalpy= 0.462095
Thermal correction to Gibbs Free Energy= 0.378431
Sum of electronic and zero-point Energies= -1067.307564
Sum of electronic and thermal Energies= -1067.282071
Sum of electronic and thermal Enthalpies= -1067.281127
Sum of electronic and thermal Free Energies= -1067.364790
M06-2X/6-311++G(d,p)/SMD(THF)//B3LYP/6-31G(d) energy = -1067.581464

INT2′

Center Number	Atomic Number	Atomic Type	Coordinates (Angstroms) X	Y	Z
1	6	0	-5.332949	1.015188	-1.026031
2	6	0	-4.770409	2.215115	-0.582750
3	6	0	-3.390785	2.337009	-0.441982
4	6	0	-2.524923	1.261612	-0.737242
5	6	0	-3.110584	0.058034	-1.192991
6	6	0	-4.489737	-0.059123	-1.333056
7	1	0	-6.409591	0.919128	-1.136933
8	1	0	-5.411130	3.061295	-0.345862

9	1	0	-2.963490	3.276870	-0.098239
10	1	0	-2.462334	-0.772667	-1.455232
11	1	0	-4.913618	-0.993732	-1.693916
12	6	0	-1.089546	1.439886	-0.584177
13	1	0	-0.755575	2.453293	-0.341945
14	7	0	-0.233980	0.464336	-0.759932
15	6	0	1.125997	0.556480	-0.627143
16	6	0	1.856472	-0.598295	-1.058709
17	6	0	1.285625	-1.525504	-2.002447
18	6	0	3.110501	-1.004287	-0.483685
19	6	0	1.845551	-2.777827	-2.239120
20	1	0	0.367188	-1.239758	-2.501160
21	6	0	3.652590	-2.264237	-0.720199
22	1	0	3.607230	-0.335221	0.211472
23	6	0	3.014096	-3.196102	-1.569382
24	1	0	1.358155	-3.450458	-2.941321
25	1	0	4.574577	-2.544036	-0.215698
26	1	0	3.447727	-4.173170	-1.753689
27	6	0	1.825964	1.707263	-0.008461
28	6	0	1.363969	2.311594	1.180174
29	6	0	3.007888	2.227321	-0.575505
30	6	0	2.048618	3.372204	1.769714
31	1	0	0.460789	1.934752	1.651288
32	6	0	3.697883	3.283830	0.017203
33	1	0	3.379080	1.795523	-1.501474
34	6	0	3.221579	3.866583	1.193418
35	1	0	1.667997	3.810979	2.689250
36	1	0	4.604322	3.661986	-0.449823
37	1	0	3.753543	4.695723	1.652317
38	1	0	-0.534560	-1.249061	0.176578
39	6	0	-1.064589	-2.223043	1.895030
40	6	0	-2.505057	-2.609639	1.541821
41	1	0	-3.100698	-2.754785	2.450326
42	1	0	-2.982671	-1.826311	0.944256
43	1	0	-2.520076	-3.541657	0.966396
44	6	0	-0.370135	-3.341759	2.674618
45	1	0	-0.364946	-4.273057	2.095316
46	1	0	0.664953	-3.066465	2.913874
47	1	0	-0.890167	-3.536566	3.618036
48	6	0	-1.013177	-0.896849	2.661171
49	1	0	0.023088	-0.612978	2.874716
50	1	0	-1.478040	-0.094790	2.077981
51	1	0	-1.551860	-0.980605	3.611547
52	8	0	-0.303585	-2.087814	0.661482
53	3	0	1.375862	-2.548269	0.009329

Zero-point correction= 0.440506 (Hartree/Particle)
Thermal correction to Energy= 0.466201
Thermal correction to Enthalpy= 0.467145
Thermal correction to Gibbs Free Energy= 0.384317
Sum of electronic and zero-point Energies= -1067.321357
Sum of electronic and thermal Energies= -1067.295662
Sum of electronic and thermal Enthalpies= -1067.294718
Sum of electronic and thermal Free Energies= -1067.377546
M06-2X/6-311++G(d,p)/SMD(THF)//B3LYP/6-31G(d) energy = -1067.603564

INT2

Center Number	Atomic Number	Atomic Type	Coordinates (Angstroms) X	Y	Z

1	6	0	-4.349250	2.610543	-1.192761
2	6	0	-3.503492	3.550289	-0.590886
3	6	0	-2.151123	3.281661	-0.412655
4	6	0	-1.577837	2.053644	-0.824226
5	6	0	-2.445415	1.126646	-1.462228
6	6	0	-3.801504	1.403215	-1.637212
7	1	0	-5.405496	2.823404	-1.329889
8	1	0	-3.906720	4.502624	-0.253911
9	1	0	-1.512614	4.022737	0.063492
10	1	0	-2.022507	0.219969	-1.888669
11	1	0	-4.431331	0.677853	-2.148833
12	6	0	-0.176482	1.795618	-0.575855
13	1	0	0.431173	2.639368	-0.244449
14	7	0	0.341143	0.587865	-0.704510
15	6	0	1.605112	0.179528	-0.420591
16	6	0	1.905421	-1.211723	-0.748435
17	6	0	1.126623	-1.937150	-1.702895
18	6	0	2.931062	-1.950224	-0.090107
19	6	0	1.324302	-3.304303	-1.925789
20	1	0	0.420706	-1.392279	-2.326370
21	6	0	3.126602	-3.301044	-0.335998
22	1	0	3.561112	-1.442486	0.633243
23	6	0	2.317851	-4.003876	-1.244103
24	1	0	0.714285	-3.810545	-2.671148
25	1	0	3.916199	-3.825101	0.198095
26	1	0	2.479753	-5.061899	-1.427372
27	6	0	2.633576	1.051281	0.201638
28	6	0	2.353027	1.828409	1.343195
29	6	0	3.935929	1.125442	-0.329732
30	6	0	3.325937	2.640277	1.923435
31	1	0	1.360508	1.785343	1.783664
32	6	0	4.911315	1.934480	0.251485
33	1	0	4.177100	0.540563	-1.213381
34	6	0	4.611742	2.699758	1.380490
35	1	0	3.081354	3.223467	2.808159
36	1	0	5.906514	1.974097	-0.184950
37	1	0	5.369882	3.334404	1.831694
38	1	0	-2.542095	-0.413261	0.203187
39	6	0	-2.947787	-1.921716	1.481871
40	6	0	-4.216267	-2.351511	0.736876
41	1	0	-4.887677	-2.906498	1.401025
42	1	0	-4.761852	-1.477431	0.361825
43	1	0	-3.964479	-2.992012	-0.114778
44	6	0	-2.131754	-3.129082	1.942419
45	1	0	-1.867704	-3.767890	1.092147
46	1	0	-1.209098	-2.808512	2.439802
47	1	0	-2.707039	-3.730327	2.653654
48	6	0	-3.271361	-0.983318	2.649299
49	1	0	-2.352297	-0.656704	3.146674
50	1	0	-3.806917	-0.093486	2.298067
51	1	0	-3.904824	-1.486884	3.387732
52	8	0	-2.086124	-1.211066	0.545164
53	3	0	-0.350771	-1.142034	-0.143958

Zero-point correction= 0.439864 (Hartree/Particle)
Thermal correction to Energy= 0.466075
Thermal correction to Enthalpy= 0.467020
Thermal correction to Gibbs Free Energy= 0.380360
Sum of electronic and zero-point Energies= -1067.331850

Sum of electronic and thermal Energies= -1067.305638
Sum of electronic and thermal Enthalpies= -1067.304694
Sum of electronic and thermal Free Energies= -1067.391354
M06-2X/6-311++G(d,p)/SMD(THF)//B3LYP/6-31G(d) energy = -1067.60747997

INT3

Center Number	Atomic Number	Atomic Type	Coordinates (Angstroms) X	Y	Z
1	6	0	-3.232826	2.931674	-2.195691
2	6	0	-2.281982	3.857375	-1.753621
3	6	0	-1.039911	3.426193	-1.303678
4	6	0	-0.703751	2.051722	-1.274917
5	6	0	-1.679541	1.129732	-1.725705
6	6	0	-2.916898	1.568714	-2.182582
7	1	0	-4.205729	3.266903	-2.544390
8	1	0	-2.511685	4.920005	-1.762392
9	1	0	-0.303917	4.153659	-0.968097
10	1	0	-1.456515	0.068005	-1.726003
11	1	0	-3.647411	0.840258	-2.526198
12	6	0	0.610520	1.675940	-0.809075
13	1	0	1.275062	2.497204	-0.529757
14	7	0	1.054768	0.432463	-0.783193
15	6	0	2.260388	0.027507	-0.315901
16	6	0	2.610928	-1.364223	-0.611949
17	6	0	1.993998	-2.054329	-1.693661
18	6	0	3.517247	-2.108100	0.189558
19	6	0	2.230273	-3.411466	-1.916550
20	1	0	1.364717	-1.497025	-2.382108
21	6	0	3.758485	-3.453304	-0.055022
22	1	0	4.009555	-1.618872	1.023640
23	6	0	3.109855	-4.124425	-1.100963
24	1	0	1.741885	-3.904435	-2.753565
25	1	0	4.449651	-3.993659	0.587276
26	1	0	3.300772	-5.178099	-1.282204
27	6	0	3.160476	0.886778	0.488554
28	6	0	2.666584	1.669912	1.551822
29	6	0	4.543079	0.937492	0.223099
30	6	0	3.518515	2.464041	2.315474
31	1	0	1.606077	1.638109	1.785588
32	6	0	5.393967	1.736308	0.985230
33	1	0	4.946253	0.348759	-0.596191
34	6	0	4.887127	2.504262	2.035557
35	1	0	3.113520	3.048966	3.137511
36	1	0	6.455775	1.763218	0.753437
37	1	0	5.550361	3.126957	2.629878
38	3	0	0.211936	-1.260015	-0.189337
39	6	0	-5.019674	-0.133783	2.378723
40	6	0	-3.677393	-0.255456	2.041020
41	6	0	-3.320796	-1.124668	0.997554
42	6	0	-4.285850	-1.867152	0.297981
43	6	0	-5.622061	-1.727829	0.653699
44	6	0	-5.994854	-0.865186	1.690807
45	1	0	-5.308559	0.536971	3.182507
46	1	0	-2.906131	0.303009	2.556133
47	1	0	-3.973461	-2.529969	-0.499013
48	1	0	-6.378190	-2.296267	0.120071
49	1	0	-7.041601	-0.762933	1.961798
50	7	0	-1.954502	-1.265149	0.651643

51	8	0	-1.607637	-2.051249	-0.291288
52	8	0	-1.059055	-0.622287	1.274084

Zero-point correction= 0.405848 (Hartree/Particle)
Thermal correction to Energy= 0.432494
Thermal correction to Enthalpy= 0.433439
Thermal correction to Gibbs Free Energy= 0.344430
Sum of electronic and zero-point Energies= -1270.446949
Sum of electronic and thermal Energies= -1270.420303
Sum of electronic and thermal Enthalpies= -1270.419359
Sum of electronic and thermal Free Energies= -1270.508368
M06-2X/6-311++G(d,p)/SMD(THF)//B3LYP/6-31G(d) energy = -1270.657761

INT3a

Center Number	Atomic Number	Atomic Type	Coordinates (Angstroms) X	Y	Z
1	6	0	-1.089638	5.093179	-0.772713
2	6	0	0.275651	5.287358	-0.542128
3	6	0	1.119948	4.190463	-0.422801
4	6	0	0.621900	2.870487	-0.527865
5	6	0	-0.761242	2.689538	-0.765796
6	6	0	-1.597377	3.793815	-0.884657
7	1	0	-1.753665	5.948159	-0.866166
8	1	0	0.678479	6.292802	-0.456302
9	1	0	2.182013	4.341946	-0.243229
10	1	0	-1.194506	1.697808	-0.869030
11	1	0	-2.656813	3.637309	-1.066930
12	6	0	1.572969	1.792751	-0.388522
13	1	0	2.614027	2.093665	-0.252322
14	7	0	1.275841	0.501951	-0.442280
15	6	0	2.202863	-0.467005	-0.238880
16	6	0	1.831082	-1.819464	-0.671225
17	6	0	0.908850	-2.004934	-1.728977
18	6	0	2.342439	-2.969529	-0.028845
19	6	0	0.497069	-3.281519	-2.104515
20	1	0	0.543443	-1.140632	-2.277770
21	6	0	1.931640	-4.241156	-0.414311
22	1	0	3.041846	-2.855097	0.792706
23	6	0	1.004777	-4.405300	-1.448843
24	1	0	-0.213407	-3.395827	-2.917994
25	1	0	2.325424	-5.110670	0.104763
26	1	0	0.683128	-5.400579	-1.741553
27	6	0	3.511089	-0.225645	0.411206
28	6	0	3.605822	0.534940	1.593940
29	6	0	4.690743	-0.775936	-0.125823
30	6	0	4.836355	0.736491	2.213891
31	1	0	2.703736	0.946487	2.037705
32	6	0	5.922261	-0.561288	0.489767
33	1	0	4.635782	-1.363746	-1.037281
34	6	0	5.999795	0.193923	1.661879
35	1	0	4.886535	1.312849	3.133574
36	1	0	6.822786	-0.984052	0.052763
37	1	0	6.959519	0.355732	2.144720
38	3	0	-0.527139	-0.492296	-0.245572
39	6	0	-5.836493	-1.201830	2.061442
40	6	0	-4.477156	-1.114348	1.786961
41	6	0	-4.058493	-0.716715	0.500440
42	6	0	-5.009155	-0.410923	-0.495126

43	6	0	-6.362497	-0.506091	-0.193791
44	6	0	-6.789056	-0.900247	1.080249
45	1	0	-6.158515	-1.509264	3.053351
46	1	0	-3.727406	-1.344807	2.533295
47	1	0	-4.662635	-0.109099	-1.475464
48	1	0	-7.094946	-0.270798	-0.962143
49	1	0	-7.849463	-0.971808	1.305427
50	7	0	-2.702517	-0.625163	0.213265
51	8	0	-2.278995	-0.265882	-0.979736
52	8	0	-1.791262	-0.898755	1.114482

Zero-point correction= 0.405552 (Hartree/Particle)
Thermal correction to Energy= 0.432250
Thermal correction to Enthalpy= 0.433194
Thermal correction to Gibbs Free Energy= 0.343083
Sum of electronic and zero-point Energies= -1270.461237
Sum of electronic and thermal Energies= -1270.434540
Sum of electronic and thermal Enthalpies= -1270.433595
Sum of electronic and thermal Free Energies= -1270.523707
M06-2X/6-311++G(d,p)/SMD(THF)//B3LYP/6-31G(d) energy = -1270.679149

2a′

Center Number	Atomic Number	Atomic Type	Coordinates (Angstroms) X	Y	Z
1	6	0	-5.566939	-0.746791	0.046082
2	6	0	-5.209975	0.558969	-0.303507
3	6	0	-3.868265	0.918658	-0.379330
4	6	0	-2.850647	-0.021493	-0.108734
5	6	0	-3.226900	-1.336771	0.245418
6	6	0	-4.567953	-1.690055	0.319561
7	1	0	-6.614739	-1.028354	0.106562
8	1	0	-5.980271	1.295478	-0.516711
9	1	0	-3.591634	1.934859	-0.651863
10	1	0	-2.445128	-2.057958	0.458887
11	1	0	-4.843179	-2.705408	0.593399
12	6	0	-1.465091	0.385094	-0.194198
13	1	0	-1.276606	1.434541	-0.443866
14	7	0	-0.475235	-0.468241	0.000021
15	6	0	0.817817	-0.116327	0.009072
16	6	0	1.804997	-1.198895	0.019233
17	6	0	1.405550	-2.528691	-0.250059
18	6	0	3.167075	-0.966189	0.313183
19	6	0	2.326726	-3.568028	-0.235801
20	1	0	0.361532	-2.717546	-0.472021
21	6	0	4.085217	-2.012781	0.327729
22	1	0	3.500947	0.038756	0.548195
23	6	0	3.673797	-3.318494	0.050793
24	1	0	1.996509	-4.580857	-0.452400
25	1	0	5.126428	-1.808243	0.563405
26	1	0	4.392503	-4.133526	0.060342
27	6	0	1.266369	1.306730	0.019100
28	6	0	0.818947	2.192336	1.015811
29	6	0	2.133631	1.800273	-0.971411
30	6	0	1.229074	3.524651	1.024536
31	1	0	0.157127	1.824387	1.795025
32	6	0	2.535497	3.135752	-0.967936
33	1	0	2.485217	1.130637	-1.751257
34	6	0	2.086680	4.002001	0.031010

35	1	0	0.880788	4.190029	1.810146
36	1	0	3.199634	3.499857	-1.747451
37	1	0	2.403886	5.041334	0.036159

Zero-point correction= 0.299966 (Hartree/Particle)
Thermal correction to Energy= 0.316754
Thermal correction to Enthalpy= 0.317698
Thermal correction to Gibbs Free Energy= 0.252544
Sum of electronic and zero-point Energies= -826.190697
Sum of electronic and thermal Energies= -826.173909
Sum of electronic and thermal Enthalpies= -826.172965
Sum of electronic and thermal Free Energies= -826.238119
M06-2X/6-311++G(d,p)/SMD(THF)//B3LYP/6-31G(d) energy = -826.3574285

INT4

Center Number	Atomic Number	Atomic Type	Coordinates (Angstroms) X	Y	Z
1	3	0	-3.637372	0.000020	0.002641
2	6	0	1.999296	1.209562	0.000182
3	6	0	0.609652	1.224077	-0.000147
4	6	0	-0.088426	0.000079	-0.000294
5	6	0	0.609440	-1.224015	-0.000151
6	6	0	1.999096	-1.209698	0.000179
7	6	0	2.703893	-0.000133	0.000342
8	1	0	2.539897	2.152484	0.000321
9	1	0	0.048485	2.149946	-0.000277
10	1	0	0.048104	-2.149773	-0.000283
11	1	0	2.539532	-2.152715	0.000315
12	1	0	3.790149	-0.000221	0.000587
13	7	0	-1.477909	0.000282	-0.000828
14	8	0	-2.169228	-1.122100	-0.000212
15	8	0	-2.169071	1.121977	-0.000220

Zero-point correction= 0.103766 (Hartree/Particle)
Thermal correction to Energy= 0.111940
Thermal correction to Enthalpy= 0.112884
Thermal correction to Gibbs Free Energy= 0.070060
Sum of electronic and zero-point Energies= -444.235656
Sum of electronic and thermal Energies= -444.227482
Sum of electronic and thermal Enthalpies= -444.226538
Sum of electronic and thermal Free Energies= -444.269362
M06-2X/6-311++G(d,p)/SMD(THF)//B3LYP/6-31G(d) energy = -444.2964927

INT5

Center Number	Atomic Number	Atomic Type	Coordinates (Angstroms) X	Y	Z
1	6	0	0.524123	5.177447	0.578575
2	6	0	-0.836132	5.206313	0.266626
3	6	0	-1.511708	4.014058	0.018500
4	6	0	-0.839329	2.779790	0.070815
5	6	0	0.531367	2.759897	0.392011
6	6	0	1.200121	3.954834	0.642658
7	1	0	1.056672	6.104270	0.774750
8	1	0	-1.368134	6.152274	0.218331
9	1	0	-2.571909	4.033018	-0.224319
10	1	0	1.084965	1.826299	0.463550

11	1	0	2.257152	3.928933	0.891484
12	6	0	-1.631509	1.578725	-0.220445
13	1	0	-2.700554	1.767835	-0.366670
14	7	0	-1.172490	0.385793	-0.318972
15	6	0	-2.078355	-0.737555	-0.673914
16	6	0	-1.616163	-1.991015	0.068338
17	6	0	-1.059589	-1.923141	1.353752
18	6	0	-1.750633	-3.245570	-0.542516
19	6	0	-0.641791	-3.085185	2.010490
20	1	0	-0.947299	-0.959969	1.846156
21	6	0	-1.340406	-4.403915	0.113937
22	1	0	-2.177534	-3.311648	-1.540613
23	6	0	-0.782070	-4.326745	1.393006
24	1	0	-0.202739	-3.011943	3.001352
25	1	0	-1.445422	-5.367393	-0.377217
26	1	0	-0.452018	-5.229194	1.899669
27	6	0	-3.575429	-0.477231	-0.522234
28	6	0	-4.350775	-0.203018	-1.655952
29	6	0	-4.200225	-0.488461	0.733278
30	6	0	-5.719376	0.051445	-1.543714
31	1	0	-3.879398	-0.191619	-2.636545
32	6	0	-5.565393	-0.230690	0.848668
33	1	0	-3.617962	-0.713576	1.622331
34	6	0	-6.329740	0.038660	-0.289668
35	1	0	-6.305463	0.256364	-2.435530
36	1	0	-6.035210	-0.247270	1.828412
37	1	0	-7.394793	0.233489	-0.198258
38	1	0	-1.892903	-0.919860	-1.741698
39	3	0	0.687667	-0.479772	-0.150809
40	6	0	6.435921	-0.011847	0.739123
41	6	0	5.047742	0.003753	0.802716
42	6	0	4.302571	-0.503805	-0.281509
43	6	0	4.959435	-1.021370	-1.416911
44	6	0	6.348442	-1.025106	-1.454580
45	6	0	7.098135	-0.523159	-0.383578
46	1	0	7.009816	0.378879	1.575896
47	1	0	4.520335	0.394098	1.663910
48	1	0	4.364634	-1.406484	-2.235520
49	1	0	6.854167	-1.424603	-2.330197
50	1	0	8.183792	-0.530951	-0.423206
51	7	0	2.914357	-0.492802	-0.231919
52	8	0	2.189201	-0.956386	-1.219524
53	8	0	2.271843	-0.015516	0.812994

Zero-point correction= 0.418620 (Hartree/Particle)
Thermal correction to Energy= 0.445592
Thermal correction to Enthalpy= 0.446536
Thermal correction to Gibbs Free Energy= 0.354906
Sum of electronic and zero-point Energies= -1271.067867
Sum of electronic and thermal Energies= -1271.040894
Sum of electronic and thermal Enthalpies= -1271.039950
Sum of electronic and thermal Free Energies= -1271.131580
M06-2X/6-311++G(d,p)/SMD(THF)//B3LYP/6-31G(d) energy = -1271.306650

TS2

Center Number	Atomic Number	Atomic Type	Coordinates (Angstroms) X	Y	Z
1	6	0	-2.544963	5.140288	-0.570813

2	6	0	-2.003810	5.059609	0.713846
3	6	0	-1.101362	4.047734	1.032371
4	6	0	-0.712728	3.088125	0.077174
5	6	0	-1.269113	3.185699	-1.214415
6	6	0	-2.169842	4.196345	-1.532756
7	1	0	-3.250552	5.927466	-0.821653
8	1	0	-2.286021	5.786912	1.470783
9	1	0	-0.683444	3.991751	2.035113
10	1	0	-0.983755	2.464711	-1.975786
11	1	0	-2.583989	4.250443	-2.536538
12	6	0	0.244052	2.056950	0.470467
13	1	0	0.588420	2.095258	1.507270
14	7	0	0.682597	1.141247	-0.336859
15	6	0	1.533317	0.077735	0.010342
16	6	0	2.325907	-0.430059	-1.132102
17	6	0	2.413300	0.276040	-2.361238
18	6	0	2.951681	-1.702140	-1.083819
19	6	0	3.039901	-0.283251	-3.484292
20	1	0	2.059870	1.304888	-2.404412
21	6	0	3.590716	-2.236414	-2.193012
22	1	0	2.914574	-2.270711	-0.159172
23	6	0	3.628836	-1.540774	-3.411635
24	1	0	3.092068	0.293083	-4.405187
25	1	0	4.054333	-3.216989	-2.117378
26	1	0	4.122716	-1.972299	-4.277283
27	6	0	2.089696	-0.045216	1.397247
28	6	0	1.239746	-0.248362	2.502121
29	6	0	3.470563	0.051604	1.648217
30	6	0	1.745940	-0.346460	3.796761
31	1	0	0.169671	-0.345754	2.337081
32	6	0	3.980613	-0.058709	2.942160
33	1	0	4.149316	0.213797	0.815670
34	6	0	3.121265	-0.254715	4.024184
35	1	0	1.065454	-0.507443	4.629305
36	1	0	5.052966	0.017741	3.104864
37	1	0	3.517439	-0.338145	5.032710
38	1	0	0.411838	-1.203967	0.036135
39	3	0	0.136399	0.101341	-1.933292
40	6	0	-4.967789	-2.390348	-0.434305
41	6	0	-3.741163	-1.955119	-0.920882
42	6	0	-2.583445	-2.169598	-0.148842
43	6	0	-2.660377	-2.819148	1.098156
44	6	0	-3.900528	-3.243116	1.561560
45	6	0	-5.058241	-3.033992	0.804777
46	1	0	-5.862310	-2.226271	-1.028613
47	1	0	-3.654375	-1.458249	-1.878859
48	1	0	-1.758035	-2.980103	1.673725
49	1	0	-3.962817	-3.743937	2.523628
50	1	0	-6.021636	-3.370514	1.176278
51	7	0	-1.356476	-1.725910	-0.636534
52	8	0	-0.245742	-2.054123	0.063281
53	8	0	-1.231360	-1.128288	-1.772242

Zero-point correction= 0.413862 (Hartree/Particle)
Thermal correction to Energy= 0.440098
Thermal correction to Enthalpy= 0.441042
Thermal correction to Gibbs Free Energy= 0.353169
Sum of electronic and zero-point Energies= -1271.014719
Sum of electronic and thermal Energies= -1270.988483
Sum of electronic and thermal Enthalpies= -1270.987539

Sum of electronic and thermal Free Energies= -1271.075411
M06-2X/6-311++G(d,p)/SMD(THF)//B3LYP/6-31G(d) energy = -1271.253000

INT6

Center Number	Atomic Number	Atomic Type	Coordinates (Angstroms) X	Y	Z
1	6	0	-2.722794	5.037072	-0.520096
2	6	0	-2.187695	4.965723	0.767994
3	6	0	-1.231765	4.002355	1.079185
4	6	0	-0.780259	3.079706	0.113588
5	6	0	-1.330578	3.169607	-1.182362
6	6	0	-2.284732	4.132324	-1.493057
7	1	0	-3.470045	5.786756	-0.764943
8	1	0	-2.516829	5.663439	1.533914
9	1	0	-0.820242	3.954499	2.085042
10	1	0	-0.994303	2.483866	-1.955791
11	1	0	-2.690541	4.180429	-2.500654
12	6	0	0.225654	2.097062	0.499329
13	1	0	0.577164	2.150282	1.532552
14	7	0	0.700730	1.202402	-0.317620
15	6	0	1.604326	0.188252	0.000776
16	6	0	2.342500	-0.336406	-1.161453
17	6	0	2.363641	0.352483	-2.406523
18	6	0	2.988825	-1.600704	-1.127757
19	6	0	2.938907	-0.217716	-3.550674
20	1	0	2.004871	1.379693	-2.445274
21	6	0	3.576198	-2.144158	-2.260358
22	1	0	3.007007	-2.156947	-0.195204
23	6	0	3.544506	-1.468181	-3.490646
24	1	0	2.940092	0.346440	-4.480637
25	1	0	4.054488	-3.118360	-2.193234
26	1	0	3.999279	-1.907533	-4.373498
27	6	0	2.157354	0.021388	1.378413
28	6	0	1.307488	-0.161204	2.488516
29	6	0	3.543935	0.045733	1.621569
30	6	0	1.818142	-0.309165	3.776184
31	1	0	0.232381	-0.203635	2.333397
32	6	0	4.057036	-0.113481	2.908768
33	1	0	4.224068	0.192741	0.787458
34	6	0	3.197914	-0.288160	3.994712
35	1	0	1.136381	-0.453032	4.610854
36	1	0	5.133043	-0.090575	3.063583
37	1	0	3.596971	-0.408610	4.998278
38	1	0	0.423131	-1.217946	0.051382
39	3	0	0.126104	0.171650	-1.905005
40	6	0	-4.912116	-2.506096	-0.427378
41	6	0	-3.699542	-2.021282	-0.901696
42	6	0	-2.538361	-2.208348	-0.127636
43	6	0	-2.597087	-2.879228	1.109061
44	6	0	-3.823740	-3.352995	1.559823
45	6	0	-4.984873	-3.171908	0.801110
46	1	0	-5.809350	-2.363730	-1.023053
47	1	0	-3.626424	-1.506434	-1.851328
48	1	0	-1.693102	-3.017149	1.687985
49	1	0	-3.872491	-3.870473	2.513734
50	1	0	-5.937503	-3.547038	1.163125
51	7	0	-1.328901	-1.708080	-0.602123
52	8	0	-0.203448	-2.044408	0.087556

53	8	0	-1.211110	-1.096582	-1.727658

Zero-point correction= 0.415015 (Hartree/Particle)
Thermal correction to Energy= 0.442027
Thermal correction to Enthalpy= 0.442971
Thermal correction to Gibbs Free Energy= 0.352873
Sum of electronic and zero-point Energies= -1271.013609
Sum of electronic and thermal Energies= -1270.986597
Sum of electronic and thermal Enthalpies= -1270.985653
Sum of electronic and thermal Free Energies= -1271.075752
M06-2X/6-311++G(d,p)/SMD(THF)//B3LYP/6-31G(d) energy = -1271.253181

INT6′

Center Number	Atomic Number	Atomic Type	Coordinates (Angstroms) X	Y	Z
1	6	0	-1.400321	5.001848	-0.937021
2	6	0	-0.072781	5.271982	-0.592218
3	6	0	0.824773	4.225398	-0.418206
4	6	0	0.418399	2.880886	-0.580318
5	6	0	-0.927105	2.623746	-0.931007
6	6	0	-1.817491	3.676516	-1.105390
7	1	0	-2.104918	5.817339	-1.075183
8	1	0	0.259882	6.297990	-0.460844
9	1	0	1.857834	4.436412	-0.151218
10	1	0	-1.280268	1.608078	-1.073830
11	1	0	-2.847475	3.462596	-1.377263
12	6	0	1.415196	1.853537	-0.388248
13	1	0	2.425569	2.206489	-0.169708
14	7	0	1.188216	0.552523	-0.493063
15	6	0	2.152007	-0.369187	-0.254447
16	6	0	1.888986	-1.732874	-0.721485
17	6	0	0.980573	-1.966626	-1.781548
18	6	0	2.501304	-2.853137	-0.114158
19	6	0	0.682593	-3.262022	-2.194780
20	1	0	0.526786	-1.122460	-2.292265
21	6	0	2.204277	-4.143766	-0.538501
22	1	0	3.191012	-2.702785	0.709531
23	6	0	1.292141	-4.357004	-1.577143
24	1	0	-0.021662	-3.413684	-3.007611
25	1	0	2.677167	-4.990436	-0.048165
26	1	0	1.060228	-5.367259	-1.902277
27	6	0	3.404427	-0.058780	0.477558
28	6	0	3.365487	0.621182	1.710523
29	6	0	4.655040	-0.455960	-0.030248
30	6	0	4.539285	0.892538	2.409643
31	1	0	2.405482	0.913966	2.126656
32	6	0	5.828939	-0.171038	0.665365
33	1	0	4.701312	-0.981040	-0.979962
34	6	0	5.775245	0.501971	1.887788
35	1	0	4.488493	1.404511	3.366694
36	1	0	6.786663	-0.475203	0.251763
37	1	0	6.690219	0.718000	2.432514
38	1	0	-1.345405	-1.863274	1.686163
39	3	0	-0.582188	-0.532473	-0.177308
40	6	0	-5.834699	0.331638	0.683678
41	6	0	-4.583274	0.137926	0.105882
42	6	0	-3.915648	-1.075306	0.302404
43	6	0	-4.502191	-2.093283	1.061079

44	6	0	-5.754164	-1.894865	1.637102
45	6	0	-6.422004	-0.680988	1.449990
46	1	0	-6.359155	1.272638	0.537364
47	1	0	-4.108264	0.900186	-0.500977
48	1	0	-3.963572	-3.028571	1.184336
49	1	0	-6.212150	-2.683621	2.227898
50	1	0	-7.400409	-0.524895	1.896977
51	7	0	-2.658066	-1.395034	-0.321562
52	8	0	-1.440028	-0.963328	1.337919
53	8	0	-2.258198	-0.513668	-1.150927

Zero-point correction= 0.413768 (Hartree/Particle)
Thermal correction to Energy= 0.441906
Thermal correction to Enthalpy= 0.442851
Thermal correction to Gibbs Free Energy= 0.349346
Sum of electronic and zero-point Energies= -1271.037554
Sum of electronic and thermal Energies= -1271.009416
Sum of electronic and thermal Enthalpies= -1271.008472
Sum of electronic and thermal Free Energies= -1271.101977
M06-2X/6-311++G(d,p)/SMD(THF)//B3LYP/6-31G(d) energy = -1271.263410

INT7

Center Number	Atomic Number	Atomic Type	Coordinates (Angstroms) X	Y	Z
1	6	0	0.999371	5.488934	0.706683
2	6	0	-0.252923	5.492299	0.084447
3	6	0	-0.966324	4.306385	-0.036524
4	6	0	-0.448720	3.086278	0.460182
5	6	0	0.820321	3.094378	1.090151
6	6	0	1.523149	4.290292	1.203335
7	1	0	1.562287	6.413762	0.803298
8	1	0	-0.669394	6.417822	-0.304222
9	1	0	-1.940515	4.308433	-0.520982
10	1	0	1.273587	2.184001	1.496210
11	1	0	2.496458	4.283232	1.687047
12	6	0	-1.269888	1.909515	0.285542
13	1	0	-2.232877	2.077296	-0.202798
14	7	0	-0.956589	0.686978	0.689881
15	6	0	-1.751263	-0.378175	0.431433
16	6	0	-1.490279	-1.601349	1.193480
17	6	0	-0.859195	-1.541836	2.458527
18	6	0	-1.834617	-2.874399	0.684869
19	6	0	-0.568185	-2.704085	3.168002
20	1	0	-0.625948	-0.573710	2.892107
21	6	0	-1.545370	-4.030409	1.401930
22	1	0	-2.307914	-2.948394	-0.288459
23	6	0	-0.908632	-3.954032	2.645116
24	1	0	-0.084181	-2.630657	4.137889
25	1	0	-1.808280	-4.998933	0.984809
26	1	0	-0.683316	-4.859813	3.201120
27	6	0	-2.830119	-0.357534	-0.588280
28	6	0	-2.567485	0.071044	-1.903300
29	6	0	-4.131496	-0.784726	-0.267980
30	6	0	-3.577467	0.075847	-2.862791
31	1	0	-1.558006	0.368823	-2.169590
32	6	0	-5.142854	-0.767688	-1.227501
33	1	0	-4.347273	-1.121090	0.742181
34	6	0	-4.869379	-0.337927	-2.527627

35	1	0	-3.353902	0.396537	-3.876776
36	1	0	-6.145291	-1.090323	-0.959005
37	1	0	-5.656599	-0.329933	-3.276680
38	1	0	2.334014	0.598640	3.081386
39	3	0	0.953566	-0.059463	1.155737
40	6	0	4.792881	-1.830651	-2.490599
41	6	0	3.439935	-1.613163	-2.289826
42	6	0	3.014619	-1.075424	-1.059611
43	6	0	3.919797	-0.750917	-0.034345
44	6	0	5.279292	-0.974686	-0.262079
45	6	0	5.711812	-1.510720	-1.475534
46	1	0	5.146299	-2.247526	-3.429587
47	1	0	2.700021	-1.845640	-3.048010
48	1	0	3.531938	-0.320897	0.904041
49	1	0	6.001401	-0.729012	0.511420
50	1	0	6.772101	-1.684235	-1.642387
51	7	0	1.646566	-0.822250	-0.772037
52	8	0	2.279742	0.442841	2.130048
53	8	0	0.843129	-1.056165	-1.672714

Zero-point correction= 0.413208 (Hartree/Particle)
Thermal correction to Energy= 0.441807
Thermal correction to Enthalpy= 0.442751
Thermal correction to Gibbs Free Energy= 0.349207
Sum of electronic and zero-point Energies= -1271.049725
Sum of electronic and thermal Energies= -1271.021127
Sum of electronic and thermal Enthalpies= -1271.020183
Sum of electronic and thermal Free Energies= -1271.113727
M06-2X/6-311++G(d,p)/SMD(THF)//B3LYP/6-31G(d) energy = -1271.284344

INT8

Center Number	Atomic Number	Atomic Type	Coordinates (Angstroms) X	Y	Z
1	6	0	4.488598	-3.311775	0.869424
2	6	0	3.811135	-3.727843	-0.280856
3	6	0	2.519857	-3.276186	-0.532954
4	6	0	1.872993	-2.393723	0.359923
5	6	0	2.569096	-1.986066	1.521372
6	6	0	3.858816	-2.440046	1.767193
7	1	0	5.496733	-3.665555	1.067641
8	1	0	4.292810	-4.405489	-0.980927
9	1	0	1.995204	-3.599976	-1.429112
10	1	0	2.069128	-1.319848	2.216589
11	1	0	4.379987	-2.120347	2.666215
12	6	0	0.532521	-1.938226	0.064066
13	1	0	0.063691	-2.337882	-0.840261
14	7	0	-0.091623	-1.062296	0.836393
15	6	0	-1.346033	-0.654303	0.609385
16	6	0	-2.211358	-1.268161	-0.444943
17	6	0	-2.644267	-0.519962	-1.553003
18	6	0	-2.594988	-2.616352	-0.346897
19	6	0	-3.443246	-1.111347	-2.532818
20	1	0	-2.341887	0.518153	-1.653957
21	6	0	-3.399757	-3.201308	-1.324338
22	1	0	-2.267721	-3.202058	0.508323
23	6	0	-3.825836	-2.449494	-2.421145
24	1	0	-3.765256	-0.522440	-3.387756
25	1	0	-3.695275	-4.242840	-1.227635

26	1	0	-4.451450	-2.904538	-3.184621
27	6	0	-1.871418	0.420889	1.451083
28	6	0	-3.232078	0.797942	1.407869
29	6	0	-1.024983	1.106453	2.353496
30	6	0	-3.718590	1.817104	2.221940
31	1	0	-3.909552	0.281519	0.736714
32	6	0	-1.516346	2.121978	3.162967
33	1	0	0.019322	0.820591	2.398297
34	6	0	-2.866462	2.486964	3.102622
35	1	0	-4.770288	2.086840	2.170855
36	1	0	-0.845090	2.636675	3.845951
37	1	0	-3.248267	3.282963	3.736345
38	6	0	2.094332	1.787960	-0.487673
39	6	0	3.362253	2.304438	-0.225216
40	6	0	3.726866	3.544250	-0.755107
41	6	0	2.829741	4.276488	-1.548036
42	6	0	1.565017	3.769772	-1.814268
43	6	0	1.204985	2.522919	-1.280312
44	1	0	1.770382	0.830251	-0.091637
45	1	0	4.062201	1.742873	0.386397
46	1	0	4.715551	3.947656	-0.552445
47	1	0	3.127854	5.239655	-1.953001
48	1	0	0.843473	4.304547	-2.423047
49	7	0	-0.070013	1.901781	-1.497935
50	8	0	-0.856898	2.547331	-2.180099

Zero-point correction= 0.398130 (Hartree/Particle)
Thermal correction to Energy= 0.423528
Thermal correction to Enthalpy= 0.424472
Thermal correction to Gibbs Free Energy= 0.334773
Sum of electronic and zero-point Energies= -1187.636602
Sum of electronic and thermal Energies= -1187.611204
Sum of electronic and thermal Enthalpies= -1187.610259
Sum of electronic and thermal Free Energies= -1187.699958
M06-2X/6-311++G(d,p)/SMD(THF)//B3LYP/6-31G(d) energy = -1187.855298

TS3a

Center Number	Atomic Number	Atomic Type	Coordinates (Angstroms) X	Y	Z
1	6	0	4.303832	2.614738	-1.966769
2	6	0	3.944684	3.012521	-0.676964
3	6	0	2.728856	2.599360	-0.136716
4	6	0	1.857203	1.781818	-0.876828
5	6	0	2.229572	1.386568	-2.174710
6	6	0	3.442269	1.802538	-2.713003
7	1	0	5.251736	2.935563	-2.390637
8	1	0	4.610290	3.644676	-0.095533
9	1	0	2.443985	2.909314	0.866305
10	1	0	1.550361	0.757248	-2.739778
11	1	0	3.722431	1.493642	-3.716561
12	6	0	0.585436	1.372624	-0.281764
13	1	0	0.374589	1.771383	0.711521
14	7	0	-0.303980	0.697926	-0.967149
15	6	0	-1.417396	0.258132	-0.323188
16	6	0	-2.076999	1.096136	0.741451
17	6	0	-2.505302	0.567580	1.968100
18	6	0	-2.329390	2.450164	0.465279
19	6	0	-3.161477	1.376076	2.896056

20	1	0	-2.298137	-0.471818	2.193958
21	6	0	-2.990137	3.255581	1.393032
22	1	0	-2.012460	2.868811	-0.485662
23	6	0	-3.407205	2.721142	2.612778
24	1	0	-3.476923	0.953283	3.846324
25	1	0	-3.178857	4.300254	1.160232
26	1	0	-3.919242	3.348093	3.337913
27	6	0	-2.255993	-0.707840	-1.079452
28	6	0	-3.615553	-0.905827	-0.787573
29	6	0	-1.672303	-1.465693	-2.112132
30	6	0	-4.369810	-1.831216	-1.509088
31	1	0	-4.089817	-0.326590	-0.003117
32	6	0	-2.425955	-2.388457	-2.828457
33	1	0	-0.622793	-1.312804	-2.334670
34	6	0	-3.779630	-2.575868	-2.530195
35	1	0	-5.422160	-1.965838	-1.273479
36	1	0	-1.958474	-2.966804	-3.621148
37	1	0	-4.368259	-3.296927	-3.091401
38	6	0	2.987380	-1.131291	1.610211
39	6	0	4.043699	-2.017070	1.786660
40	6	0	3.846624	-3.392792	1.619844
41	6	0	2.579024	-3.876148	1.272569
42	6	0	1.512049	-3.003326	1.096229
43	6	0	1.710158	-1.616044	1.263738
44	1	0	3.123548	-0.060810	1.727994
45	1	0	5.026771	-1.636863	2.051646
46	1	0	4.674289	-4.083067	1.757685
47	1	0	2.425402	-4.944242	1.139632
48	1	0	0.525152	-3.360338	0.824391
49	7	0	0.692361	-0.669524	1.126456
50	8	0	-0.460240	-1.131929	0.830574

Zero-point correction= 0.399656 (Hartree/Particle)
Thermal correction to Energy= 0.423186
Thermal correction to Enthalpy= 0.424130
Thermal correction to Gibbs Free Energy= 0.342389
Sum of electronic and zero-point Energies= -1187.625807
Sum of electronic and thermal Energies= -1187.602277
Sum of electronic and thermal Enthalpies= -1187.601333
Sum of electronic and thermal Free Energies= -1187.683074
M06-2X/6-311++G(d,p)/SMD(THF)//B3LYP/6-31G(d) energy = -1187.845401

TS3b

Center Number	Atomic Number	Atomic Type	Coordinates (Angstroms) X	Y	Z
1	6	0	4.017452	-0.757791	2.981319
2	6	0	3.610293	-1.942080	2.359485
3	6	0	2.436481	-1.971688	1.613533
4	6	0	1.644966	-0.812567	1.472267
5	6	0	2.069513	0.377209	2.100015
6	6	0	3.242069	0.399241	2.846762
7	1	0	4.932039	-0.736363	3.567989
8	1	0	4.208671	-2.843679	2.460549
9	1	0	2.121570	-2.889588	1.124942
10	1	0	1.463191	1.269409	1.990806
11	1	0	3.556742	1.321553	3.328328
12	6	0	0.427613	-0.882980	0.679863
13	1	0	0.112947	-1.878820	0.370078

14	7	0	-0.448741	0.150114	0.706802
15	6	0	-1.702087	0.051092	0.324132
16	6	0	-2.349898	-1.211715	-0.148978
17	6	0	-1.840818	-1.933156	-1.241913
18	6	0	-3.485766	-1.705559	0.517087
19	6	0	-2.455151	-3.115851	-1.655446
20	1	0	-0.967842	-1.560901	-1.767561
21	6	0	-4.088210	-2.894353	0.109657
22	1	0	-3.888740	-1.156348	1.363445
23	6	0	-3.575922	-3.601699	-0.980664
24	1	0	-2.051402	-3.658672	-2.505691
25	1	0	-4.958833	-3.267456	0.642494
26	1	0	-4.048560	-4.526127	-1.301705
27	6	0	-2.523775	1.278105	0.409333
28	6	0	-3.743601	1.398608	-0.285229
29	6	0	-2.080505	2.377821	1.173569
30	6	0	-4.490423	2.573360	-0.217449
31	1	0	-4.097752	0.573802	-0.894045
32	6	0	-2.831695	3.545008	1.244190
33	1	0	-1.144343	2.287268	1.712404
34	6	0	-4.041172	3.649986	0.548715
35	1	0	-5.423975	2.648090	-0.768703
36	1	0	-2.477640	4.377212	1.847132
37	1	0	-4.626900	4.563631	0.605161
38	6	0	1.732337	1.542151	-1.460669
39	6	0	2.543321	2.642136	-1.728629
40	6	0	3.864777	2.455309	-2.145067
41	6	0	4.378759	1.161645	-2.291885
42	6	0	3.578451	0.055717	-2.027520
43	6	0	2.251458	0.249044	-1.614621
44	1	0	0.708112	1.664830	-1.125398
45	1	0	2.145586	3.646679	-1.613466
46	1	0	4.494686	3.315820	-2.354219
47	1	0	5.407439	1.019865	-2.612819
48	1	0	3.946012	-0.958605	-2.135435
49	7	0	1.369904	-0.850367	-1.370708
50	8	0	1.826256	-1.983974	-1.597261

Zero-point correction= 0.399248 (Hartree/Particle)
Thermal correction to Energy= 0.423136
Thermal correction to Enthalpy= 0.424080
Thermal correction to Gibbs Free Energy= 0.340939
Sum of electronic and zero-point Energies= -1187.627486
Sum of electronic and thermal Energies= -1187.603598
Sum of electronic and thermal Enthalpies= -1187.602654
Sum of electronic and thermal Free Energies= -1187.685795
M06-2X/6-311++G(d,p)/SMD(THF)//B3LYP/6-31G(d) energy = -1187.848326

INT9a

Center Number	Atomic Number	Atomic Type	Coordinates (Angstroms) X	Y	Z
1	6	0	5.861091	-0.052147	0.803224
2	6	0	5.456392	0.182579	-0.511714
3	6	0	4.108123	0.076906	-0.851601
4	6	0	3.153118	-0.267004	0.116682
5	6	0	3.569892	-0.498230	1.438713
6	6	0	4.914451	-0.391512	1.777025
7	1	0	6.910853	0.031357	1.072076

8	1	0	6.188370	0.448244	-1.269497
9	1	0	3.787424	0.260666	-1.874645
10	1	0	2.821437	-0.752162	2.182130
11	1	0	5.230507	-0.568970	2.801571
12	6	0	1.740102	-0.374260	-0.279015
13	1	0	1.521716	-0.104522	-1.318230
14	7	0	0.834825	-0.764890	0.535306
15	6	0	-0.555912	-0.750356	0.102087
16	6	0	-0.788096	-1.174215	-1.358368
17	6	0	-1.572205	-0.429446	-2.245440
18	6	0	-0.245228	-2.391416	-1.797717
19	6	0	-1.797769	-0.886286	-3.547058
20	1	0	-2.004964	0.511052	-1.926757
21	6	0	-0.469369	-2.845442	-3.095468
22	1	0	0.353306	-2.987357	-1.114522
23	6	0	-1.247494	-2.092006	-3.978053
24	1	0	-2.406773	-0.291811	-4.223236
25	1	0	-0.038707	-3.790402	-3.416235
26	1	0	-1.424150	-2.444848	-4.990628
27	6	0	-1.400907	-1.625434	1.038810
28	6	0	-2.794758	-1.648709	0.890297
29	6	0	-0.810844	-2.405261	2.036352
30	6	0	-3.583036	-2.431228	1.730990
31	1	0	-3.263036	-1.054043	0.111780
32	6	0	-1.601352	-3.199870	2.870697
33	1	0	0.265465	-2.377617	2.155900
34	6	0	-2.987598	-3.213551	2.724127
35	1	0	-4.662896	-2.435259	1.607146
36	1	0	-1.128550	-3.804881	3.640218
37	1	0	-3.601741	-3.829554	3.375933
38	6	0	-0.282983	3.909798	-0.601448
39	6	0	-0.724890	5.201217	-0.358221
40	6	0	-1.800863	5.427104	0.511235
41	6	0	-2.429669	4.342198	1.136959
42	6	0	-2.002947	3.041637	0.905670
43	6	0	-0.915199	2.804787	0.025021
44	1	0	0.549583	3.712316	-1.269698
45	1	0	-0.232898	6.039831	-0.843499
46	1	0	-2.145002	6.439790	0.701445
47	1	0	-3.261365	4.518718	1.814222
48	1	0	-2.481825	2.201056	1.392744
49	7	0	-0.394247	1.577770	-0.292573
50	8	0	-1.101635	0.591141	0.348743

Zero-point correction= 0.400943 (Hartree/Particle)
Thermal correction to Energy= 0.424713
Thermal correction to Enthalpy= 0.425657
Thermal correction to Gibbs Free Energy= 0.343248
Sum of electronic and zero-point Energies= -1187.645027
Sum of electronic and thermal Energies= -1187.621257
Sum of electronic and thermal Enthalpies= -1187.620313
Sum of electronic and thermal Free Energies= -1187.702722
M06-2X/6-311++G(d,p)/SMD(THF)//B3LYP/6-31G(d) energy = -1187.878400

INT9b

Center Number	Atomic Number	Atomic Type	Coordinates (Angstroms) X	Y	Z
1	6	0	3.104665	-2.119265	3.280084

2	6	0	3.428352	-2.498221	1.974722
3	6	0	2.651236	-2.059389	0.904520
4	6	0	1.543831	-1.229633	1.125397
5	6	0	1.219700	-0.858619	2.433285
6	6	0	1.997983	-1.301333	3.505671
7	1	0	3.711029	-2.461756	4.114393
8	1	0	4.287703	-3.137168	1.789417
9	1	0	2.902739	-2.358619	-0.109602
10	1	0	0.359530	-0.221213	2.609846
11	1	0	1.736871	-1.003892	4.518036
12	6	0	0.669374	-0.825566	-0.061447
13	1	0	0.163476	-1.721464	-0.431868
14	7	0	-0.236581	0.255572	0.264342
15	6	0	-1.504334	0.166728	0.057292
16	6	0	-2.217288	-1.021669	-0.525945
17	6	0	-1.928508	-1.490975	-1.816782
18	6	0	-3.198604	-1.679775	0.233882
19	6	0	-2.608273	-2.596365	-2.331667
20	1	0	-1.161644	-1.005728	-2.411660
21	6	0	-3.864994	-2.791448	-0.278669
22	1	0	-3.434306	-1.319496	1.231546
23	6	0	-3.574401	-3.249547	-1.565930
24	1	0	-2.374284	-2.948194	-3.332530
25	1	0	-4.613484	-3.297035	0.325497
26	1	0	-4.098398	-4.112233	-1.968518
27	6	0	-2.339782	1.344207	0.448272
28	6	0	-3.639932	1.517753	-0.053447
29	6	0	-1.814321	2.325076	1.308151
30	6	0	-4.393504	2.639016	0.295751
31	1	0	-4.059793	0.780888	-0.729573
32	6	0	-2.569921	3.437808	1.661939
33	1	0	-0.806153	2.197728	1.686056
34	6	0	-3.863935	3.599601	1.156856
35	1	0	-5.394700	2.760486	-0.108748
36	1	0	-2.150964	4.182562	2.333478
37	1	0	-4.453055	4.470087	1.433181
38	6	0	2.531139	1.545909	-0.210092
39	6	0	3.446578	2.591583	-0.300217
40	6	0	4.222612	2.769694	-1.447733
41	6	0	4.073073	1.884316	-2.519255
42	6	0	3.167265	0.831987	-2.448121
43	6	0	2.392048	0.654824	-1.288193
44	1	0	1.918402	1.432564	0.673249
45	1	0	3.548396	3.276517	0.537347
46	1	0	4.932725	3.589630	-1.507837
47	1	0	4.668700	2.012499	-3.419303
48	1	0	3.040914	0.136239	-3.267802
49	7	0	1.491921	-0.435931	-1.252262
50	8	0	1.363571	-1.188760	-2.284890

Zero-point correction= 0.403150 (Hartree/Particle)
Thermal correction to Energy= 0.426740
Thermal correction to Enthalpy= 0.427684
Thermal correction to Gibbs Free Energy= 0.345635
Sum of electronic and zero-point Energies= -1187.656829
Sum of electronic and thermal Energies= -1187.633239
Sum of electronic and thermal Enthalpies= -1187.632295
Sum of electronic and thermal Free Energies= -1187.714344
M06-2X/6-311++G(d,p)/SMD(THF)//B3LYP/6-31G(d) energy = -1187.897118

INT10

Center Number	Atomic Number	Atomic Type	Coordinates (Angstroms) X	Y	Z
1	6	0	-3.650276	3.924941	-1.146070
2	6	0	-3.255579	3.049852	-2.161412
3	6	0	-2.483971	1.928157	-1.861127
4	6	0	-2.105766	1.670945	-0.537973
5	6	0	-2.500103	2.549982	0.476352
6	6	0	-3.270385	3.674154	0.172995
7	1	0	-4.252553	4.797783	-1.382861
8	1	0	-3.550075	3.241001	-3.189714
9	1	0	-2.177198	1.246135	-2.648873
10	1	0	-2.197624	2.359253	1.503724
11	1	0	-3.574526	4.350451	0.967317
12	6	0	-1.219193	0.475196	-0.201631
13	1	0	-1.262240	0.270614	0.872284
14	7	0	0.123231	0.746678	-0.680259
15	6	0	1.076459	1.035294	0.145108
16	6	0	0.974534	0.883249	1.634322
17	6	0	0.821356	1.989895	2.479905
18	6	0	1.048278	-0.411537	2.172427
19	6	0	0.714175	1.801368	3.858839
20	1	0	0.783022	2.992150	2.060739
21	6	0	0.951393	-0.587107	3.554441
22	1	0	1.211209	-1.248873	1.492435
23	6	0	0.778337	0.513560	4.396747
24	1	0	0.588490	2.660329	4.512682
25	1	0	1.015471	-1.588302	3.972023
26	1	0	0.700977	0.370371	5.471303
27	6	0	2.359053	1.517543	-0.426197
28	6	0	3.549113	1.461395	0.317036
29	6	0	2.392256	2.046584	-1.729427
30	6	0	4.748031	1.904350	-0.238277
31	1	0	3.538105	1.048659	1.319647
32	6	0	3.590033	2.493873	-2.277920
33	1	0	1.466296	2.112298	-2.291091
34	6	0	4.772210	2.420928	-1.534737
35	1	0	5.664646	1.841711	0.341301
36	1	0	3.602800	2.906928	-3.282874
37	1	0	5.707325	2.770289	-1.964290
38	6	0	-3.628625	-1.011213	0.646818
39	6	0	-4.704593	-1.774717	1.092545
40	6	0	-4.994596	-3.009530	0.509096
41	6	0	-4.193779	-3.481885	-0.535907
42	6	0	-3.115219	-2.736800	-0.995429
43	6	0	-2.824047	-1.493824	-0.402272
44	1	0	-3.441481	-0.044148	1.095386
45	1	0	-5.323487	-1.393841	1.899990
46	1	0	-5.835576	-3.597898	0.864179
47	1	0	-4.407706	-4.443371	-0.994072
48	1	0	-2.480959	-3.095028	-1.795746
49	7	0	-1.714498	-0.769973	-0.880114
50	8	0	-1.097308	-1.192830	-1.930234
51	8	0	1.863416	-2.049288	-0.680060
52	3	0	0.826696	-1.086889	-1.612045
53	6	0	2.774898	-3.081031	-0.549448
54	6	0	4.011083	-2.597357	0.248791
55	1	0	3.707251	-2.267663	1.250357

56	1	0	4.773356	-3.380103	0.364853
57	1	0	4.468486	-1.741231	-0.261272
58	6	0	2.124613	-4.269135	0.199756
59	1	0	1.787853	-3.946918	1.193179
60	1	0	1.245043	-4.618538	-0.354432
61	1	0	2.811919	-5.116382	0.330033
62	6	0	3.243862	-3.567720	-1.942063
63	1	0	2.380224	-3.918202	-2.522027
64	1	0	3.705951	-2.735773	-2.488890
65	1	0	3.973596	-4.387492	-1.885908

Zero-point correction= 0.530561 (Hartree/Particle)
Thermal correction to Energy= 0.563336
Thermal correction to Enthalpy= 0.564280
Thermal correction to Gibbs Free Energy= 0.460760
Sum of electronic and zero-point Energies= -1428.209654
Sum of electronic and thermal Energies= -1428.176880
Sum of electronic and thermal Enthalpies= -1428.175935
Sum of electronic and thermal Free Energies= -1428.279456
M06-2X/6-311++G(d,p)/SMD(THF)//B3LYP/6-31G(d) energy = -1428.531747

TS4

Center Number	Atomic Number	Atomic Type	Coordinates (Angstroms) X	Y	Z
1	6	0	-1.072261	4.225656	-0.672333
2	6	0	-0.979212	3.403284	-1.797172
3	6	0	-0.831585	2.024993	-1.645034
4	6	0	-0.780566	1.443929	-0.368401
5	6	0	-0.873393	2.280349	0.751176
6	6	0	-1.016701	3.660292	0.602651
7	1	0	-1.186839	5.300171	-0.789191
8	1	0	-1.023869	3.835204	-2.793765
9	1	0	-0.763244	1.386866	-2.522436
10	1	0	-0.828798	1.843458	1.744298
11	1	0	-1.083778	4.293176	1.483695
12	6	0	-0.588781	-0.047598	-0.226490
13	1	0	-0.548277	-0.700251	1.013217
14	7	0	0.646610	-0.574486	-0.691647
15	6	0	1.845698	-0.130405	-0.503346
16	6	0	2.237348	1.060579	0.319114
17	6	0	2.859361	2.175929	-0.257873
18	6	0	2.052823	1.020102	1.709577
19	6	0	3.264237	3.247536	0.538009
20	1	0	3.017830	2.205964	-1.332444
21	6	0	2.478848	2.084551	2.504721
22	1	0	1.570480	0.152308	2.152571
23	6	0	3.079429	3.202190	1.921513
24	1	0	3.730619	4.114850	0.078140
25	1	0	2.340510	2.040739	3.582117
26	1	0	3.405797	4.032734	2.542047
27	6	0	2.947260	-0.946868	-1.095181
28	6	0	4.266514	-0.860077	-0.617304
29	6	0	2.675104	-1.847098	-2.145209
30	6	0	5.274416	-1.659879	-1.156901
31	1	0	4.500874	-0.173982	0.189573
32	6	0	3.683672	-2.642807	-2.681850
33	1	0	1.666119	-1.893123	-2.543756
34	6	0	4.989135	-2.554731	-2.188621

35	1	0	6.285569	-1.583667	-0.765717
36	1	0	3.454627	-3.323217	-3.498117
37	1	0	5.776859	-3.172581	-2.611378
38	6	0	-3.652975	0.494277	-0.276730
39	6	0	-5.008086	0.757132	-0.474068
40	6	0	-5.750712	0.036155	-1.409233
41	6	0	-5.121901	-0.970883	-2.147624
42	6	0	-3.773785	-1.250782	-1.959582
43	6	0	-3.018898	-0.509428	-1.029557
44	1	0	-3.105542	1.058474	0.463993
45	1	0	-5.484277	1.531515	0.121351
46	1	0	-6.805496	0.250040	-1.556752
47	1	0	-5.686269	-1.545337	-2.877519
48	1	0	-3.282347	-2.034339	-2.521941
49	7	0	-1.653210	-0.859331	-0.879853
50	8	0	-1.372288	-2.119297	-1.111275
51	8	0	-0.178598	-1.684441	1.717442
52	3	0	0.108770	-2.441748	0.017832
53	6	0	-1.018013	-2.152479	2.769384
54	6	0	-0.120433	-2.461366	3.979802
55	1	0	0.395966	-1.553359	4.310607
56	1	0	-0.699363	-2.857120	4.823719
57	1	0	0.640160	-3.201913	3.706916
58	6	0	-2.049082	-1.073460	3.144574
59	1	0	-1.540876	-0.148453	3.442258
60	1	0	-2.707202	-0.850009	2.297161
61	1	0	-2.677639	-1.398817	3.982003
62	6	0	-1.743062	-3.432238	2.311907
63	1	0	-2.348826	-3.231889	1.420625
64	1	0	-1.010764	-4.213465	2.064447
65	1	0	-2.401422	-3.833232	3.092433

Zero-point correction= 0.525362 (Hartree/Particle)
Thermal correction to Energy= 0.557287
Thermal correction to Enthalpy= 0.558231
Thermal correction to Gibbs Free Energy= 0.458471
Sum of electronic and zero-point Energies= -1428.182825
Sum of electronic and thermal Energies= -1428.150901
Sum of electronic and thermal Enthalpies= -1428.149956
Sum of electronic and thermal Free Energies= -1428.249716
M06-2X/6-311++G(d,p)/SMD(THF)//B3LYP/6-31G(d) energy = -1428.50639996

INT11

Center Number	Atomic Number	Atomic Type	Coordinates (Angstroms) X	Y	Z
1	6	0	-3.670295	3.114393	-1.119471
2	6	0	-3.363099	2.140249	-2.070845
3	6	0	-2.530162	1.074318	-1.734420
4	6	0	-1.990302	0.965478	-0.443015
5	6	0	-2.301839	1.949361	0.504364
6	6	0	-3.138905	3.013926	0.166704
7	1	0	-4.320065	3.946021	-1.379088
8	1	0	-3.772455	2.207691	-3.075279
9	1	0	-2.297500	0.313071	-2.474053
10	1	0	-1.894273	1.884436	1.506760
11	1	0	-3.373055	3.767166	0.914272
12	6	0	-1.057694	-0.183678	-0.184294
13	1	0	1.682299	-3.915061	0.532790

14	7	0	0.273929	-0.093755	-0.279051
15	6	0	1.012013	1.075449	-0.170741
16	6	0	0.900389	1.916736	1.023493
17	6	0	1.128031	3.312247	0.992820
18	6	0	0.551440	1.343341	2.270494
19	6	0	1.014003	4.086829	2.143645
20	1	0	1.360775	3.789111	0.045892
21	6	0	0.445283	2.121092	3.419523
22	1	0	0.371942	0.273051	2.322208
23	6	0	0.674661	3.500044	3.366667
24	1	0	1.178567	5.159976	2.083471
25	1	0	0.188477	1.648710	4.364889
26	1	0	0.587990	4.107412	4.263451
27	6	0	2.062755	1.258349	-1.161687
28	6	0	3.241997	2.009437	-0.915281
29	6	0	1.962098	0.619455	-2.426064
30	6	0	4.241808	2.119924	-1.874968
31	1	0	3.379971	2.484269	0.050215
32	6	0	2.967868	0.733782	-3.380053
33	1	0	1.062754	0.055394	-2.652581
34	6	0	4.116850	1.486092	-3.116515
35	1	0	5.135423	2.696750	-1.648142
36	1	0	2.849692	0.241417	-4.342606
37	1	0	4.900504	1.577282	-3.863612
38	6	0	-3.849438	-1.148162	0.792495
39	6	0	-5.182140	-1.558946	0.822740
40	6	0	-5.615155	-2.622680	0.030372
41	6	0	-4.698359	-3.282532	-0.792725
42	6	0	-3.367114	-2.878046	-0.834321
43	6	0	-2.942320	-1.791148	-0.057877
44	1	0	-3.515582	-0.339669	1.431248
45	1	0	-5.879080	-1.051845	1.484783
46	1	0	-6.652898	-2.943108	0.062874
47	1	0	-5.021221	-4.120244	-1.405615
48	1	0	-2.639289	-3.391569	-1.450948
49	7	0	-1.561225	-1.435395	-0.108489
50	8	0	-0.713226	-2.489508	-0.169359
51	8	0	2.257054	-3.251117	0.113782
52	3	0	0.978439	-1.912012	-0.392673
53	6	0	3.491812	-3.138425	0.888971
54	6	0	4.316221	-2.070305	0.171544
55	1	0	3.793698	-1.107673	0.156858
56	1	0	5.274193	-1.927125	0.681972
57	1	0	4.517883	-2.368774	-0.862259
58	6	0	3.138636	-2.713141	2.318407
59	1	0	2.610892	-1.753541	2.318523
60	1	0	2.495740	-3.459195	2.802507
61	1	0	4.043164	-2.607829	2.927435
62	6	0	4.196305	-4.498586	0.863579
63	1	0	3.578792	-5.270798	1.339703
64	1	0	4.398640	-4.806088	-0.167238
65	1	0	5.147084	-4.453276	1.405763

Zero-point correction= 0.530315 (Hartree/Particle)
Thermal correction to Energy= 0.563148
Thermal correction to Enthalpy= 0.564092
Thermal correction to Gibbs Free Energy= 0.460166
Sum of electronic and zero-point Energies= -1428.229257
Sum of electronic and thermal Energies= -1428.196424
Sum of electronic and thermal Enthalpies= -1428.195480

Sum of electronic and thermal Free Energies= -1428.299406
M06-2X/6-311++G(d,p)/SMD(THF)//B3LYP/6-31G(d) energy = -1428.559285

INT11′

Center Number	Atomic Number	Atomic Type	Coordinates (Angstroms) X	Y	Z
1	6	0	-0.876988	-4.379742	1.059087
2	6	0	-0.156362	-4.230755	-0.132360
3	6	0	0.362491	-2.992947	-0.501843
4	6	0	0.179344	-1.853130	0.311240
5	6	0	-0.538947	-2.022179	1.516995
6	6	0	-1.060723	-3.266983	1.884556
7	1	0	-1.272162	-5.350176	1.346203
8	1	0	-0.006707	-5.087221	-0.784907
9	1	0	0.902891	-2.894567	-1.437549
10	1	0	-0.646829	-1.170505	2.184080
11	1	0	-1.586062	-3.370346	2.831634
12	6	0	0.627382	-0.508955	-0.086354
13	1	0	-2.729727	-1.773465	0.824238
14	7	0	-0.267110	0.498847	0.086130
15	6	0	0.032599	1.828299	0.091538
16	6	0	-1.097608	2.702743	-0.204103
17	6	0	-2.108196	2.283440	-1.117756
18	6	0	-1.294216	3.958313	0.429446
19	6	0	-3.259741	3.047409	-1.337267
20	1	0	-1.910372	1.415227	-1.746624
21	6	0	-2.438958	4.708949	0.202287
22	1	0	-0.541647	4.319216	1.123058
23	6	0	-3.442478	4.258348	-0.671617
24	1	0	-3.994719	2.709615	-2.065042
25	1	0	-2.563466	5.656889	0.720397
26	1	0	-4.332730	4.856127	-0.843825
27	6	0	1.332297	2.388928	0.489145
28	6	0	1.830026	3.573424	-0.091852
29	6	0	2.103237	1.783251	1.502476
30	6	0	3.039071	4.125236	0.321805
31	1	0	1.268290	4.044214	-0.892911
32	6	0	3.312874	2.335133	1.914051
33	1	0	1.735539	0.880042	1.980505
34	6	0	3.789980	3.509903	1.326827
35	1	0	3.404293	5.033130	-0.152125
36	1	0	3.884137	1.849812	2.701785
37	1	0	4.736487	3.938153	1.646047
38	6	0	3.759307	-1.289204	-1.847258
39	6	0	4.904906	-2.076958	-1.785381
40	6	0	5.239965	-2.753885	-0.608869
41	6	0	4.418451	-2.624532	0.513173
42	6	0	3.268048	-1.838921	0.464274
43	6	0	2.924812	-1.182739	-0.725894
44	1	0	3.491449	-0.735228	-2.738635
45	1	0	5.543697	-2.159090	-2.661135
46	1	0	6.137994	-3.363875	-0.562839
47	1	0	4.680760	-3.125199	1.441697
48	1	0	2.650575	-1.722762	1.347495
49	7	0	1.769676	-0.343215	-0.839516
50	8	0	1.874482	0.660338	-1.660511
51	8	0	-3.346376	-1.061003	0.556997
52	3	0	-2.198662	0.397401	0.216536

53	6	0	-4.520826	-1.659790	-0.078173
54	6	0	-4.067616	-2.389477	-1.347297
55	1	0	-3.596358	-1.692475	-2.048696
56	1	0	-3.341774	-3.175204	-1.107056
57	1	0	-4.920478	-2.858328	-1.850409
58	6	0	-5.161410	-2.624675	0.924936
59	1	0	-5.434316	-2.097697	1.844909
60	1	0	-6.065354	-3.076964	0.503062
61	1	0	-4.469270	-3.435589	1.181274
62	6	0	-5.451304	-0.491219	-0.397682
63	1	0	-6.365666	-0.854930	-0.877566
64	1	0	-5.731196	0.042631	0.516597
65	1	0	-4.970613	0.218555	-1.080233

Zero-point correction= 0.529216 (Hartree/Particle)
Thermal correction to Energy= 0.561961
Thermal correction to Enthalpy= 0.562905
Thermal correction to Gibbs Free Energy= 0.459694
Sum of electronic and zero-point Energies= -1428.193807
Sum of electronic and thermal Energies= -1428.161062
Sum of electronic and thermal Enthalpies= -1428.160118
Sum of electronic and thermal Free Energies= -1428.263329
M06-2X/6-311++G(d,p)/SMD(THF)//B3LYP/6-31G(d) energy = -1428.530248

TS5

Center Number	Atomic Number	Atomic Type	Coordinates (Angstroms) X	Y	Z
1	6	0	-2.296706	-4.108327	1.116034
2	6	0	-1.580041	-4.101309	-0.090857
3	6	0	-0.758233	-3.034317	-0.429396
4	6	0	-0.615916	-1.920857	0.438513
5	6	0	-1.340109	-1.944299	1.661922
6	6	0	-2.164071	-3.022289	1.988421
7	1	0	-2.932451	-4.950022	1.375116
8	1	0	-1.673039	-4.938443	-0.778476
9	1	0	-0.228594	-3.036348	-1.376008
10	1	0	-1.203720	-1.126142	2.363667
11	1	0	-2.689071	-3.022787	2.941245
12	6	0	0.145233	-0.747964	0.075664
13	1	0	-3.086482	-0.737898	0.884674
14	7	0	-0.227044	0.483065	0.508078
15	6	0	0.714718	1.468063	0.188721
16	6	0	0.095949	2.724947	-0.297558
17	6	0	-0.889979	2.642997	-1.311867
18	6	0	0.358298	3.988211	0.265897
19	6	0	-1.598858	3.777539	-1.719960
20	1	0	-0.999245	1.696828	-1.838213
21	6	0	-0.355598	5.113768	-0.141495
22	1	0	1.111292	4.077887	1.041998
23	6	0	-1.343910	5.016199	-1.128819
24	1	0	-2.330449	3.695805	-2.520730
25	1	0	-0.146590	6.074776	0.321519
26	1	0	-1.894841	5.898581	-1.442222
27	6	0	2.011603	1.521466	0.907665
28	6	0	3.081922	2.291130	0.410594
29	6	0	2.208231	0.800235	2.097221
30	6	0	4.294227	2.349435	1.090859
31	1	0	2.960928	2.818108	-0.530955

32	6	0	3.426341	0.853933	2.774018
33	1	0	1.390087	0.206198	2.491448
34	6	0	4.473722	1.631512	2.277847
35	1	0	5.109489	2.944442	0.686653
36	1	0	3.555537	0.288711	3.693762
37	1	0	5.423316	1.673802	2.804836
38	6	0	3.044992	-1.410057	-2.188839
39	6	0	4.123181	-2.265229	-2.396875
40	6	0	4.384491	-3.315636	-1.513186
41	6	0	3.555946	-3.487074	-0.402704
42	6	0	2.479768	-2.631735	-0.172816
43	6	0	2.206163	-1.590934	-1.075974
44	1	0	2.836563	-0.588554	-2.862938
45	1	0	4.764709	-2.108558	-3.260787
46	1	0	5.225611	-3.982557	-1.681278
47	1	0	3.756802	-4.284242	0.309044
48	1	0	1.869620	-2.758814	0.713288
49	7	0	1.094361	-0.724455	-0.938387
50	8	0	1.246742	0.534585	-1.456185
51	8	0	-3.488155	0.102240	0.573411
52	3	0	-1.991746	1.197341	0.272231
53	6	0	-4.763209	-0.187646	-0.080481
54	6	0	-4.497558	-1.083656	-1.295311
55	1	0	-3.839979	-0.580438	-2.012543
56	1	0	-4.017719	-2.021249	-0.992001
57	1	0	-5.435023	-1.331624	-1.805199
58	6	0	-5.673753	-0.880566	0.938618
59	1	0	-5.815426	-0.247529	1.820584
60	1	0	-6.655440	-1.090321	0.500232
61	1	0	-5.239293	-1.833963	1.261229
62	6	0	-5.323147	1.172759	-0.493654
63	1	0	-6.289285	1.050810	-0.993663
64	1	0	-5.468138	1.815181	0.381645
65	1	0	-4.646024	1.679663	-1.191475

Zero-point correction= 0.528091 (Hartree/Particle)
Thermal correction to Energy= 0.560275
Thermal correction to Enthalpy= 0.561220
Thermal correction to Gibbs Free Energy= 0.460658
Sum of electronic and zero-point Energies= -1428.179307
Sum of electronic and thermal Energies= -1428.147123
Sum of electronic and thermal Enthalpies= -1428.146179
Sum of electronic and thermal Free Energies= -1428.246740
M06-2X/6-311++G(d,p)/SMD(THF)//B3LYP/6-31G(d) energy = -1428.50900957

TS4a

Center Number	Atomic Number	Atomic Type	Coordinates (Angstroms) X	Y	Z
1	6	0	4.518323	-2.149644	1.750805
2	6	0	3.993195	-2.934570	0.722374
3	6	0	2.740438	-2.632337	0.188072
4	6	0	2.002174	-1.546196	0.676275
5	6	0	2.537970	-0.760471	1.705874
6	6	0	3.787651	-1.063627	2.241486
7	1	0	5.493337	-2.383776	2.170166
8	1	0	4.555323	-3.782734	0.340859
9	1	0	2.325778	-3.244875	-0.609733
10	1	0	1.957425	0.076906	2.079200

11	1	0	4.194947	-0.452369	3.042460
12	6	0	0.670182	-1.237625	0.089133
13	1	0	0.294096	-2.037628	-0.558995
14	7	0	-0.222660	-0.557626	0.845174
15	6	0	-1.267827	-0.071095	-0.016595
16	6	0	-2.251338	-1.186980	-0.403960
17	6	0	-2.363307	-1.650596	-1.718257
18	6	0	-3.032413	-1.777455	0.599981
19	6	0	-3.247272	-2.689974	-2.024115
20	1	0	-1.766177	-1.190247	-2.497392
21	6	0	-3.909734	-2.814685	0.292454
22	1	0	-2.948347	-1.420860	1.622420
23	6	0	-4.020685	-3.275044	-1.022795
24	1	0	-3.329722	-3.037759	-3.050746
25	1	0	-4.508305	-3.264630	1.080222
26	1	0	-4.707472	-4.082454	-1.262707
27	6	0	-1.985528	1.132548	0.588144
28	6	0	-2.918980	1.838694	-0.184706
29	6	0	-1.729336	1.544795	1.899157
30	6	0	-3.577974	2.945493	0.344786
31	1	0	-3.126754	1.517253	-1.201396
32	6	0	-2.399895	2.649638	2.431357
33	1	0	-1.006643	0.993501	2.489339
34	6	0	-3.321560	3.353620	1.657578
35	1	0	-4.296913	3.487268	-0.264248
36	1	0	-2.196922	2.959335	3.453412
37	1	0	-3.841119	4.213501	2.072369
38	6	0	2.987442	0.724870	-1.550047
39	6	0	3.942960	1.731532	-1.609665
40	6	0	3.575780	3.066414	-1.407531
41	6	0	2.240334	3.385299	-1.137258
42	6	0	1.274273	2.388675	-1.058519
43	6	0	1.641076	1.042512	-1.265274
44	1	0	3.257805	-0.310755	-1.725069
45	1	0	4.977056	1.477270	-1.825302
46	1	0	4.323708	3.852417	-1.463956
47	1	0	1.951835	4.421217	-0.979728
48	1	0	0.239856	2.625881	-0.844553
49	7	0	0.738238	-0.024760	-1.266647
50	8	0	-0.595943	0.447187	-1.231104

Zero-point correction= 0.399839 (Hartree/Particle)
Thermal correction to Energy= 0.422774
Thermal correction to Enthalpy= 0.423718
Thermal correction to Gibbs Free Energy= 0.343469
Sum of electronic and zero-point Energies= -1187.603319
Sum of electronic and thermal Energies= -1187.580383
Sum of electronic and thermal Enthalpies= -1187.579439
Sum of electronic and thermal Free Energies= -1187.659689
M06-2X/6-311++G(d,p)/SMD(THF)//B3LYP/6-31G(d) energy = -1187.835064

TS4b

Center Number	Atomic Number	Atomic Type	Coordinates (Angstroms) X	Y	Z
1	6	0	4.445182	-2.163909	1.793297
2	6	0	3.913332	-2.938152	0.760819
3	6	0	2.694137	-2.582879	0.182283
4	6	0	2.001543	-1.448218	0.620334

5	6	0	2.535191	-0.683276	1.663244
6	6	0	3.751227	-1.038985	2.244915
7	1	0	5.392007	-2.440192	2.249704
8	1	0	4.440809	-3.821836	0.411914
9	1	0	2.275731	-3.190940	-0.617022
10	1	0	1.986708	0.181931	2.019954
11	1	0	4.157162	-0.438228	3.054389
12	6	0	0.682830	-1.089542	-0.022322
13	1	0	0.222173	-2.010078	-0.419677
14	7	0	-0.284435	-0.495948	0.899515
15	6	0	-1.375469	-0.083351	0.192596
16	6	0	-2.317969	-1.186220	-0.280475
17	6	0	-2.815093	-1.260132	-1.587703
18	6	0	-2.728147	-2.139422	0.662296
19	6	0	-3.712420	-2.269695	-1.939878
20	1	0	-2.483436	-0.540767	-2.327039
21	6	0	-3.620393	-3.151213	0.304022
22	1	0	-2.341656	-2.083425	1.675339
23	6	0	-4.117423	-3.217781	-0.997987
24	1	0	-4.091087	-2.317144	-2.957677
25	1	0	-3.924405	-3.886821	1.044093
26	1	0	-4.812638	-4.004503	-1.278901
27	6	0	-2.025051	1.180471	0.677400
28	6	0	-3.334198	1.526163	0.309253
29	6	0	-1.302415	2.062248	1.499422
30	6	0	-3.901459	2.723692	0.745470
31	1	0	-3.913925	0.855447	-0.315548
32	6	0	-1.875078	3.249739	1.945604
33	1	0	-0.289325	1.799588	1.784898
34	6	0	-3.177177	3.588218	1.566647
35	1	0	-4.915492	2.976376	0.447137
36	1	0	-1.303526	3.915399	2.587186
37	1	0	-3.622033	4.518781	1.909144
38	6	0	3.054467	0.528833	-1.540625
39	6	0	4.016361	1.528807	-1.660077
40	6	0	3.661510	2.874081	-1.533207
41	6	0	2.328612	3.210756	-1.290757
42	6	0	1.356288	2.219892	-1.163992
43	6	0	1.721738	0.870318	-1.267722
44	1	0	3.329352	-0.512279	-1.663470
45	1	0	5.047001	1.253184	-1.866895
46	1	0	4.414125	3.651032	-1.634699
47	1	0	2.037744	4.254212	-1.200467
48	1	0	0.317849	2.473976	-0.992772
49	7	0	0.730012	-0.160080	-1.188721
50	8	0	-0.525368	0.261976	-1.397787

Zero-point correction= 0.399761 (Hartree/Particle)
Thermal correction to Energy= 0.422703
Thermal correction to Enthalpy= 0.423647
Thermal correction to Gibbs Free Energy= 0.343431
Sum of electronic and zero-point Energies= -1187.594257
Sum of electronic and thermal Energies= -1187.571315
Sum of electronic and thermal Enthalpies= -1187.570371
Sum of electronic and thermal Free Energies= -1187.650587
M06-2X/6-311++G(d,p)/SMD(THF)//B3LYP/6-31G(d) energy = -1187.828529

INT10′

Center Atomic Atomic Coordinates (Angstroms)

Number	Number	Type	X	Y	Z
1	6	0	4.428070	-2.197982	1.783580
2	6	0	3.949947	-3.014985	0.758359
3	6	0	2.738537	-2.712389	0.134663
4	6	0	2.000427	-1.589630	0.523256
5	6	0	2.483350	-0.777090	1.555539
6	6	0	3.690860	-1.080106	2.181817
7	1	0	5.368729	-2.433406	2.274283
8	1	0	4.513876	-3.890604	0.448409
9	1	0	2.361763	-3.354023	-0.658831
10	1	0	1.908809	0.090252	1.865611
11	1	0	4.057179	-0.443334	2.982486
12	6	0	0.687856	-1.285137	-0.162856
13	1	0	0.297044	-2.219159	-0.601135
14	7	0	-0.335445	-0.802191	0.740549
15	6	0	-1.279700	-0.110869	-0.091941
16	6	0	-2.459884	-1.079097	-0.329492
17	6	0	-2.674203	-1.663104	-1.581930
18	6	0	-3.305179	-1.407153	0.739255
19	6	0	-3.735296	-2.553514	-1.764915
20	1	0	-2.019779	-1.407403	-2.406901
21	6	0	-4.357005	-2.301497	0.553769
22	1	0	-3.138196	-0.959096	1.713957
23	6	0	-4.576651	-2.876772	-0.700641
24	1	0	-3.901660	-2.993201	-2.744912
25	1	0	-5.006176	-2.549393	1.389309
26	1	0	-5.399583	-3.571632	-0.845638
27	6	0	-1.746450	1.208539	0.523755
28	6	0	-2.481447	2.103365	-0.266521
29	6	0	-1.474142	1.533170	1.856241
30	6	0	-2.925611	3.310895	0.267821
31	1	0	-2.696260	1.852255	-1.301149
32	6	0	-1.925425	2.743465	2.390400
33	1	0	-0.910970	0.837133	2.468524
34	6	0	-2.649066	3.634730	1.599599
35	1	0	-3.491070	3.999363	-0.354563
36	1	0	-1.706720	2.987047	3.426820
37	1	0	-2.998500	4.575872	2.015688
38	6	0	2.991493	0.515754	-1.600367
39	6	0	3.937150	1.537016	-1.632341
40	6	0	3.562742	2.856993	-1.369779
41	6	0	2.229190	3.139309	-1.075068
42	6	0	1.276561	2.120255	-1.018820
43	6	0	1.657143	0.795465	-1.264462
44	1	0	3.277059	-0.503166	-1.838077
45	1	0	4.967709	1.300536	-1.884165
46	1	0	4.298540	3.655418	-1.410035
47	1	0	1.918392	4.163319	-0.883635
48	1	0	0.242351	2.354050	-0.804240
49	7	0	0.735961	-0.303454	-1.295538
50	8	0	-0.625719	0.143862	-1.377552

Zero-point correction= 0.402153 (Hartree/Particle)
Thermal correction to Energy= 0.424991
Thermal correction to Enthalpy= 0.425936
Thermal correction to Gibbs Free Energy= 0.346562
Sum of electronic and zero-point Energies= -1187.615523
Sum of electronic and thermal Energies= -1187.592684
Sum of electronic and thermal Enthalpies= -1187.591740

Sum of electronic and thermal Free Energies= -1187.671113
M06-2X/6-311++G(d,p)/SMD(THF)//B3LYP/6-31G(d) energy = -1187.862158

INT11″

Center Number	Atomic Number	Atomic Type	Coordinates (Angstroms) X	Y	Z
1	6	0	-2.040505	4.429310	-1.812071
2	6	0	-2.841058	3.453063	-2.410124
3	6	0	-2.495594	2.107302	-2.306658
4	6	0	-1.352980	1.721889	-1.591721
5	6	0	-0.551957	2.700288	-0.991928
6	6	0	-0.898391	4.049325	-1.108341
7	1	0	-2.305965	5.479571	-1.897066
8	1	0	-3.732721	3.739140	-2.961299
9	1	0	-3.115995	1.347480	-2.774221
10	1	0	0.339769	2.437782	-0.426450
11	1	0	-0.266667	4.799962	-0.641602
12	6	0	-1.003421	0.245591	-1.560774
13	1	0	-0.802402	-0.066228	-2.604313
14	7	0	0.197720	-0.133719	-0.839823
15	6	0	0.017214	-1.529011	-0.482549
16	6	0	1.053774	-2.317987	-1.310187
17	6	0	0.698692	-2.895387	-2.535648
18	6	0	2.386239	-2.389778	-0.876081
19	6	0	1.662985	-3.544916	-3.308353
20	1	0	-0.331920	-2.850130	-2.867113
21	6	0	3.348032	-3.028580	-1.658890
22	1	0	2.669851	-1.969113	0.084715
23	6	0	2.989054	-3.608629	-2.877468
24	1	0	1.373925	-4.001604	-4.251106
25	1	0	4.375550	-3.078727	-1.309736
26	1	0	3.737491	-4.110366	-3.484517
27	6	0	0.152622	-1.768821	1.027868
28	6	0	-0.463170	-2.905045	1.568800
29	6	0	0.898856	-0.925663	1.860702
30	6	0	-0.334898	-3.194664	2.925317
31	1	0	-1.052304	-3.550442	0.925658
32	6	0	1.023123	-1.222975	3.221184
33	1	0	1.385094	-0.030878	1.477585
34	6	0	0.409363	-2.353875	3.756946
35	1	0	-0.819783	-4.077327	3.333728
36	1	0	1.601080	-0.558113	3.857352
37	1	0	0.506899	-2.579886	4.815406
38	6	0	-4.063747	-1.024416	0.175488
39	6	0	-4.879278	-0.834323	1.288741
40	6	0	-4.472102	0.023235	2.313259
41	6	0	-3.243051	0.677418	2.220842
42	6	0	-2.413020	0.473263	1.117715
43	6	0	-2.824171	-0.379896	0.086996
44	1	0	-4.369756	-1.672074	-0.640237
45	1	0	-5.837019	-1.343947	1.349796
46	1	0	-5.111064	0.184189	3.177259
47	1	0	-2.919354	1.345711	3.013866
48	1	0	-1.456706	0.979754	1.068673
49	7	0	-2.068336	-0.667272	-1.110921
50	8	0	-1.310263	-1.895348	-0.954395
51	6	0	3.579400	2.676317	1.012967
52	6	0	3.472756	4.105516	0.433118

53	1	0	4.099991	4.827049	0.973310
54	1	0	3.778237	4.102348	-0.620018
55	1	0	2.432904	4.452282	0.481909
56	6	0	3.124525	2.695188	2.491498
57	1	0	2.074526	3.005743	2.554350
58	1	0	3.206880	1.688486	2.920263
59	1	0	3.724170	3.378984	3.106696
60	6	0	5.051217	2.208563	0.942135
61	1	0	5.137385	1.191278	1.343039
62	1	0	5.384300	2.189420	-0.102677
63	1	0	5.728890	2.861198	1.508713
64	8	0	2.777334	1.813403	0.287465
65	3	0	1.993140	0.762382	-0.699340

Zero-point correction= 0.529913 (Hartree/Particle)
Thermal correction to Energy= 0.562119
Thermal correction to Enthalpy= 0.563063
Thermal correction to Gibbs Free Energy= 0.461124
Sum of electronic and zero-point Energies= -1428.158156
Sum of electronic and thermal Energies= -1428.125950
Sum of electronic and thermal Enthalpies= -1428.125006
Sum of electronic and thermal Free Energies= -1428.226944
M06-2X/6-311++G(d,p)/SMD(THF)//B3LYP/6-31G(d) energy = -1428.487494

TS5′

Center Number	Atomic Number	Atomic Type	Coordinates (Angstroms) X	Y	Z
1	6	0	0.736132	-4.759547	-1.496730
2	6	0	0.706511	-4.593281	-0.110652
3	6	0	0.552146	-3.321908	0.440121
4	6	0	0.411924	-2.203704	-0.392006
5	6	0	0.448407	-2.372655	-1.779412
6	6	0	0.609842	-3.646173	-2.328074
7	1	0	0.861639	-5.750086	-1.925436
8	1	0	0.810069	-5.453656	0.544834
9	1	0	0.537159	-3.192916	1.518445
10	1	0	0.354639	-1.507337	-2.427631
11	1	0	0.637382	-3.765648	-3.407904
12	6	0	0.298254	-0.832709	0.244652
13	1	0	1.303995	-0.631357	0.695556
14	7	0	0.128139	0.290702	-0.643745
15	6	0	-0.565162	1.304314	0.131694
16	6	0	0.420571	2.485045	0.288341
17	6	0	1.088094	2.715394	1.497572
18	6	0	0.724384	3.286232	-0.827024
19	6	0	2.031981	3.740770	1.592240
20	1	0	0.866527	2.089091	2.352593
21	6	0	1.682494	4.297483	-0.730220
22	1	0	0.190302	3.136837	-1.761513
23	6	0	2.337733	4.528086	0.482403
24	1	0	2.539837	3.911665	2.537082
25	1	0	1.905200	4.911493	-1.598553
26	1	0	3.081556	5.315922	0.558724
27	6	0	-1.880009	1.734973	-0.530546
28	6	0	-2.880958	2.301765	0.267412
29	6	0	-2.088072	1.611107	-1.909155
30	6	0	-4.071335	2.741991	-0.307988
31	1	0	-2.728011	2.378693	1.338821

32	6	0	-3.283364	2.052134	-2.482418
33	1	0	-1.323503	1.155965	-2.531034
34	6	0	-4.276120	2.620234	-1.684780
35	1	0	-4.843031	3.177179	0.321253
36	1	0	-3.436856	1.945728	-3.553063
37	1	0	-5.206017	2.962529	-2.130700
38	6	0	-2.699146	-1.513916	2.376053
39	6	0	-3.988936	-2.040697	2.358607
40	6	0	-4.601996	-2.343761	1.140942
41	6	0	-3.921243	-2.107360	-0.054205
42	6	0	-2.636946	-1.561810	-0.041344
43	6	0	-2.018135	-1.264468	1.178727
44	1	0	-2.197168	-1.288067	3.311734
45	1	0	-4.509216	-2.226982	3.294267
46	1	0	-5.602908	-2.766535	1.123368
47	1	0	-4.391708	-2.342217	-1.005119
48	1	0	-2.124745	-1.376139	-0.977614
49	7	0	-0.693973	-0.703670	1.329853
50	8	0	-0.788094	0.741882	1.450579
51	6	0	4.504425	-0.537579	0.125005
52	6	0	4.333339	-1.467460	1.349558
53	1	0	5.269752	-1.966118	1.633119
54	1	0	3.978182	-0.886629	2.209631
55	1	0	3.586583	-2.239547	1.128197
56	6	0	4.981025	-1.378175	-1.082385
57	1	0	4.224502	-2.131441	-1.331286
58	1	0	5.113786	-0.729109	-1.957279
59	1	0	5.932314	-1.892072	-0.888613
60	6	0	5.569485	0.534856	0.449652
61	1	0	5.697605	1.203062	-0.411411
62	1	0	5.239860	1.141540	1.301725
63	1	0	6.547553	0.098575	0.694062
64	8	0	3.294550	0.067639	-0.172087
65	3	0	2.147314	1.025700	-0.894391

Zero-point correction= 0.528744 (Hartree/Particle)
Thermal correction to Energy= 0.560219
Thermal correction to Enthalpy= 0.561164
Thermal correction to Gibbs Free Energy= 0.461899
Sum of electronic and zero-point Energies= -1428.150496
Sum of electronic and thermal Energies= -1428.119021
Sum of electronic and thermal Enthalpies= -1428.118077
Sum of electronic and thermal Free Energies= -1428.217342
M06-2X/6-311++G(d,p)/SMD(THF)//B3LYP/6-31G(d) energy = -1428.481158

INT12

Center Number	Atomic Number	Atomic Type	Coordinates (Angstroms) X	Y	Z
1	6	0	-2.555077	4.210036	-0.956749
2	6	0	-1.778318	4.201987	0.220245
3	6	0	-0.896084	3.172531	0.496397
4	6	0	-0.753970	2.064649	-0.397311
5	6	0	-1.531307	2.099800	-1.604699
6	6	0	-2.402072	3.155844	-1.866408
7	1	0	-3.235676	5.029696	-1.165983
8	1	0	-1.873636	5.019951	0.931088
9	1	0	-0.318758	3.182436	1.415469
10	1	0	-1.356874	1.338251	-2.361697

11	1	0	-2.954823	3.165327	-2.804152
12	6	0	0.076508	0.951818	-0.111572
13	1	0	-3.074545	0.736924	-1.013278
14	7	0	-0.035734	-0.271596	-0.689597
15	6	0	0.841682	-1.165376	0.044523
16	6	0	-0.002359	-2.392080	0.424536
17	6	0	-0.569942	-2.536898	1.699911
18	6	0	-0.335984	-3.326762	-0.574846
19	6	0	-1.444371	-3.593072	1.969314
20	1	0	-0.312843	-1.819253	2.470435
21	6	0	-1.227509	-4.370189	-0.305570
22	1	0	0.129765	-3.248091	-1.554122
23	6	0	-1.783938	-4.508568	0.968763
24	1	0	-1.860744	-3.700880	2.967638
25	1	0	-1.469496	-5.085198	-1.087726
26	1	0	-2.465064	-5.327500	1.182516
27	6	0	2.133453	-1.549281	-0.687712
28	6	0	3.066771	-2.381874	-0.053586
29	6	0	2.405778	-1.079475	-1.974356
30	6	0	4.251480	-2.734396	-0.695178
31	1	0	2.864458	-2.746701	0.950407
32	6	0	3.593280	-1.434785	-2.620324
33	1	0	1.682302	-0.430697	-2.457376
34	6	0	4.517676	-2.262313	-1.984262
35	1	0	4.969424	-3.376278	-0.190840
36	1	0	3.795132	-1.059713	-3.620535
37	1	0	5.441683	-2.538242	-2.485850
38	6	0	3.225170	1.285623	1.893117
39	6	0	4.419836	1.997607	1.940538
40	6	0	4.646884	3.071417	1.075130
41	6	0	3.660816	3.415521	0.151260
42	6	0	2.463589	2.701911	0.080287
43	6	0	2.238321	1.627947	0.954044
44	1	0	3.049411	0.453792	2.564864
45	1	0	5.178528	1.712197	2.665168
46	1	0	5.577978	3.629324	1.121639
47	1	0	3.821888	4.242783	-0.535600
48	1	0	1.711395	2.973265	-0.650498
49	7	0	0.991062	0.936955	0.982265
50	8	0	1.162835	-0.463908	1.285279
51	6	0	-4.539539	-0.078214	0.099959
52	6	0	-4.081732	0.571504	1.410977
53	1	0	-4.922184	0.664075	2.108018
54	1	0	-3.303298	-0.031318	1.891604
55	1	0	-3.675063	1.572968	1.231480
56	6	0	-5.618868	0.761040	-0.591531
57	1	0	-5.256427	1.778313	-0.779638
58	1	0	-5.902157	0.310381	-1.548415
59	1	0	-6.513393	0.832776	0.036805
60	6	0	-5.004245	-1.518043	0.316349
61	1	0	-5.291552	-1.979291	-0.634791
62	1	0	-4.211095	-2.122498	0.772622
63	1	0	-5.870862	-1.542248	0.985241
64	8	0	-3.392753	-0.173560	-0.803268
65	3	0	-1.765662	-1.113259	-0.745582

Zero-point correction= 0.529976 (Hartree/Particle)
Thermal correction to Energy= 0.562176
Thermal correction to Enthalpy= 0.563120
Thermal correction to Gibbs Free Energy= 0.462596

Sum of electronic and zero-point Energies= -1428.196022
Sum of electronic and thermal Energies= -1428.163822
Sum of electronic and thermal Enthalpies= -1428.162878
Sum of electronic and thermal Free Energies= -1428.263401
M06-2X/6-311++G(d,p)/SMD(THF)//B3LYP/6-31G(d) energy = -1428.535817

INT13

Center Number	Atomic Number	Atomic Type	Coordinates (Angstroms) X	Y	Z
1	6	0	0.517962	4.663339	-1.500466
2	6	0	-0.714474	4.320164	-2.061669
3	6	0	-1.265429	3.064217	-1.821393
4	6	0	-0.581951	2.139135	-1.015232
5	6	0	0.657530	2.486302	-0.450650
6	6	0	1.200410	3.745757	-0.698806
7	1	0	0.946723	5.643555	-1.690488
8	1	0	-1.244241	5.028776	-2.691898
9	1	0	-2.215016	2.789266	-2.268366
10	1	0	1.198468	1.781300	0.177688
11	1	0	2.159869	4.007415	-0.262400
12	6	0	-1.145492	0.798826	-0.776873
13	7	0	-0.451936	-0.268679	-0.546452
14	6	0	-1.401431	-1.362614	-0.284040
15	6	0	-1.022150	-2.584472	-1.125199
16	6	0	-1.552875	-2.766979	-2.407290
17	6	0	-0.062740	-3.487204	-0.646160
18	6	0	-1.133447	-3.839944	-3.194820
19	1	0	-2.297218	-2.071947	-2.778450
20	6	0	0.366361	-4.549915	-1.443051
21	1	0	0.341444	-3.369686	0.355532
22	6	0	-0.170234	-4.730942	-2.718529
23	1	0	-1.558758	-3.976112	-4.185622
24	1	0	1.114414	-5.238830	-1.060818
25	1	0	0.159019	-5.561856	-3.336407
26	6	0	-1.509886	-1.671995	1.213636
27	6	0	-2.604637	-2.424701	1.661754
28	6	0	-0.555734	-1.232863	2.138008
29	6	0	-2.744936	-2.729438	3.013264
30	1	0	-3.350499	-2.760988	0.947373
31	6	0	-0.694787	-1.549119	3.493280
32	1	0	0.299441	-0.650036	1.810205
33	6	0	-1.787376	-2.293753	3.934138
34	1	0	-3.601306	-3.308129	3.349297
35	1	0	0.056554	-1.207739	4.200198
36	1	0	-1.895045	-2.535434	4.988227
37	6	0	-4.786074	1.388785	-0.663119
38	6	0	-5.781681	2.071183	0.032860
39	6	0	-5.489238	2.681785	1.255114
40	6	0	-4.201772	2.594778	1.787888
41	6	0	-3.205653	1.893074	1.108977
42	6	0	-3.499264	1.300207	-0.123279
43	1	0	-4.991069	0.918687	-1.619840
44	1	0	-6.782805	2.135419	-0.383844
45	1	0	-6.263604	3.222833	1.791618
46	1	0	-3.972538	3.061658	2.741526
47	1	0	-2.211294	1.800264	1.534391
48	7	0	-2.513315	0.578629	-0.875236
49	8	0	-2.676842	-0.854435	-0.777116

50	3	0	1.528693	-0.598195	-0.642840
51	6	0	7.258343	0.433490	-0.265419
52	6	0	5.960575	0.097752	-0.631324
53	6	0	4.921607	0.235905	0.312050
54	6	0	5.197373	0.709369	1.611311
55	6	0	6.504287	1.038016	1.951139
56	6	0	7.543240	0.904784	1.022109
57	1	0	8.059032	0.326129	-0.992971
58	1	0	5.723366	-0.269489	-1.621802
59	1	0	4.382836	0.805527	2.318073
60	1	0	6.716497	1.401856	2.953501
61	1	0	8.561572	1.163928	1.297950
62	7	0	3.619295	-0.094558	-0.041796
63	8	0	2.625334	0.026846	0.811547
64	8	0	3.326658	-0.535800	-1.240558

Zero-point correction= 0.497299 (Hartree/Particle)
Thermal correction to Energy= 0.530175
Thermal correction to Enthalpy= 0.531119
Thermal correction to Gibbs Free Energy= 0.424508
Sum of electronic and zero-point Energies= -1631.362227
Sum of electronic and thermal Energies= -1631.329351
Sum of electronic and thermal Enthalpies= -1631.328407
Sum of electronic and thermal Free Energies= -1631.435018
M06-2X/6-311++G(d,p)/SMD(THF)//B3LYP/6-31G(d) energy = -1631.648035

3aa

Center Number	Atomic Number	Atomic Type	Coordinates (Angstroms) X	Y	Z
1	6	0	4.275932	-2.669602	1.832823
2	6	0	4.230268	-2.316104	0.483720
3	6	0	3.116070	-1.655007	-0.031850
4	6	0	2.033123	-1.342581	0.802220
5	6	0	2.079651	-1.711979	2.155818
6	6	0	3.195852	-2.367467	2.666852
7	1	0	5.146724	-3.182104	2.232813
8	1	0	5.061729	-2.558110	-0.172432
9	1	0	3.080611	-1.395481	-1.083659
10	1	0	1.230211	-1.481693	2.789983
11	1	0	3.223599	-2.644928	3.717032
12	6	0	0.821144	-0.668564	0.293456
13	7	0	-0.327371	-0.755053	0.857977
14	6	0	-1.284519	-0.056212	0.016072
15	6	0	-2.195408	-1.087088	-0.667432
16	6	0	-2.277052	-1.177547	-2.058880
17	6	0	-2.955775	-1.958873	0.123192
18	6	0	-3.112876	-2.126917	-2.653942
19	1	0	-1.681853	-0.506442	-2.667994
20	6	0	-3.785437	-2.906764	-0.471606
21	1	0	-2.887675	-1.895019	1.205275
22	6	0	-3.868075	-2.992956	-1.864431
23	1	0	-3.169179	-2.188222	-3.737933
24	1	0	-4.368199	-3.580110	0.151794
25	1	0	-4.516796	-3.731482	-2.328313
26	6	0	-2.083607	1.005990	0.770300
27	6	0	-3.056024	1.750559	0.089640
28	6	0	-1.838714	1.275935	2.119667
29	6	0	-3.760810	2.757551	0.746019

30	1	0	-3.260550	1.537910	-0.955794
31	6	0	-2.554620	2.276973	2.780570
32	1	0	-1.093426	0.691446	2.648767
33	6	0	-3.513985	3.022509	2.095697
34	1	0	-4.508658	3.332114	0.205661
35	1	0	-2.360981	2.471931	3.832383
36	1	0	-4.070006	3.802548	2.609281
37	6	0	2.527744	1.131361	-2.307464
38	6	0	3.463255	2.143023	-2.533218
39	6	0	3.667146	3.131204	-1.569282
40	6	0	2.931854	3.112436	-0.380080
41	6	0	1.998247	2.104310	-0.150467
42	6	0	1.808597	1.106957	-1.112744
43	1	0	2.344101	0.357617	-3.046879
44	1	0	4.026936	2.160754	-3.461795
45	1	0	4.394411	3.919334	-1.744455
46	1	0	3.085611	3.885912	0.367349
47	1	0	1.406470	2.089651	0.760084
48	7	0	0.843398	0.049154	-0.923001
49	8	0	-0.483393	0.646096	-0.989237

Zero-point correction= 0.392113 (Hartree/Particle)
Thermal correction to Energy= 0.414843
Thermal correction to Enthalpy= 0.415787
Thermal correction to Gibbs Free Energy= 0.336015
Sum of electronic and zero-point Energies= -1187.085236
Sum of electronic and thermal Energies= -1187.062507
Sum of electronic and thermal Enthalpies= -1187.061562
Sum of electronic and thermal Free Energies= -1187.141334
M06-2X/6-311++G(d,p)/SMD(THF)//B3LYP/6-31G(d) energy = -1187.322622

TS2′

Center Number	Atomic Number	Atomic Type	Coordinates (Angstroms) X	Y	Z
1	6	0	3.894710	-0.387421	3.259843
2	6	0	3.810296	0.813563	2.554636
3	6	0	2.753991	1.028620	1.668830
4	6	0	1.771065	0.049884	1.482434
5	6	0	1.860978	-1.152914	2.195521
6	6	0	2.917691	-1.369930	3.077263
7	1	0	4.716148	-0.557258	3.951080
8	1	0	4.564285	1.583736	2.693370
9	1	0	2.693835	1.963257	1.116513
10	1	0	1.102876	-1.913567	2.047234
11	1	0	2.979066	-2.306475	3.625354
12	6	0	0.632912	0.294409	0.534889
13	1	0	0.610413	1.329782	0.174819
14	7	0	-0.611423	-0.053672	1.081288
15	6	0	-1.843504	0.019913	0.456084
16	6	0	-2.582444	-1.155676	0.113249
17	6	0	-2.086679	-2.467126	0.400000
18	6	0	-3.818570	-1.071725	-0.596599
19	6	0	-2.776177	-3.602159	-0.020285
20	1	0	-1.162837	-2.568231	0.959064
21	6	0	-4.494292	-2.215096	-0.997775
22	1	0	-4.222308	-0.094129	-0.837315
23	6	0	-3.982815	-3.493370	-0.723465
24	1	0	-2.373466	-4.583347	0.219551

25	1	0	-5.430378	-2.113169	-1.541040
26	1	0	-4.517191	-4.382557	-1.043729
27	6	0	-2.431692	1.391430	0.387105
28	6	0	-1.950690	2.327282	-0.543719
29	6	0	-3.461104	1.768089	1.266064
30	6	0	-2.496713	3.610162	-0.595533
31	1	0	-1.178210	2.014715	-1.240264
32	6	0	-4.001830	3.052337	1.211377
33	1	0	-3.830166	1.049728	1.992779
34	6	0	-3.518827	3.977169	0.282543
35	1	0	-2.126955	4.322849	-1.328289
36	1	0	-4.796409	3.332580	1.897935
37	1	0	-3.938809	4.978783	0.242677
38	3	0	-0.837200	-1.794695	-1.537843
39	6	0	3.556130	1.550370	-2.624689
40	6	0	2.310591	1.150157	-2.140033
41	6	0	2.202564	-0.075149	-1.485539
42	6	0	3.302130	-0.909735	-1.315839
43	6	0	4.541686	-0.500452	-1.810951
44	6	0	4.673417	0.727981	-2.460446
45	1	0	3.648972	2.500749	-3.143489
46	1	0	1.425769	1.755857	-2.292803
47	1	0	3.168254	-1.857350	-0.811371
48	1	0	5.406052	-1.147110	-1.686643
49	1	0	5.640764	1.041182	-2.843979
50	7	0	0.877629	-0.495944	-0.947543
51	8	0	0.824991	-1.835984	-0.718377
52	8	0	-0.128552	-0.074304	-1.772114

Zero-point correction= 0.404287 (Hartree/Particle)
Thermal correction to Energy= 0.429571
Thermal correction to Enthalpy= 0.430515
Thermal correction to Gibbs Free Energy= 0.346262
Sum of electronic and zero-point Energies= -1270.355798
Sum of electronic and thermal Energies= -1270.330514
Sum of electronic and thermal Enthalpies= -1270.329570
Sum of electronic and thermal Free Energies= -1270.413823
M06-2X/6-311++G(d,p)/SMD(THF)//B3LYP/6-31G(d) energy = -1270.593086

TS2″

Center Number	Atomic Number	Atomic Type	Coordinates (Angstroms) X	Y	Z
1	6	0	5.647467	2.176711	-1.028696
2	6	0	5.062633	2.547194	0.184366
3	6	0	3.888316	1.930001	0.608636
4	6	0	3.277104	0.930139	-0.171045
5	6	0	3.877535	0.565926	-1.392907
6	6	0	5.048600	1.184156	-1.814224
7	1	0	6.563089	2.657694	-1.362341
8	1	0	5.521843	3.317458	0.798371
9	1	0	3.432385	2.218892	1.553216
10	1	0	3.403425	-0.202579	-1.994085
11	1	0	5.500904	0.895938	-2.759750
12	6	0	2.051365	0.303534	0.308150
13	1	0	1.657501	0.686617	1.257074
14	7	0	1.454516	-0.648154	-0.342896
15	6	0	0.266158	-1.191589	0.126216
16	6	0	-0.146484	-2.432141	-0.607013

17	6	0	0.418432	-2.748806	-1.861615
18	6	0	-1.223352	-3.224418	-0.150990
19	6	0	-0.071432	-3.811651	-2.621675
20	1	0	1.242792	-2.141919	-2.217232
21	6	0	-1.711561	-4.286058	-0.918462
22	1	0	-1.678003	-3.001426	0.809207
23	6	0	-1.141541	-4.586472	-2.158636
24	1	0	0.386198	-4.036922	-3.581653
25	1	0	-2.540062	-4.879540	-0.541001
26	1	0	-1.522031	-5.411683	-2.753590
27	6	0	-0.012504	-1.182437	1.615155
28	6	0	-1.095881	-0.512464	2.194731
29	6	0	0.858437	-1.901862	2.452369
30	6	0	-1.304632	-0.561087	3.576113
31	1	0	-1.767722	0.046320	1.555875
32	6	0	0.650374	-1.949413	3.829685
33	1	0	1.704979	-2.426723	2.016745
34	6	0	-0.434991	-1.277385	4.397547
35	1	0	-2.151095	-0.032825	4.007540
36	1	0	1.335458	-2.511633	4.459106
37	1	0	-0.599078	-1.312976	5.471392
38	3	0	-1.819356	-1.284522	-1.688638
39	6	0	-1.893579	4.108682	0.300500
40	6	0	-1.690856	2.753027	0.070027
41	6	0	-2.673919	2.017329	-0.618569
42	6	0	-3.849761	2.647614	-1.075639
43	6	0	-4.030662	4.004222	-0.832084
44	6	0	-3.060284	4.741490	-0.143987
45	1	0	-1.133282	4.679424	0.826891
46	1	0	-0.789140	2.244636	0.387284
47	1	0	-4.590144	2.060059	-1.605698
48	1	0	-4.936353	4.492611	-1.182412
49	1	0	-3.210506	5.801375	0.040941
50	7	0	-2.484011	0.655043	-0.818029
51	8	0	-3.267682	-0.056454	-1.537731
52	8	0	-0.906884	-0.043909	-0.662422

Zero-point correction= 0.402753 (Hartree/Particle)
Thermal correction to Energy= 0.429385
Thermal correction to Enthalpy= 0.430329
Thermal correction to Gibbs Free Energy= 0.340748
Sum of electronic and zero-point Energies= -1270.379214
Sum of electronic and thermal Energies= -1270.352582
Sum of electronic and thermal Enthalpies= -1270.351638
Sum of electronic and thermal Free Energies= -1270.441219
M06-2X/6-311++G(d,p)/SMD(THF)//B3LYP/6-31G(d) energy = -1270.592219

Supplementary References

1. Li, K., Weber, A. E., Tseng, L., Malcolmson, S. J. *Org. Lett*. **2017**, *19*, 4239.
2. Schaufelberger, F., Hu, L., Ramström, O. *Chem. Eur. J.* **2015**, *21*. 9776.
3. Cai, B. G., Chen, Z. L., Xu, G. Y., Xuan, J., Xiao, W. J. *Org. Lett.* **2019**, *21*, 4234.
4. Raghavendra, B., Bakthavachalam, K., Ramakrishna, B., Reddy, N. D. *Organometallics* **2017**, *36*, 4005.
5. (a) Becke, A. D. *J. Chem. Phys*. **1993**, *98*, 5648. (b) Lee, C., Yang, W., Parr, R. G. *Phys. Rev. B*, **1988**, *37*, 785.
6. Hariharan, P. C., Pople, J. A. *Theor. Chim. Acta.* **1973**, *28*, 213.
7. Fukui, K. *Acc. Chem. Res.* **1981**, *14*, 363.
8. Marenich, A. V., Cramer, C. J., Truhlar, D. G. *J. Phys. Chem. B* **2009**, *113*, 6378.
9. (a) Zhao, Y.; Truhlar, D. G. *Acc. Chem. Res.* **2008**, *41*, 157. (b) Zhao, Y.; Truhlar, D. G. *Theor. Chem. Acc.* **2008**, *120*, 215.
10. Frisch, M. J., Trucks, G. W., Schlegel, H. B., Scuseria, G. E., Robb, M. A., Cheeseman, J. R., Scalmani, G., Barone, V., Mennucci, B., Petersson, G. A., Nakatsuji, H., Caricato, M., Li, X., Hratchian, H. P., Izmaylov, A. F., Bloino, J., Zheng, G., Sonnenberg, J. L., Hada, M., Ehara, M., Toyota, K., Fukuda, R., Hasegawa, J., Ishida, M., Nakajima, T., Honda, Y., Kitao, O., Nakai, H., Vreven, T., Montgomery, J. A., Peralta, J. E., Ogliaro, F., Bearpark, M., Heyd, J. J., Brothers, E., Kudin, K. N., Staroverov, V. N., Kobayashi, R., Normand, J., Raghavachari, K., Rendell, A., Burant, J. C., Iyengar, S. S., Tomasi, J., Cossi, M., Rega, N., Millam, N. J., Klene, M., Knox, J. E., Cross, J. B., Bakken, V., Adamo, C., Jaramillo, J., Gomperts, R., Stratmann, R. E., Yazyev, O., Austin, A. J., Cammi, R., Pomelli, C., Ochterski, J. W., Martin, R. L., Morokuma, K., Zakrzewski, V. G., Voth, G. A., Salvador, P., Dannenberg, J. J., Dapprich, S., Daniels, A. D., Farkas, O., Foresman, J. B., Ortiz, J. V., Cioslowski, J., Fox, D. J., *Gaussian 09, Revision C.01*, Gaussian, Inc., Wallingford CT, **2010**.

NMR Spectra

Supplementary Figure 3. ^{1}H NMR Spectrum of 3aa (500 MHz, $CDCl_3$)

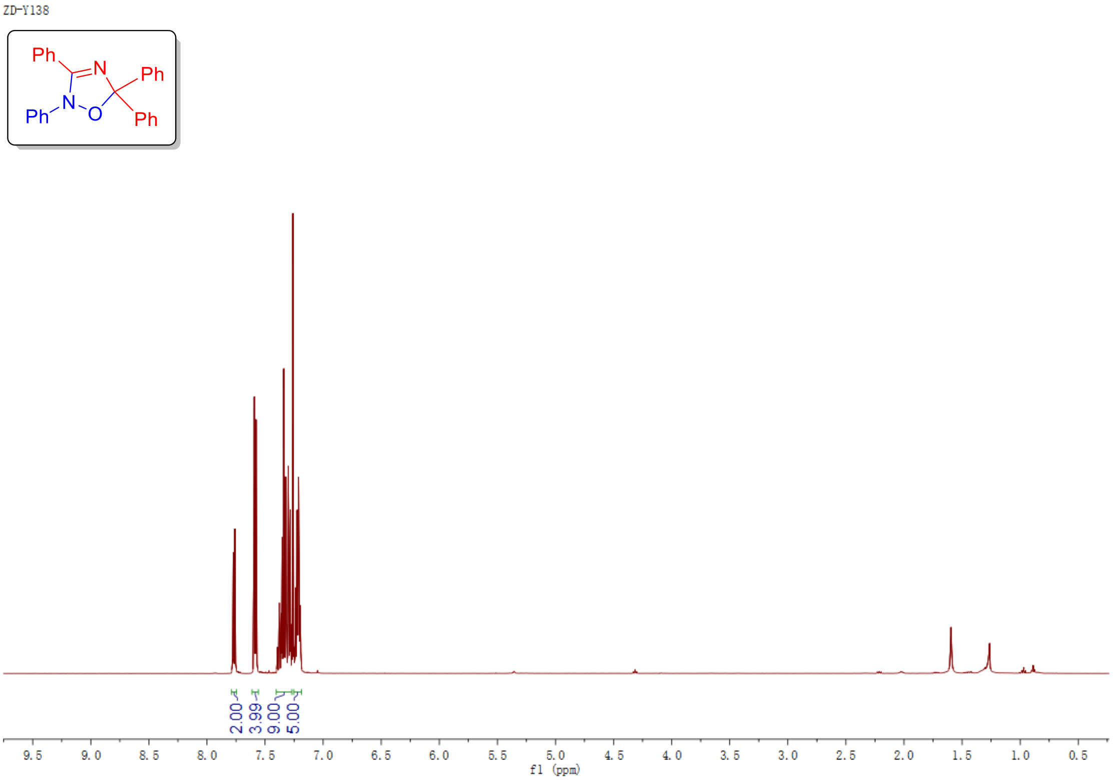

Supplementary Figure 4. ^{13}C NMR Spectrum of 3aa (125 MHz, $CDCl_3$)

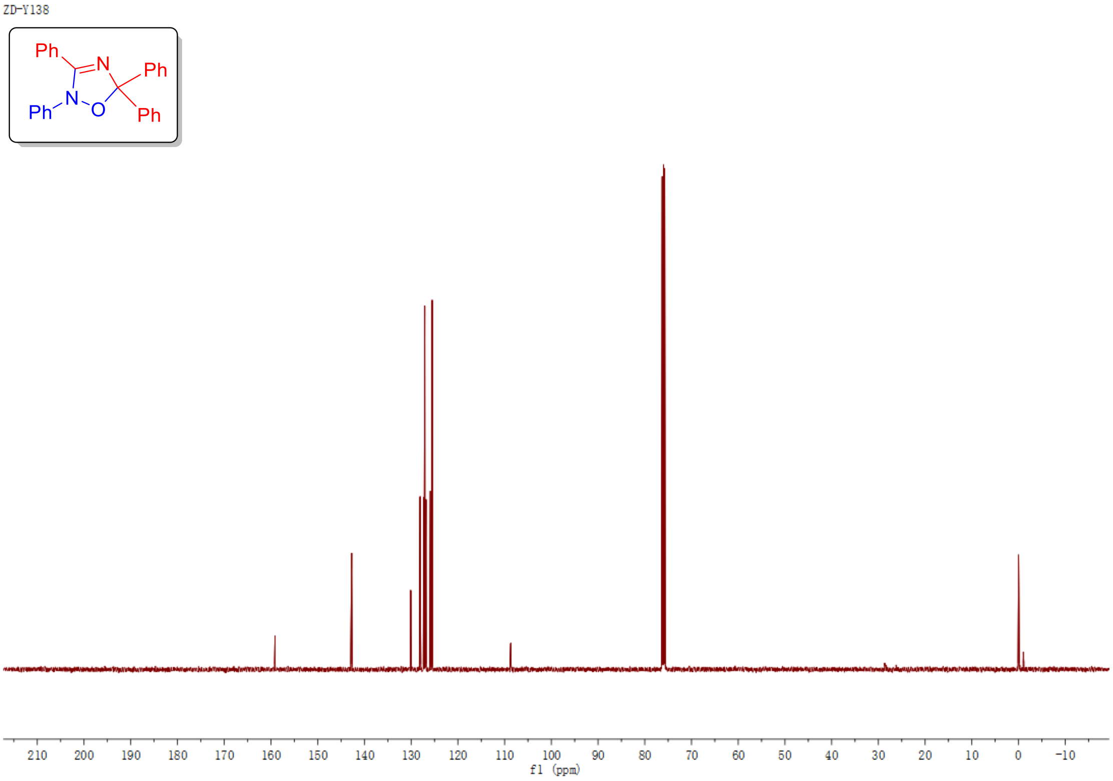

Supplementary Figure 5. ^{1}**H NMR Spectrum of 3ab (500 MHz, CDCl$_3$)**

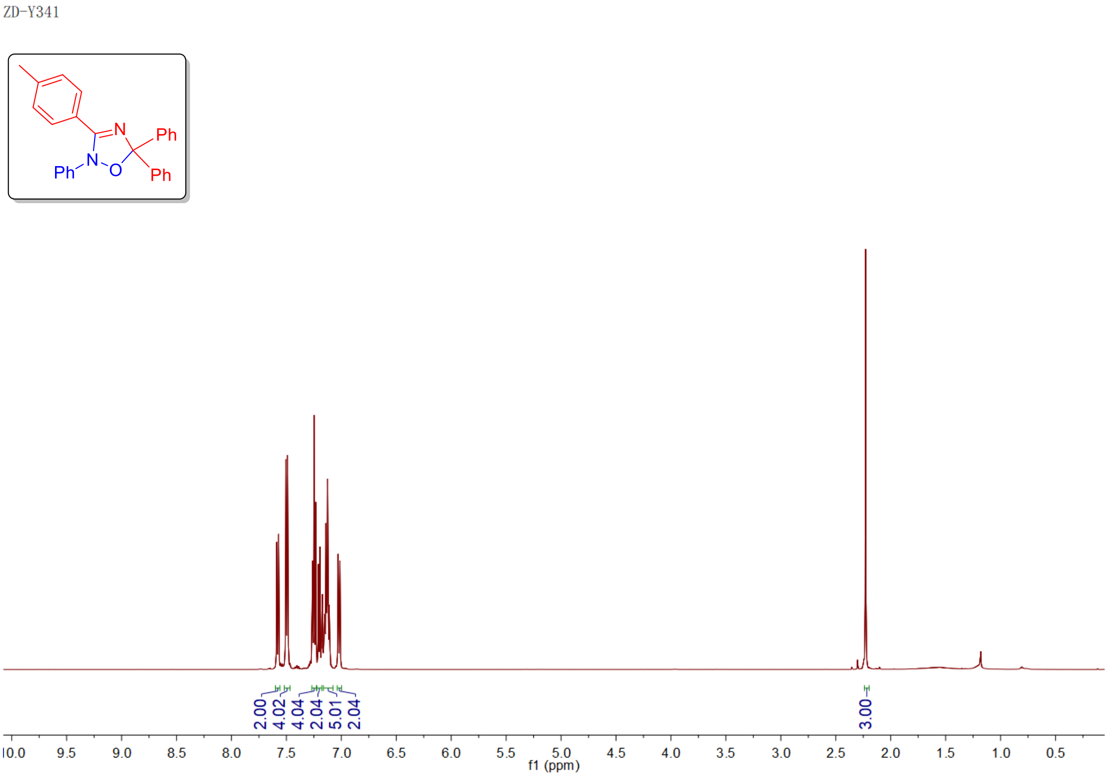

Supplementary Figure 6. ^{13}**C NMR Spectrum of 3ab (125 MHz, CDCl$_3$)**

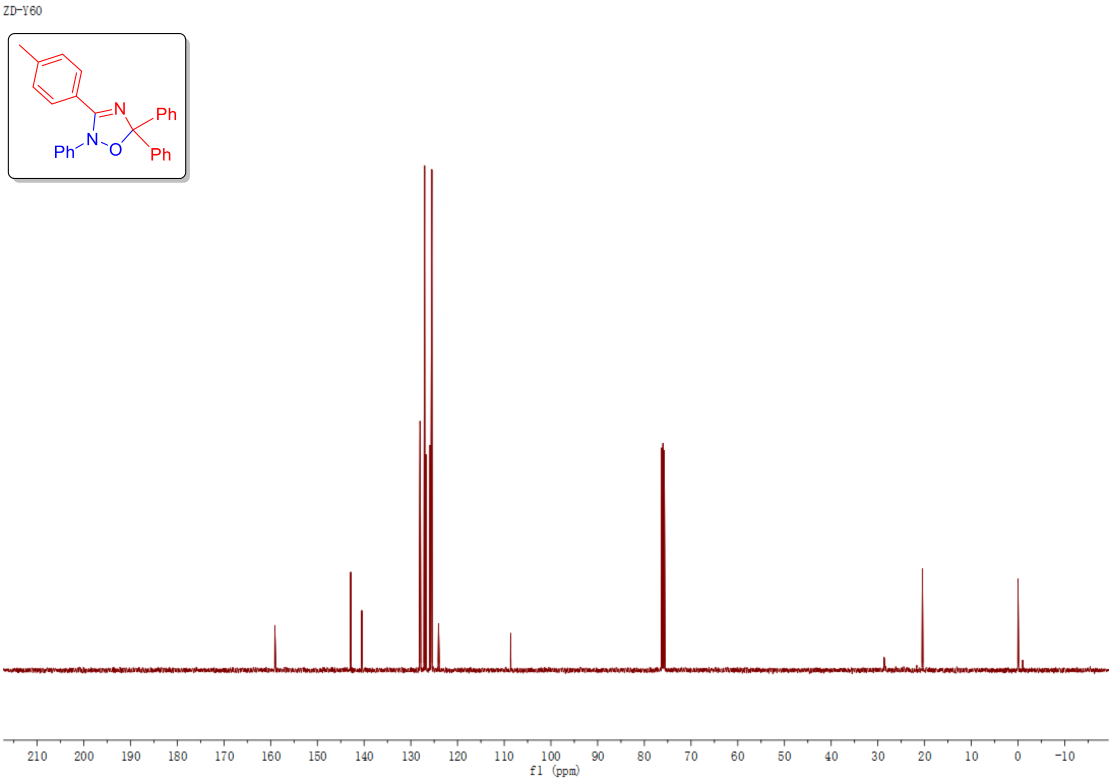

Supplementary Figure 7. ^{1}H NMR Spectrum of 3ac (500 MHz, $CDCl_3$)

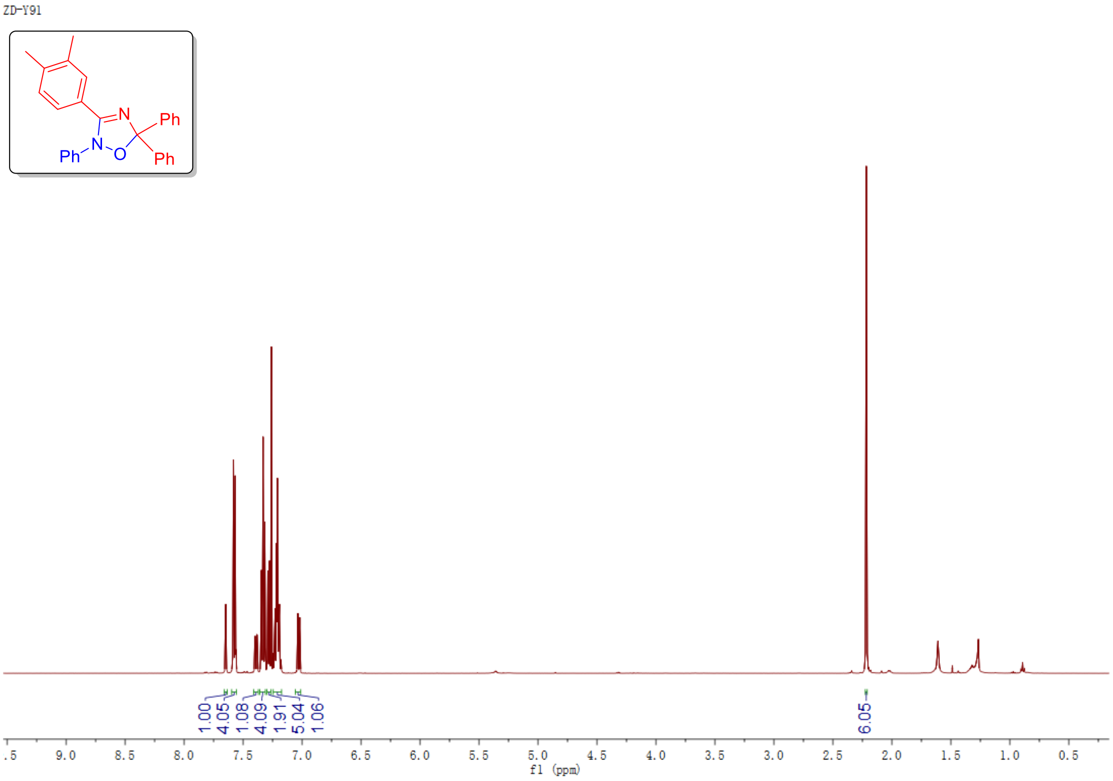

Supplementary Figure 8. ^{13}C NMR Spectrum of 3ac (125 MHz, $CDCl_3$)

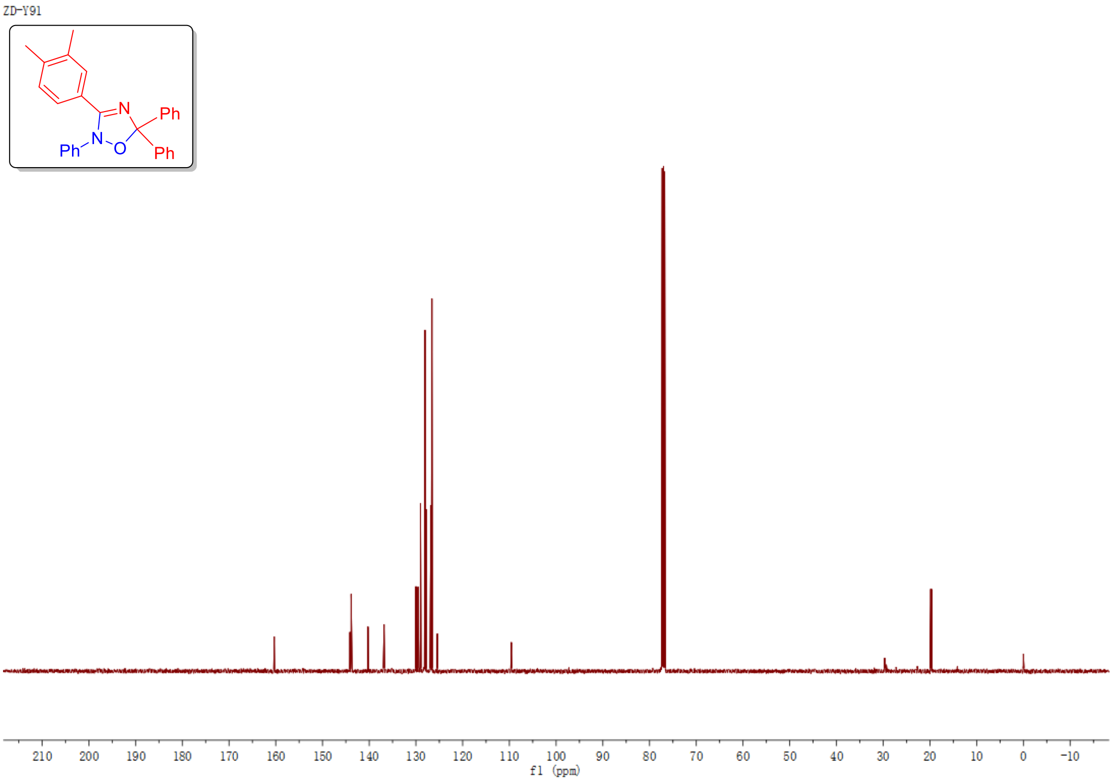

Supplementary Figure 9. ^{1}H NMR Spectrum of 3ad (500 MHz, $CDCl_3$)

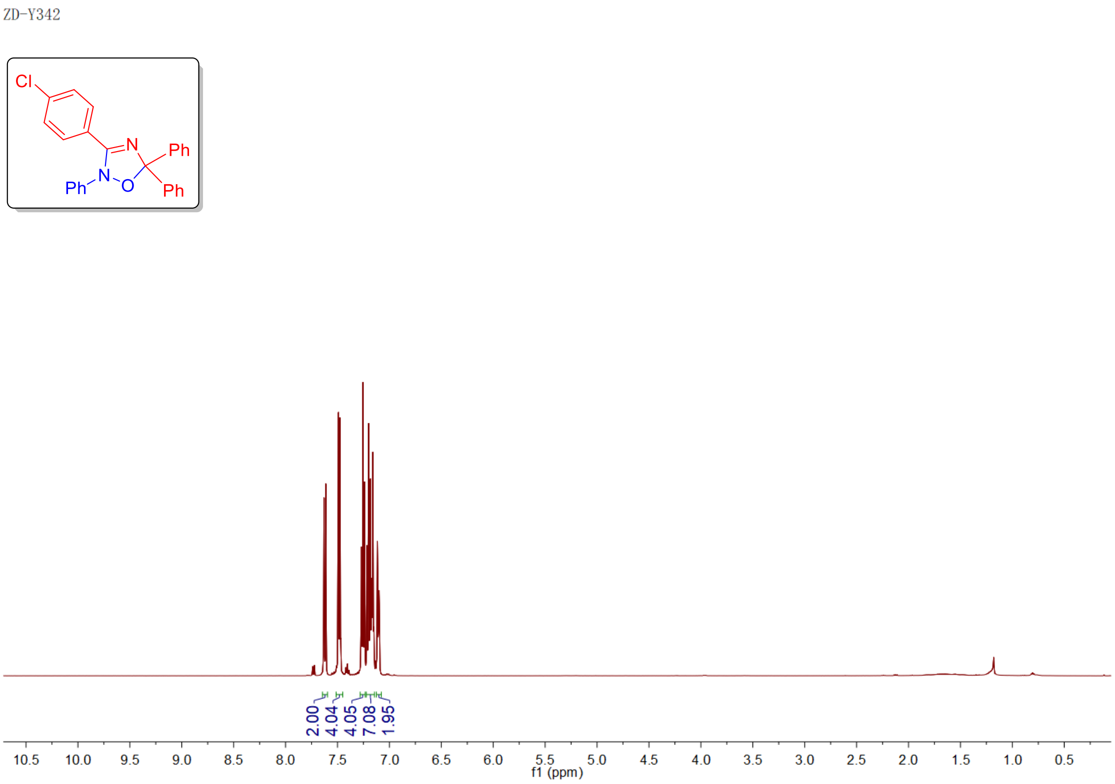

Supplementary Figure 10. ^{13}C NMR Spectrum of 3ad (125 MHz, $CDCl_3$)

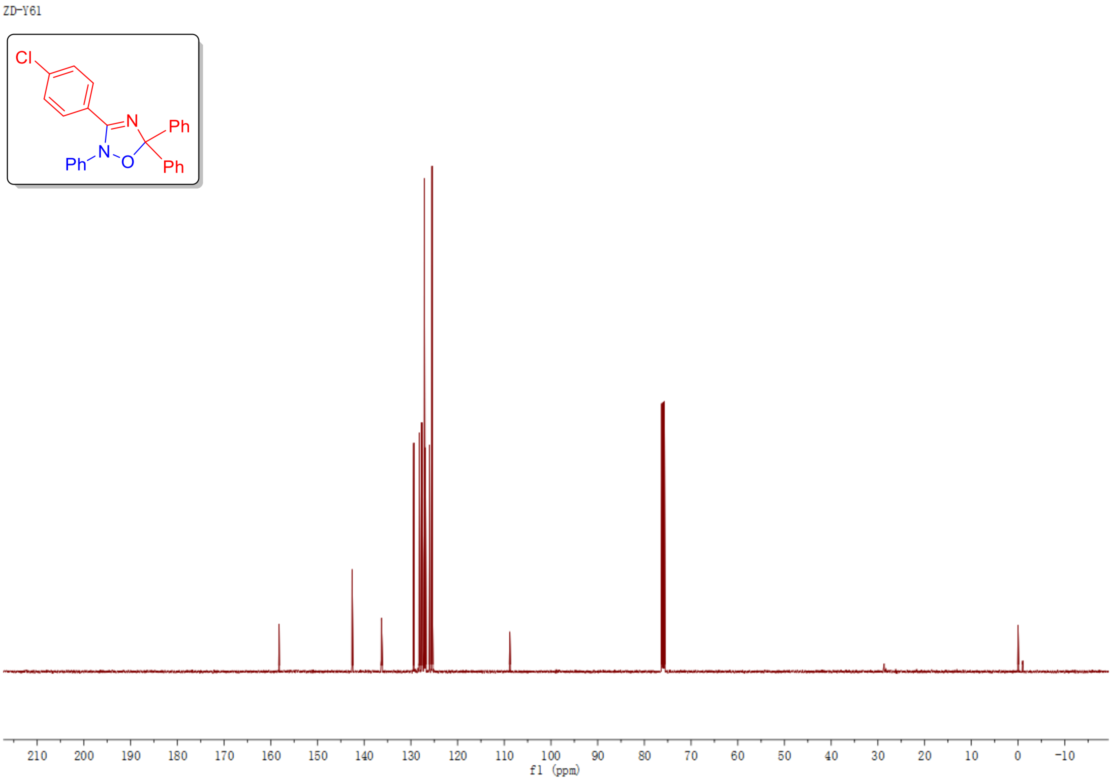

Supplementary Figure 11. ^{1}**H NMR Spectrum of 3ae (500 MHz, CDCl$_3$)**

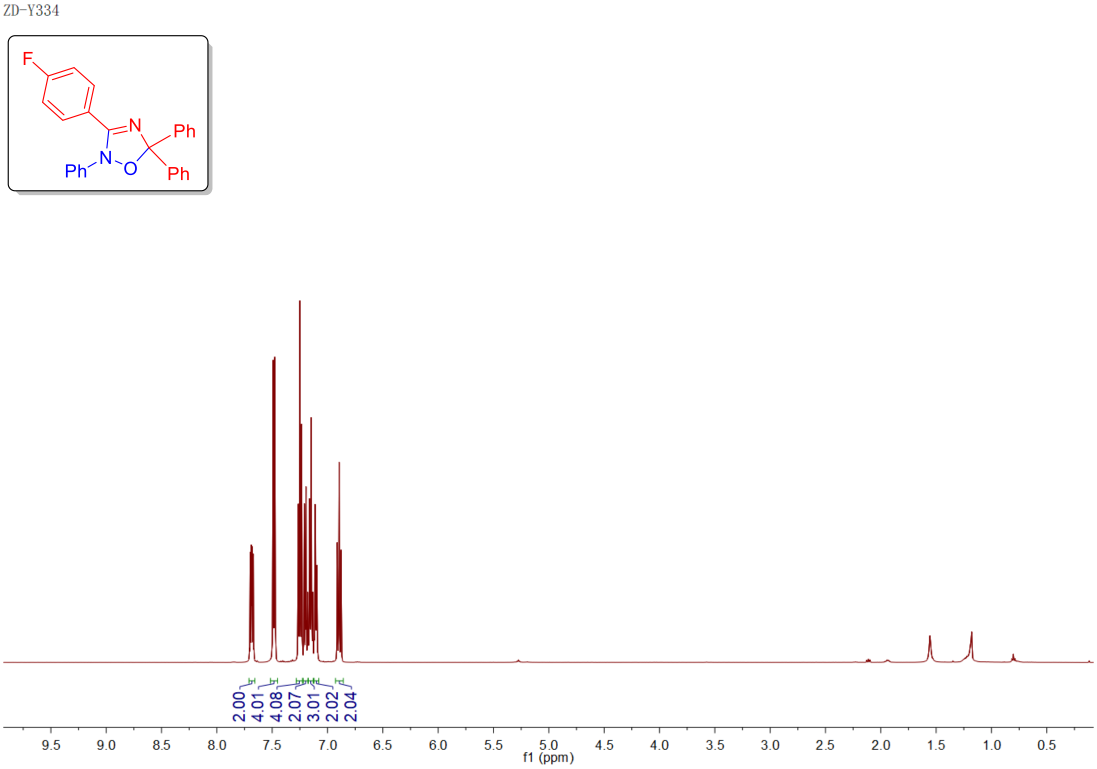

Supplementary Figure 12. ^{13}**C NMR Spectrum of 3ae (125 MHz, CDCl$_3$)**

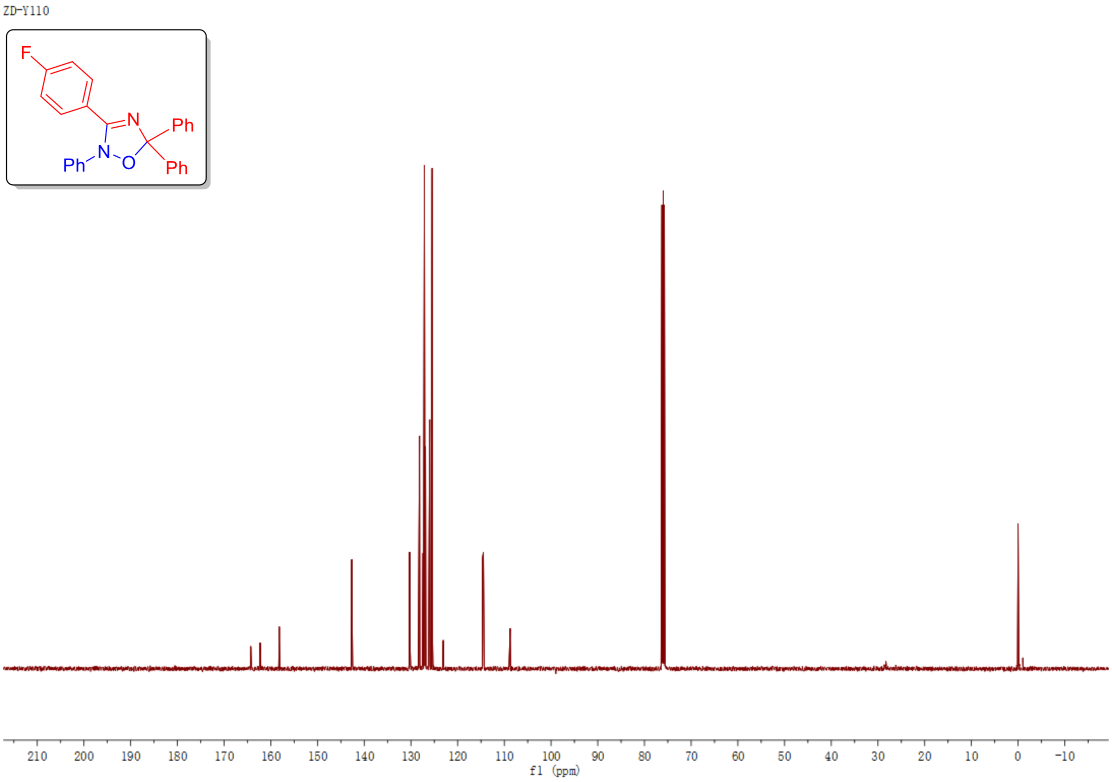

Supplementary Figure 13. ^{1}**H NMR Spectrum of 3af (500 MHz,** $CDCl_3$**)**

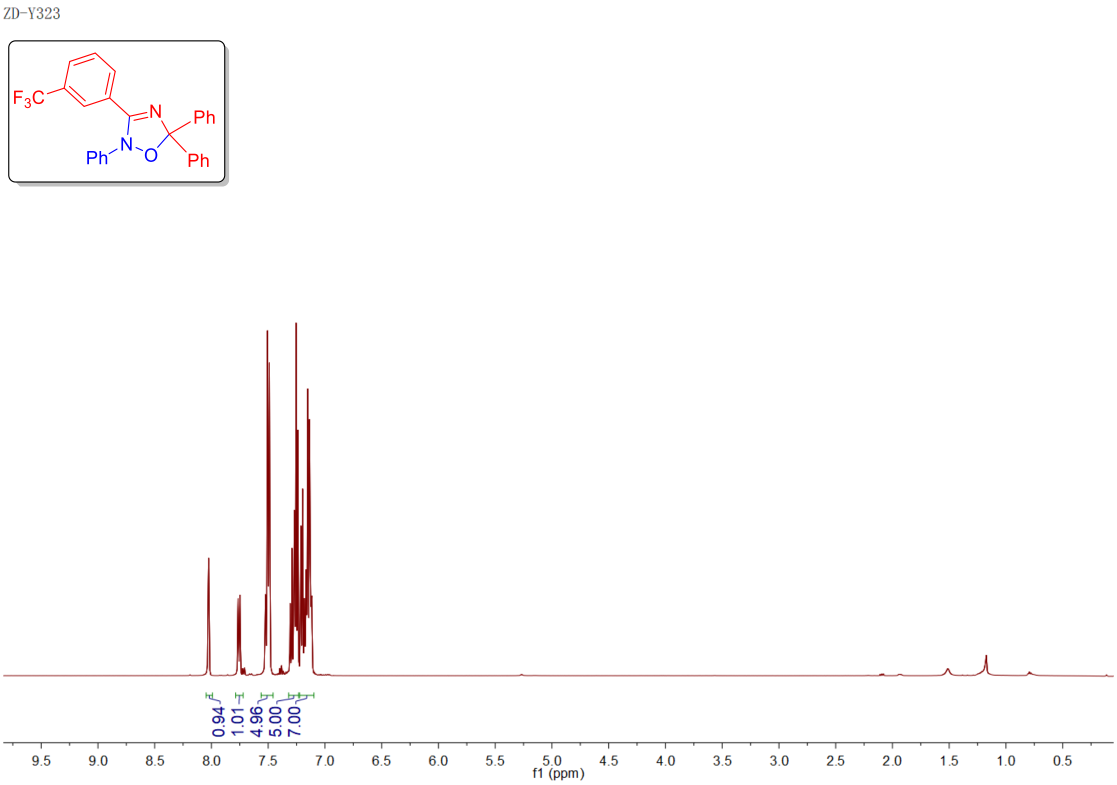

Supplementary Figure 14. ^{13}**C NMR Spectrum of 3af (125 MHz,** $CDCl_3$**)**

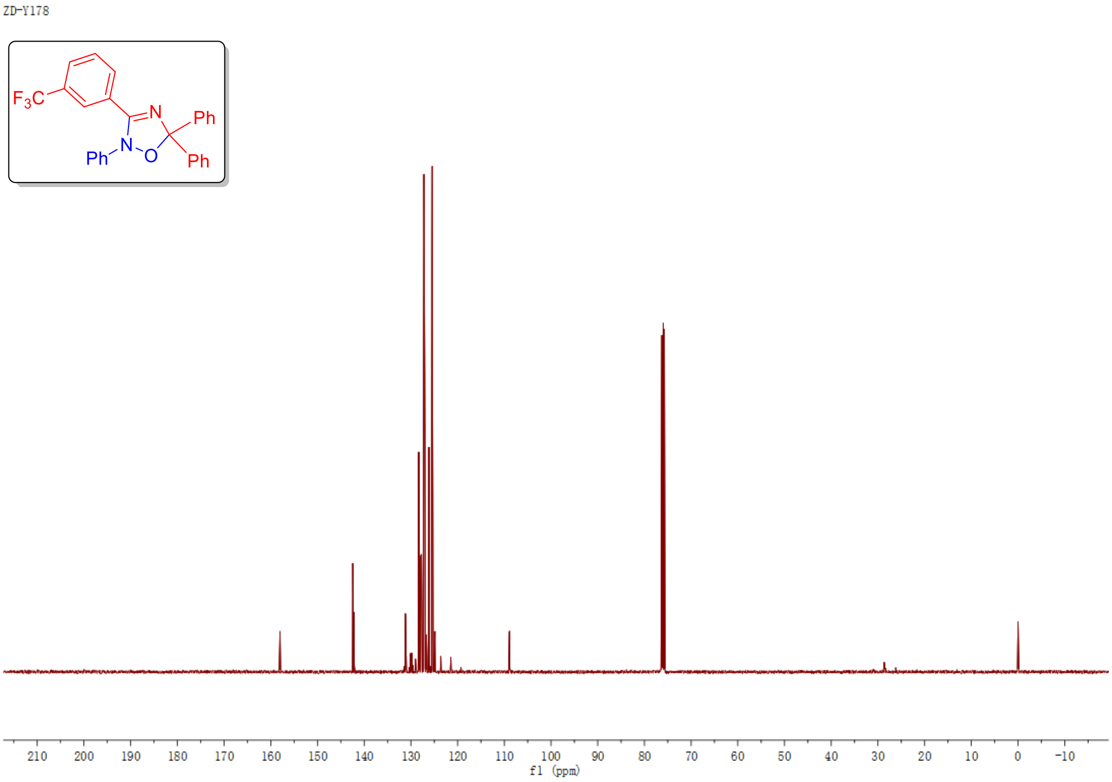

Supplementary Figure 15. ¹H NMR Spectrum of 3ag (500 MHz, CDCl₃)

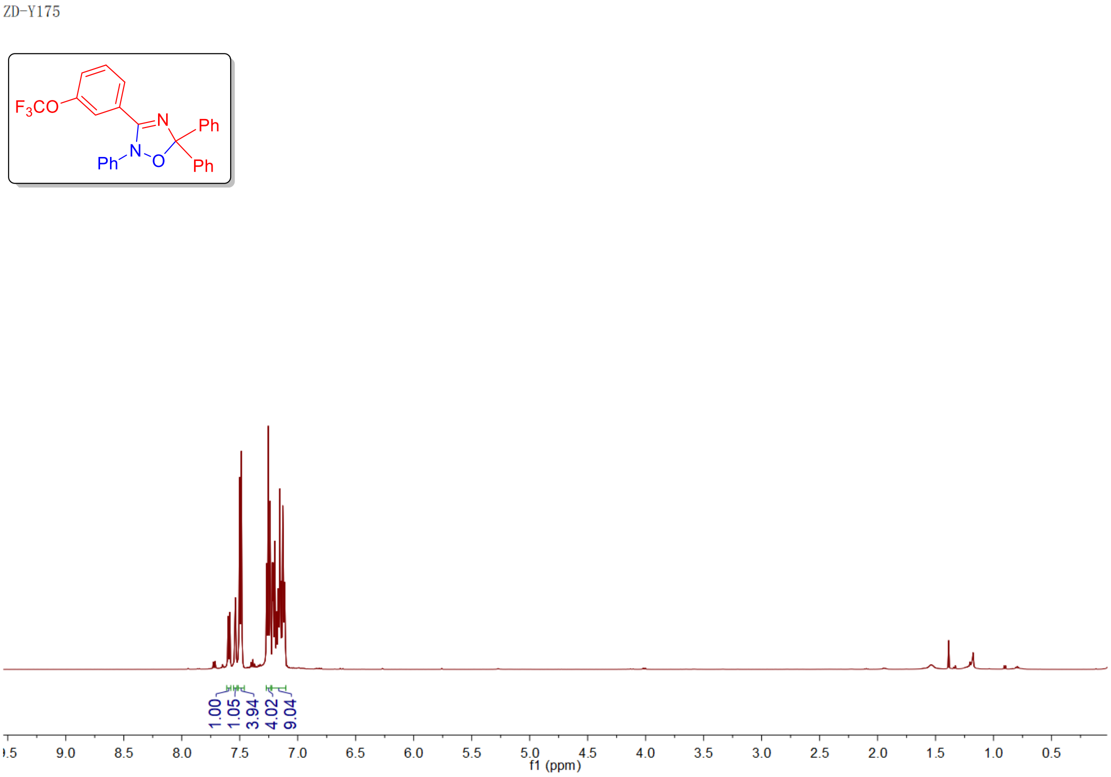

Supplementary Figure 16. ¹³C NMR Spectrum of 3ag (125 MHz, CDCl₃)

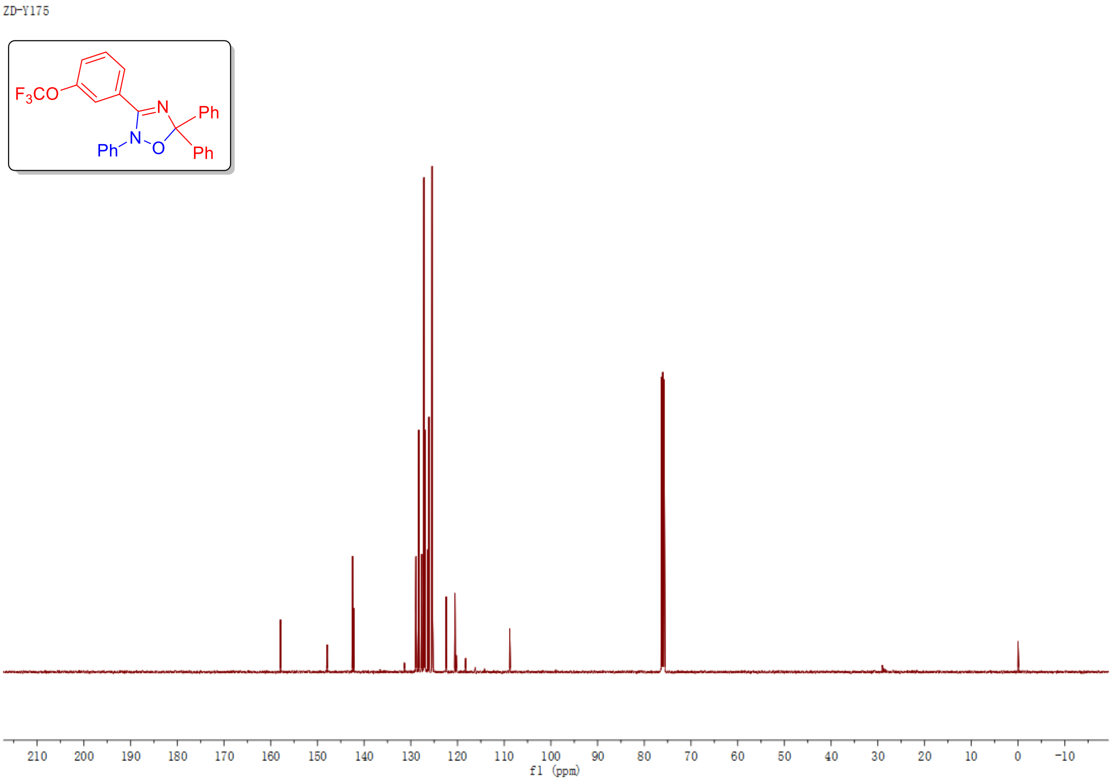

Supplementary Figure 17. ^{1}H NMR Spectrum of 3ah (500 MHz, $CDCl_3$)

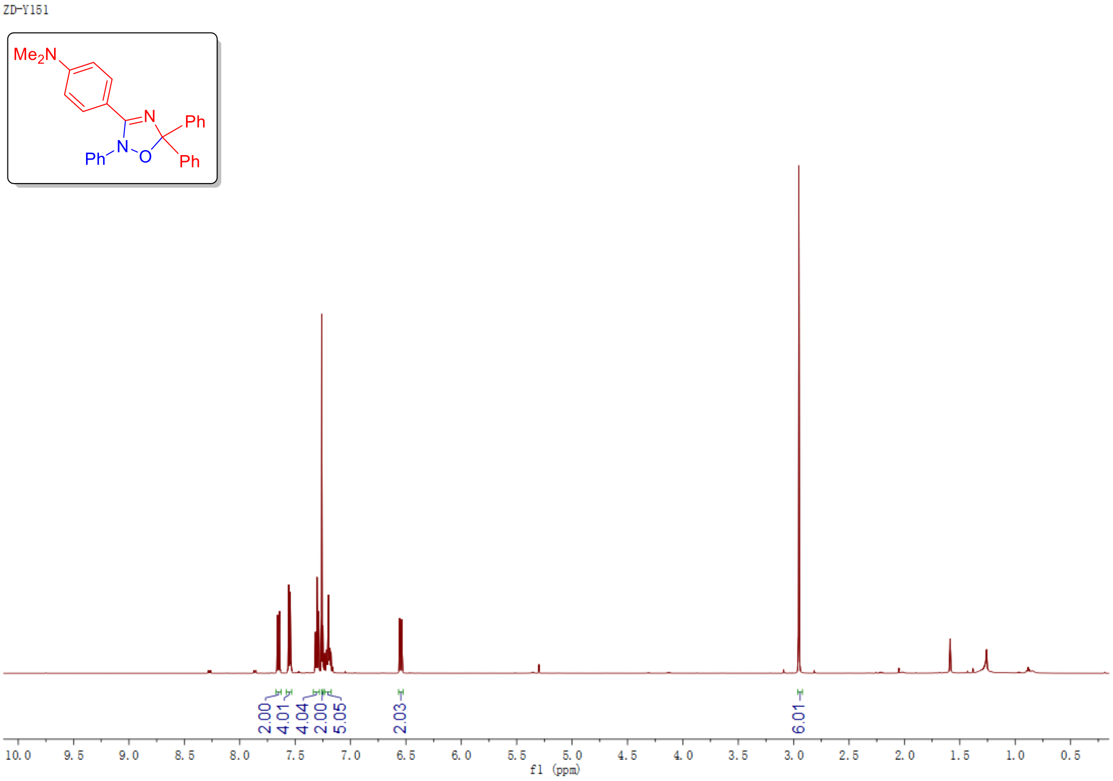

Supplementary Figure 18. ^{13}C NMR Spectrum of 3ah (125 MHz, $CDCl_3$)

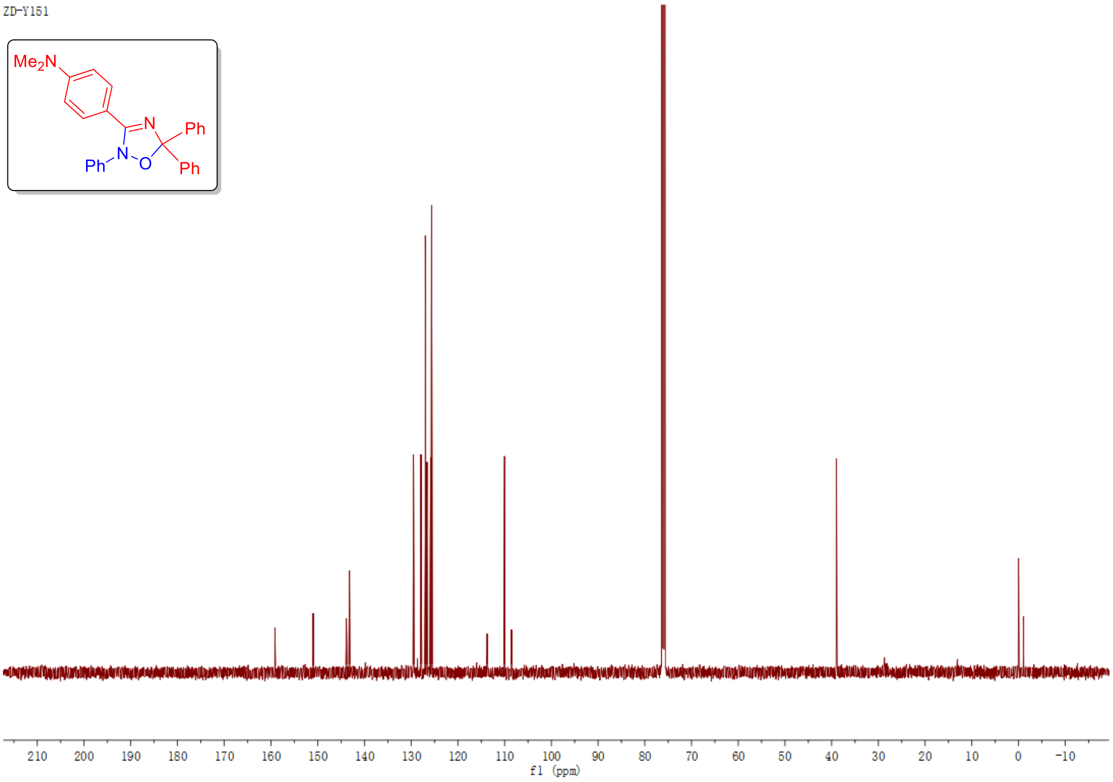

Supplementary Figure 19. ^{1}**H NMR Spectrum of 3ai (500 MHz, CDCl$_3$)**

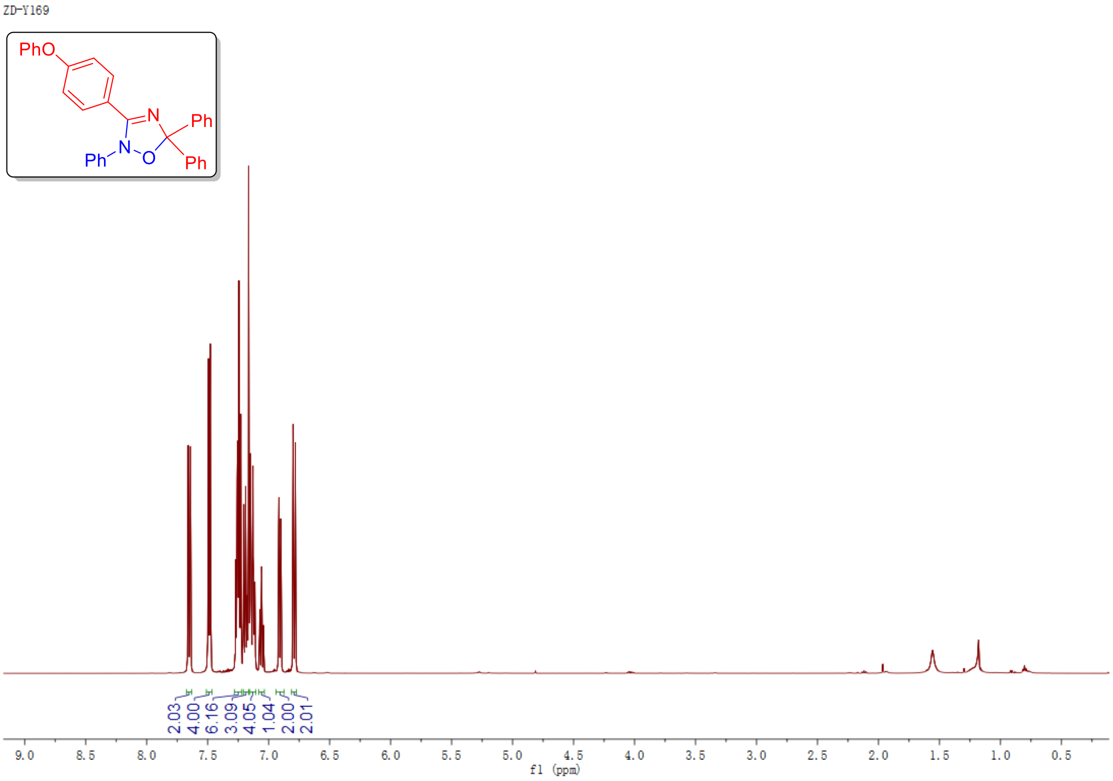

Supplementary Figure 20. ^{13}**C NMR Spectrum of 3ai (125 MHz, CDCl$_3$)**

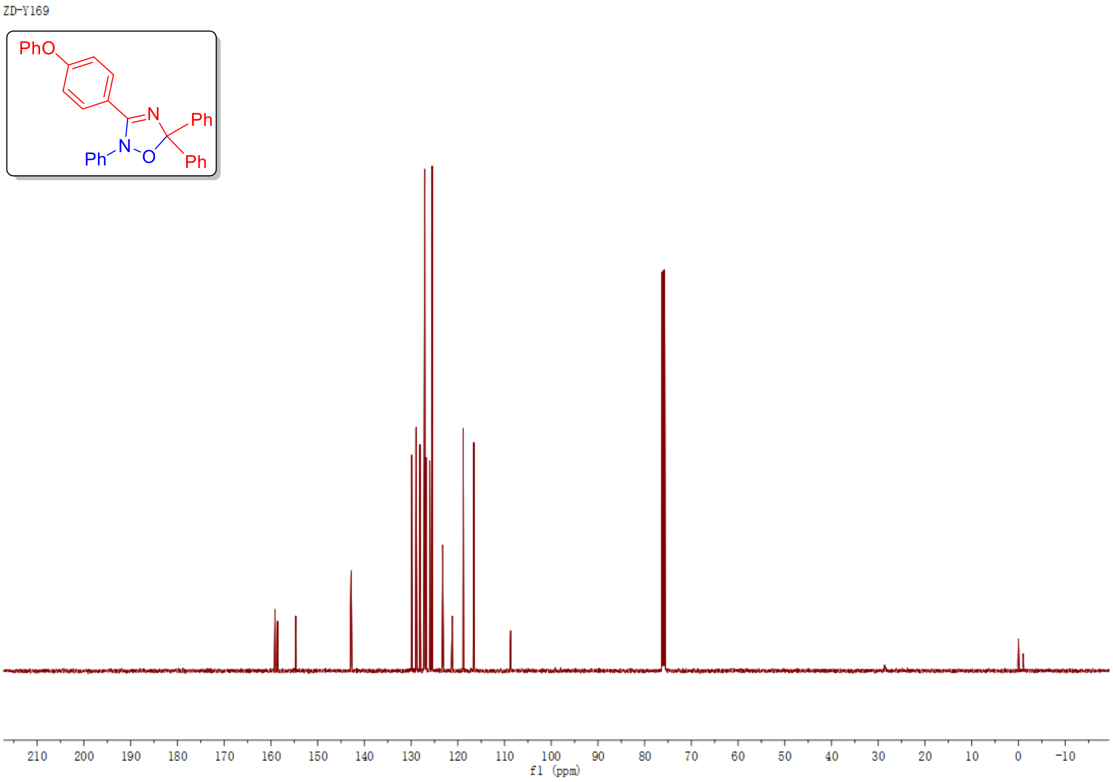

Supplementary Figure 21. ^{1}H NMR Spectrum of 3aj (500 MHz, $CDCl_3$)

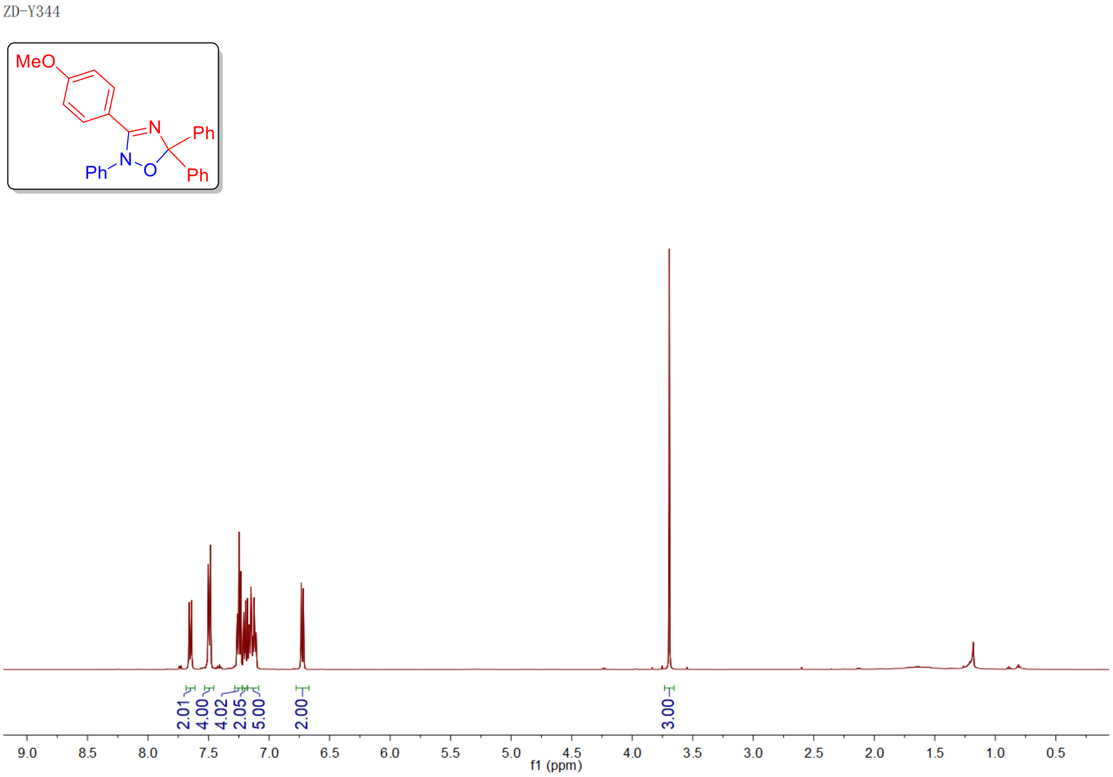

Supplementary Figure 22. ^{13}C NMR Spectrum of 3aj (125 MHz, $CDCl_3$)

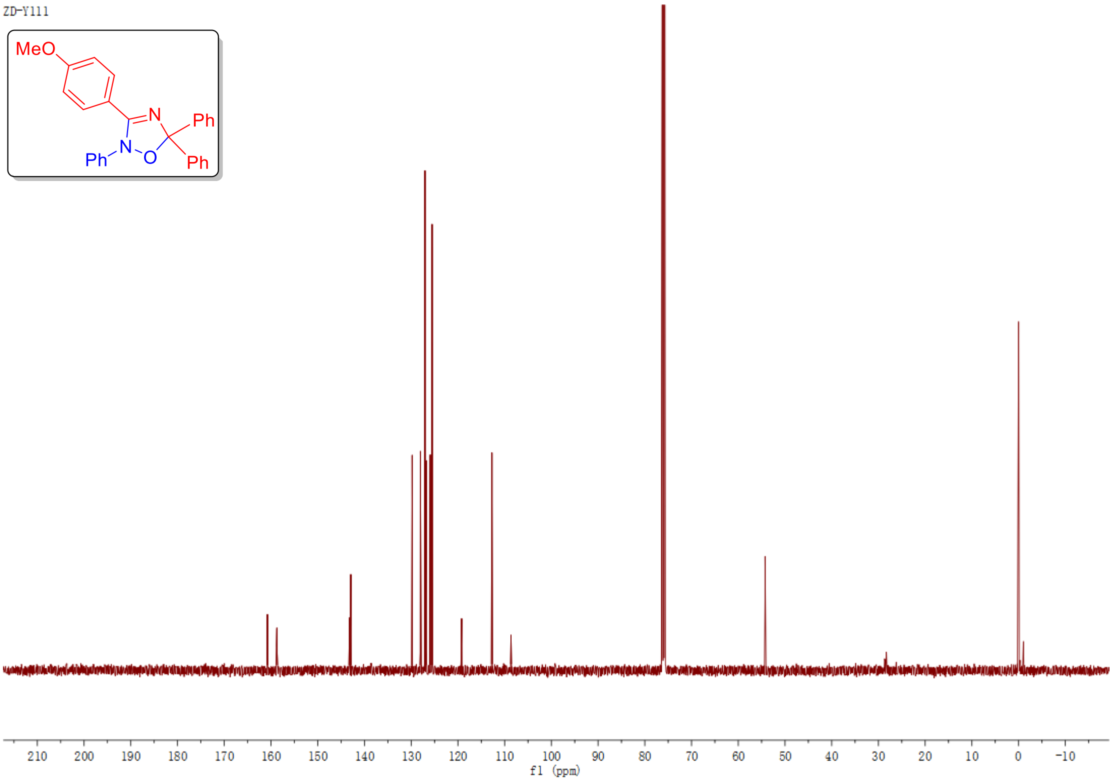

Supplementary Figure 23. ^{1}H NMR Spectrum of 3ak (500 MHz, $CDCl_3$)

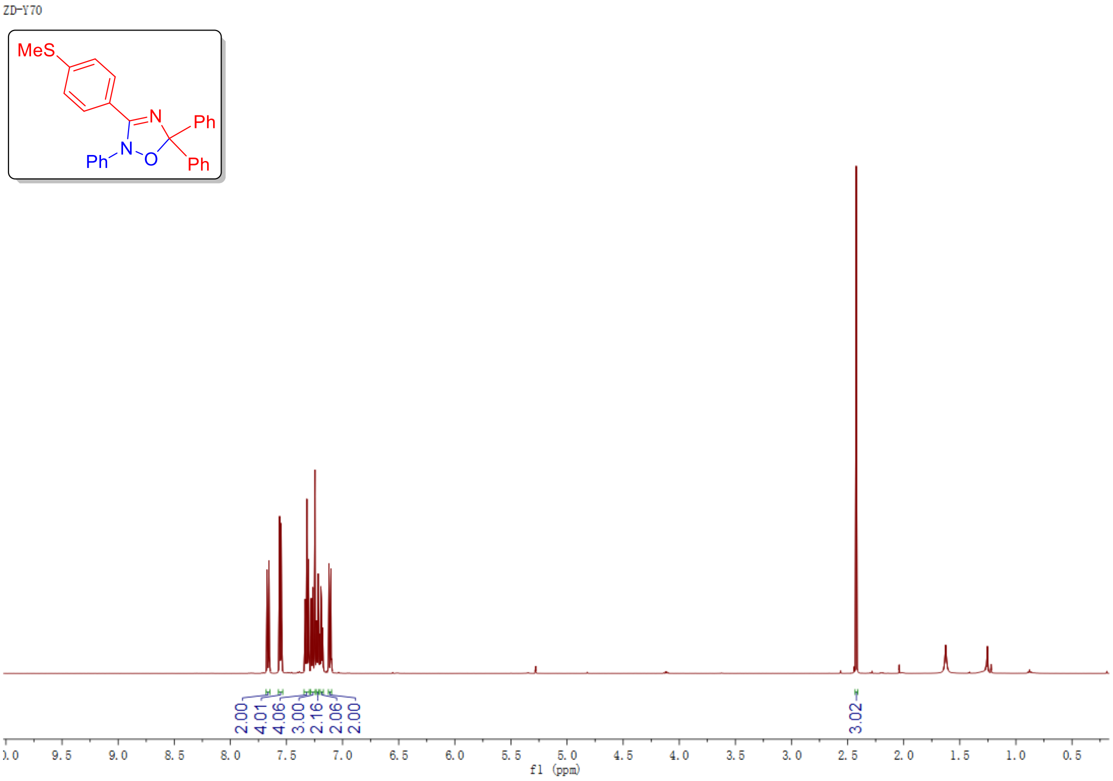

Supplementary Figure 24. ^{13}C NMR Spectrum of 3ak (125 MHz, $CDCl_3$)

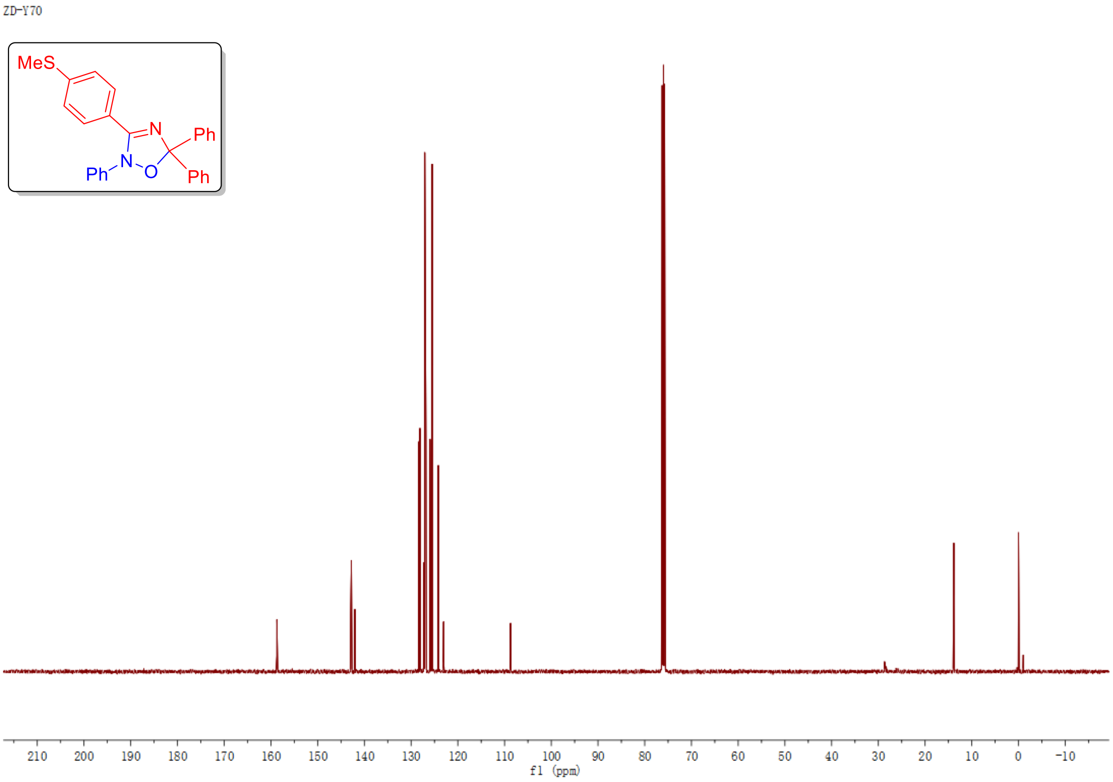

Supplementary Figure 25. **^{1}H NMR Spectrum of 3al (500 MHz, CDCl$_3$)**

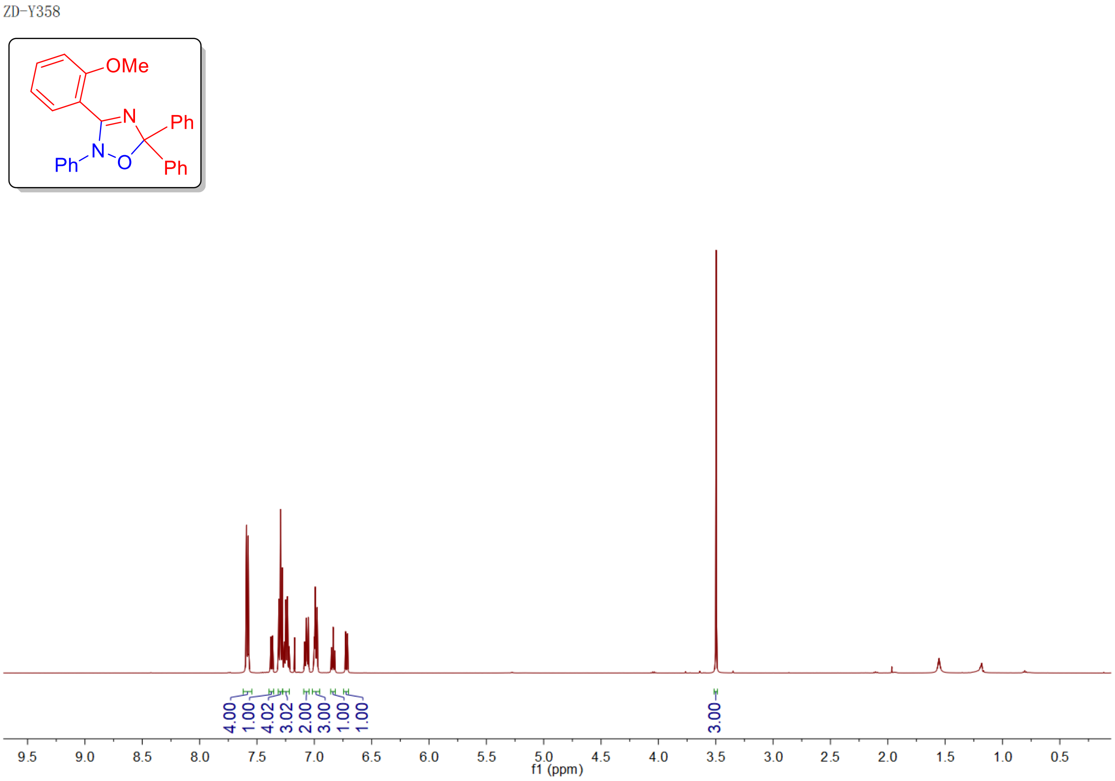

Supplementary Figure 26. **^{13}C NMR Spectrum of 3al (125 MHz, CDCl$_3$)**

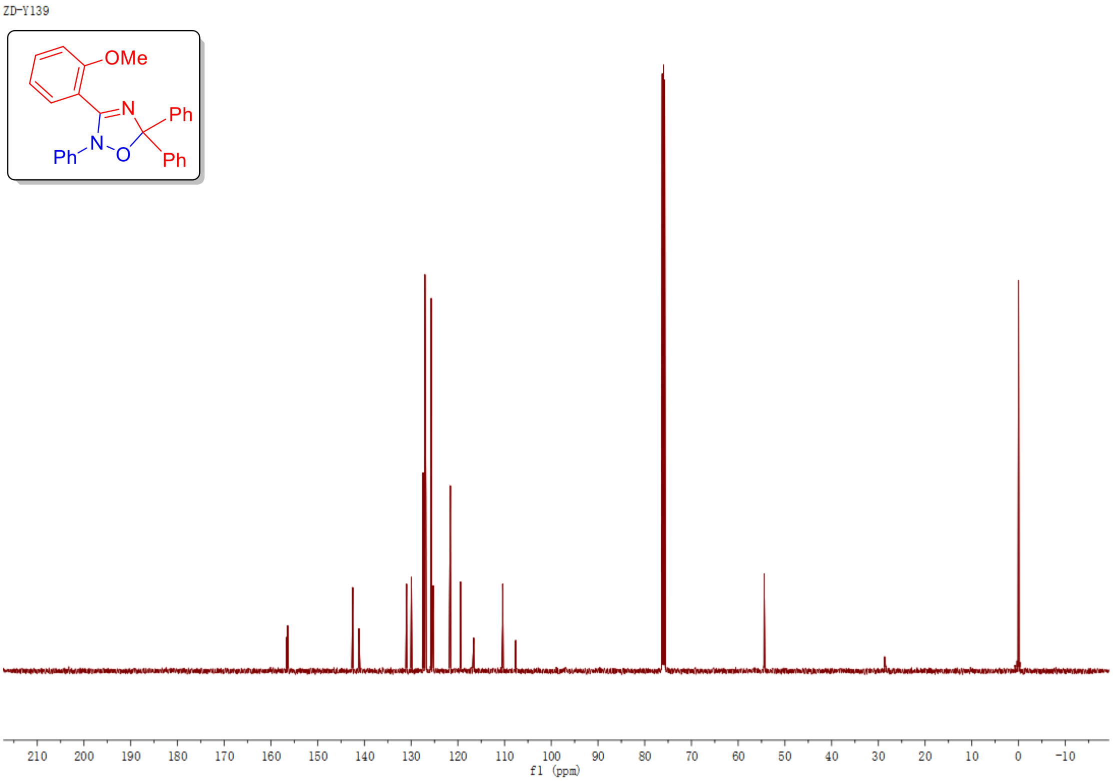

Supplementary Figure 27. ^{1}**H NMR Spectrum of 3am (500 MHz, CDCl$_3$)**

ZD-Y324

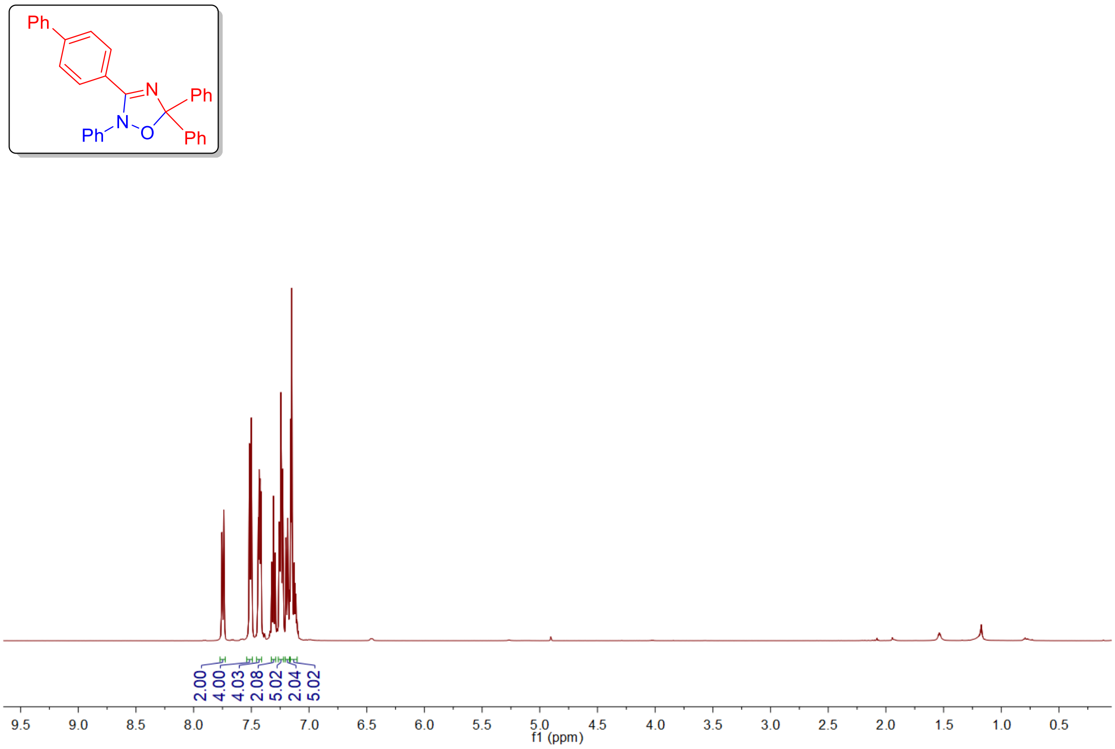

Supplementary Figure 28. ^{13}**C NMR Spectrum of 3am (125 MHz, CDCl$_3$)**

ZD-Y324

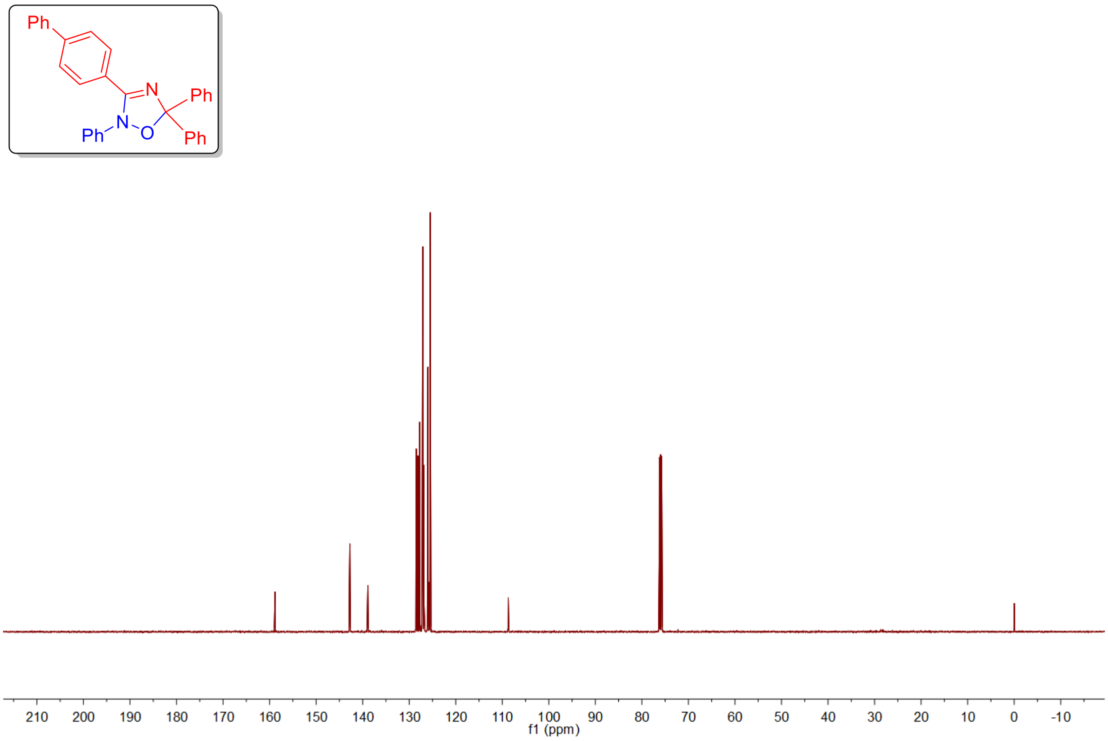

Supplementary Figure 29. ^{1}H NMR Spectrum of 3an (500 MHz, $CDCl_3$)

ZD-Y286

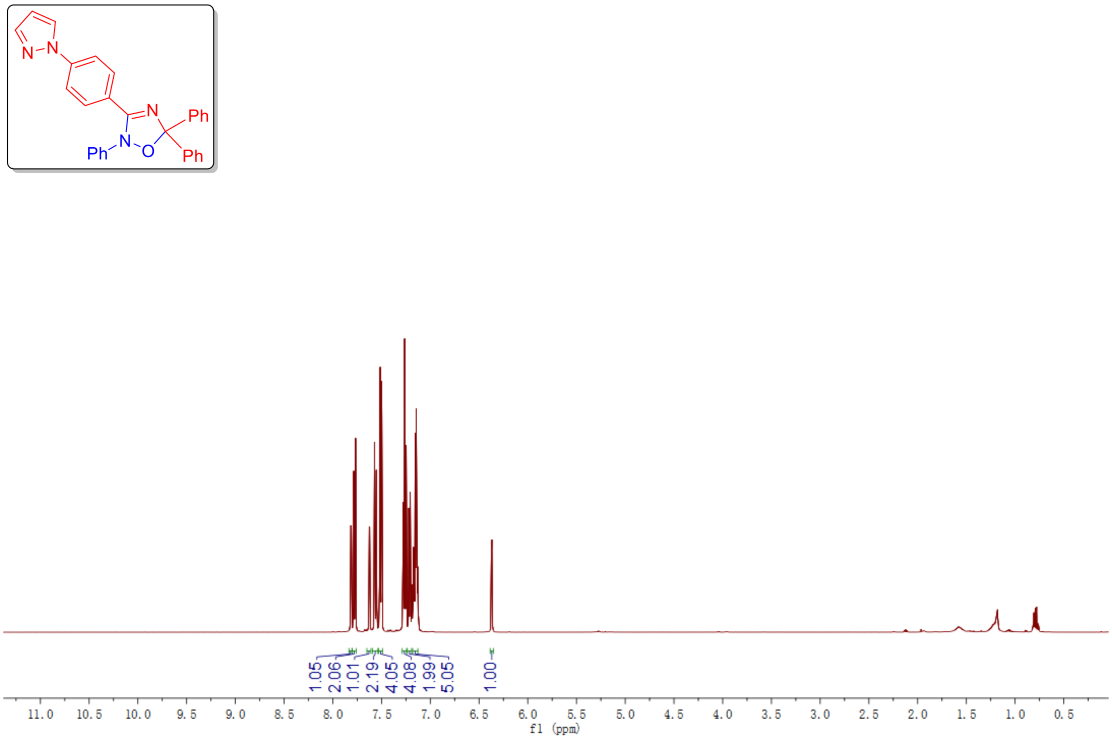

Supplementary Figure 30. ^{13}C NMR Spectrum of 3an (125 MHz, $CDCl_3$)

ZD-Y176

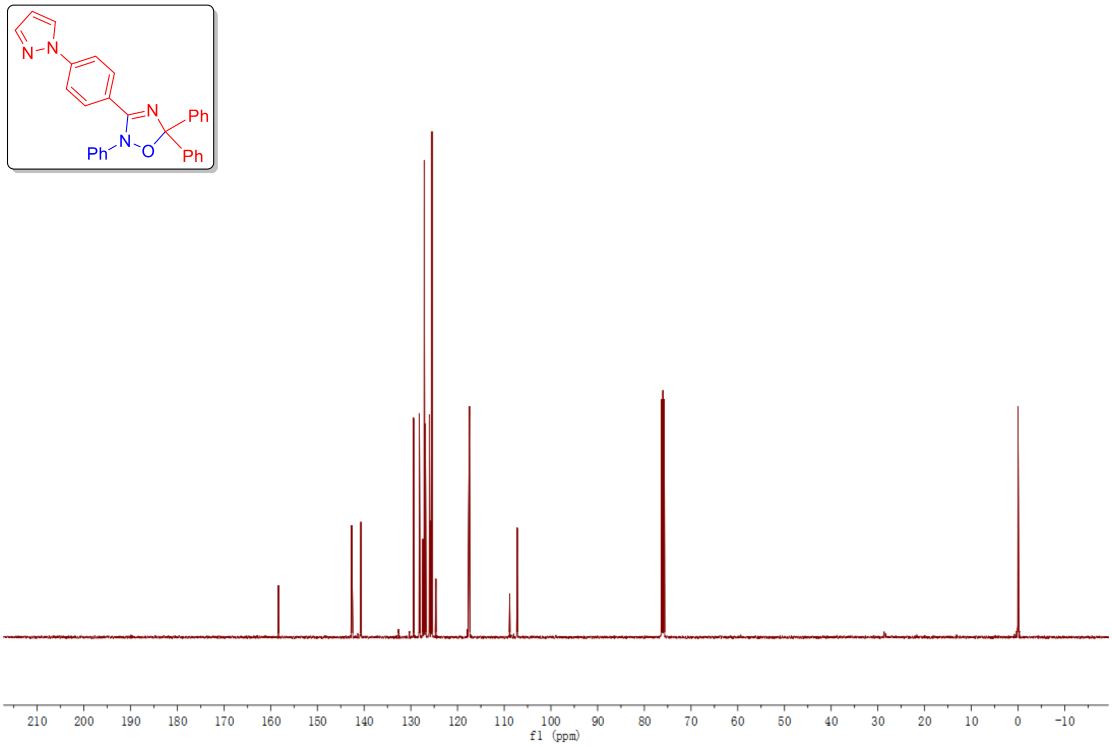

Supplementary Figure 31. [1]**H NMR Spectrum of 3ao (500 MHz, CDCl₃)**

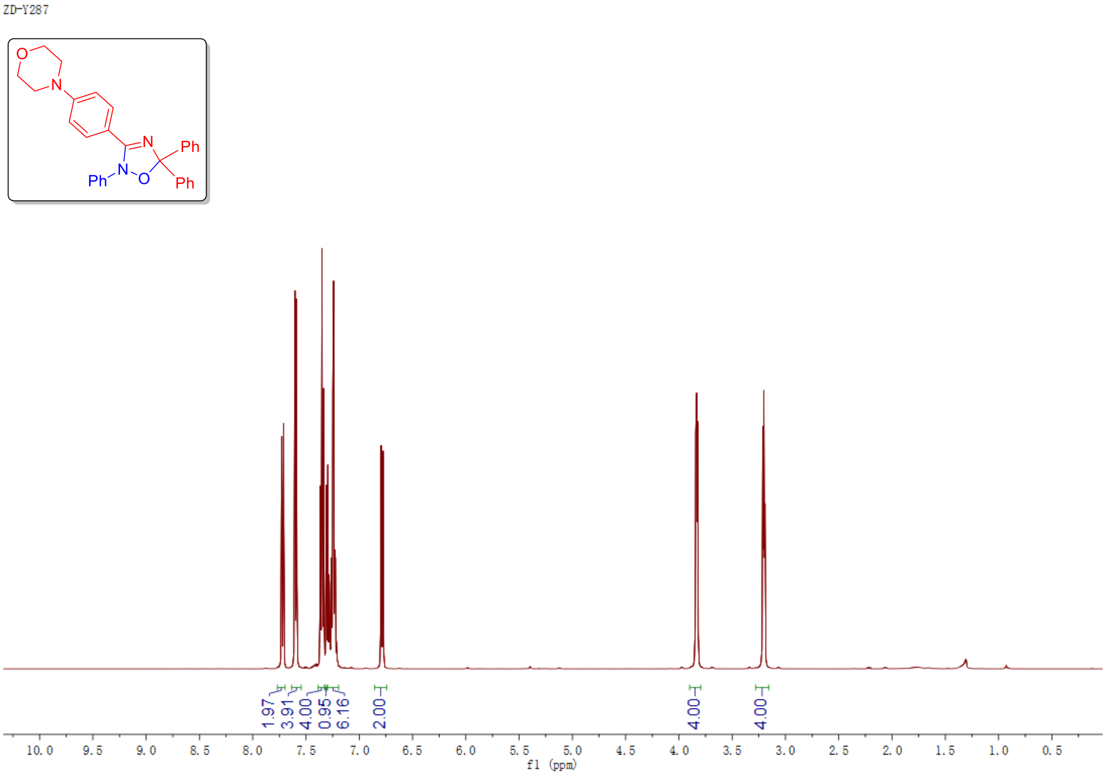

Supplementary Figure 32. [13]**C NMR Spectrum of 3ao (125 MHz, CDCl₃)**

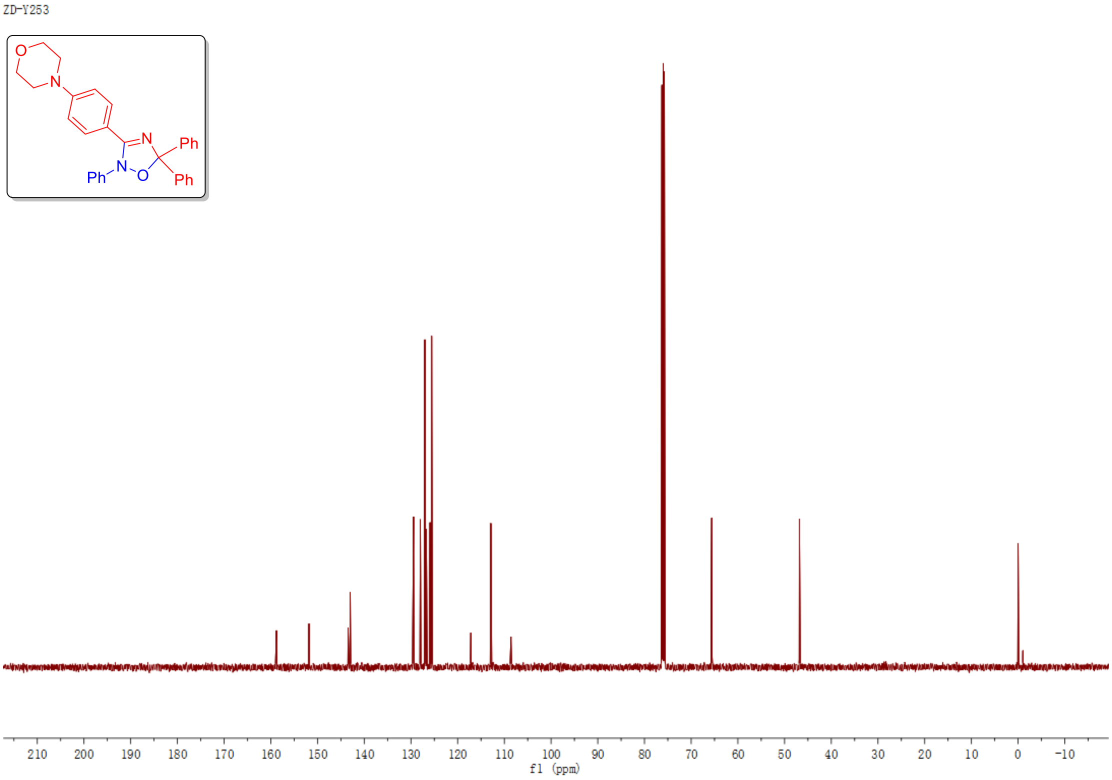

Supplementary Figure 33. ^{1}H NMR Spectrum of 3ap (500 MHz, CDCl₃)

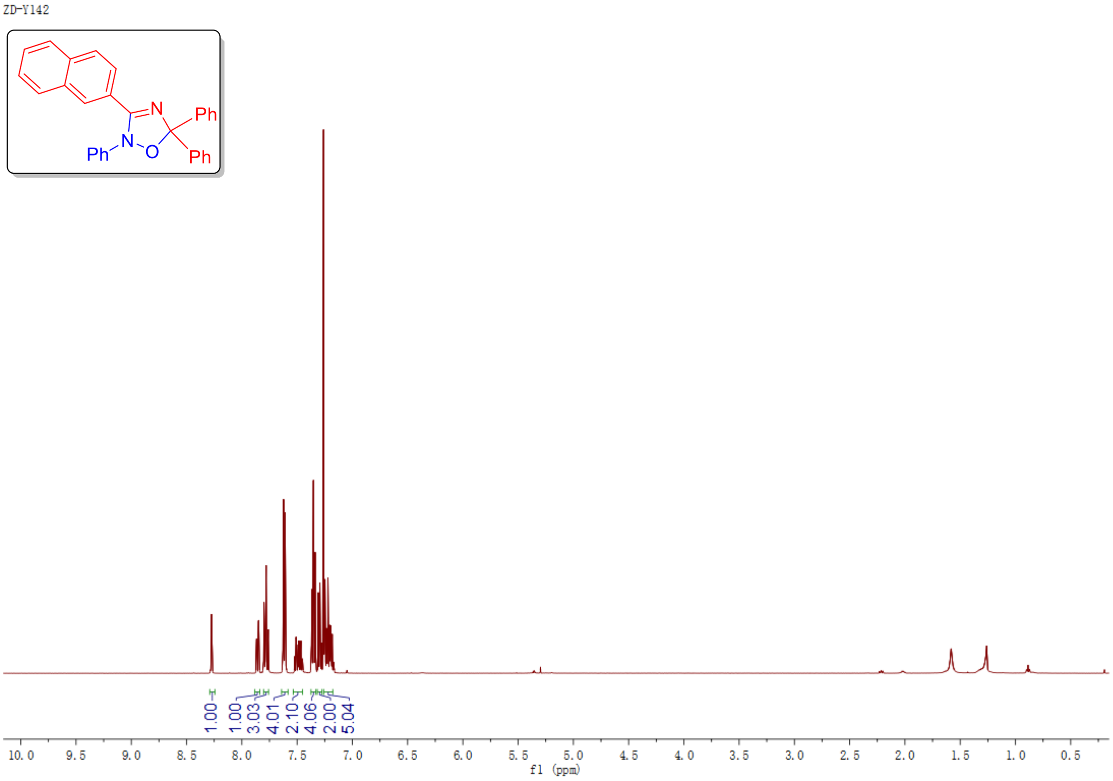

Supplementary Figure 34. ^{13}C NMR Spectrum of 3ap (125 MHz, CDCl₃)

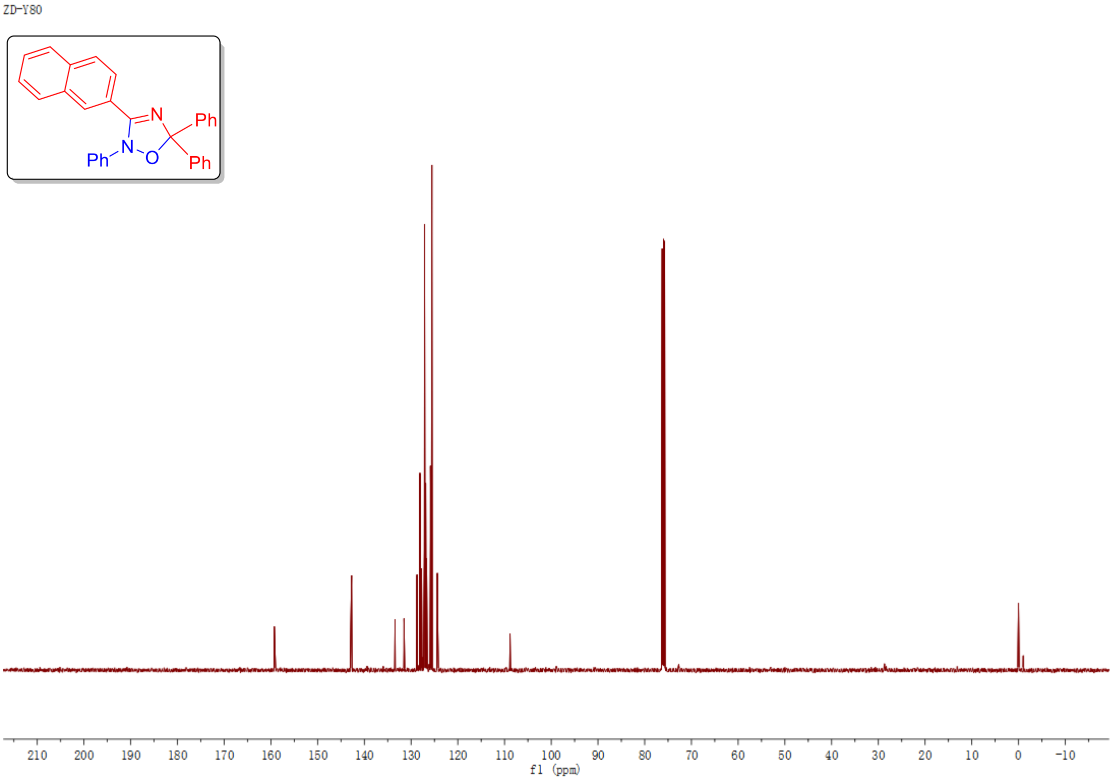

Supplementary Figure 35. ^{1}H NMR Spectrum of 3aq (500 MHz, $CDCl_3$)

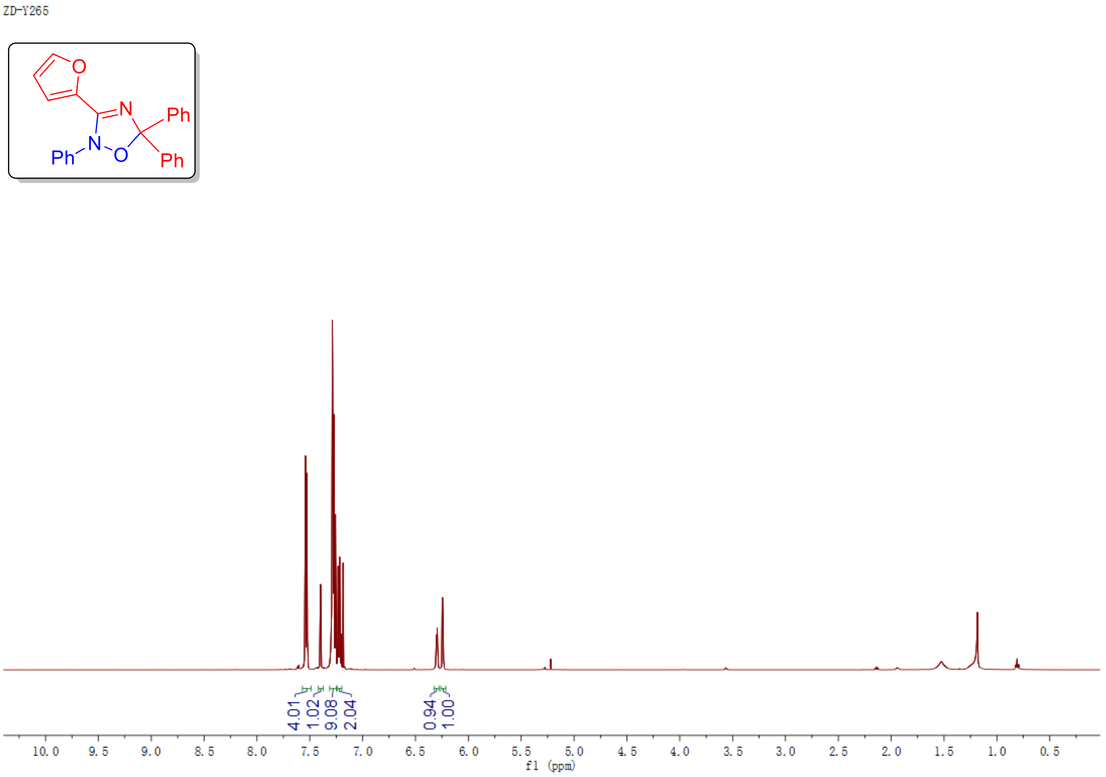

Supplementary Figure 36. ^{13}C NMR Spectrum of 3aq (125 MHz, $CDCl_3$)

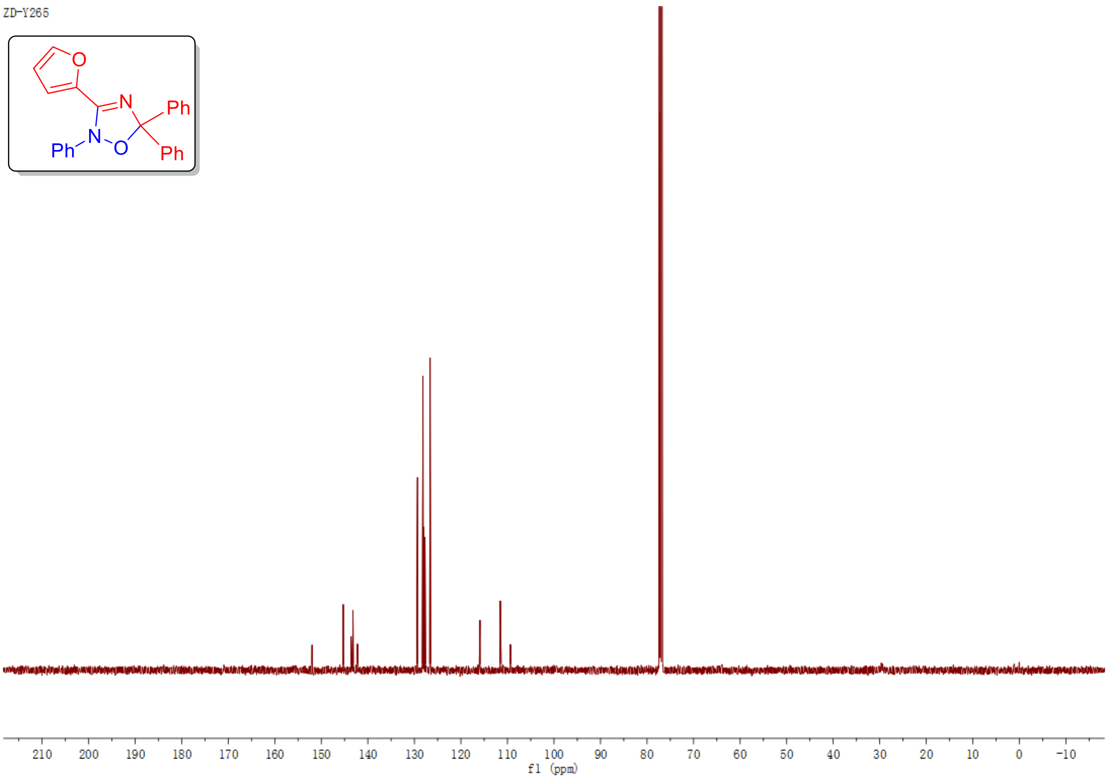

Supplementary Figure 37. ^{1}H NMR Spectrum of 3ar (500 MHz, $CDCl_3$)

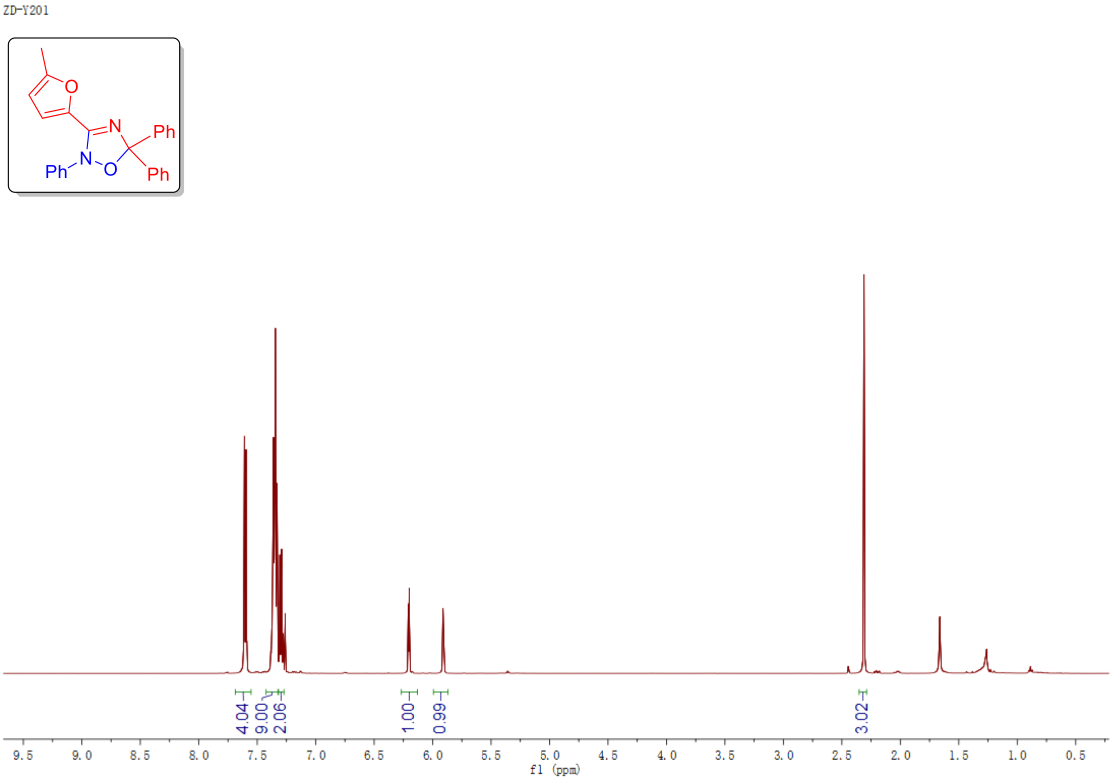

Supplementary Figure 38. ^{13}C NMR Spectrum of 3ar (125 MHz, $CDCl_3$)

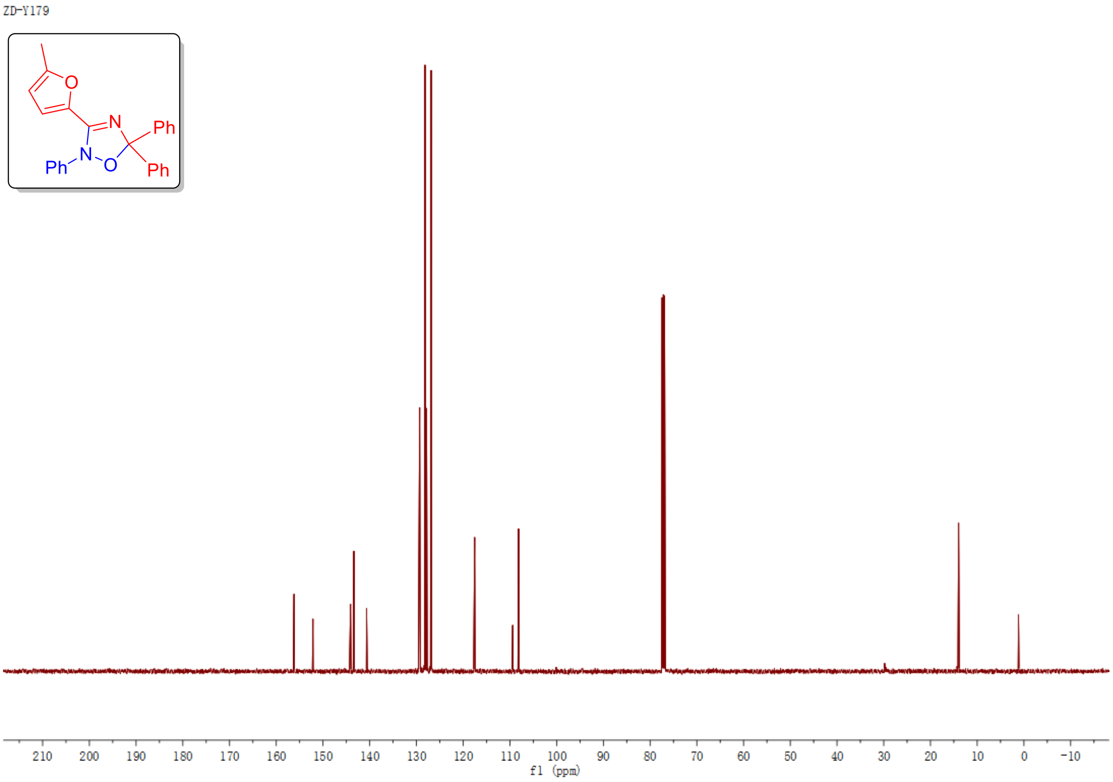

Supplementary Figure 39. ^{1}**H NMR Spectrum of 3as (500 MHz, CDCl$_3$)**

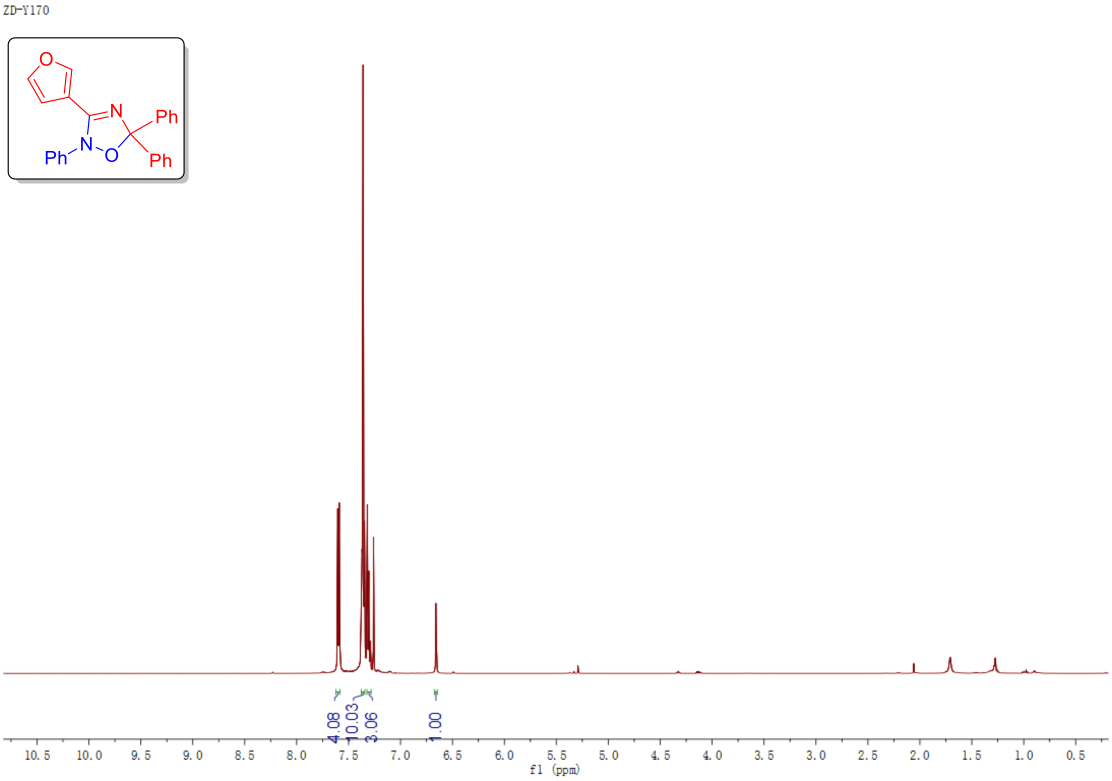

Supplementary Figure 40. ^{13}**C NMR Spectrum of 3as (125 MHz, CDCl$_3$)**

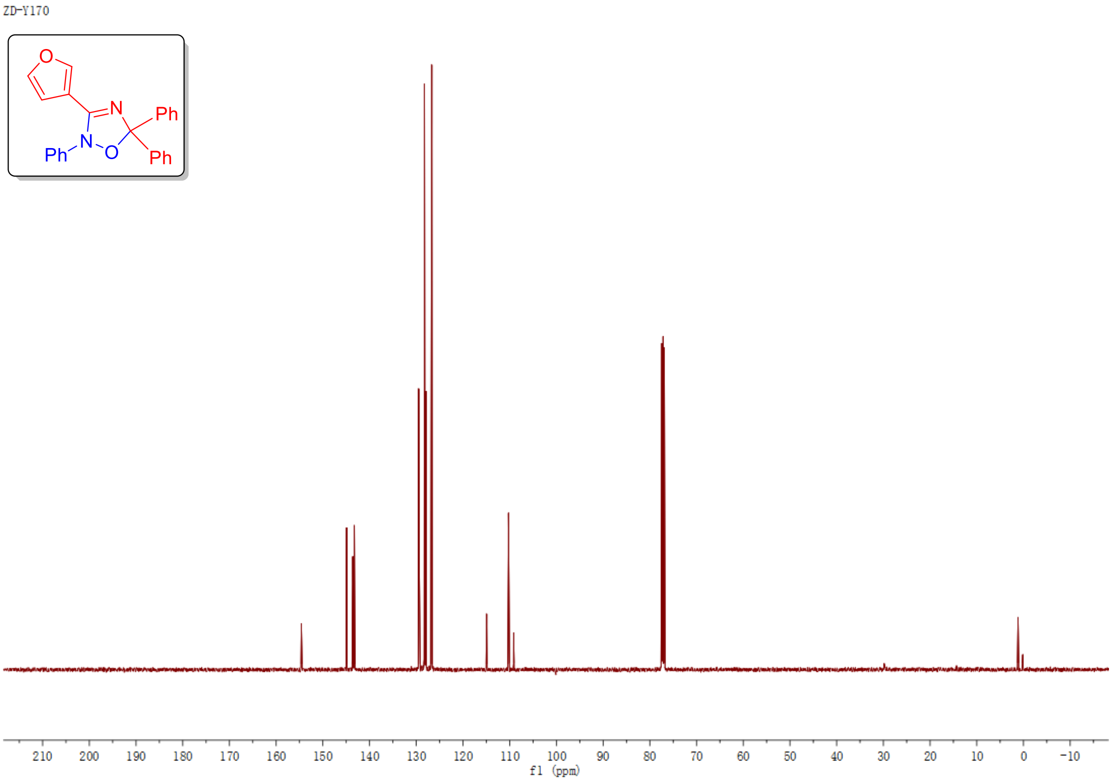

Supplementary Figure 41. ^{1}H NMR Spectrum of 3at (500 MHz, $CDCl_3$)

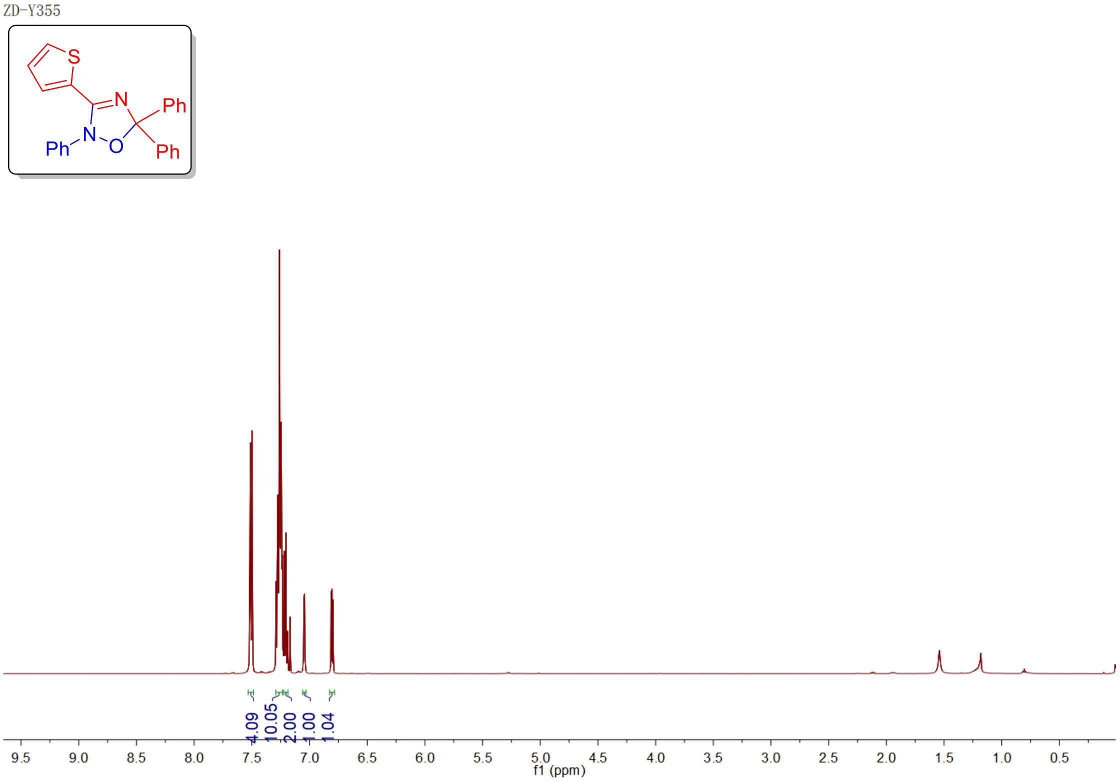

Supplementary Figure 42. ^{13}C NMR Spectrum of 3at (125 MHz, $CDCl_3$)

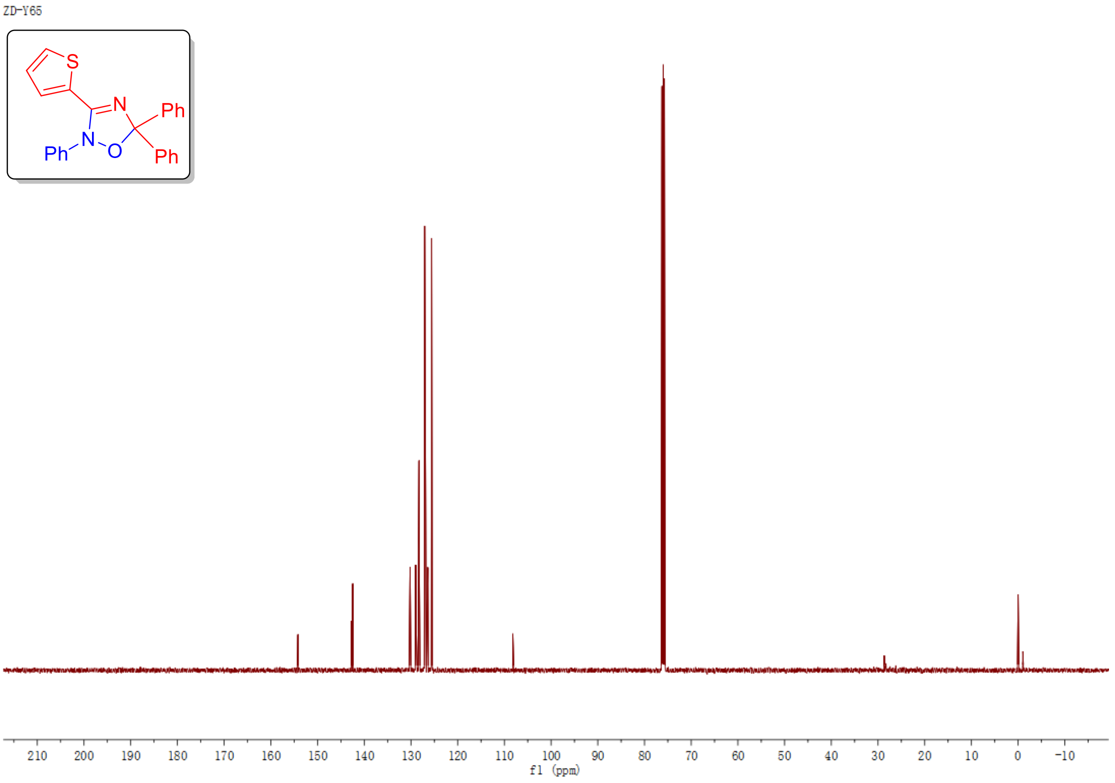

Supplementary Figure 43. ^{1}H NMR Spectrum of 3au (500 MHz, $CDCl_3$)

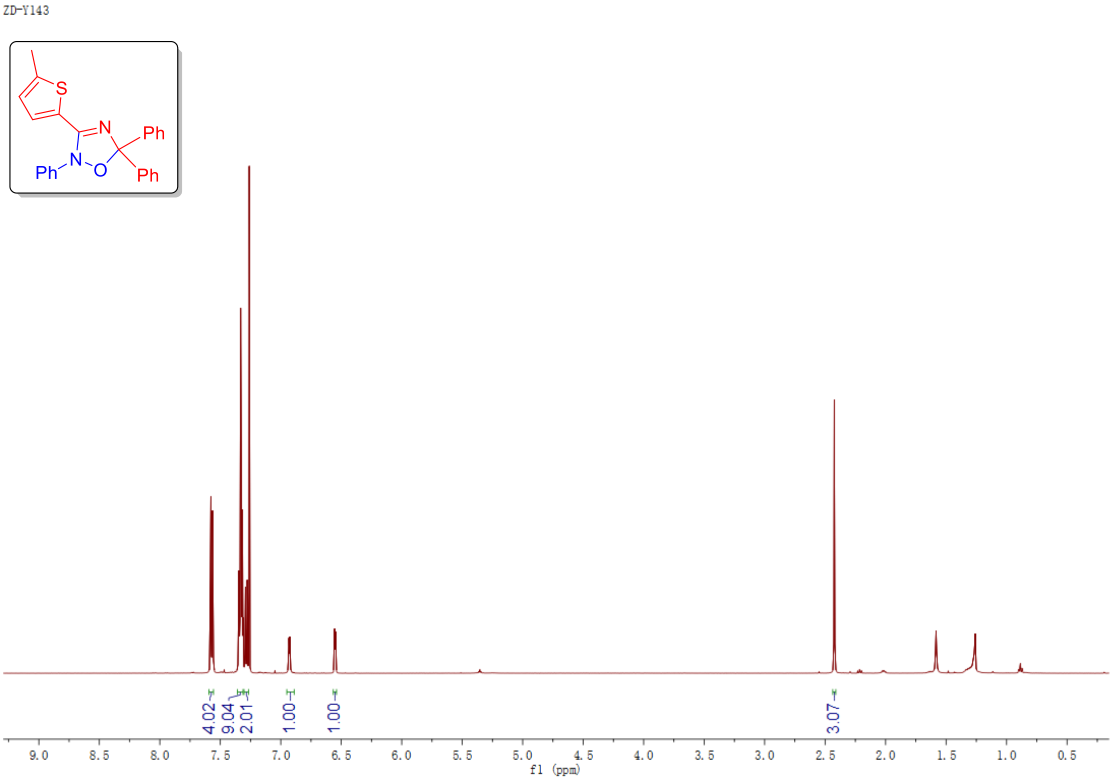

Supplementary Figure 44. ^{13}C NMR Spectrum of 3au (125 MHz, $CDCl_3$)

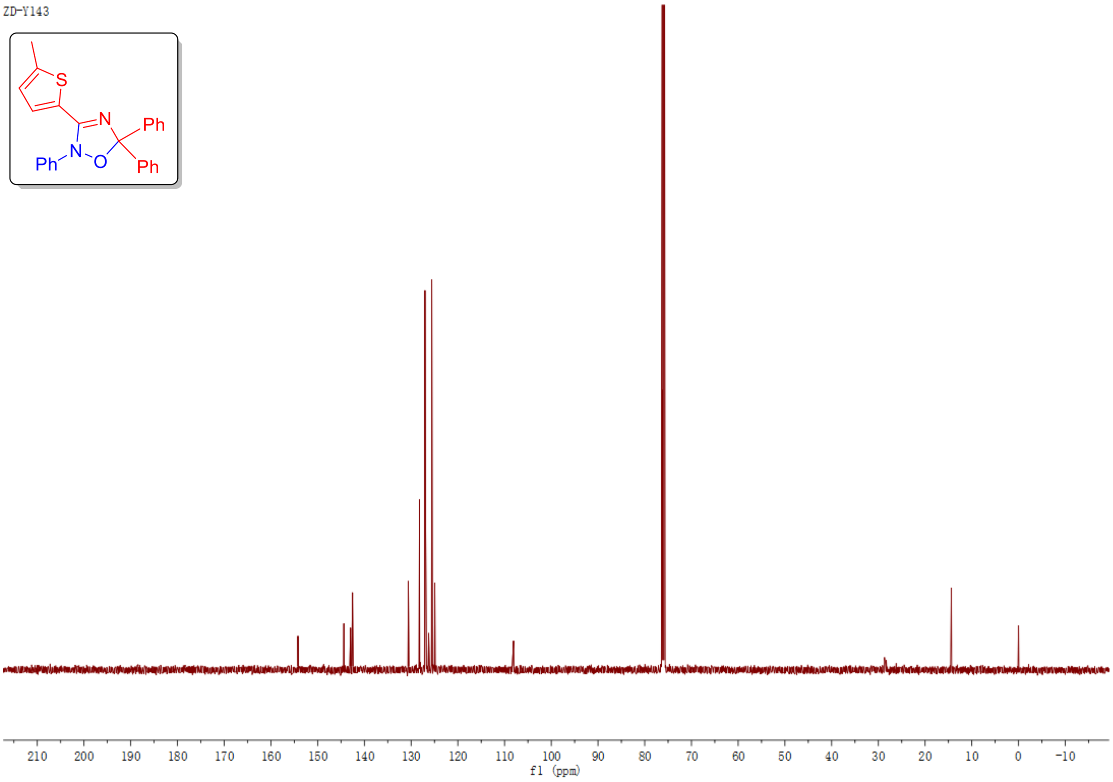

Supplementary Figure 45. ^{1}H NMR Spectrum of 3av (500 MHz, $CDCl_3$)

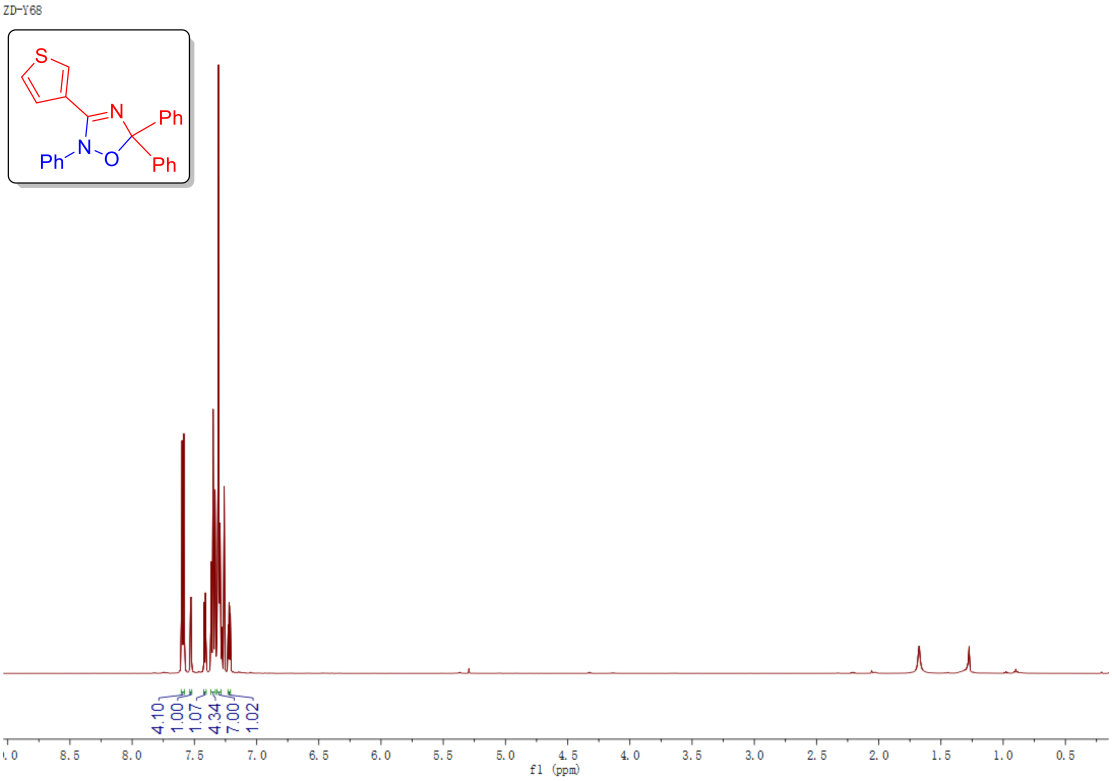

Supplementary Figure 46. ^{13}C NMR Spectrum of 3av (125 MHz, $CDCl_3$)

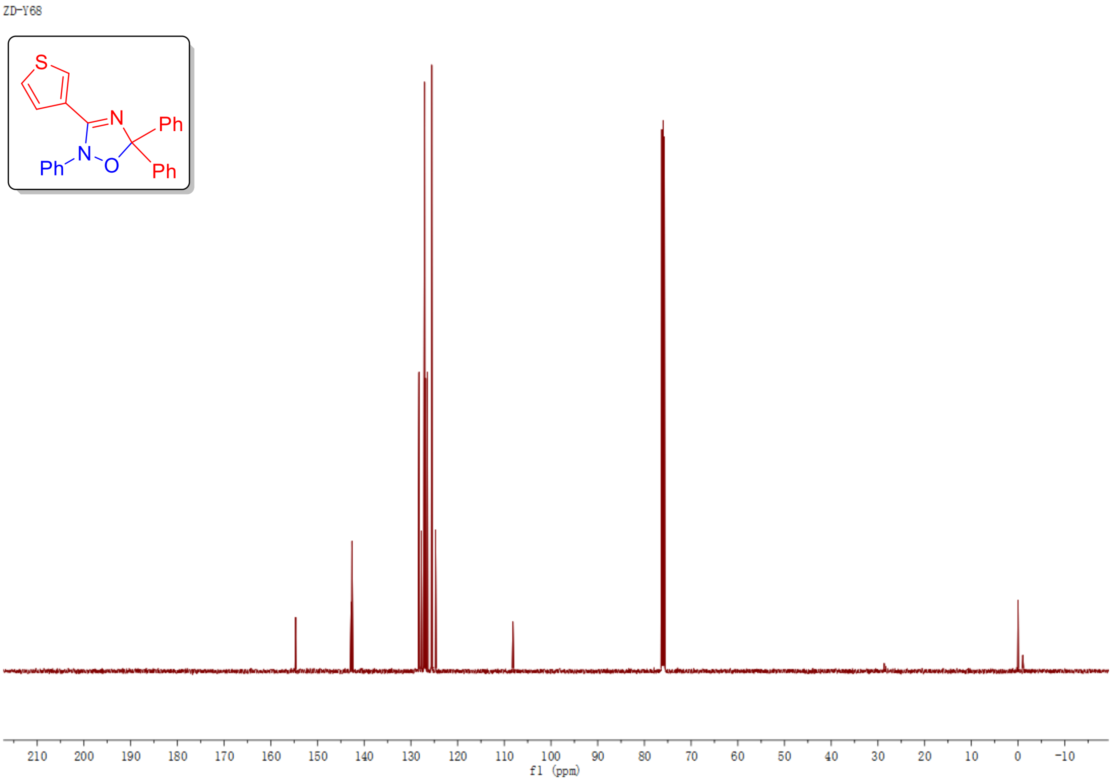

Supplementary Figure 47. ^{1}H NMR Spectrum of 3aa' (500 MHz, $CDCl_3$)

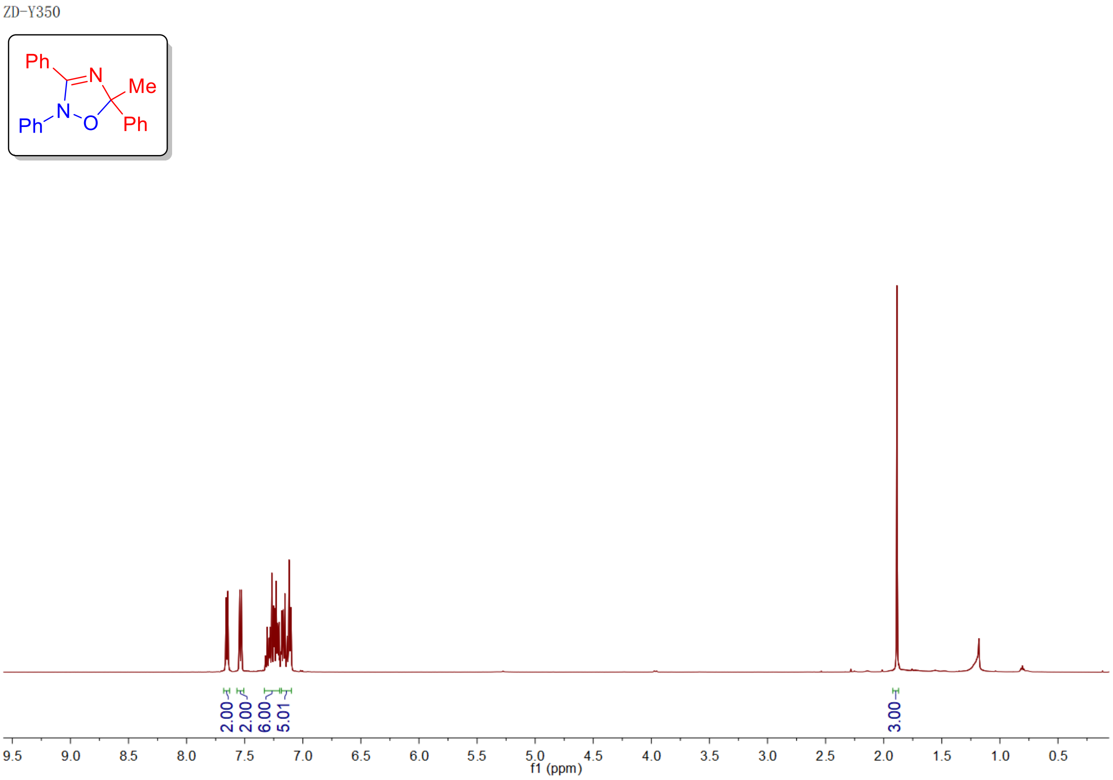

Supplementary Figure 48. ^{13}C NMR Spectrum of 3aa' (125 MHz, $CDCl_3$)

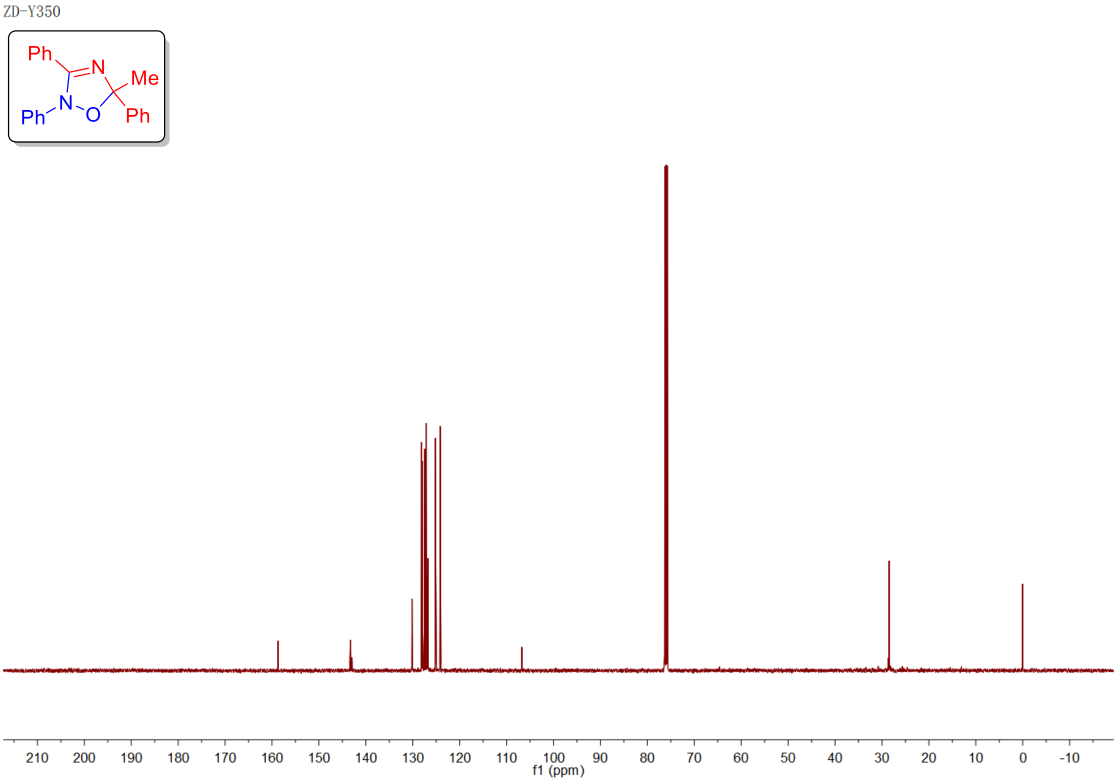

Supplementary Figure 49. ^{1}H NMR Spectrum of 3ba (500 MHz, $CDCl_3$)

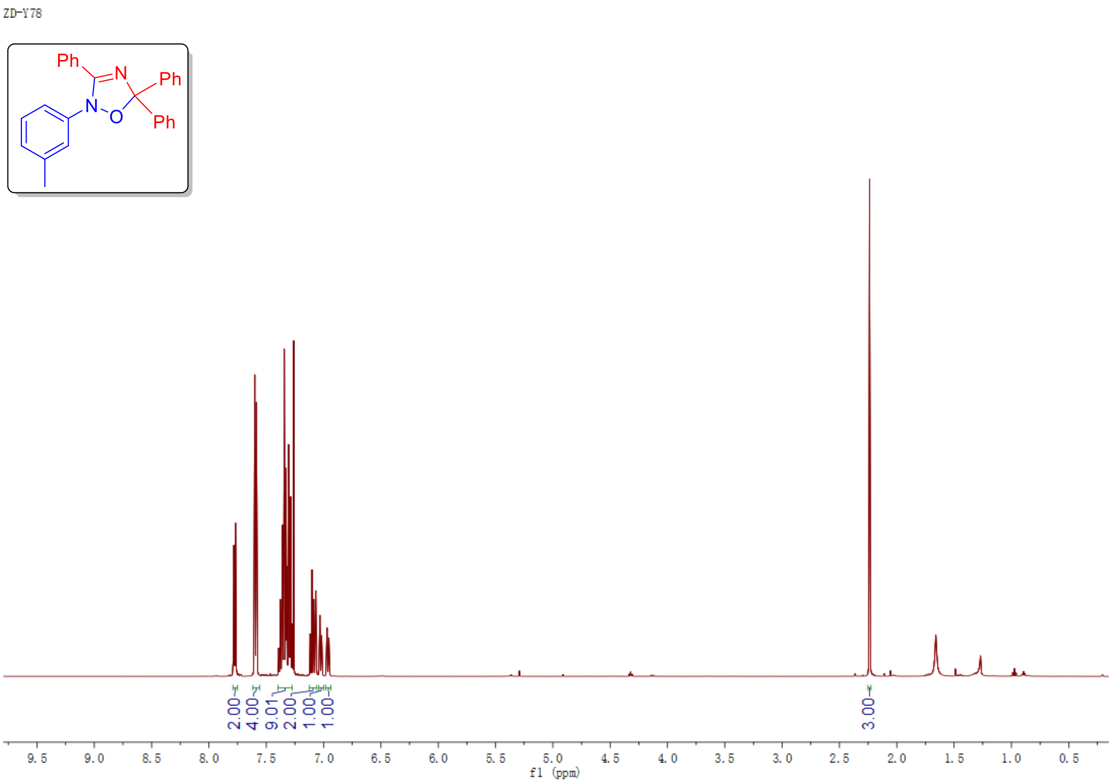

Supplementary Figure 50. ^{13}C NMR Spectrum of 3ba (125 MHz, $CDCl_3$)

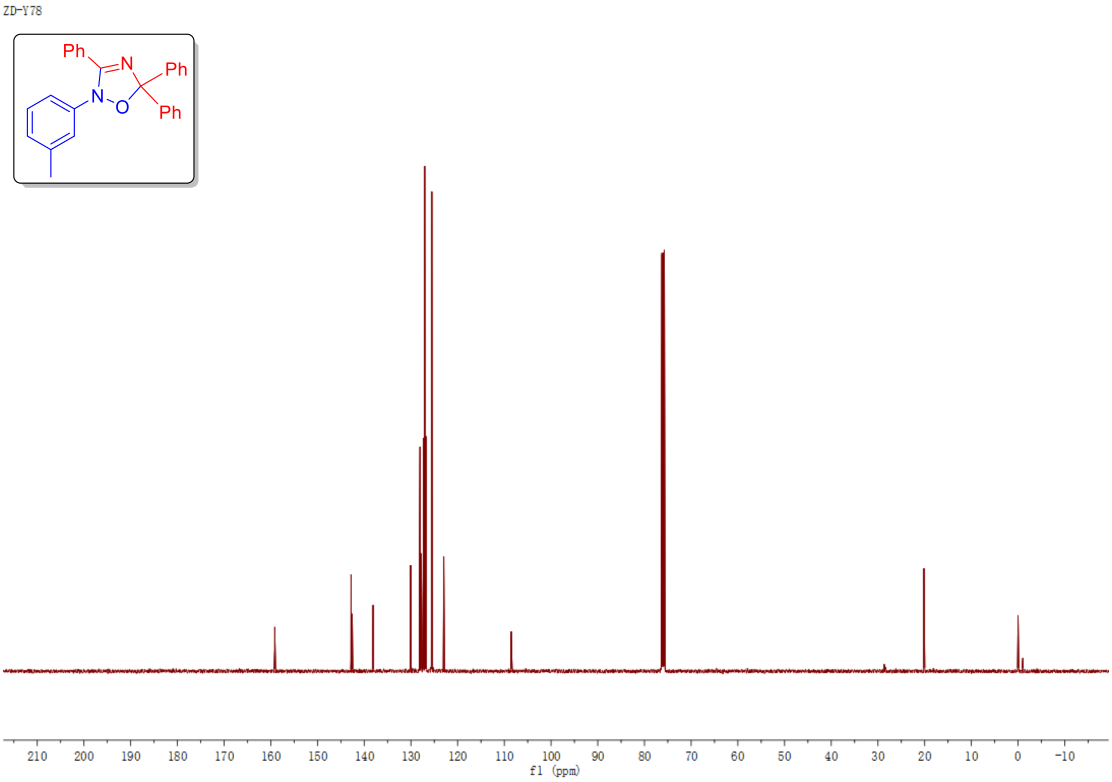

Supplementary Figure 51. ^{1}H NMR Spectrum of 3ca (500 MHz, $CDCl_3$)

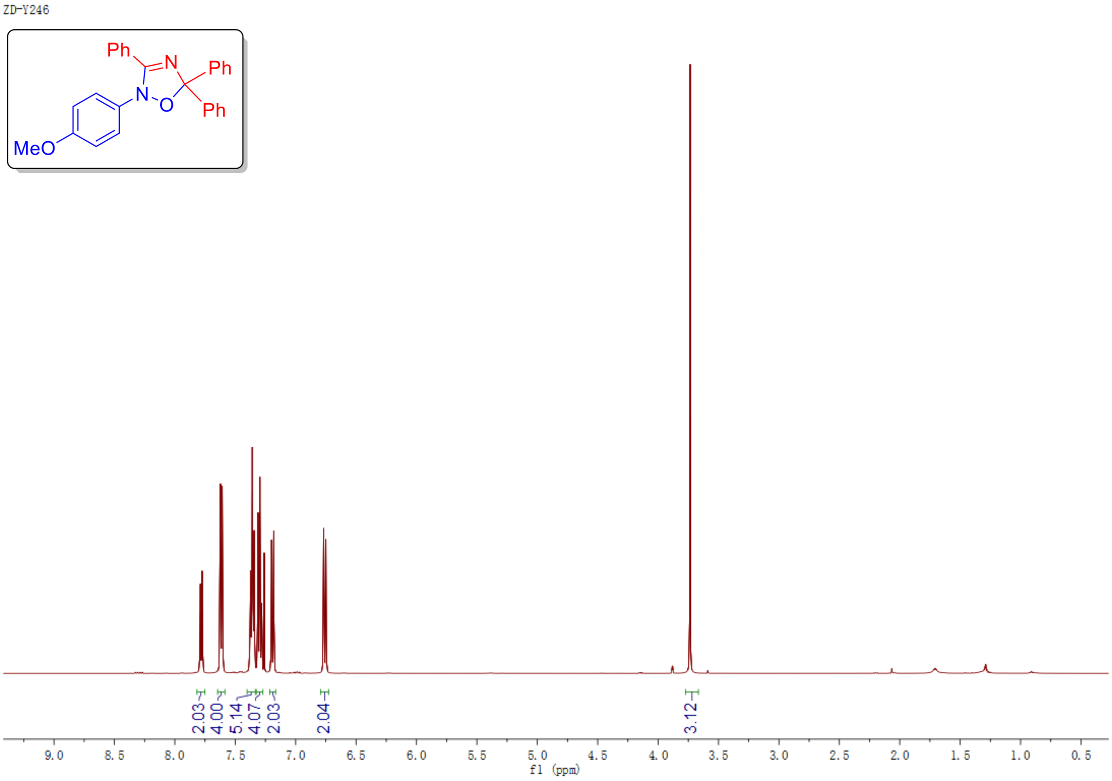

Supplementary Figure 52. ^{13}C NMR Spectrum of 3ca (125 MHz, $CDCl_3$)

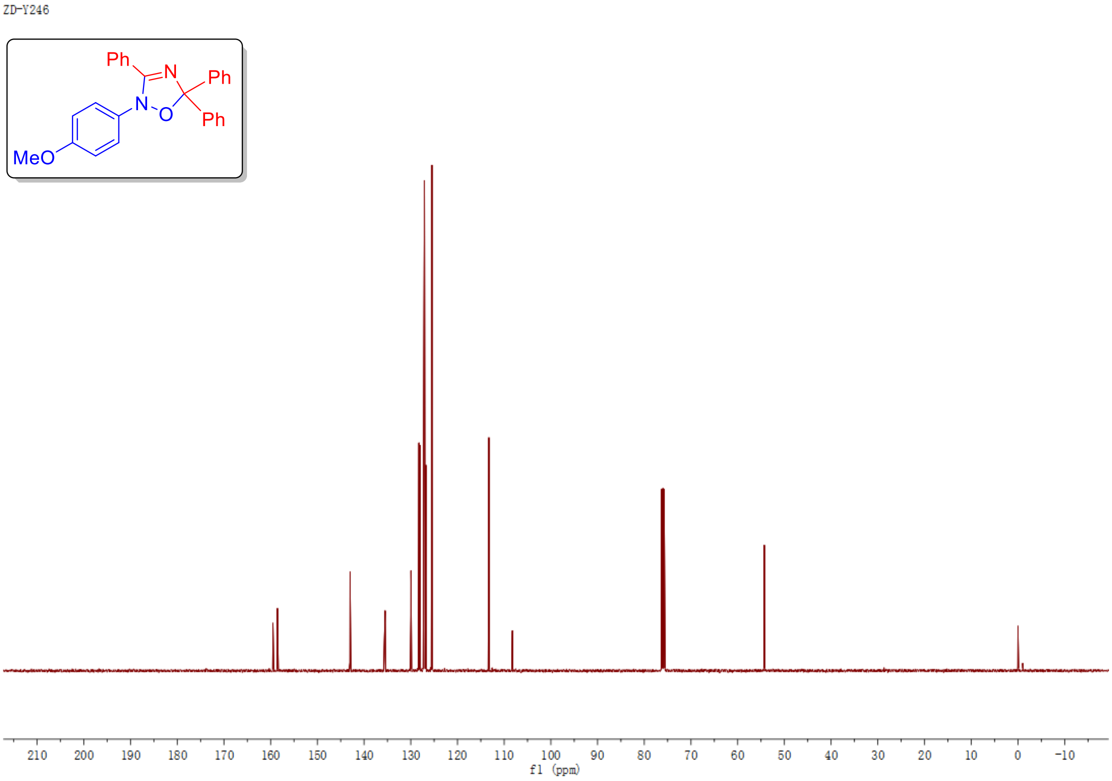

Supplementary Figure 53. ^{1}H NMR Spectrum of 3da (500 MHz, CDCl$_3$)

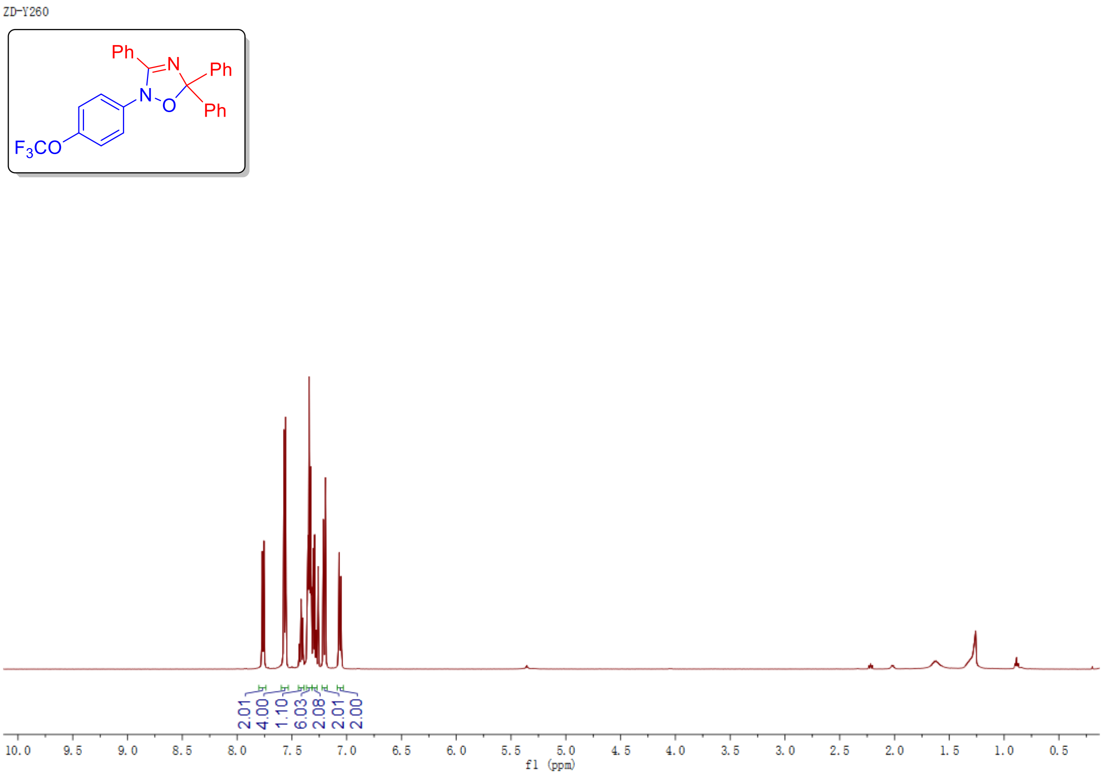

Supplementary Figure 54. ^{13}C NMR Spectrum of 3da (125 MHz, CDCl$_3$)

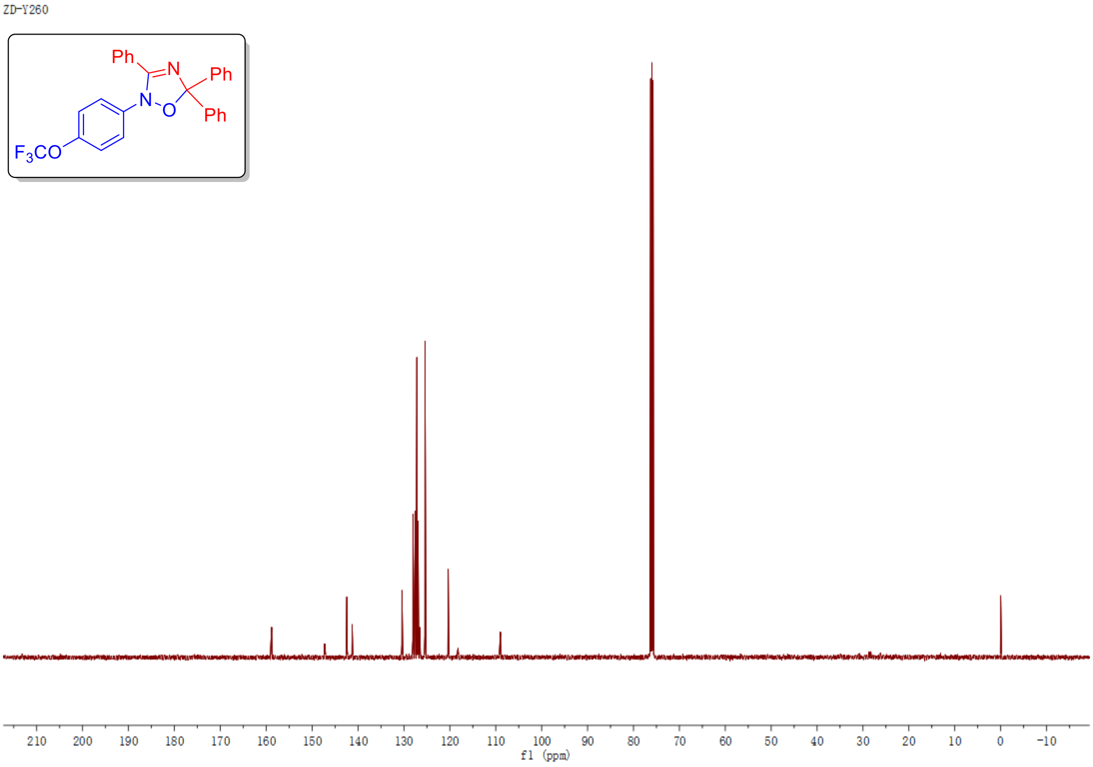

Supplementary Figure 55. ^{1}H NMR Spectrum of 3ea (500 MHz, $CDCl_3$)

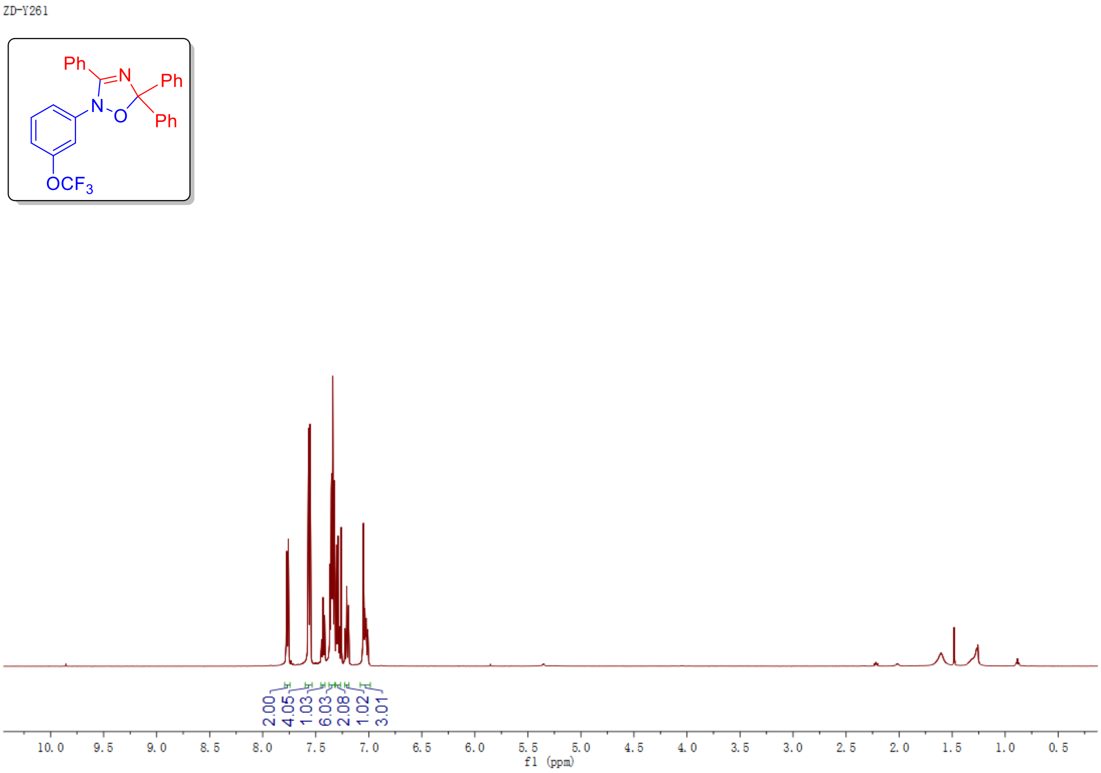

Supplementary Figure 56. ^{13}C NMR Spectrum of 3ea (125 MHz, $CDCl_3$)

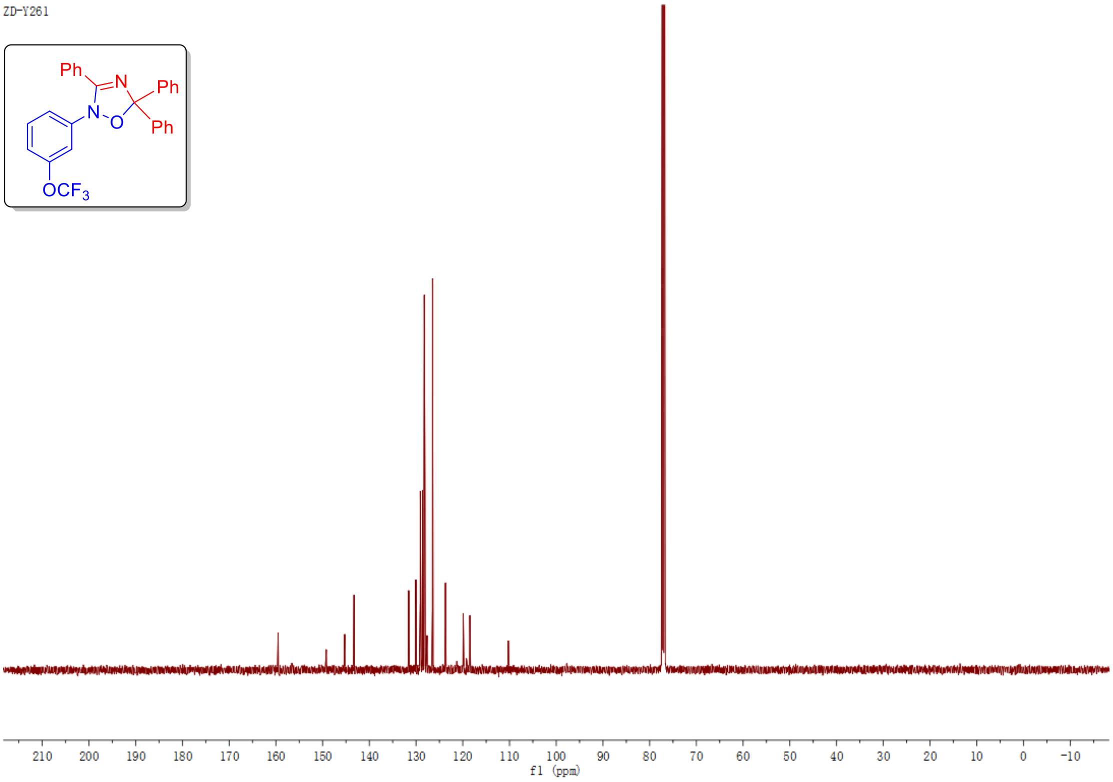

Supplementary Figure 57. ^{1}H NMR Spectrum of 3fa (500 MHz, $CDCl_3$)

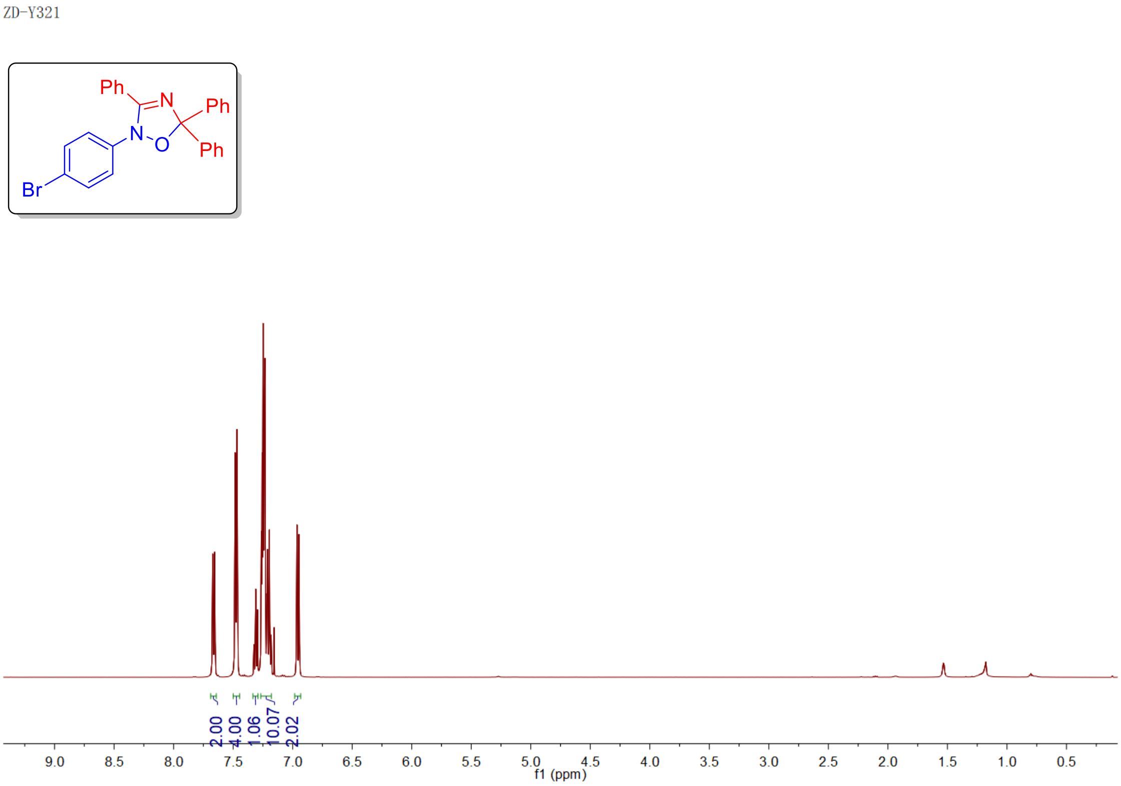

Supplementary Figure 58. ^{13}C NMR Spectrum of 3fa (125 MHz, $CDCl_3$)

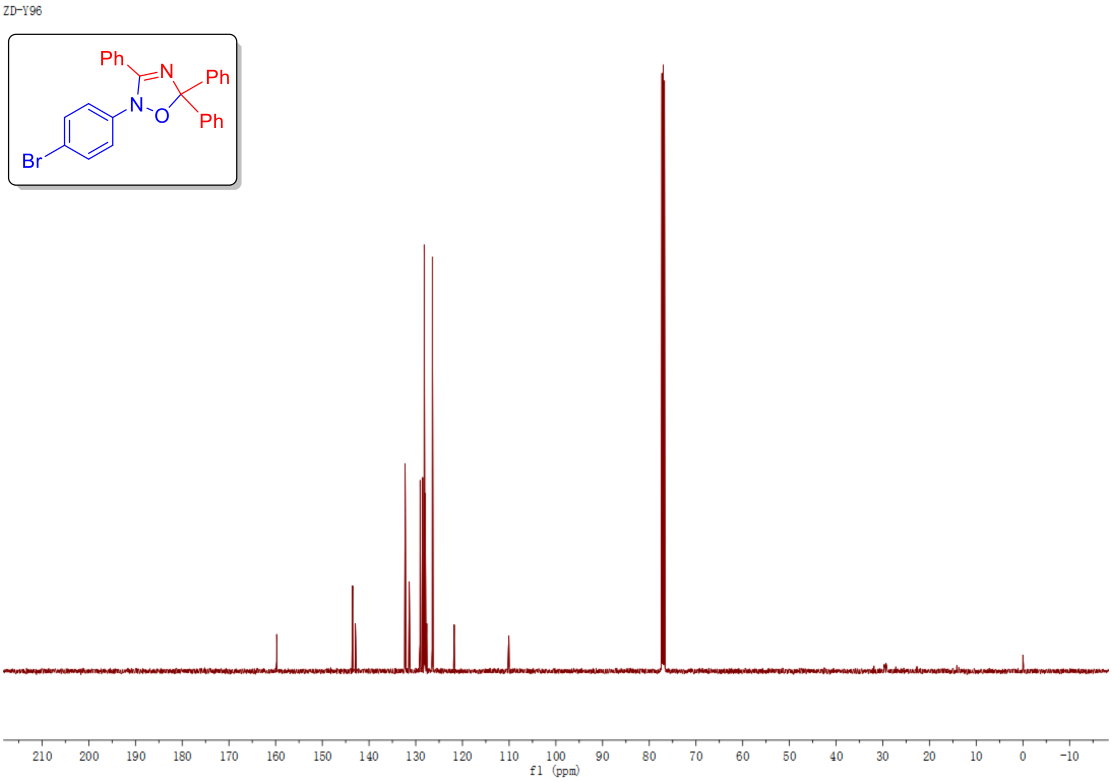

Supplementary Figure 59. ^{1}H NMR Spectrum of 3ga (500 MHz, $CDCl_3$)

ZD-Y329

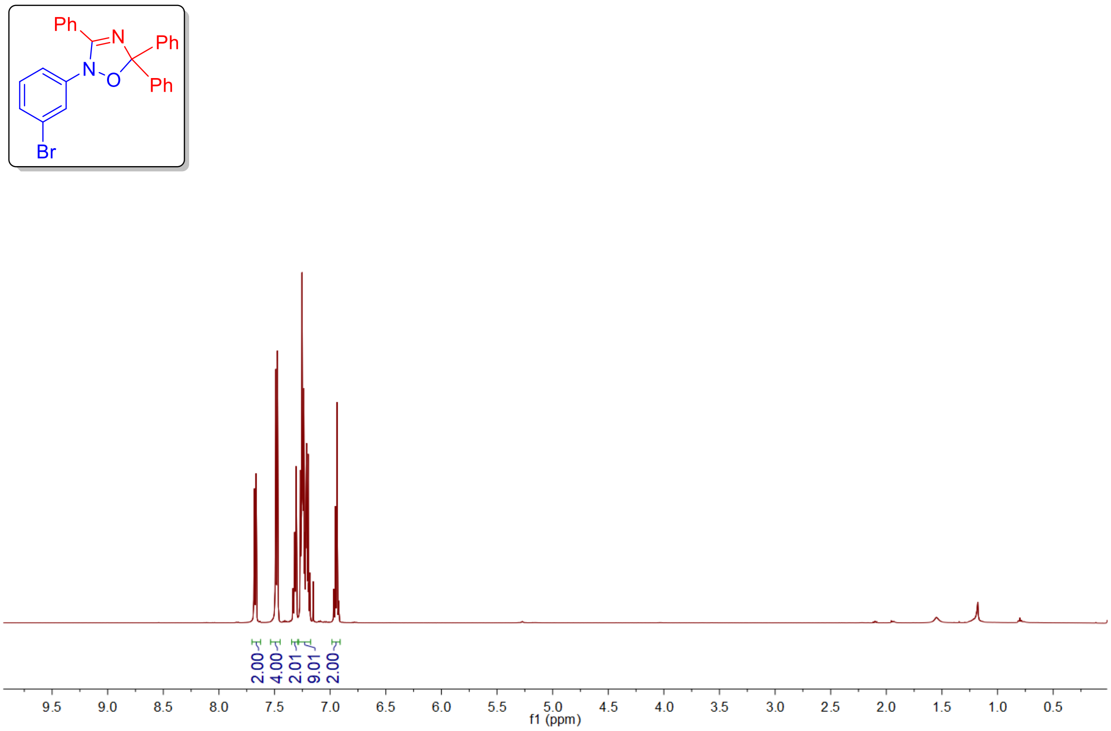

Supplementary Figure 60. ^{13}C NMR Spectrum of 3ga (125 MHz, $CDCl_3$)

ZD-Y75

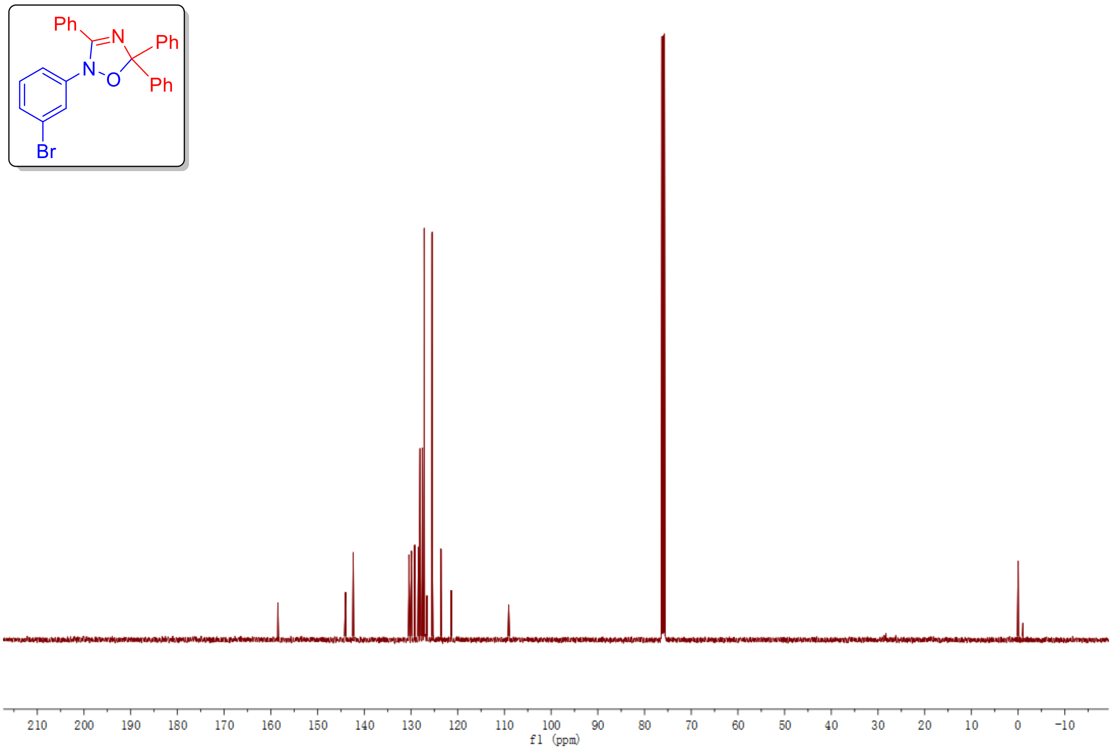

Supplementary Figure 61. ^{1}**H NMR Spectrum of 3ha (500 MHz,** $CDCl_3$**)**

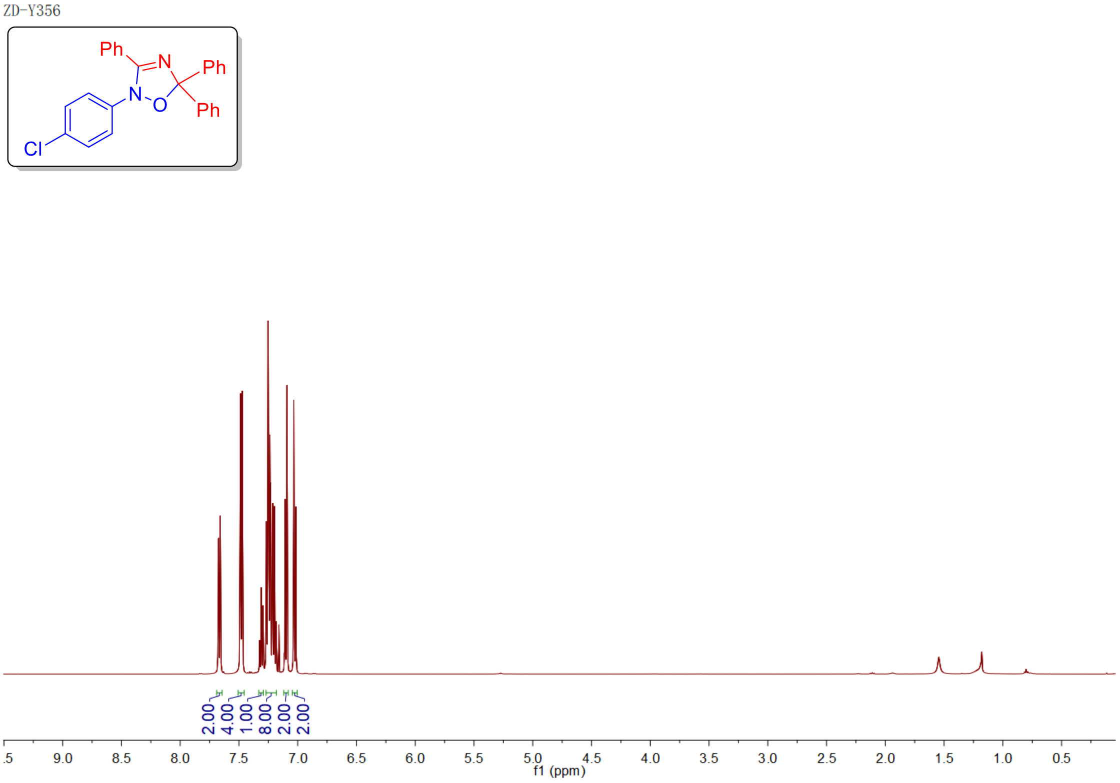

Supplementary Figure 62. ^{13}**C NMR Spectrum of 3ha (125 MHz,** $CDCl_3$**)**

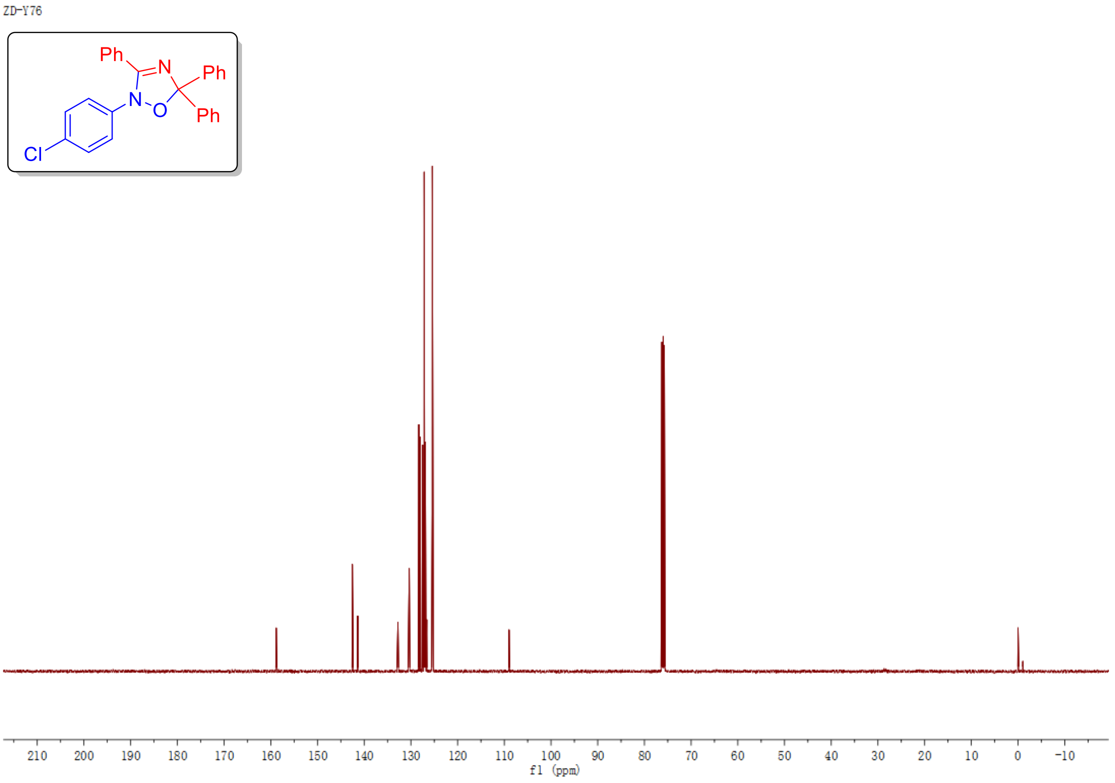

Supplementary Figure 63. ^{1}H NMR Spectrum of 3ia (500 MHz, $CDCl_3$)

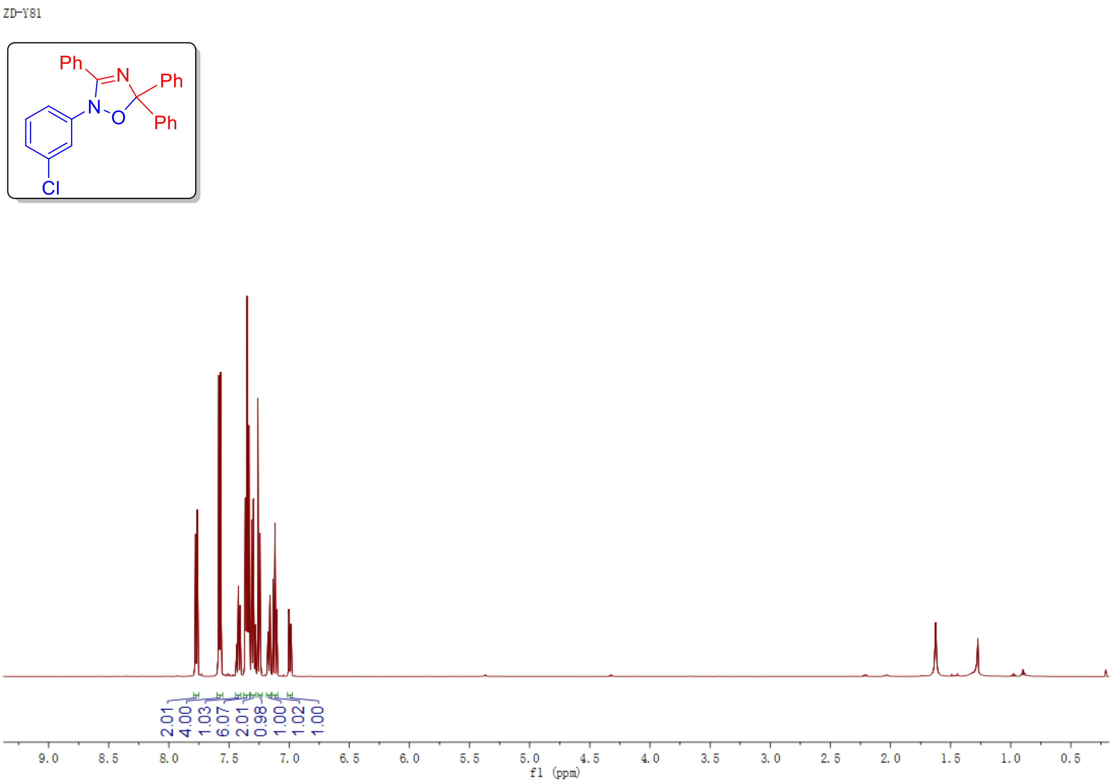

Supplementary Figure 64. ^{13}C NMR Spectrum of 3ia (125 MHz, $CDCl_3$)

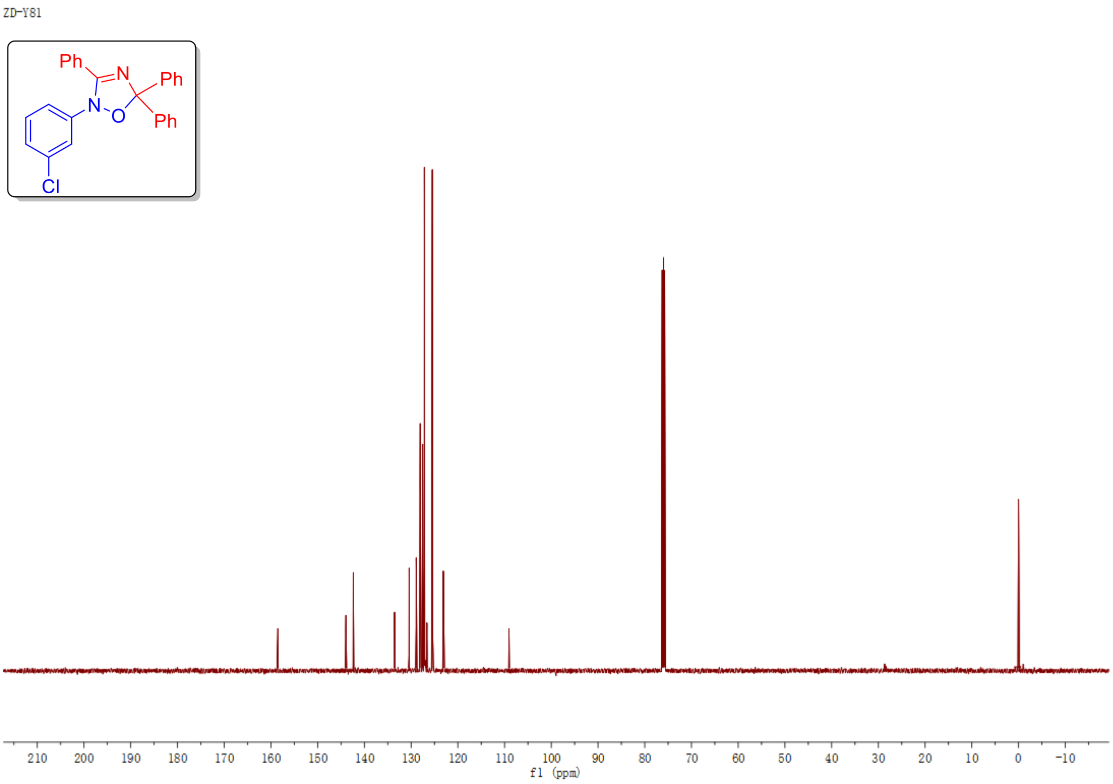

Supplementary Figure 65. ^{1}H NMR Spectrum of 3ja (500 MHz, CDCl₃)

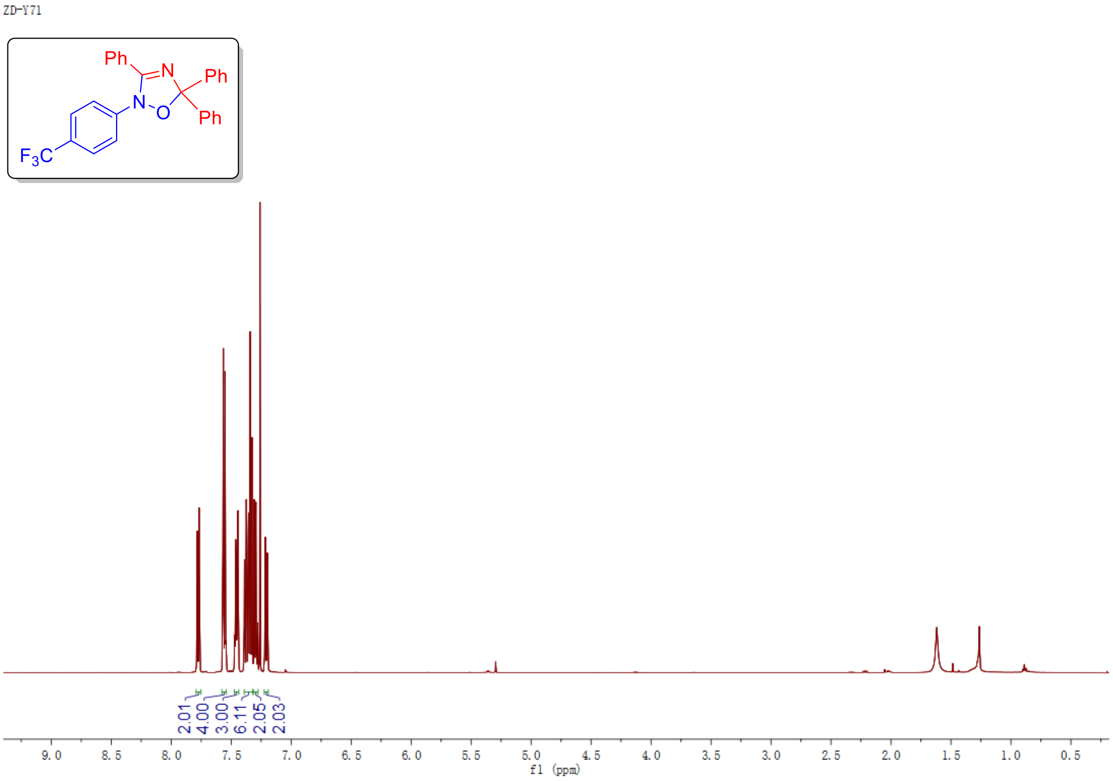

Supplementary Figure 66. ^{13}C NMR Spectrum of 3ja (125 MHz, CDCl₃)

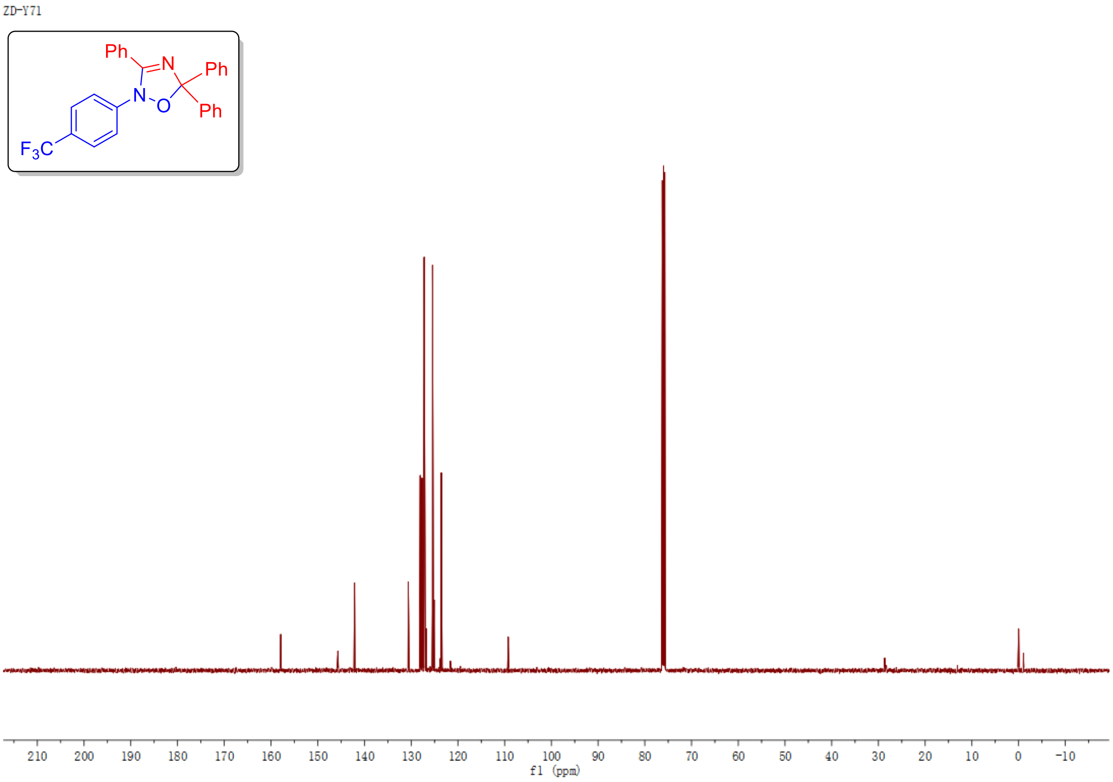

Supplementary Figure 67. ^{1}H NMR Spectrum of 3ka (500 MHz, $CDCl_3$)

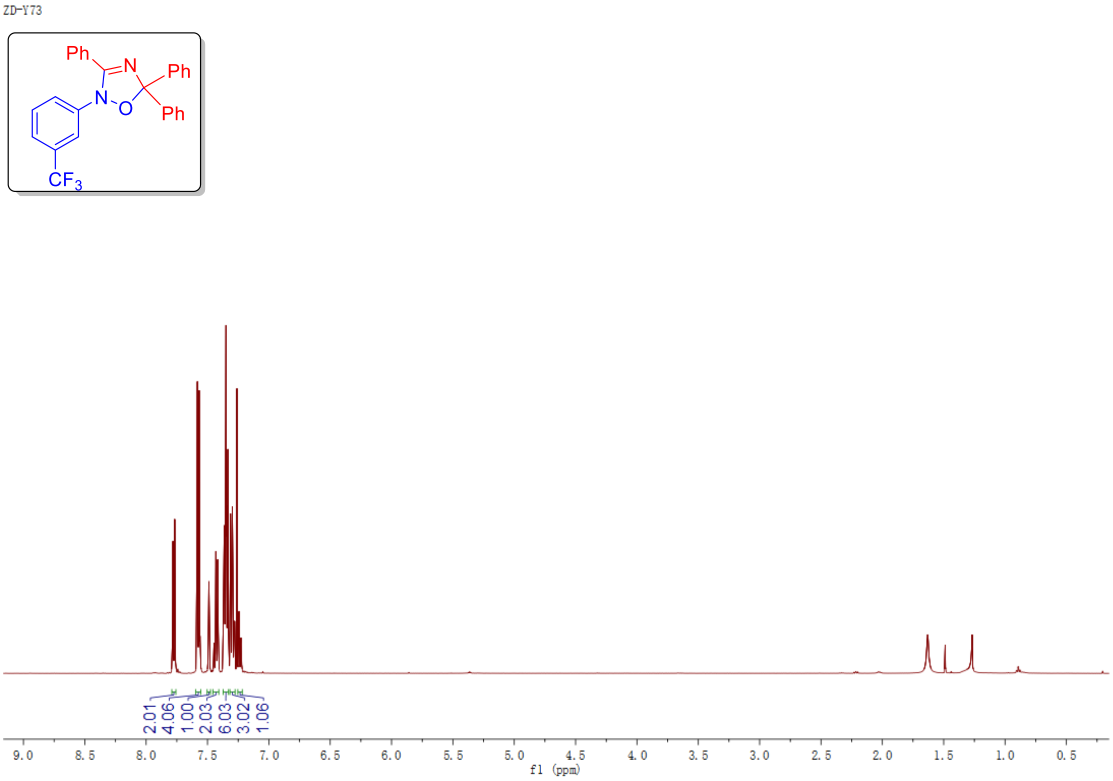

Supplementary Figure 68. ^{13}C NMR Spectrum of 3ka (125 MHz, $CDCl_3$)

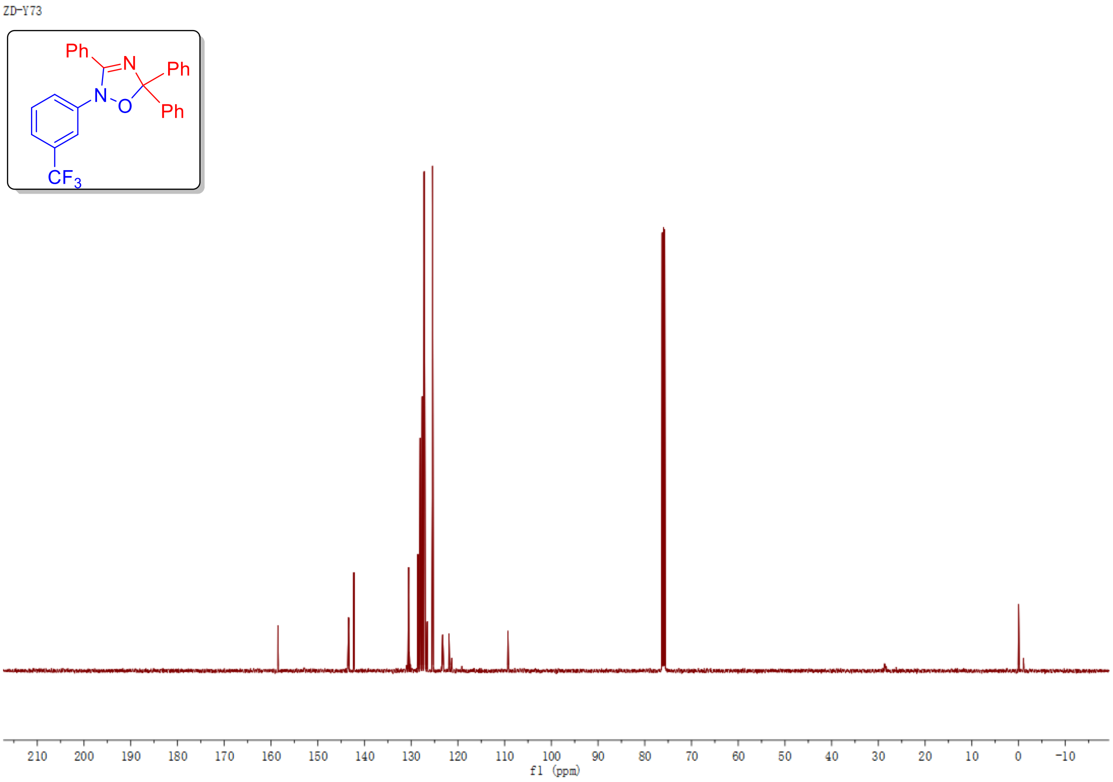

Supplementary Figure 69. ^{1}H NMR Spectrum of 3la (500 MHz, $CDCl_3$)

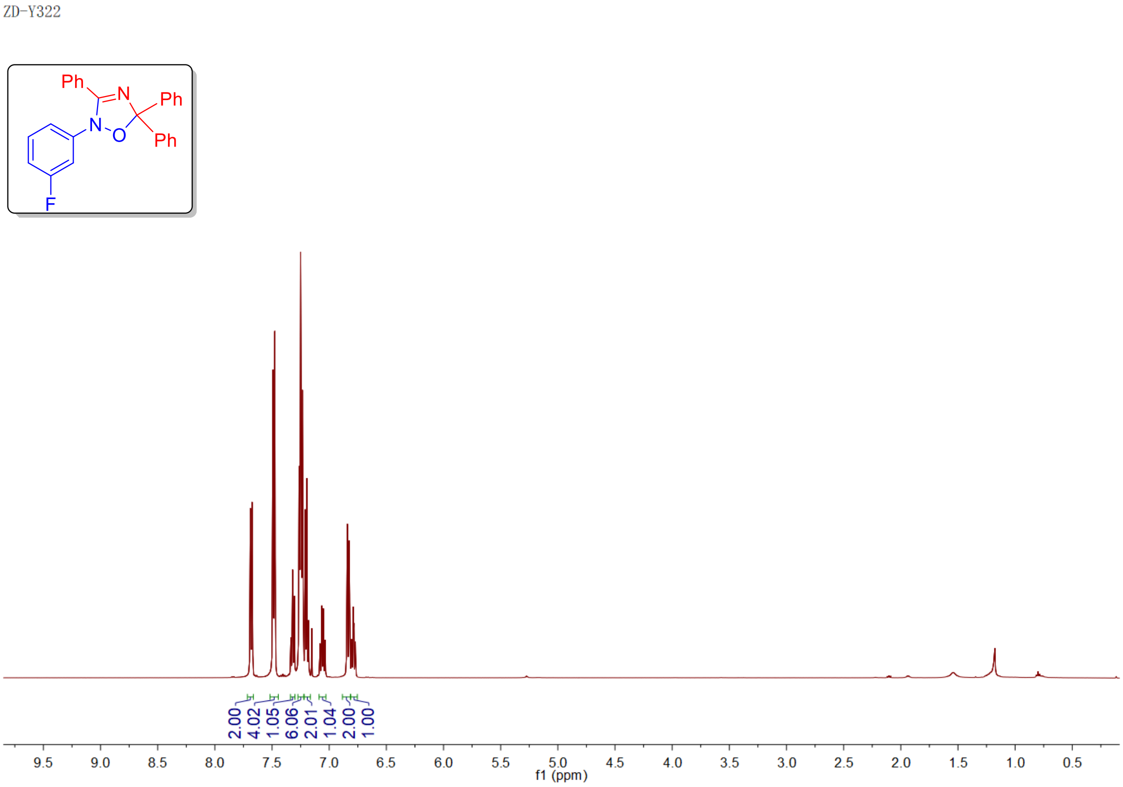

Supplementary Figure 70. ^{13}C NMR Spectrum of 3la (125 MHz, $CDCl_3$)

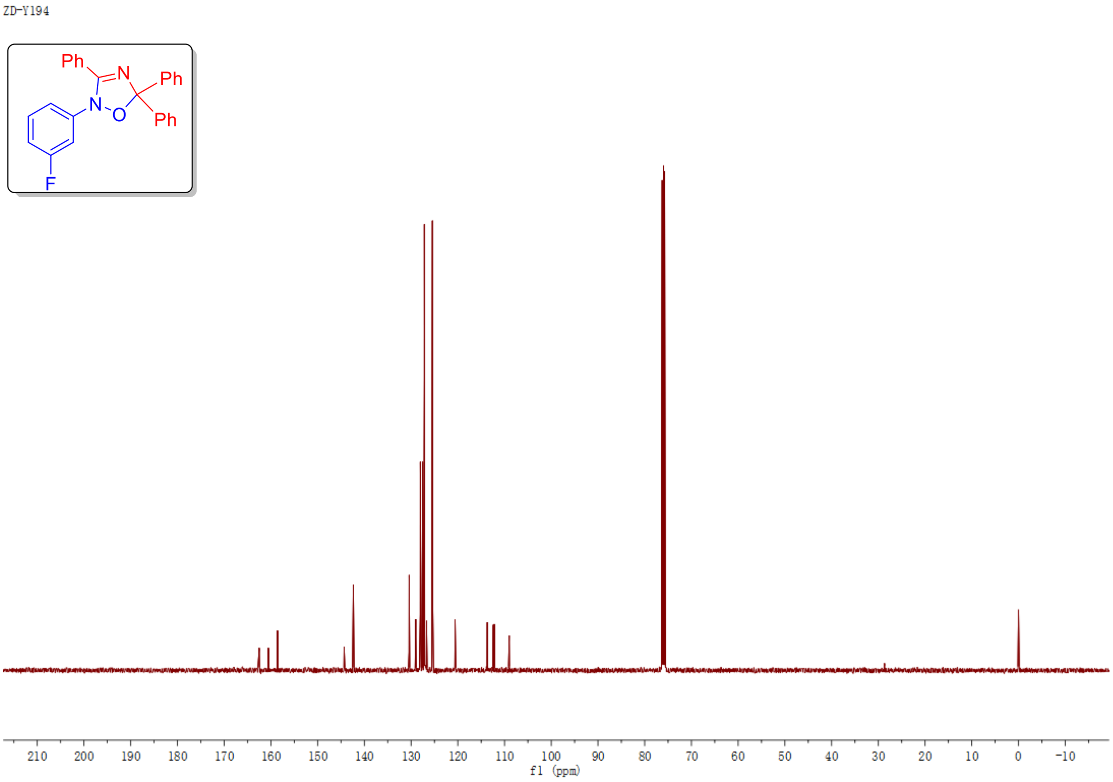

Supplementary Figure 71. ^{1}H **NMR Spectrum of 3ma (500 MHz,** $CDCl_3$**)**

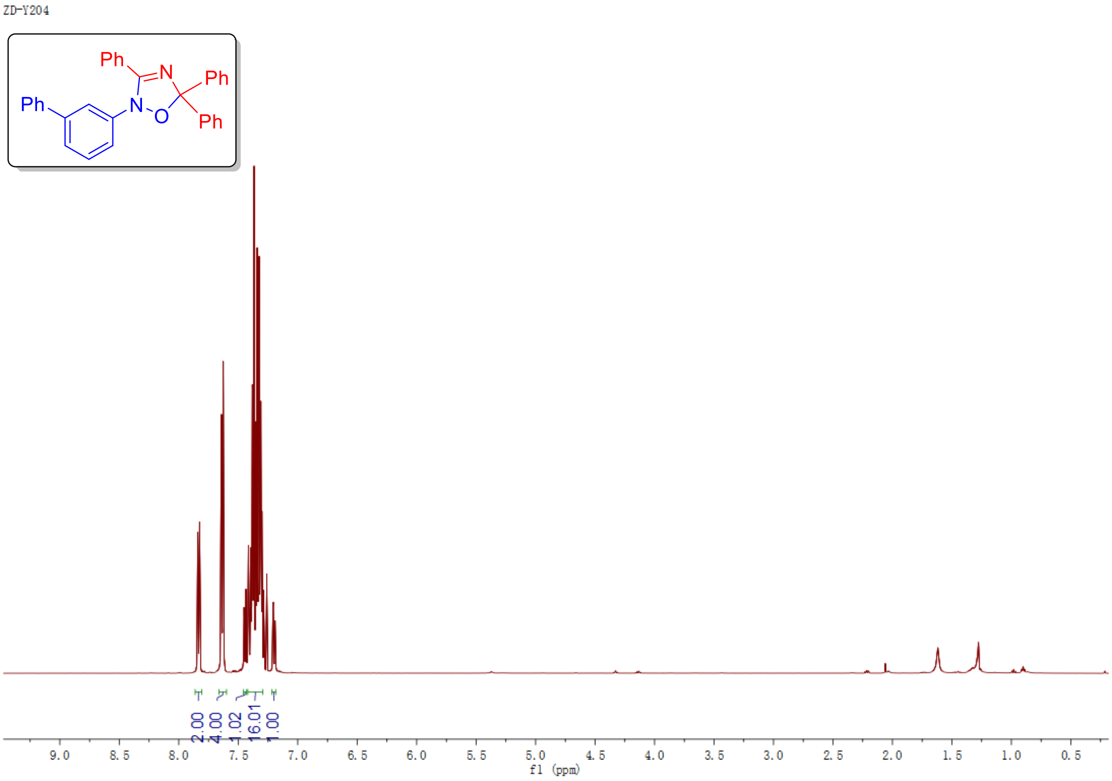

Supplementary Figure 72. ^{13}C **NMR Spectrum of 3ma (125 MHz,** $CDCl_3$**)**

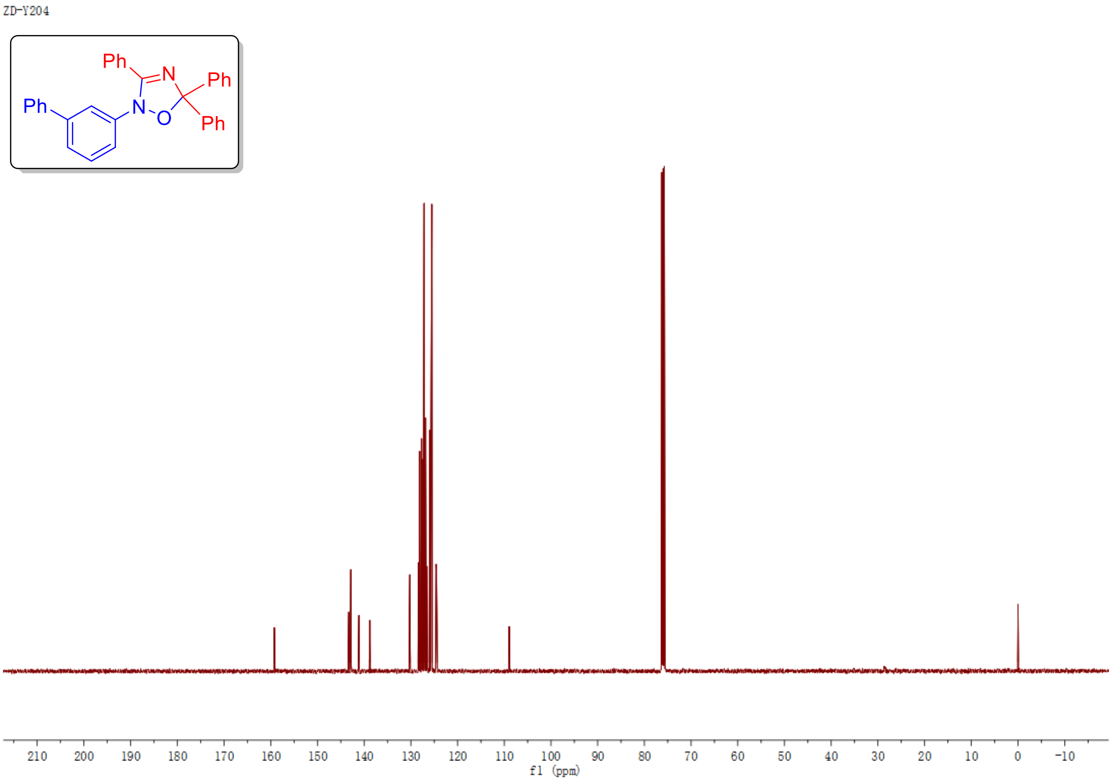

Supplementary Figure 73. ^{1}H NMR Spectrum of 3na (500 MHz, $CDCl_3$)

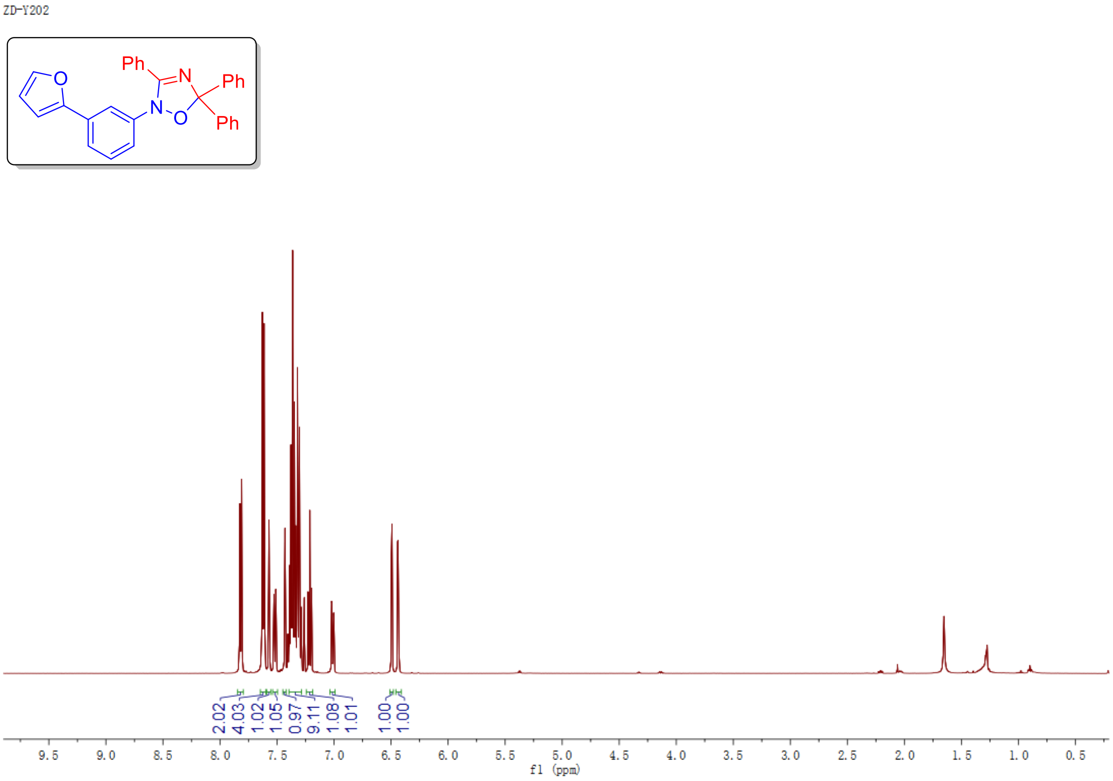

Supplementary Figure 74. ^{13}C NMR Spectrum of 3na (125 MHz, $CDCl_3$)

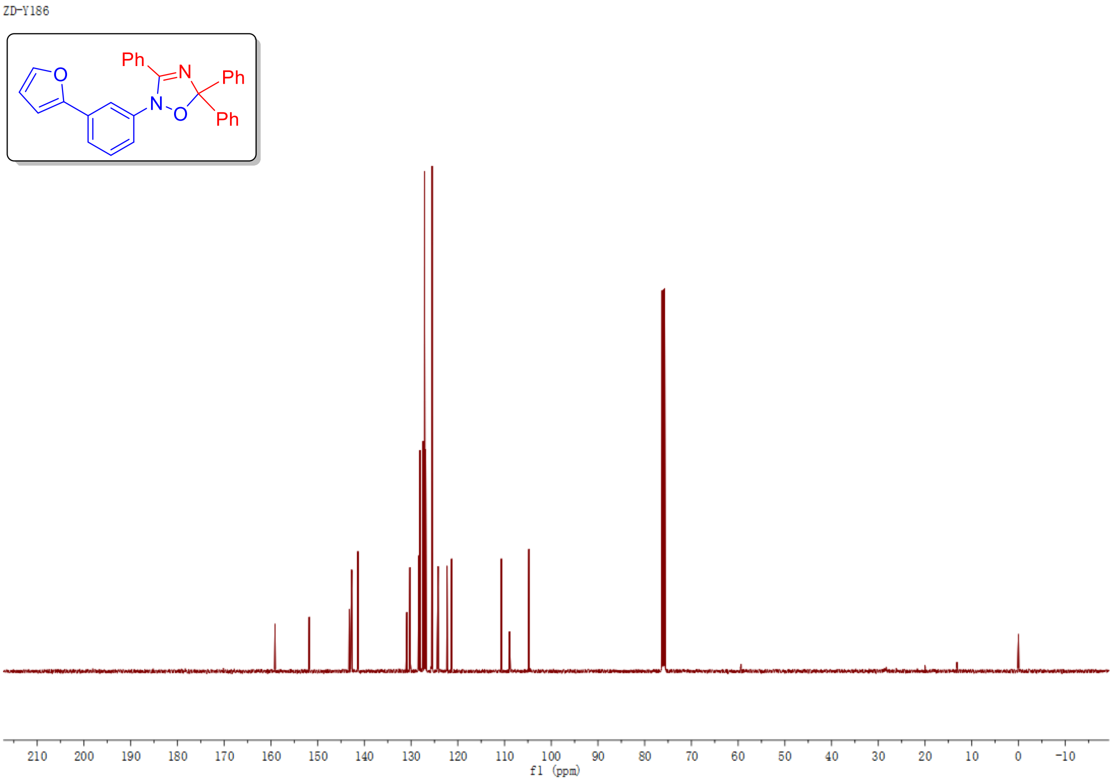

Supplementary Figure 75. ^{1}H NMR Spectrum of 3oa (500 MHz, $CDCl_3$)

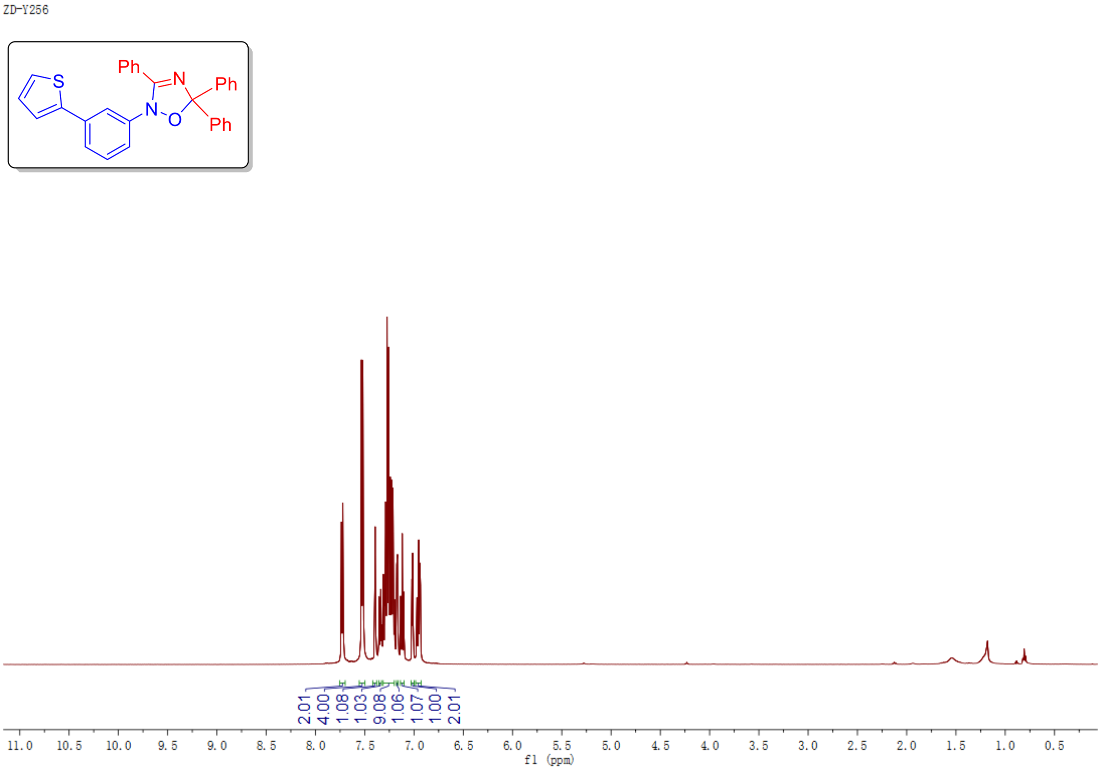

Supplementary Figure 76. ^{13}C NMR Spectrum of 3oa (125 MHz, $CDCl_3$)

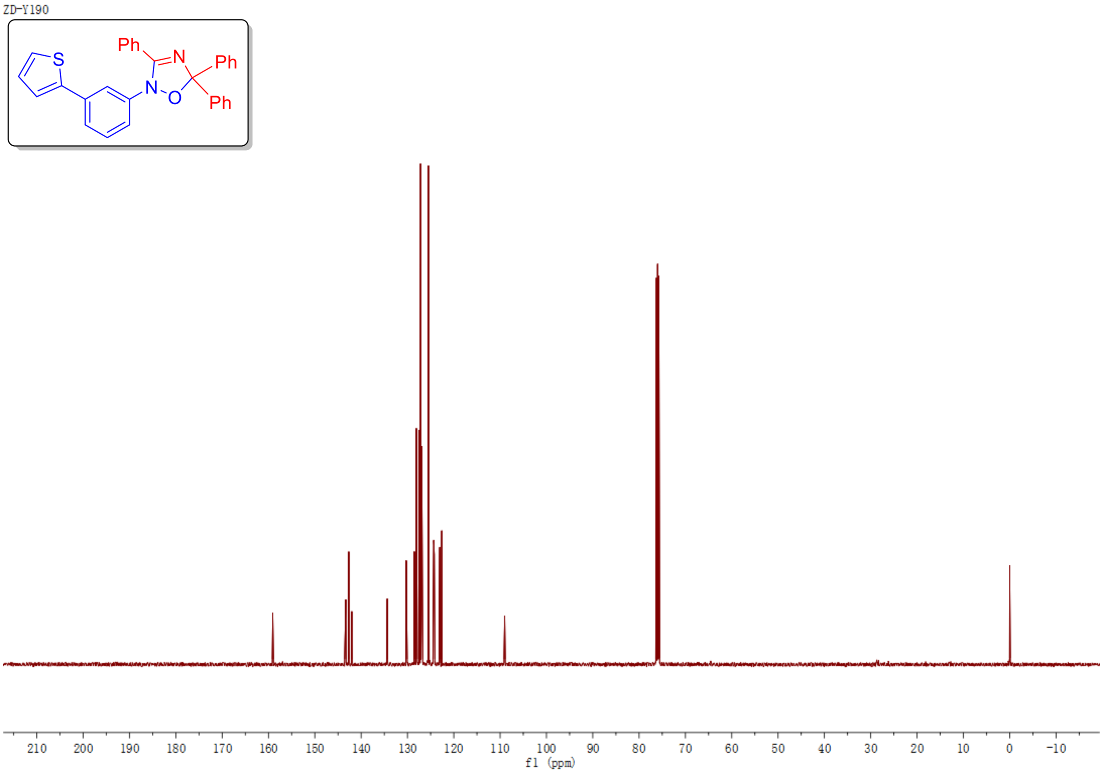

Supplementary Figure 77. **^{1}H NMR Spectrum of 3pa (500 MHz, $CDCl_3$)**

ZD-Y357

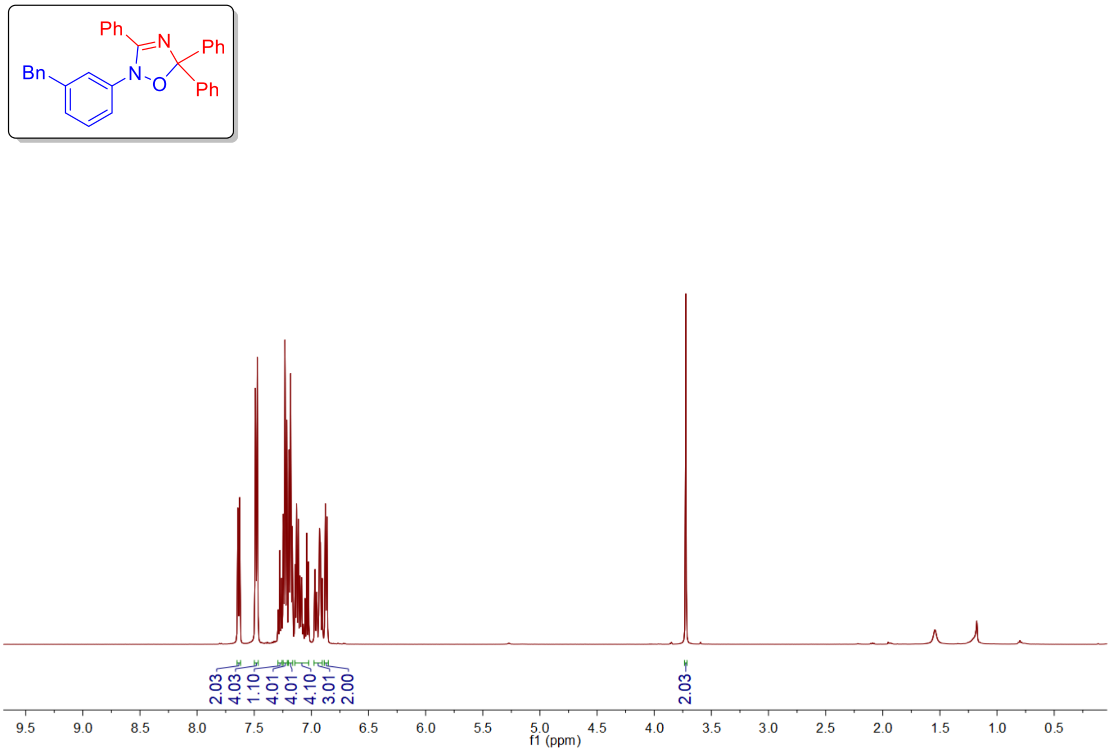

Supplementary Figure 78. **^{13}C NMR Spectrum of 3pa (125 MHz, $CDCl_3$)**

ZD-Y357

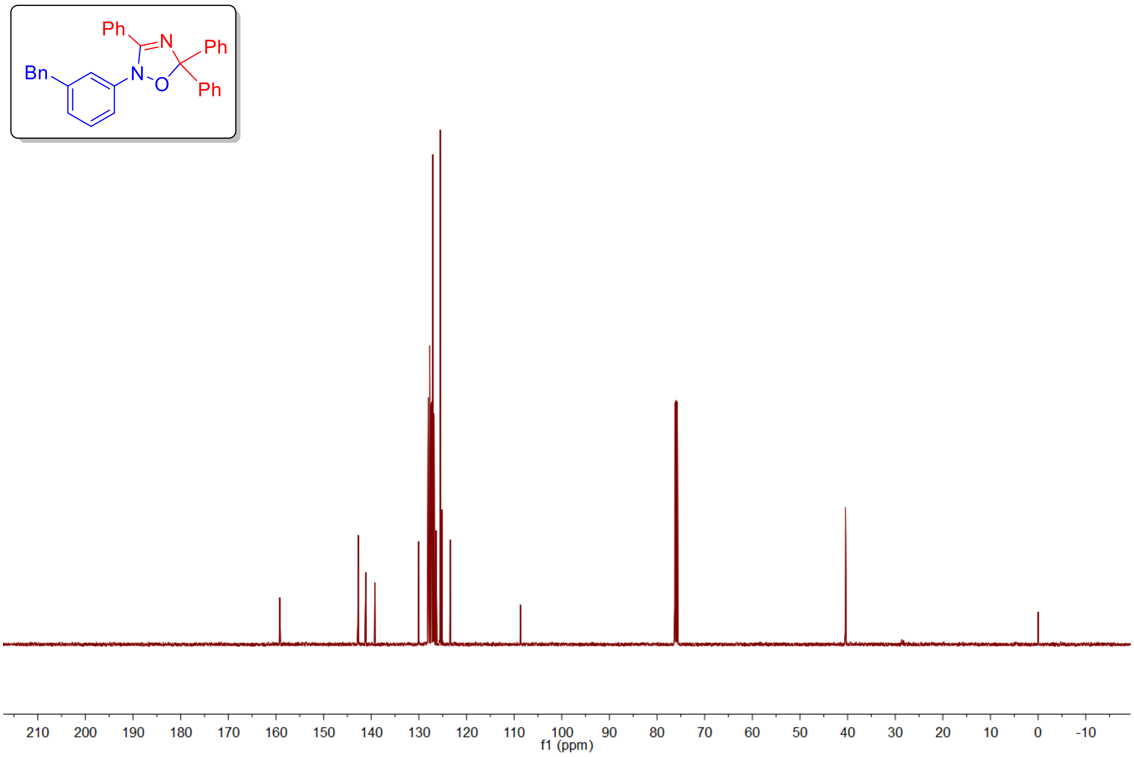

Supplementary Figure 79. ^{1}H NMR Spectrum of 3qa (500 MHz, $CDCl_3$)

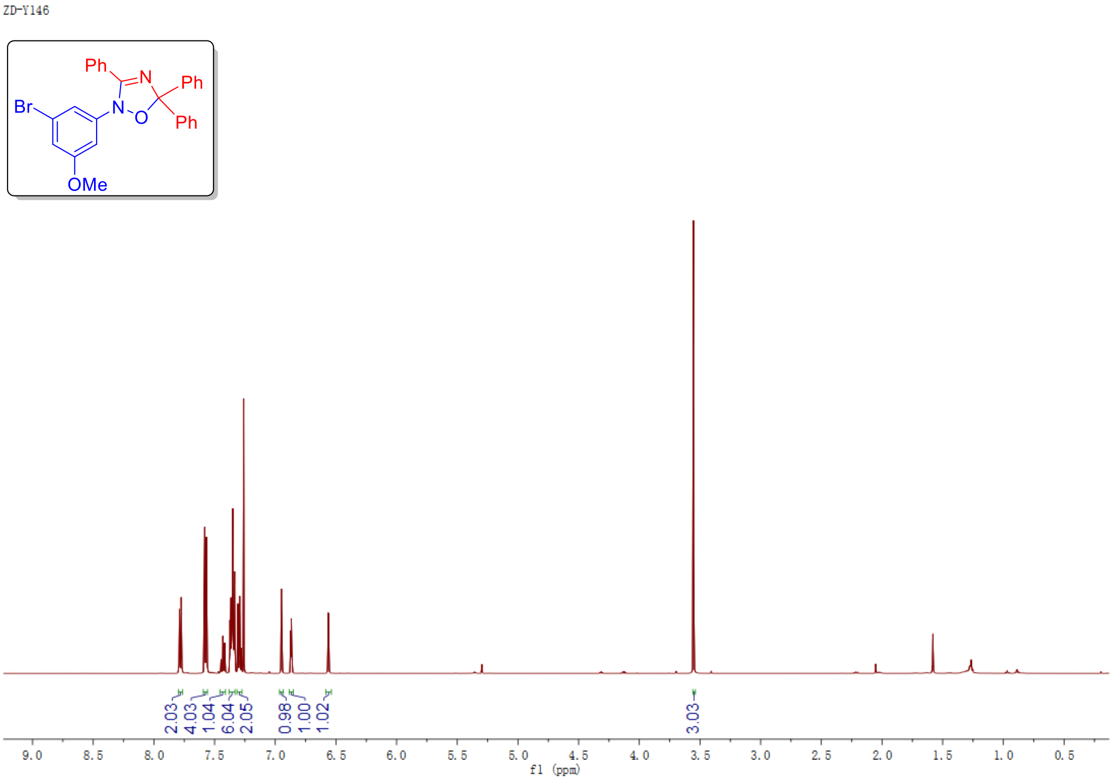

Supplementary Figure 80. ^{13}C NMR Spectrum of 3qa (125 MHz, $CDCl_3$)

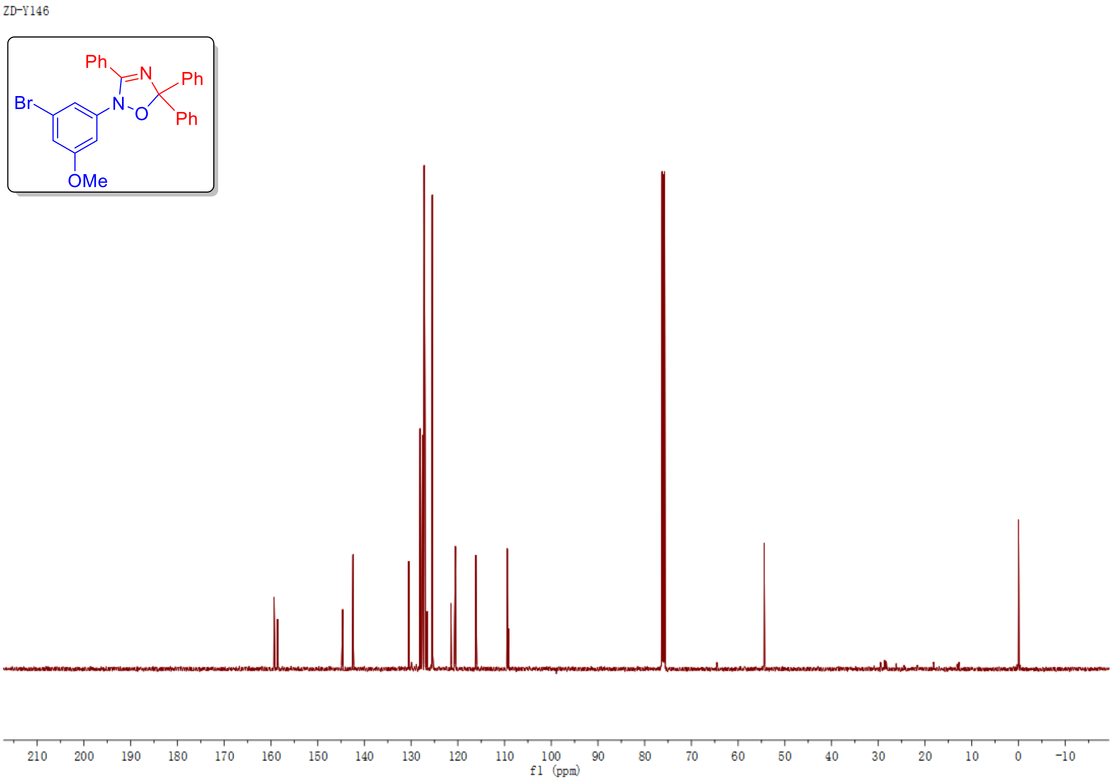

Supplementary Figure 81. ^{1}H NMR Spectrum of 3ra (500 MHz, CDCl$_3$)

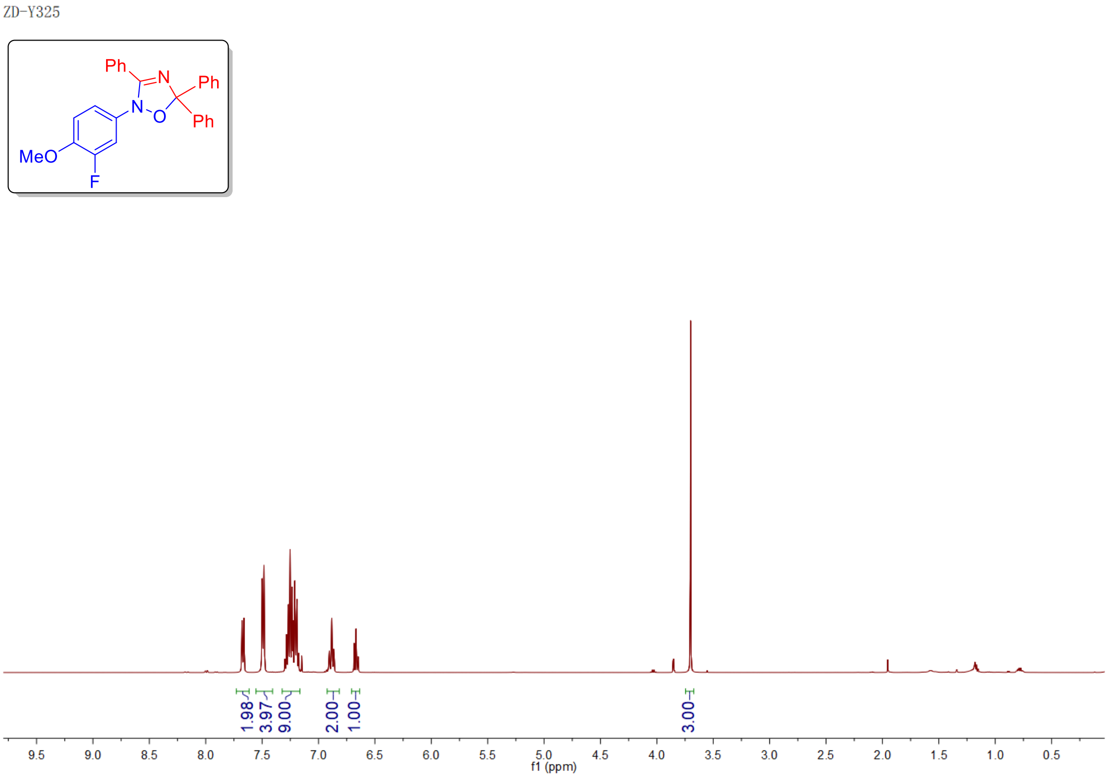

Supplementary Figure 82. ^{13}C NMR Spectrum of 3ra (125 MHz, CDCl$_3$)

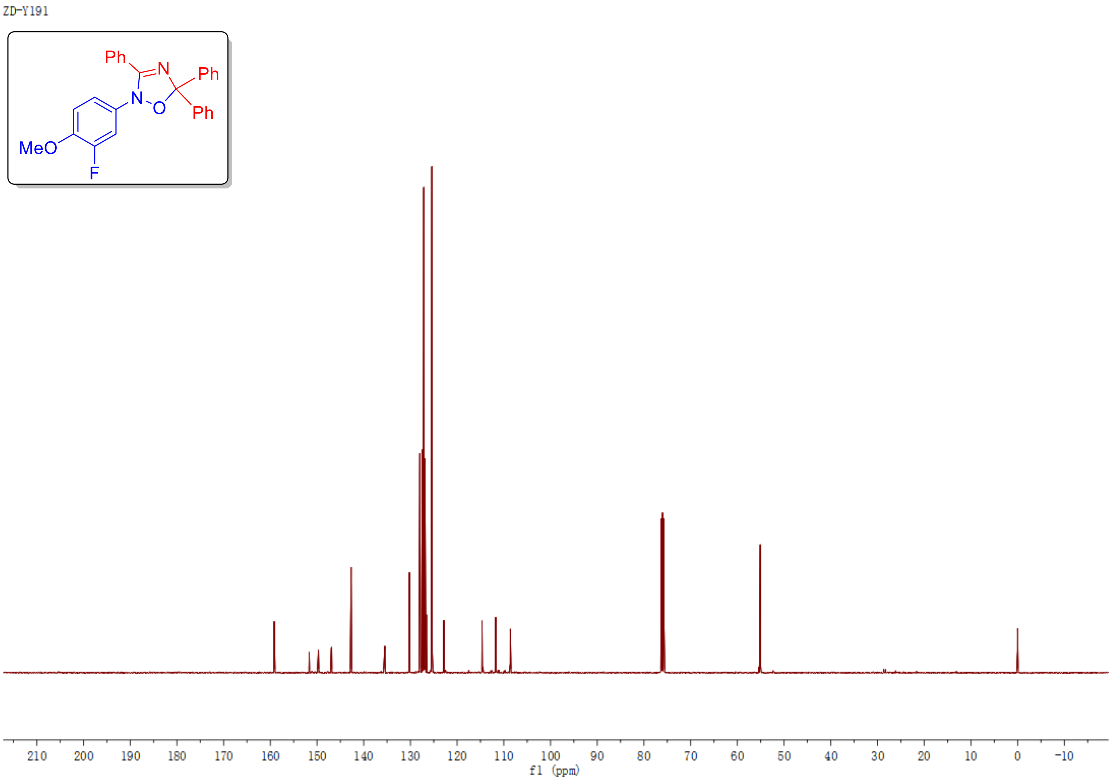

Supplementary Figure 83. ^{1}H NMR Spectrum of 3sa (500 MHz, CDCl$_3$)

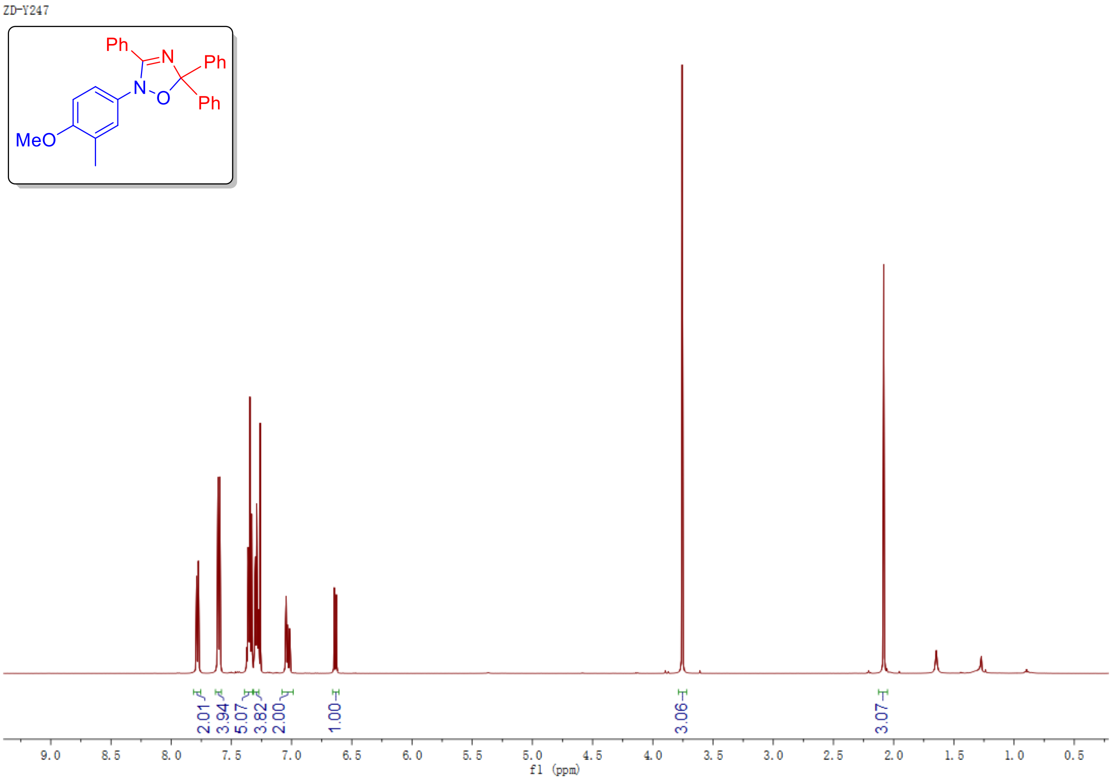

Supplementary Figure 84. ^{13}C NMR Spectrum of 3sa (125 MHz, CDCl$_3$)

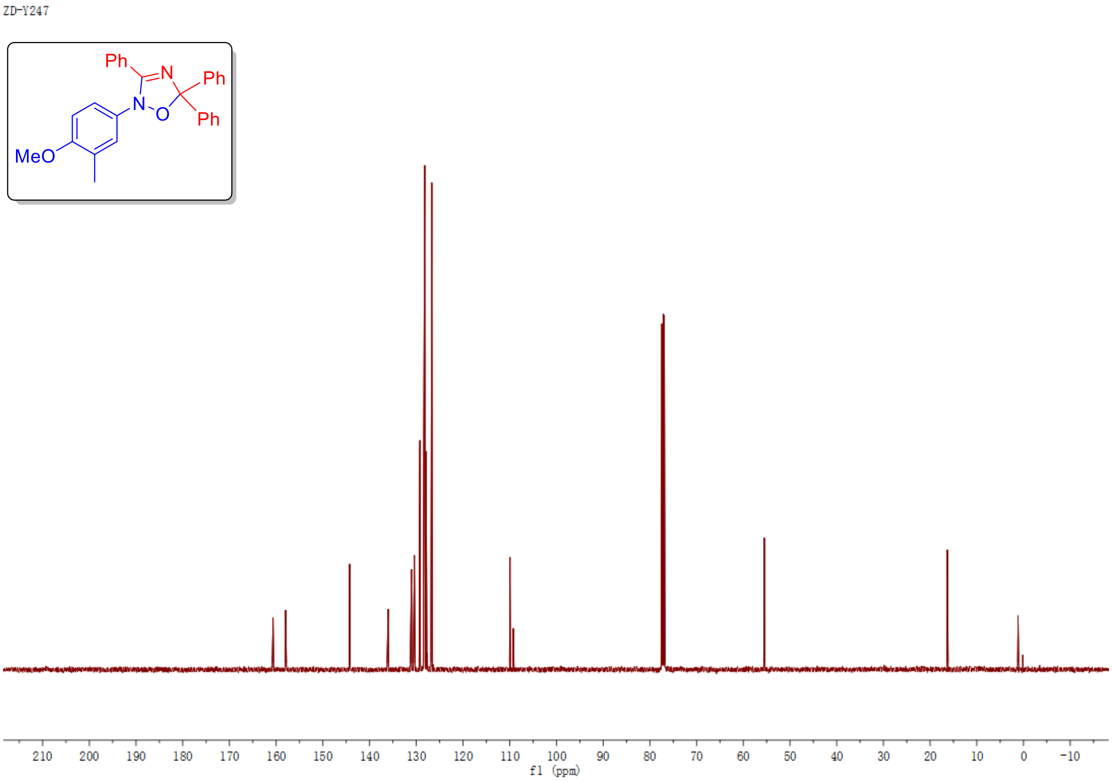

Supplementary Figure 85. ^{1}**H NMR Spectrum of 5 (500 MHz, CDCl$_3$)**

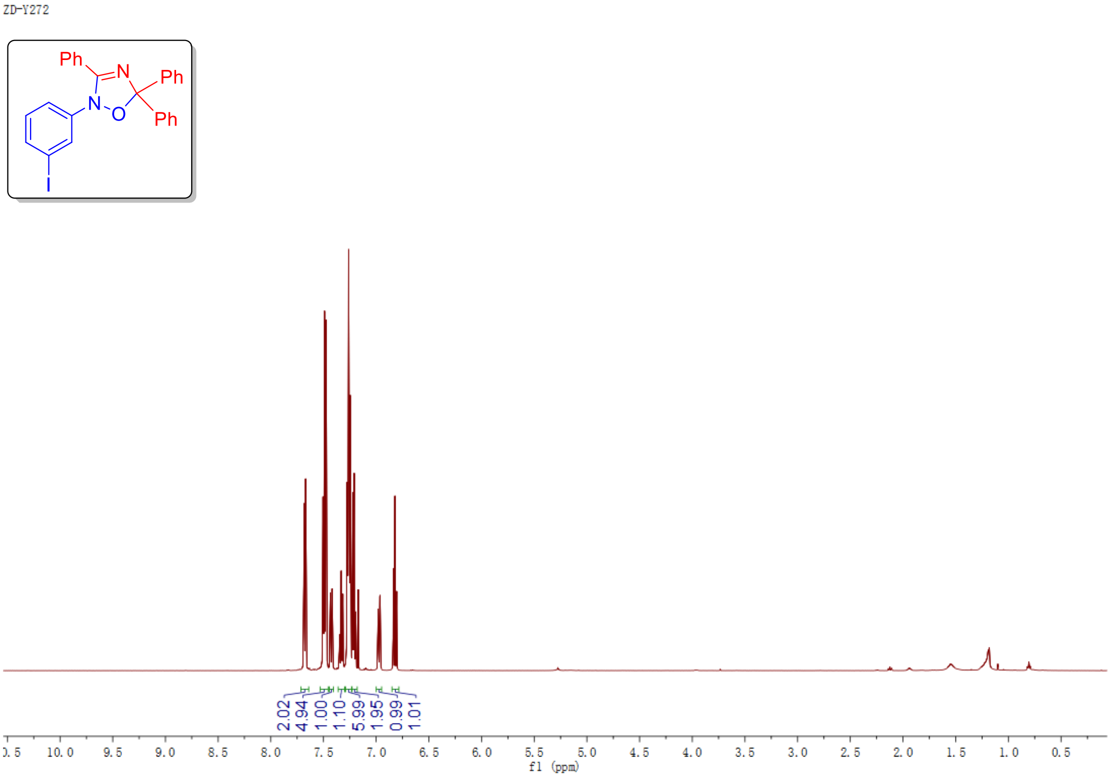

Supplementary Figure 86. ^{13}**C NMR Spectrum of 5 (125 MHz, CDCl$_3$)**

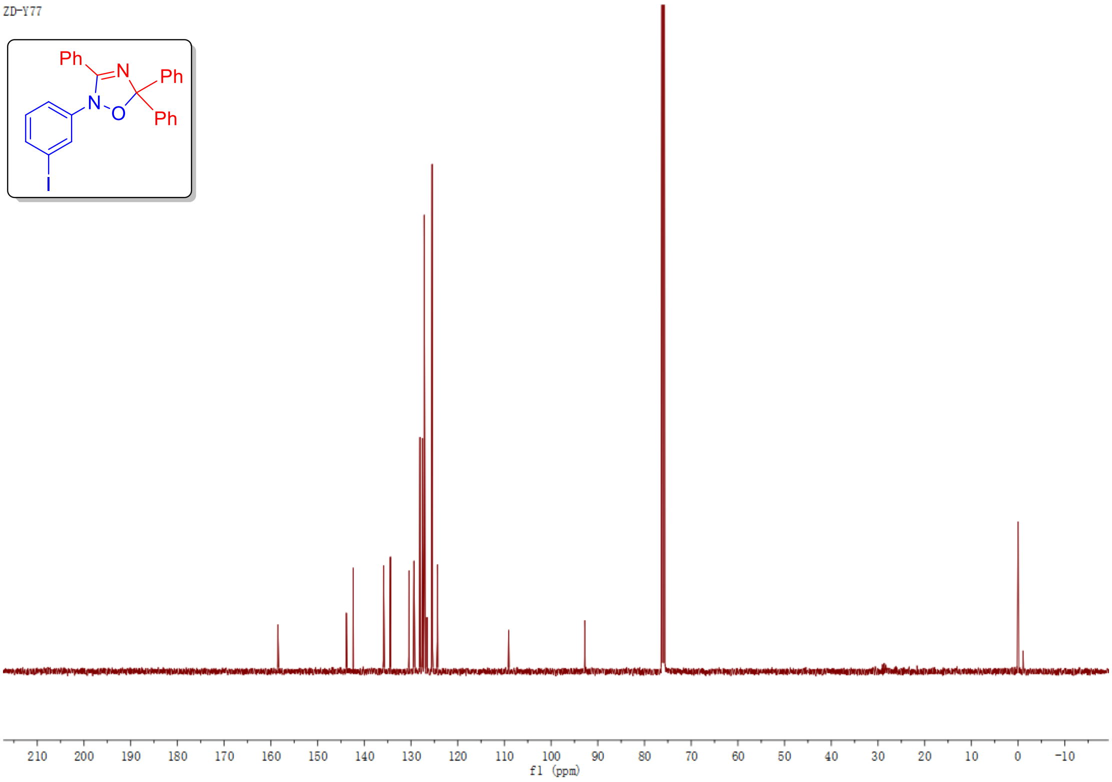

Supplementary Figure 87. ^{1}H NMR Spectrum of 3aA (500 MHz, $CDCl_3$)

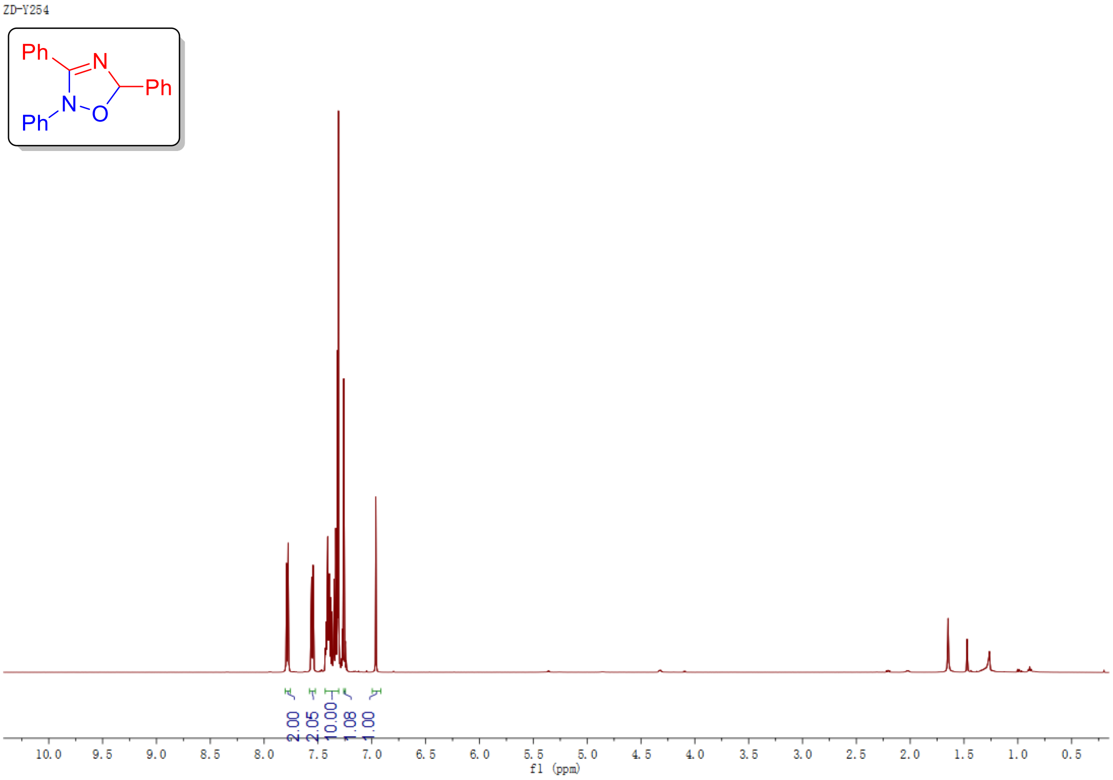

Supplementary Figure 88. ^{1}H NMR Spectrum of 3aB (500 MHz, $CDCl_3$)

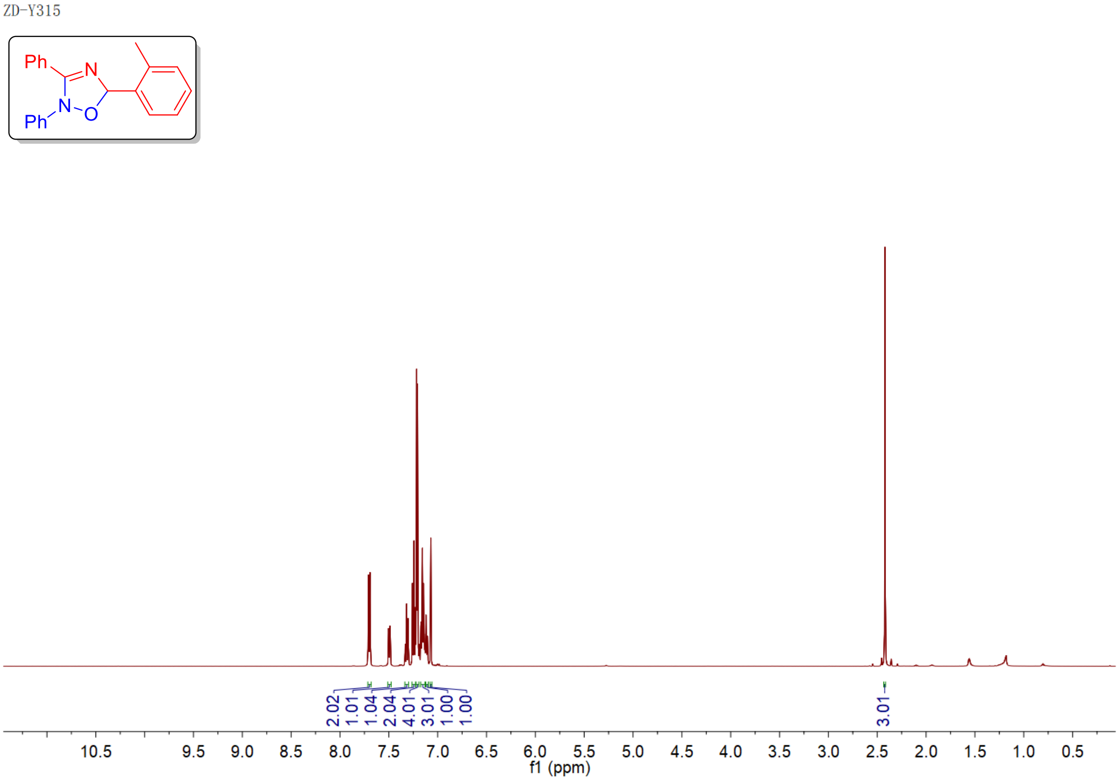

Supplementary Figure 89. ^{13}C NMR Spectrum of 3aB (125 MHz, $CDCl_3$)

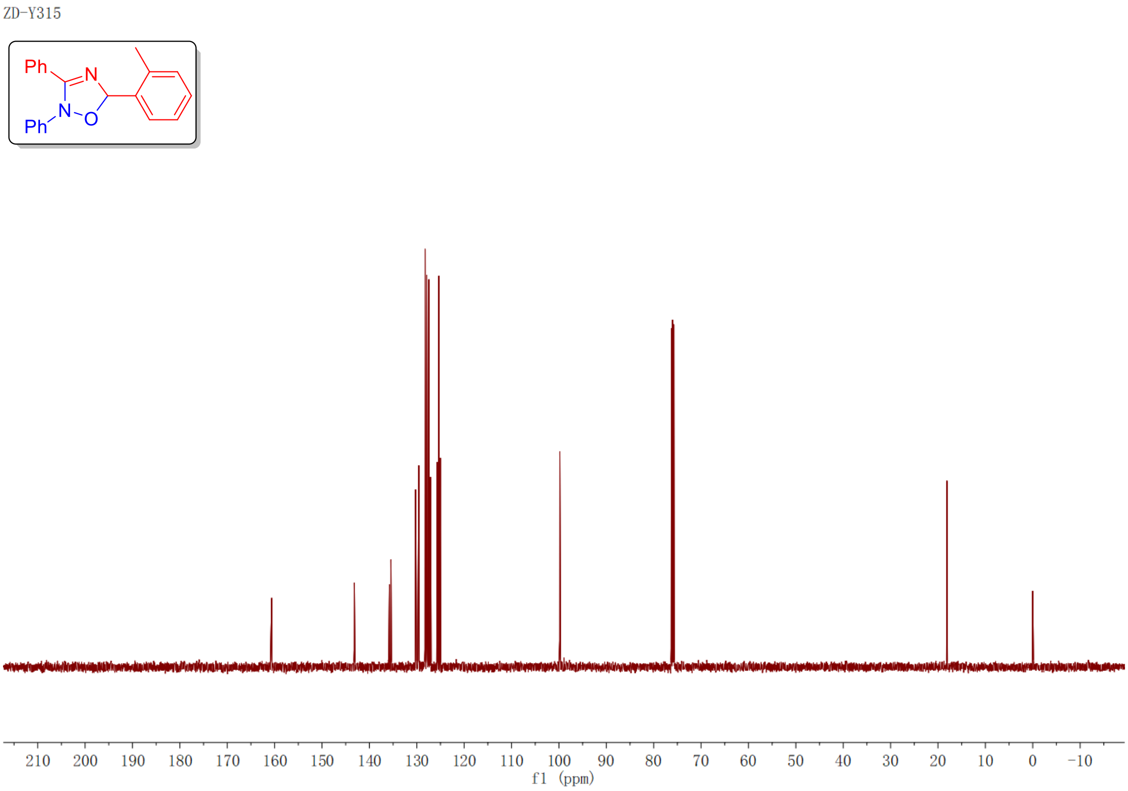

Supplementary Figure 90. ^{1}H-^{1}H COSY Spectrum of 3aB (500 MHz, $CDCl_3$)

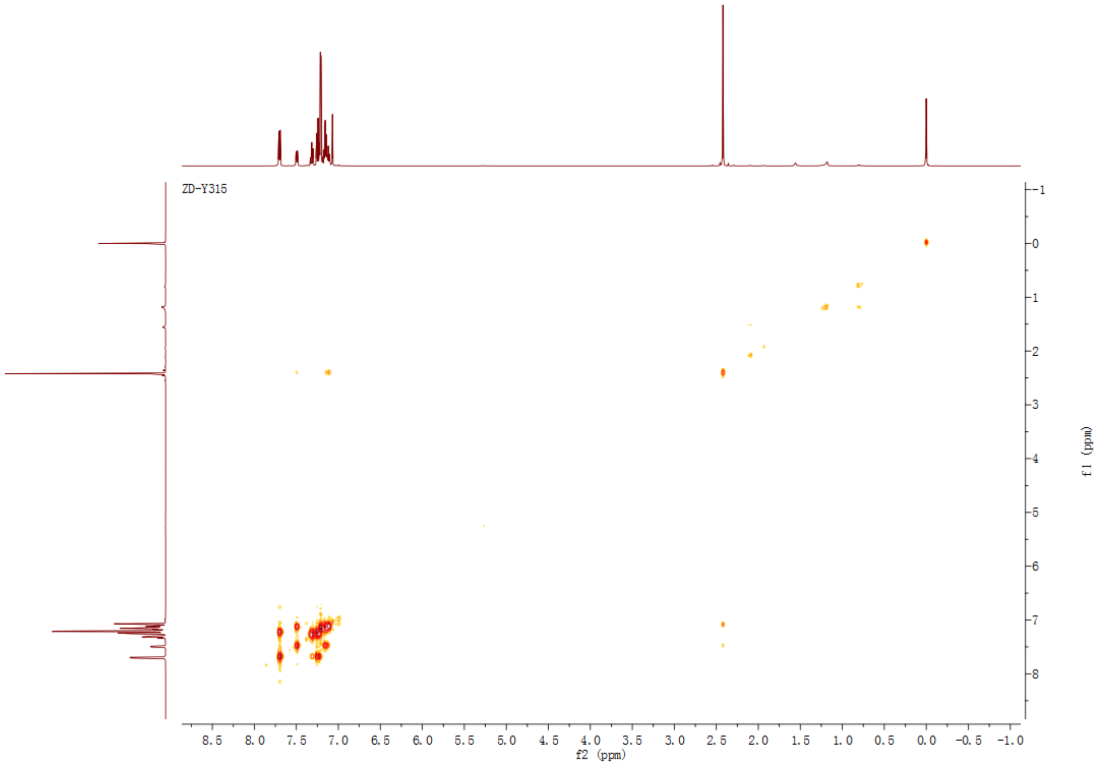

Supplementary Figure 91. HMQC Spectrum of 3aB (500 MHz, $CDCl_3$)

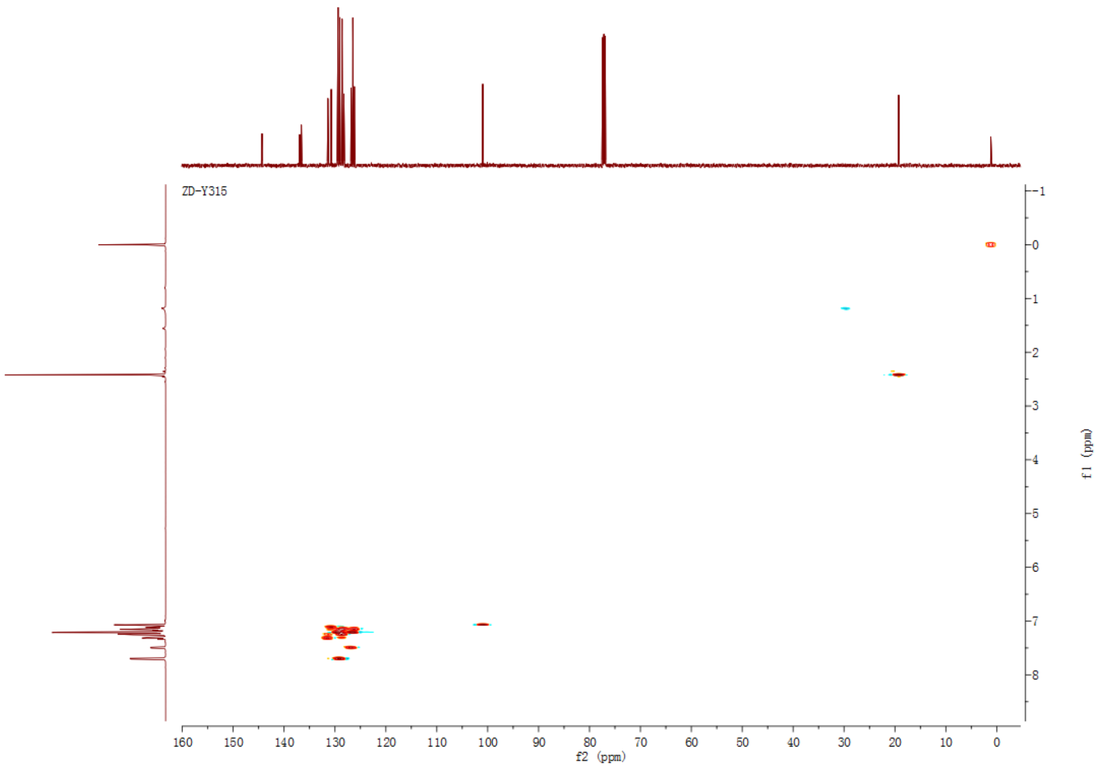

Supplementary Figure 92. HMBC Spectrum of 3aB (500 MHz, $CDCl_3$)

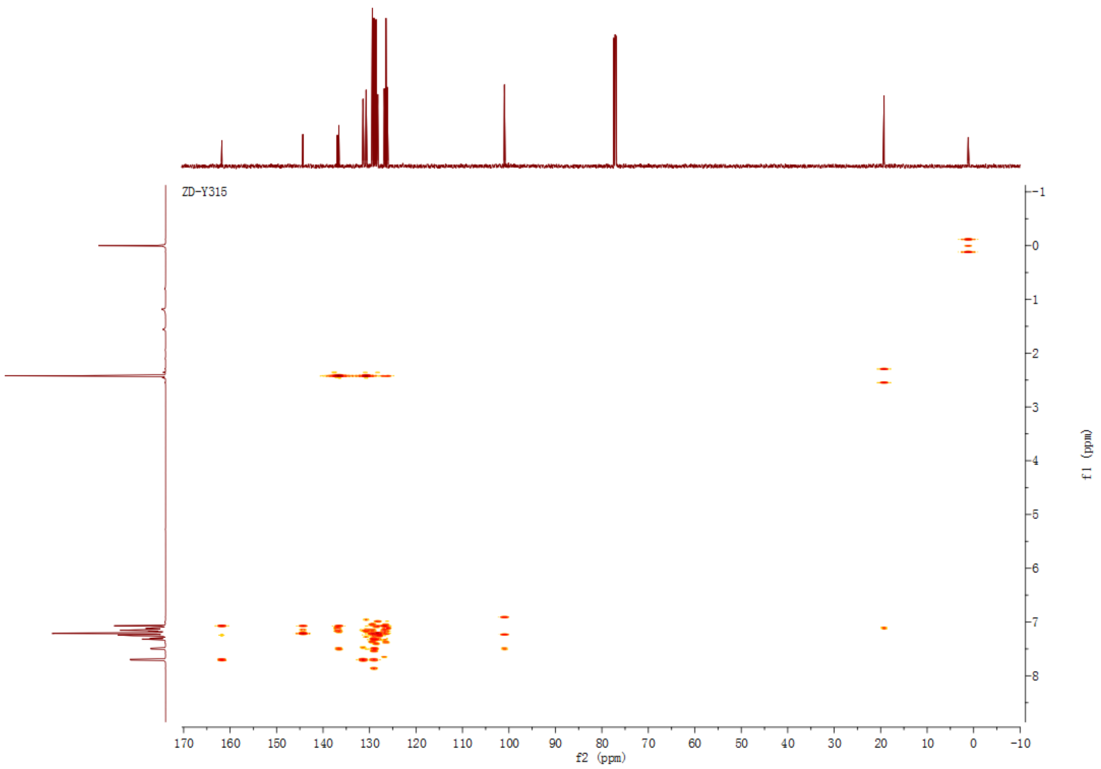

Supplementary Figure 93. ¹H NMR Spectrum of 3aC (500 MHz, $CDCl_3$)

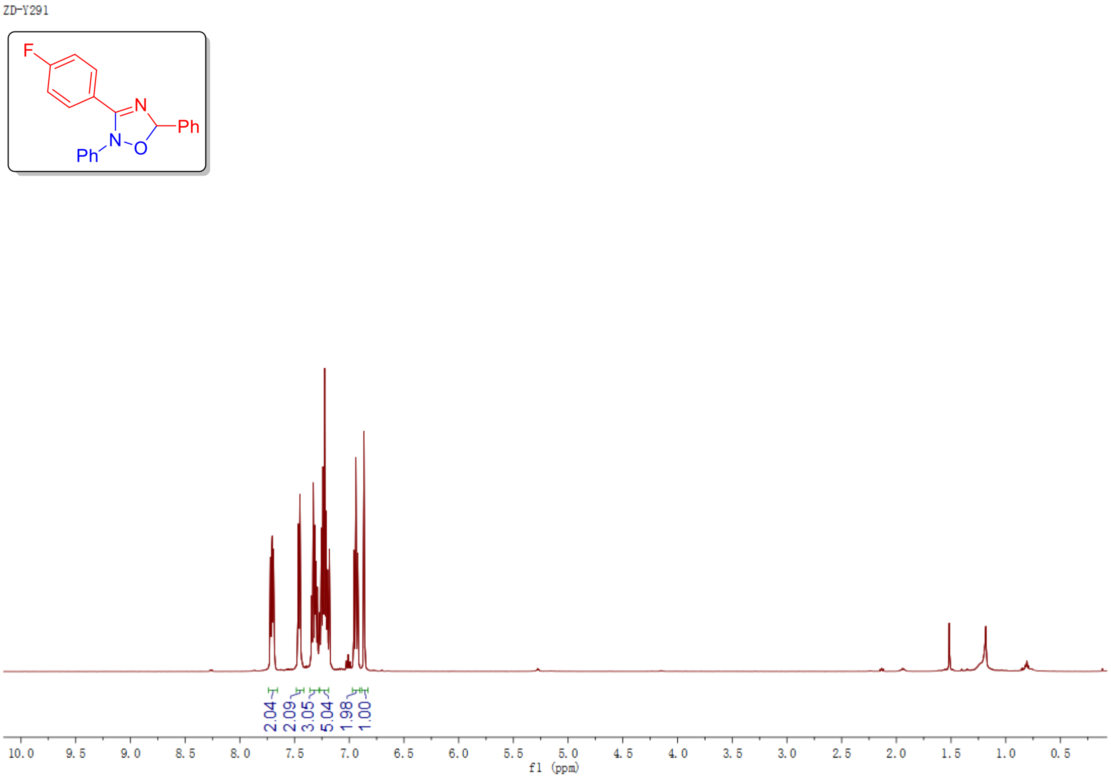

Supplementary Figure 94. ¹H NMR Spectrum of 3aD (500 MHz, $CDCl_3$)

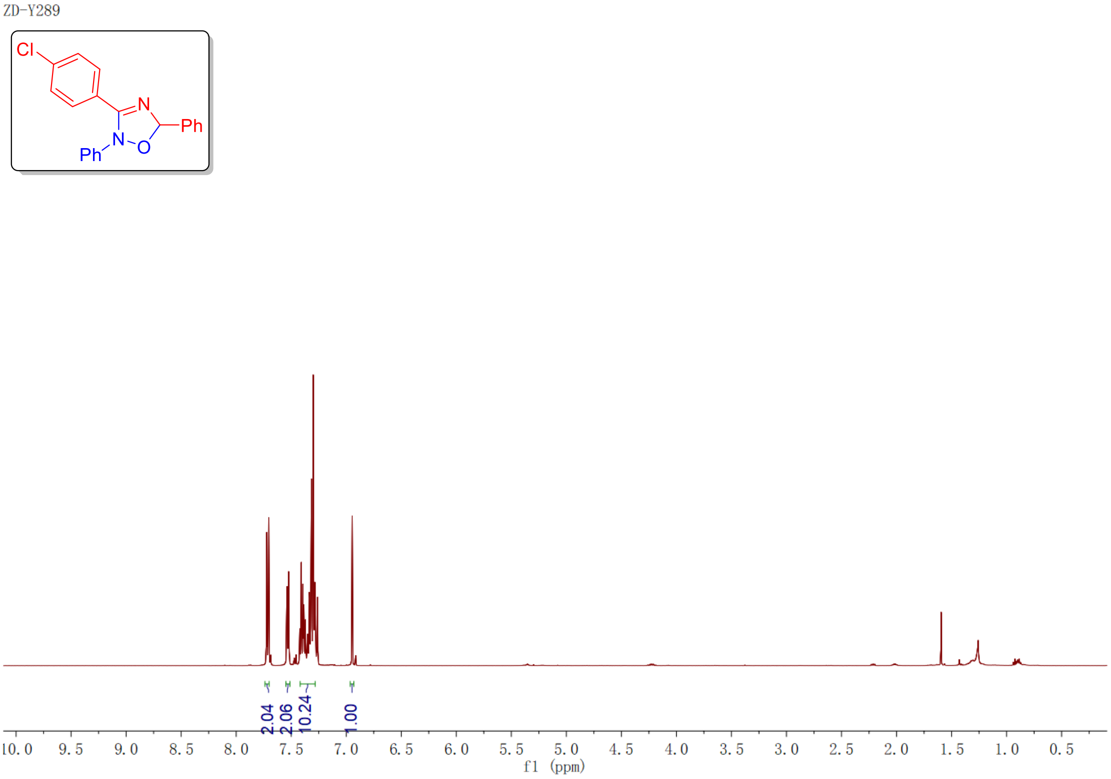

Supplementary Figure 95. ^{1}H NMR Spectrum of 3aE (500 MHz, $CDCl_3$)

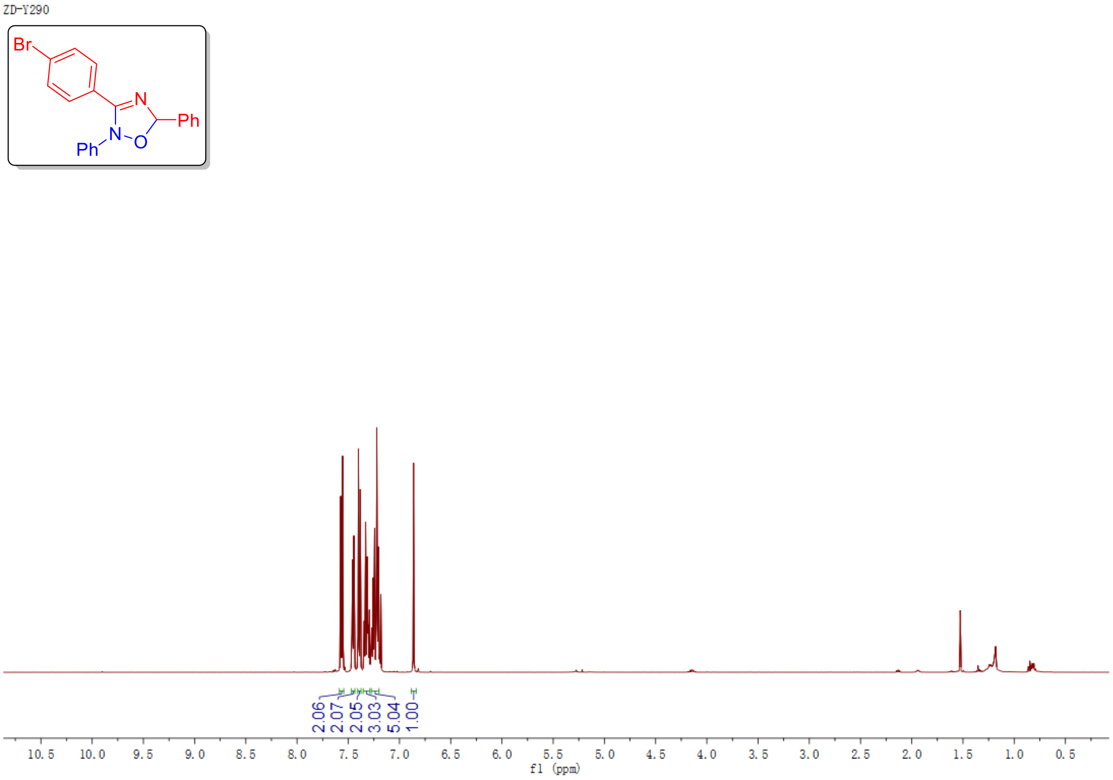

Supplementary Figure 96. ^{13}C NMR Spectrum of 3aE (125 MHz, $CDCl_3$)

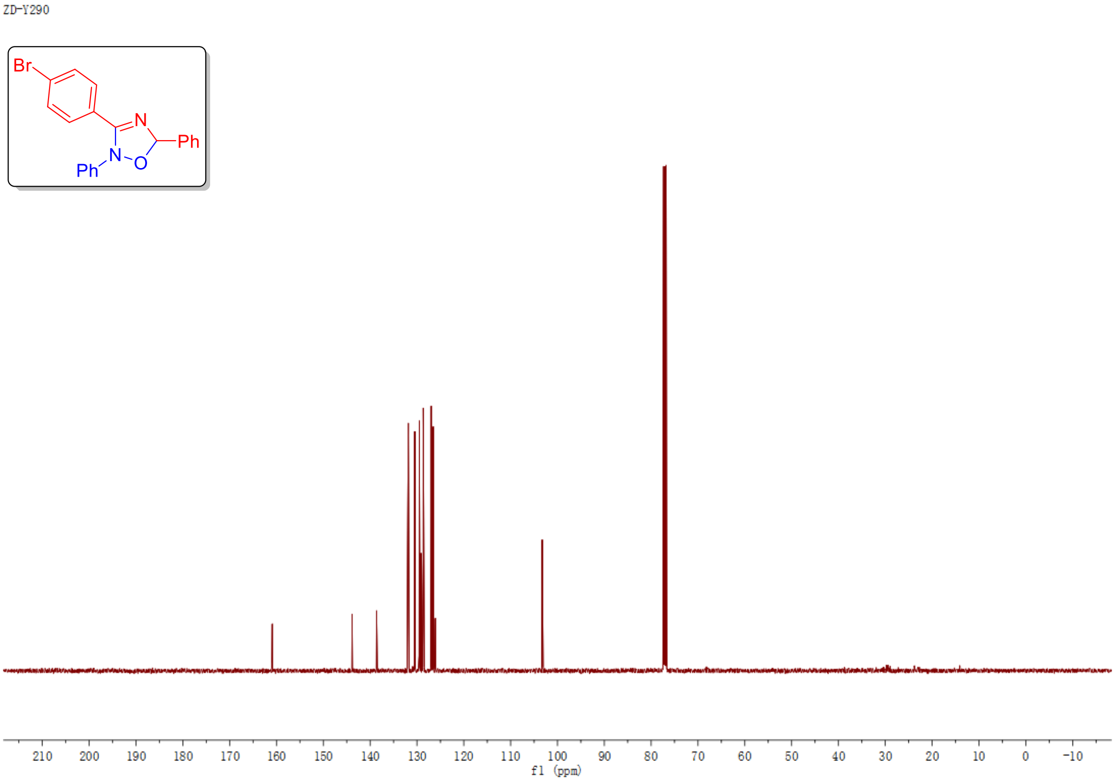

Supplementary Figure 97. ^{1}H NMR Spectrum of 3aF and 3aF' (500 MHz, CDCl3)

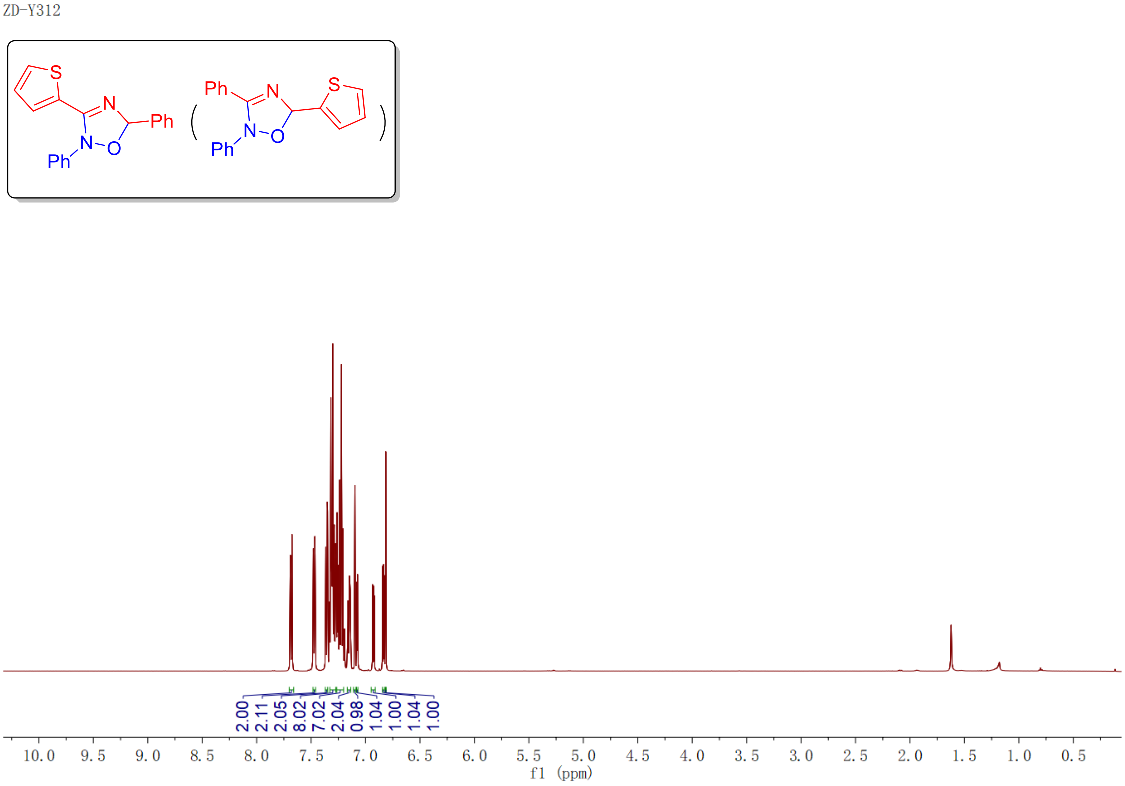

Supplementary Figure 98. ^{13}C NMR Spectrum of 3aE and 3aF' (125 MHz, CDCl3)

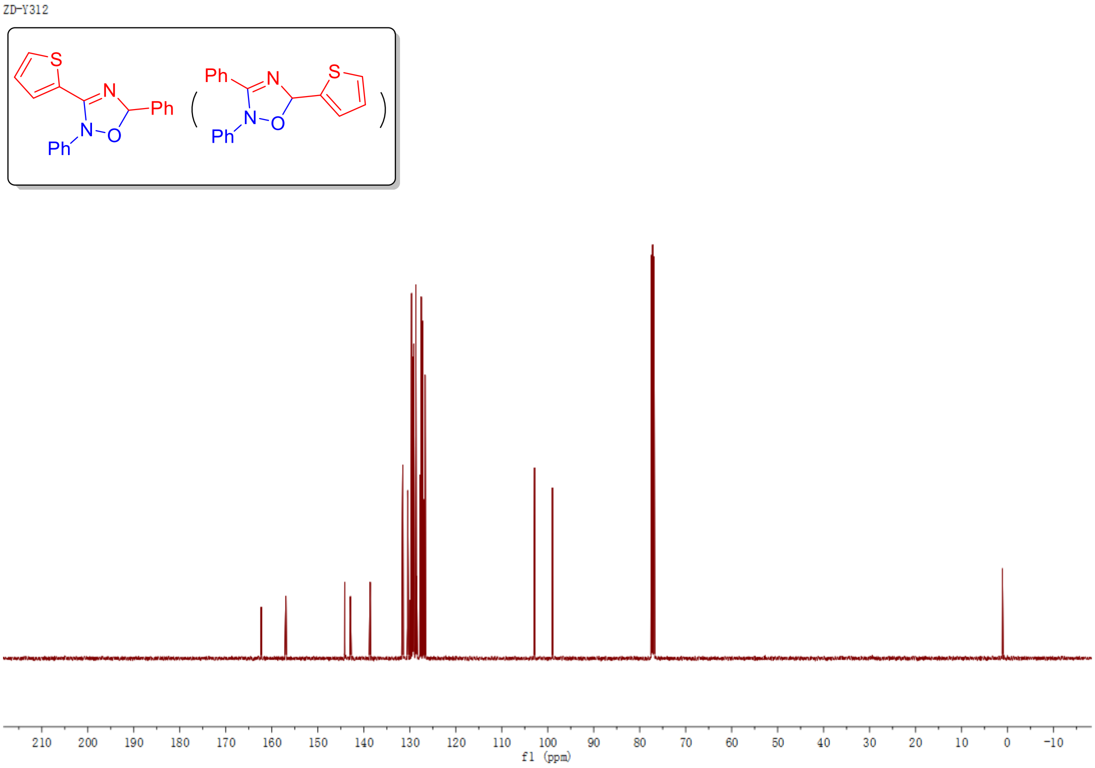

Supplementary Figure 99. ^{1}H NMR Spectrum of 3aG and 3aG' (500 MHz, $CDCl_3$)

ZD-Y313

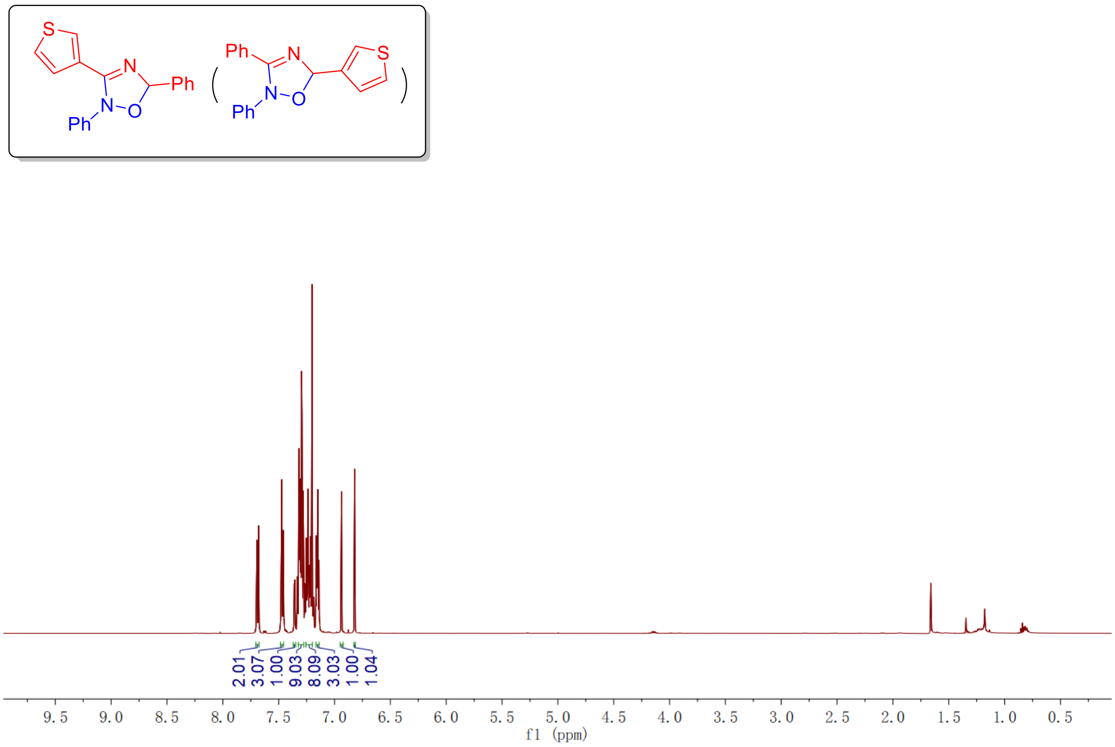

Supplementary Figure 100. ^{13}C NMR Spectrum of 3aG and 3aG' (125 MHz, $CDCl_3$)

ZD-Y313

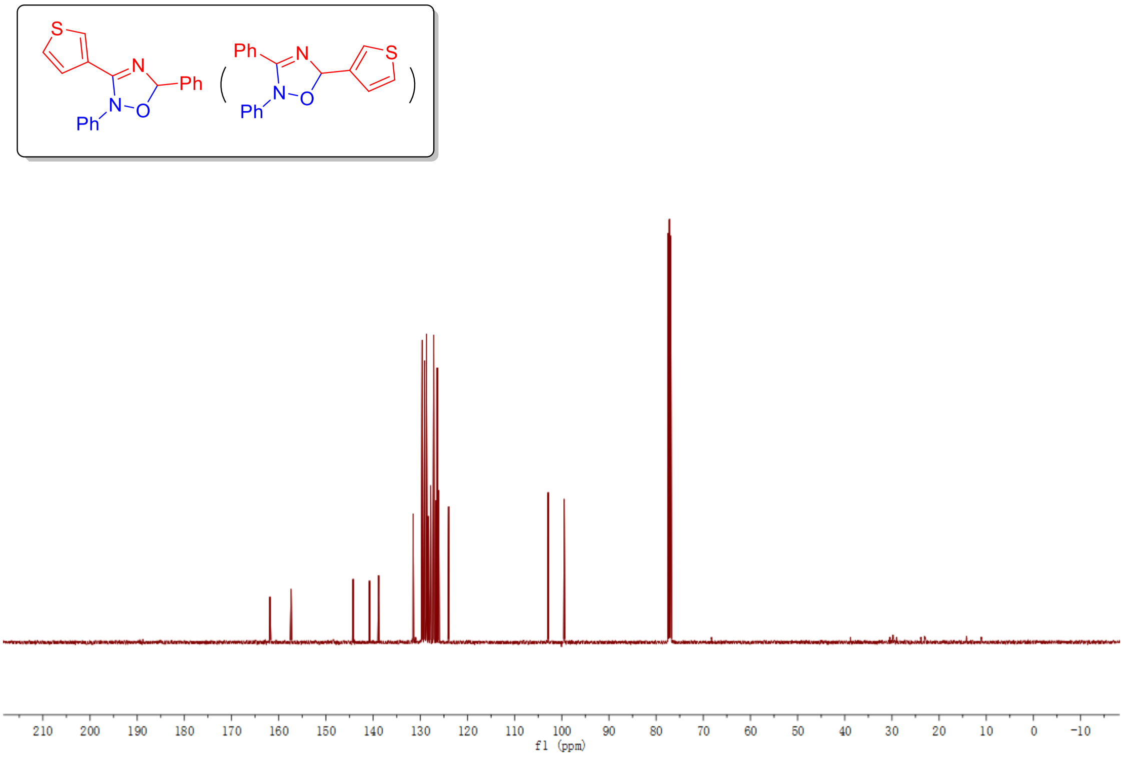

Supplementary Figure 101. ^{1}H NMR Spectrum of 3cA (500 MHz, CDCl$_3$)

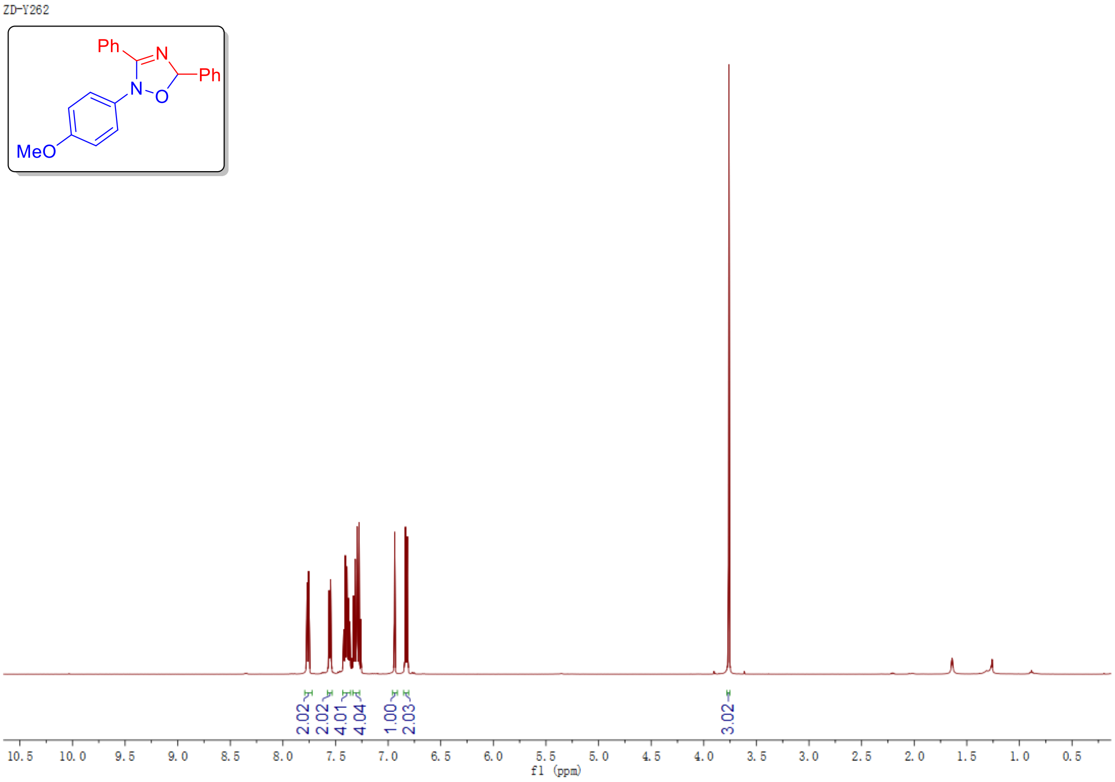

Supplementary Figure 102. ^{13}C NMR Spectrum of 3cA (125 MHz, CDCl$_3$)

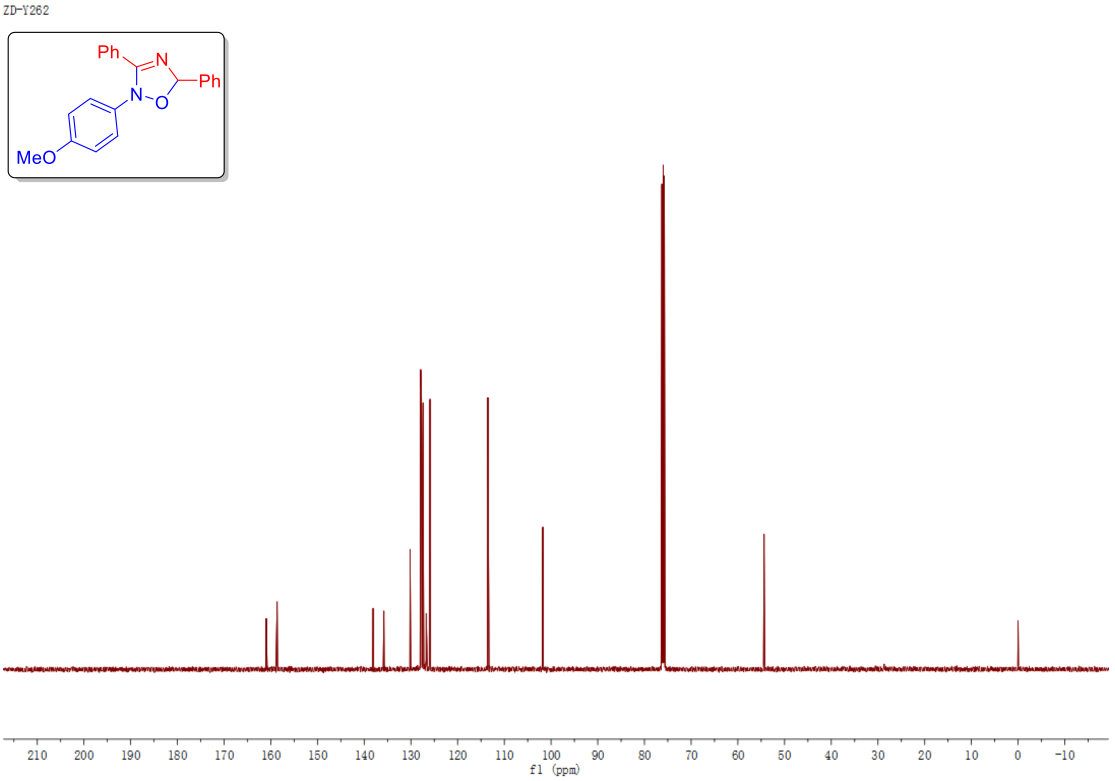

Supplementary Figure 103. ^{1}H NMR Spectrum of 3gA (500 MHz, $CDCl_3$)

ZD-Y292

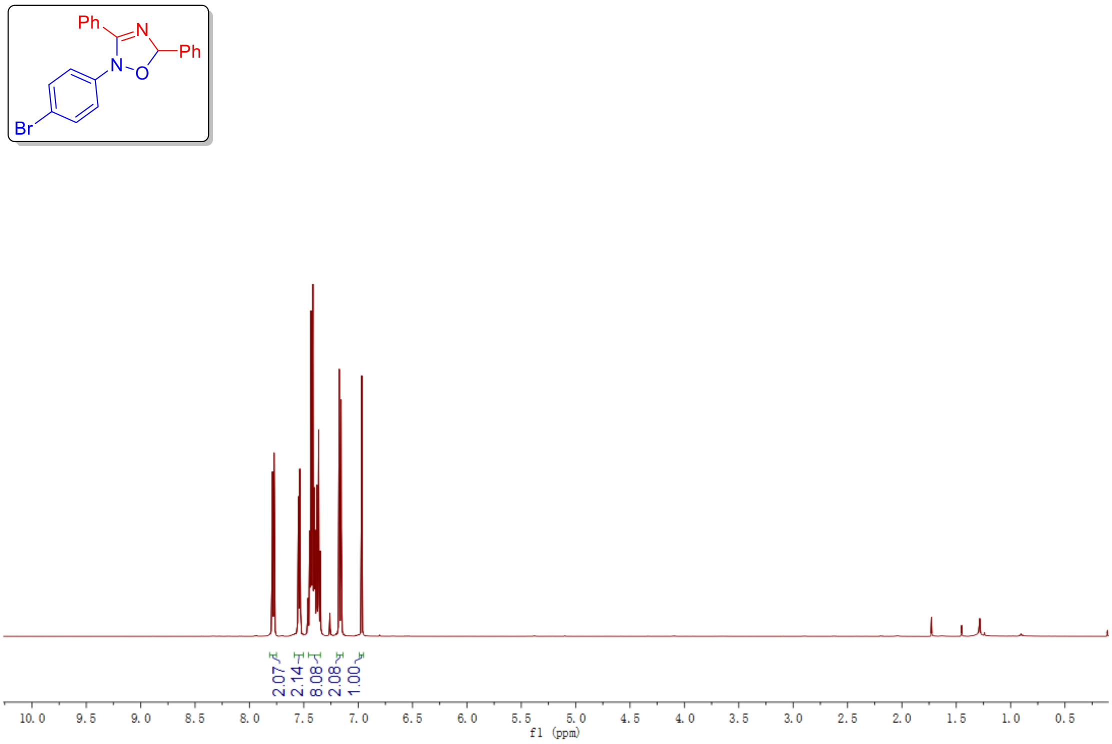

Supplementary Figure 104. ^{1}H NMR Spectrum of 3hA (500 MHz, $CDCl_3$)

ZD-Y299

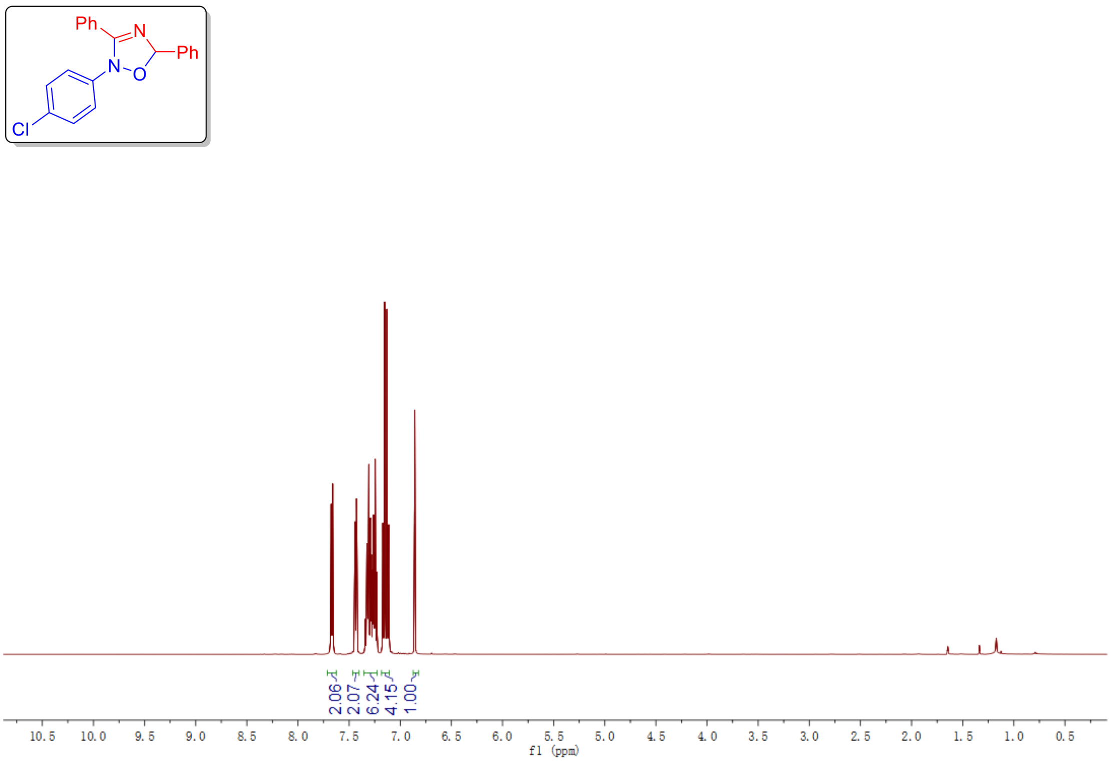

Supplementary Figure 105. ^{1}H NMR Spectrum of 3jA (500 MHz, DMSO-d_6)

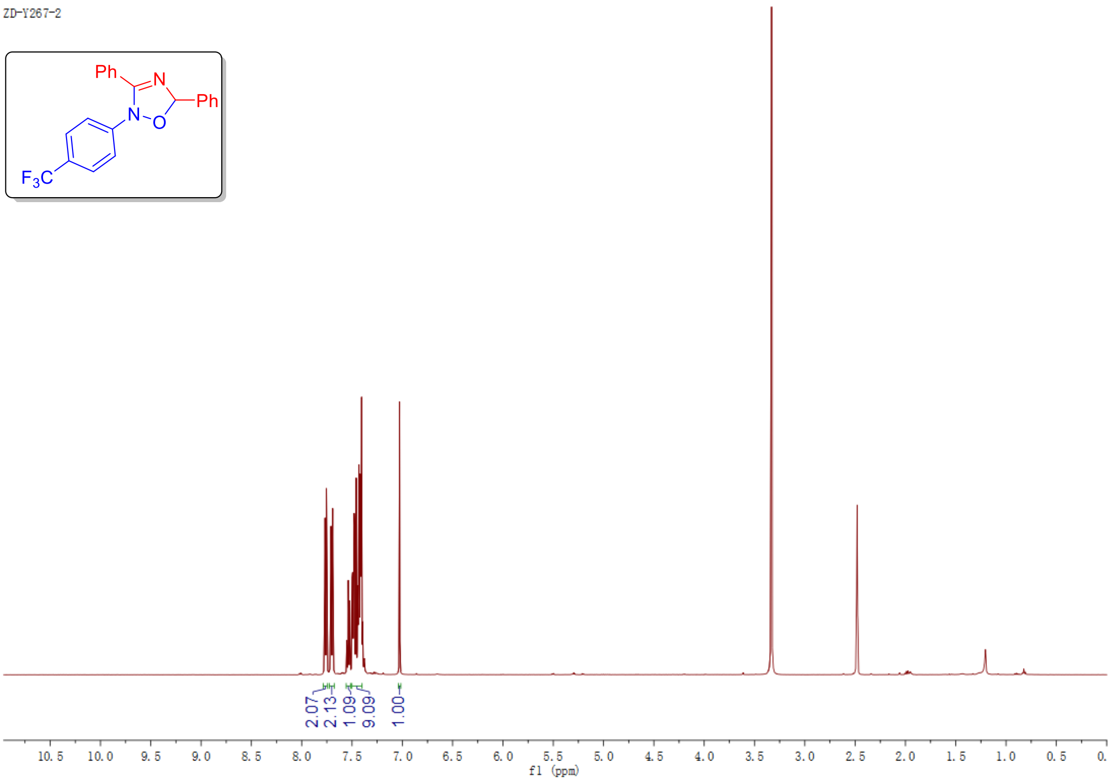

Supplementary Figure 106. ^{13}C NMR Spectrum of 3jA (125 MHz, DMSO-d_6)

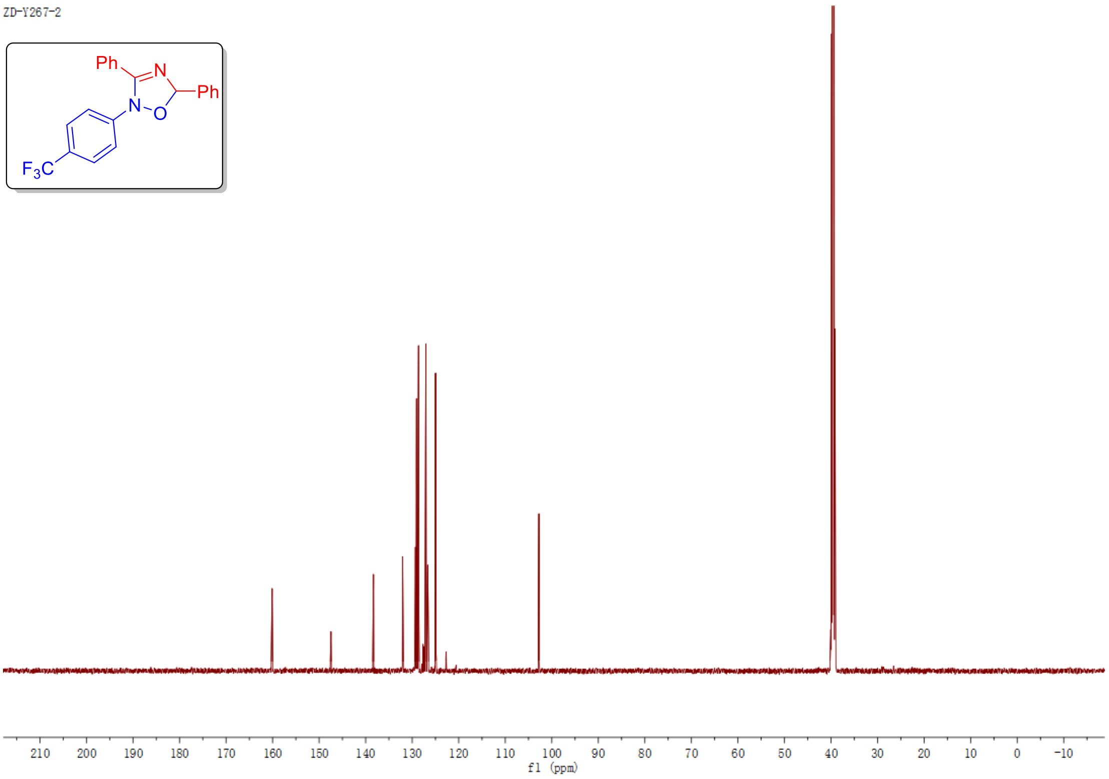

Supplementary Figure 107. ^{1}H NMR Spectrum of 3lA (500 MHz, $CDCl_3$)

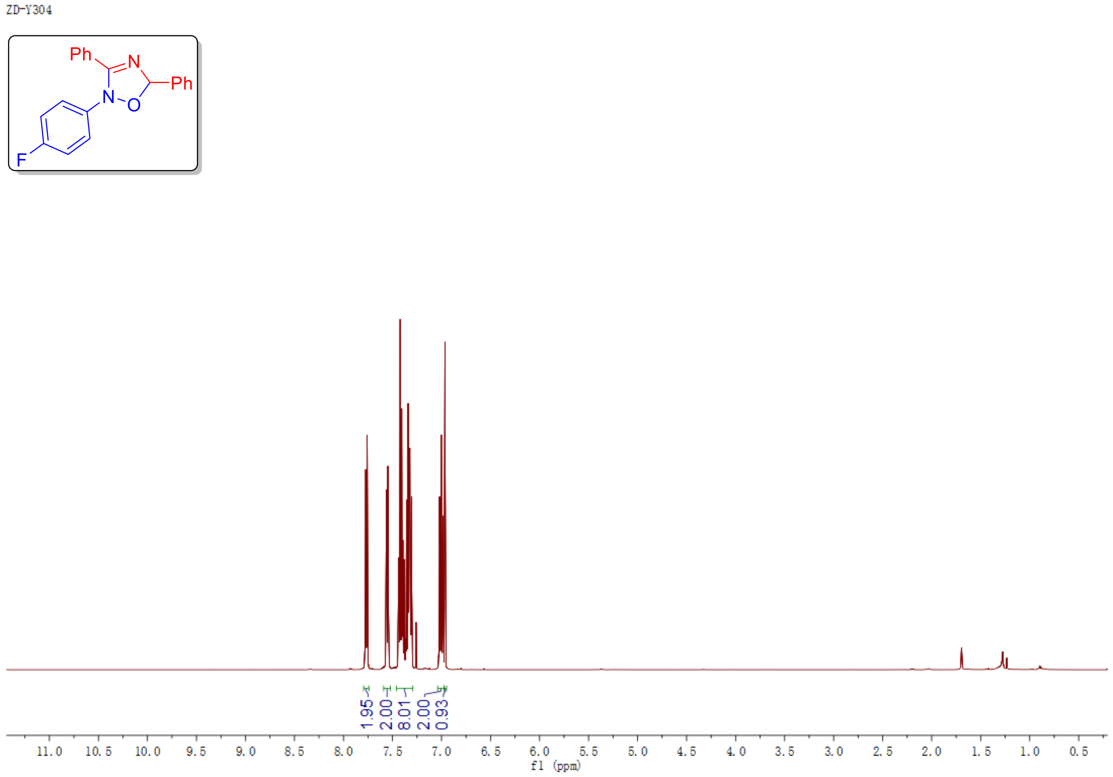

Supplementary Figure 108. ^{13}C NMR Spectrum of 3lA (125 MHz, $CDCl_3$)

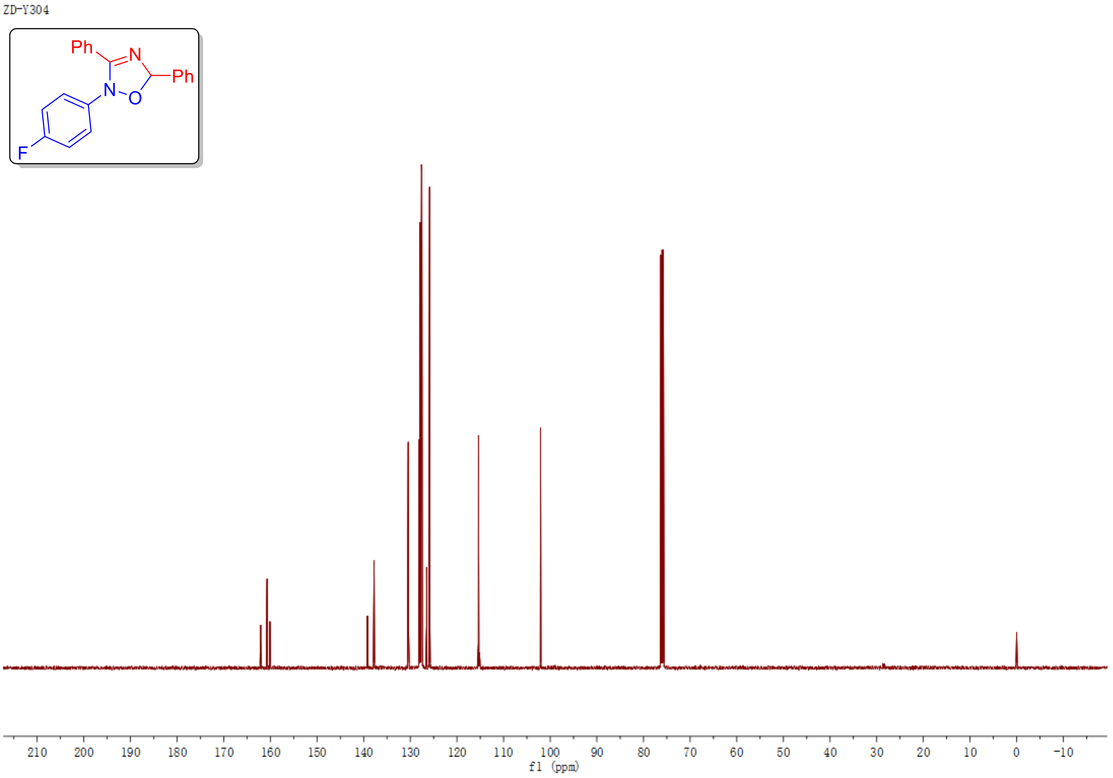

Supplementary Figure 109. ^{1}H NMR Spectrum of 3AA (500 MHz, $CDCl_3$)

ZD-Y270

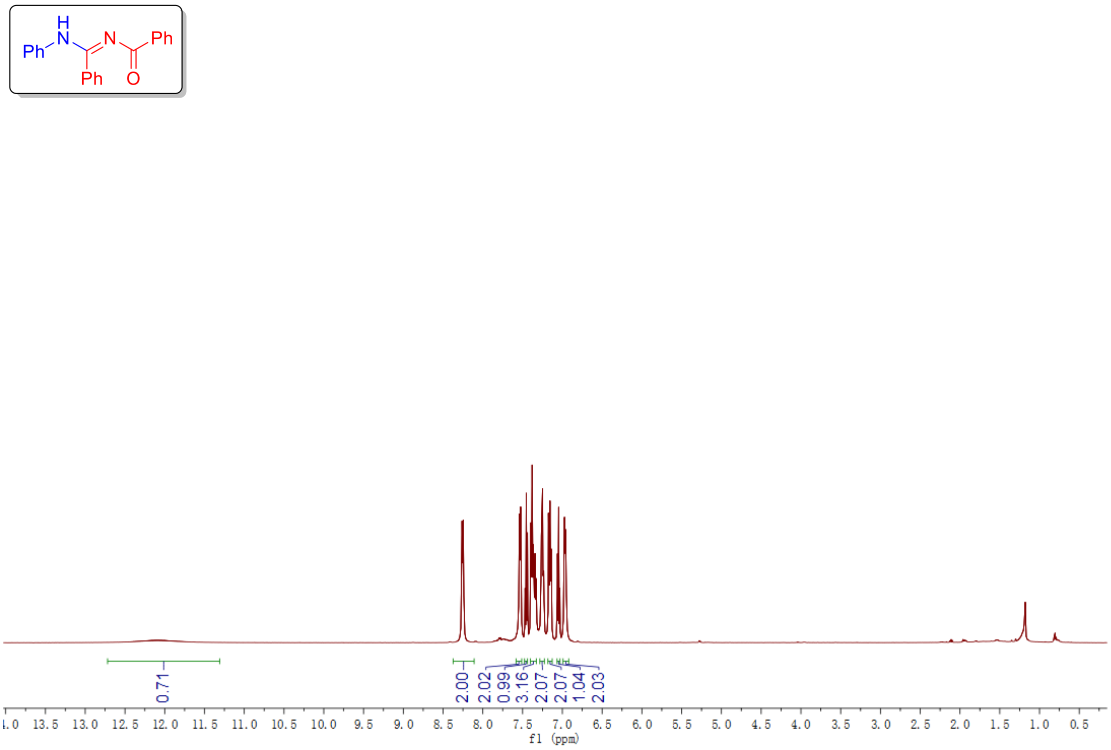

Supplementary Figure 110. ^{1}H NMR Spectrum of 3CA (500 MHz, $CDCl_3$)

ZD-Y306

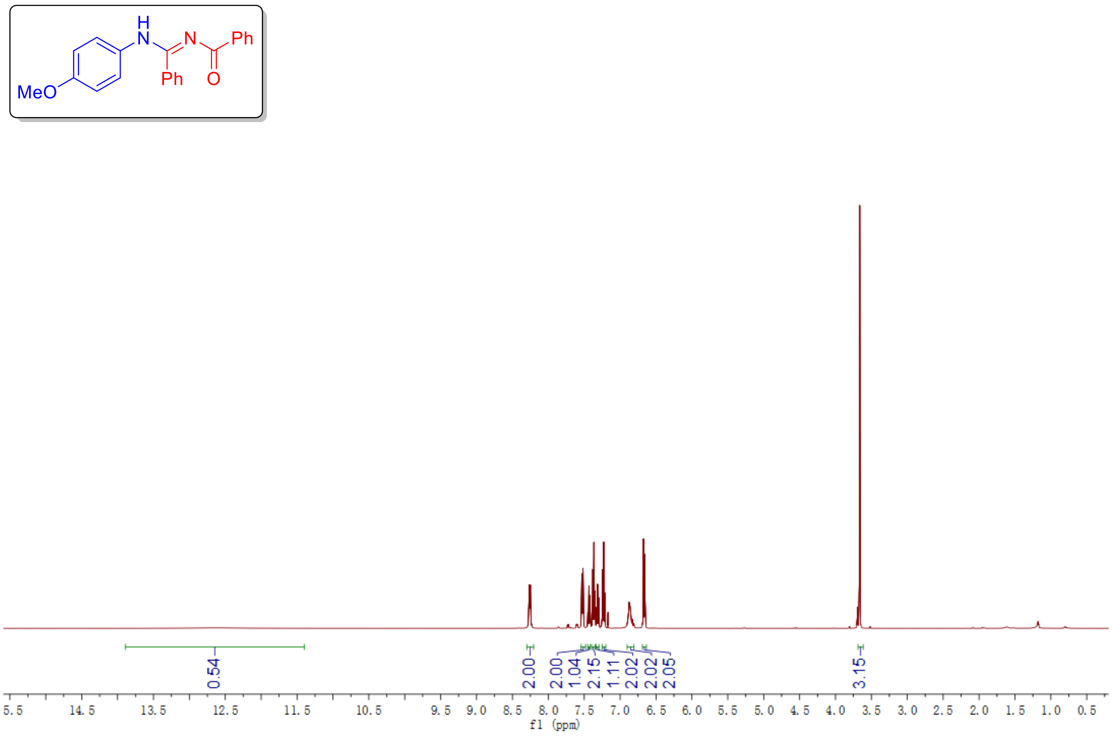

Supplementary Figure 111. ^{1}H NMR Spectrum of 3LA (500 MHz, $CDCl_3$)

ZD-Y295

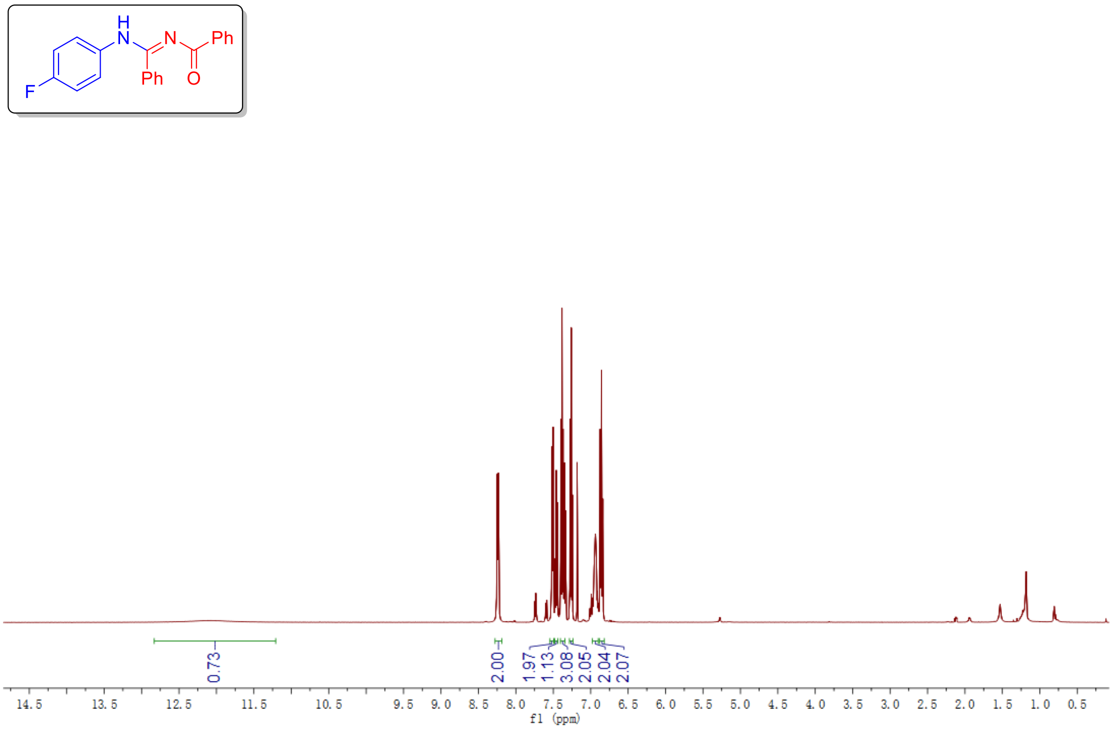

Supplementary Figure 112. ^{1}H NMR Spectrum of 3TA (500 MHz, $CDCl_3$)

ZD-Y298

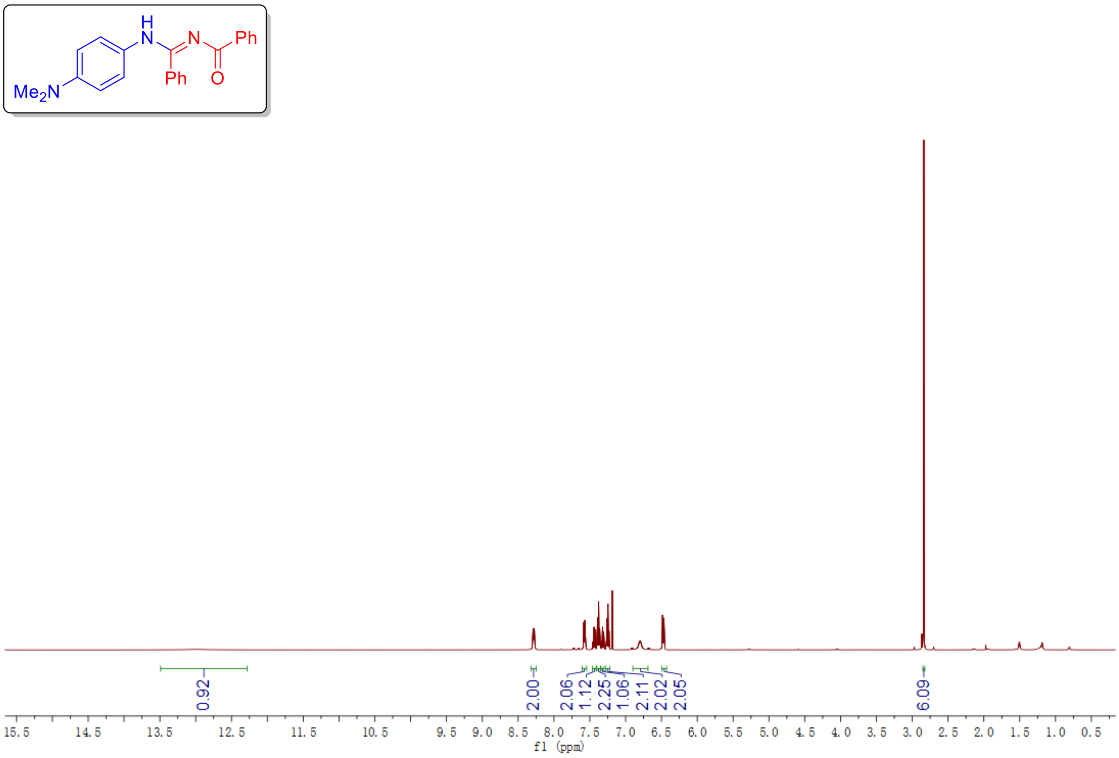

Supplementary Figure 113. ^{13}C NMR Spectrum of 3TA (125 MHz, $CDCl_3$)

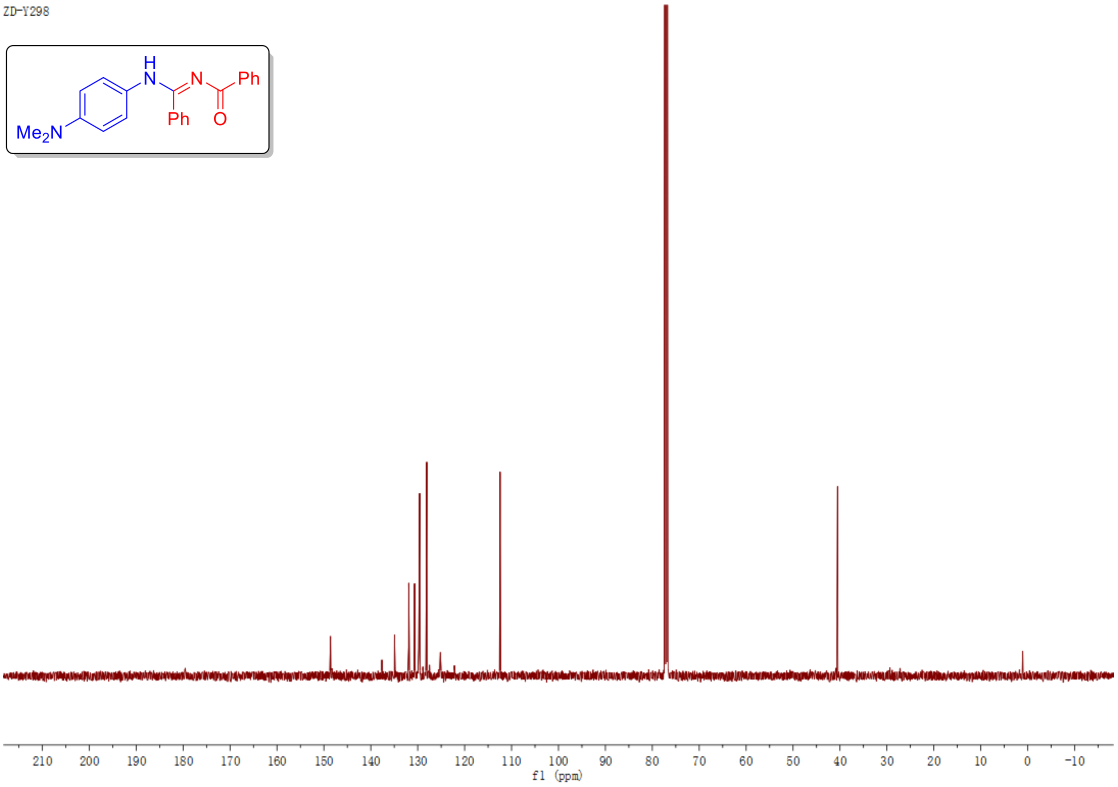

Supplementary Figure 114. The X-ray structure of 3aa.

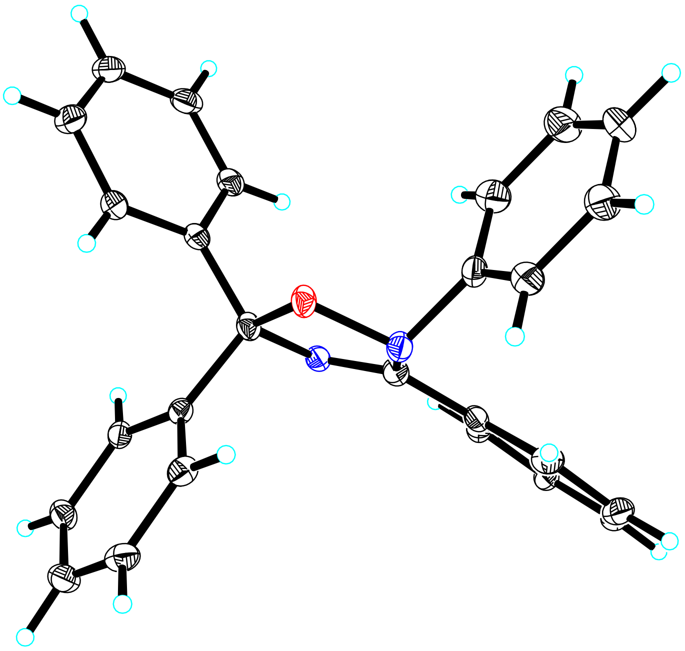

Supplementary Figure 115. The X-ray structure of 3aE.

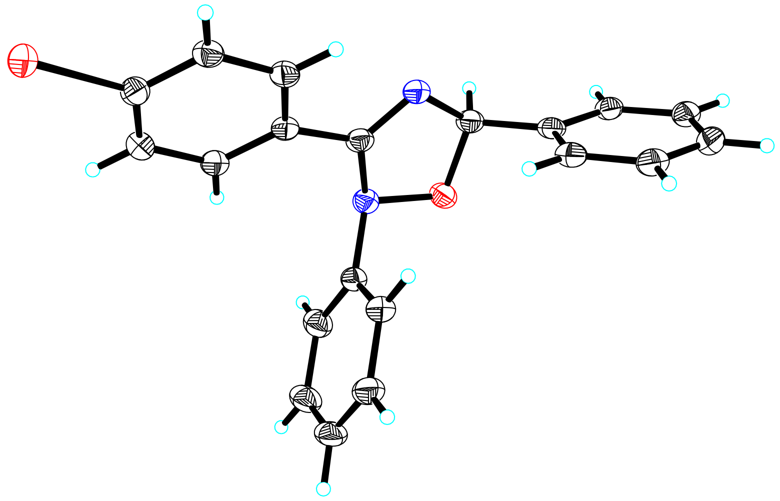

Supplement: Supplementary file 1 — Supplementary Information [file 41467_2021_26767_MOESM1_ESM.pdf]
